# Macrocyclic geminal diols: synthesis, structures, stability and photophysical properties†

**DOI:** 10.1039/d5sc08216a

**Published:** 2025-11-12

**Authors:** Bo Zou, Xiaolin Chen, Haoran Liu, Sijie Wen, Jieqing Huang, Hengshan Wei, Jinqing Huang, Yucheng Gu, Bingjia Xu, Jun Fan, Hua-Wei Jiang

**Affiliations:** a School of Chemistry, South China Normal University Guangzhou 510006 China fanj@scnu.edu.cn hwjiang@m.scnu.edu.cn; b Department of Chemistry, The Hong Kong University of Science and Technology Clear Water Bay, Kowloon Hong Kong China; c Syngenta Jealott's Hill International Research Centre Bracknell Berkshire UK; d School of Environmental and Chemical Engineering, Wuyi University Jiangmen 529020 China bingjiaxu@m.scnu.edu.cn

## Abstract

Geminal diols are generally unstable and prone to dehydration, yielding carbonyl compounds and making their isolation as discrete species highly challenging. Herein, we report the synthesis, structural characterization, and stability of a series of crystalline, stable, and rigid macrocyclic *gem*-diols obtained *via* acid hydrolysis of macrocyclic ketal precursors at −25 °C. Single-crystal X-ray diffraction analysis of these compounds reveals O–C–O bond angles of approximately 111°, along with extensive hydrogen-bonding networks that contribute to stabilizing the *gem*-diols. Thermogravimetric and hydrolytic analyses reveal a pronounced size-dependent stability trend. Theoretical calculations indicate that the enhanced stability of smaller *gem*-diol macrocycles stems from their ability to relieve substantial angle strain *via* sp^3^ hybridization at the methylene carbon, an effect that diminishes as ring size increases. Based on our experimental and computational results, the inner angle value of the macrocyclic ketone is proposed as a criterion for evaluating the relative stability of macrocyclic ketones *versus* their *gem*-diol forms. The photophysical properties of the macrocyclic geminal diols and macrocyclic diketones are also examined. This work broadens the scope of stable geminal diols and provides fundamental insights into their structure–stability relationships, thereby laying the groundwork for the strategic design and synthesis of structurally diverse macrocyclic geminal diols.

## Abstract 2

New rigid, stable, crystalline macrocyclic *gem*-diols were synthesized, and their stability was found to depend on ring size: the *gem*-diol form is favored in smaller rings, whereas the diketone form is more stable in larger ones.

## Graphical abstract

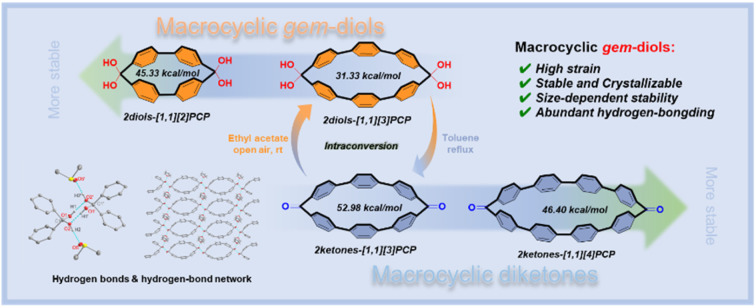

## Introduction

**Fig. 1:**
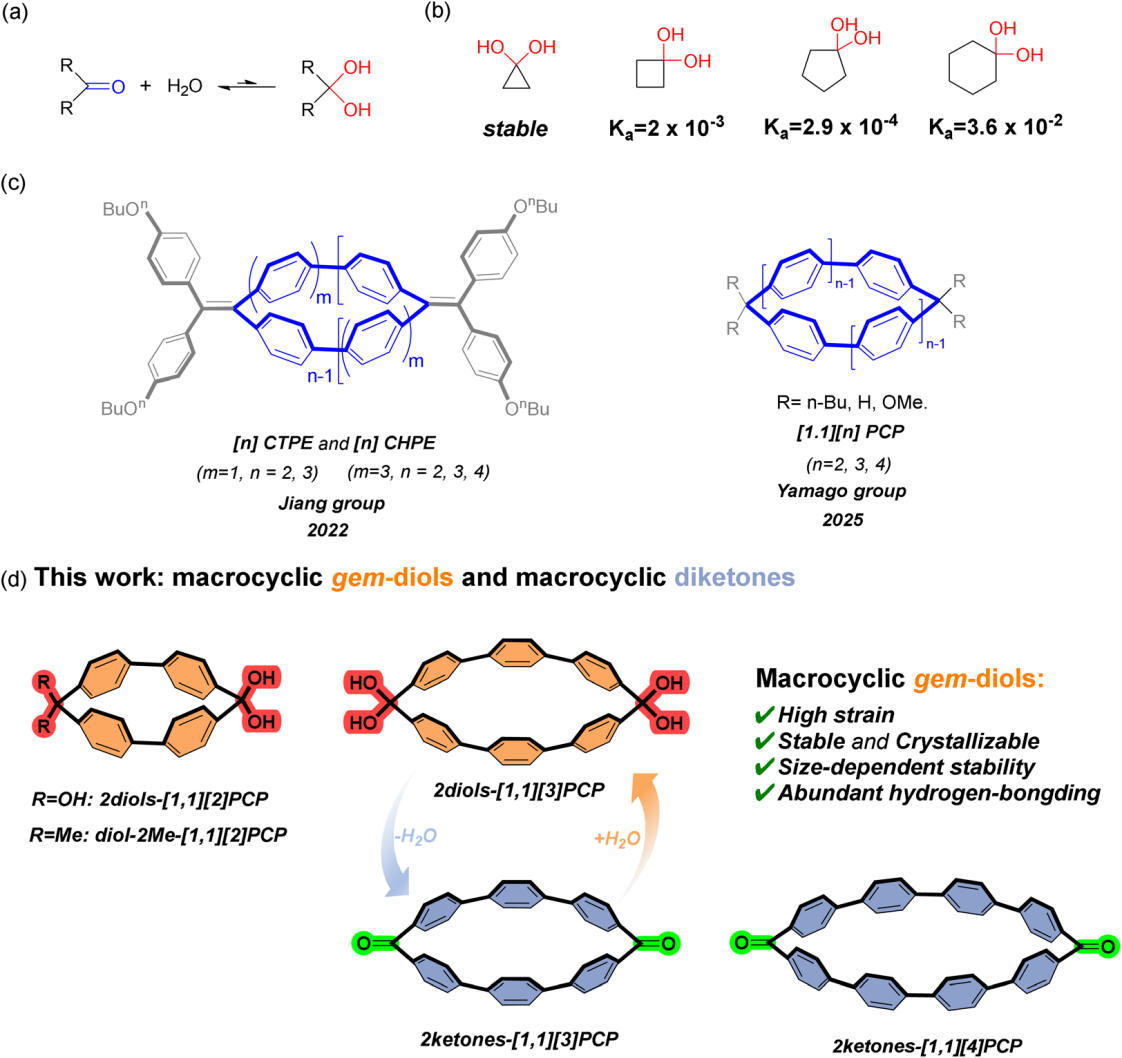
(a) The reversible hydration equilibrium between carbonyl compounds and geminal diols. (b) 1,1-Cyclopropanediol, 1,1-cyclobutanediol, 1,1-cyclopentanediol and 1,1-cyclohexanediol, along with their dehydration equilibrium constants. (c) Reported examples of macrocyclic framework. (d) Structures of 2diols-[1,1][2]PCP, 2diols-[1,1][3]PCP, 2ketones-[1.1][3]PCP, 2ketones-[1.1][4]PCP and diol-2Me-[1,1][2]PCP.

A geminal diol (*gem*-diol) is defined as a diol in which two hydroxyl groups are bonded to the same carbon atom. These species are recognized as key intermediates in a variety of organic and biochemical transformations,^1–5^ as well as in atmospheric processes,^6–8^ and they hold considerable potential for applications in organic synthesis,^9^ environmental chemistry,^10^ biochemistry,^11,12^ and coordination chemistry.^13–20^*gem*-Diols readily undergo dehydration to yield carbonyl compounds, making them generally difficult to isolate (Fig. 1a). For example, methanediol—the simplest geminal diol—although known to exist predominantly as a stable species in equilibrium with formaldehyde in aqueous solution,^21^ was not successfully isolated in the gas phase until 2022 by Kaiser *et al.*^22^ The stability of geminal diols is primarily influenced by steric hindrance, hydrogen bonding, strong electron-withdrawing effects, and ring strain.^23–27^ For instance, acetone forms its hydrate less readily than formaldehyde owing to steric hindrance,^21^ while chloral hydrate^28^ and ninhydrin hydrate^29^ are stabilized by strong electron-withdrawing groups. Although ring strain is recognized as an important contributor to the stability of geminal diols, only a limited number of strain-stabilized examples have been reported to date. 1,1-Cyclopropanediol, the smallest cyclic diol, can be synthesized *via* hydration of cyclopropenone,^30,31^ and its stability is attributed to the substantial angle strain of the three-membered ring, which enables its isolation as a stable compound.^21,23^ However, 1,1-cyclobutanediol, 1,1-cyclopentanediol, and 1,1-cyclohexanediol can only be formed in small amounts at equilibrium with their corresponding ketones and water (Fig. 1b).^21,32^ Additionally, coordination of a metal ion to certain polycyclic compounds can increase strain and promote *gem*-diol formation.^33,34^ Recent years have witnessed substantial progress in the synthesis of strained macrocyclic organic compounds.^35–50^ Macrocyclic organic compounds, in contrast to aliphatic small-ring analogues, exhibit greater structural diversity and offer enhanced potential for rational design and functional modification. Therefore, investigating the synthesis, structures, and stability of strained macrocyclic diols is of considerable interest.

We previously accessed two series of rigid porphyrin wheel β–β connected and biphenylene-linked—*via* platinum-mediated cyclization, with the highest strain energies of each series reaching 77.4 kcal mol^−1^ and 49.3 kcal mol^−1^, respectively.^51,52^ One-dimensional porphyrin nanotubes were also constructed *via* hydrogen-bond-driven self-assembly of porphyrin wheels bearing carboxylic acid groups.^53^ In 2022, we first reported [*n*]CTPE and [*n*]CHPE—two rigid macrocycles composed of *para*-biphenyl units and ylidene linkers (Fig. 1c, left).^54^ [2]CTPE exhibited distinctive dual-state emission properties and a long solid-state fluorescence lifetime, whereas larger macrocycles displayed enhanced aggregation-induced emission relative to the TPE monomer, indicating size-dependent physicochemical behavior. We subsequently made considerable efforts to synthesize analogous biphenylene macrocycles with varied bridging units. During this period, in 2025, the Yamago group reported similar rigid macrocycles, [1.1][*n*]paracyclophanes ([1,1][*n*]PCPs) (Fig. 1c, right),^55^ composed of *para*-biphenyl units connected by methylene bridges, and confirmed the presence of through-space conjugation between biphenyl units within the macrocycles. Here, we report the synthesis of macrocyclic *gem*-diols (2diols-[1.1][2]PCP, 2diols-[1.1][3]PCP, diol-2Me-[1.1][2]PCP) *via* hydrolysis of the corresponding ketal precursors (2ketals-[1.1][2]PCP, 2ketals-[1.1][3]PCP, 2ketals-[1.1][4]PCP, ketal-2Me-[1.1][2]PCP). The structures of these macrocyclic *gem*-diols, as well as two macrocyclic diketones (2ketones-[1.1][3]PCP, 2ketones-[1.1][4]PCP), were elucidated. The thermal stability of these macrocyclic *gem*-diols was investigated using thermogravimetric analysis (TGA) and further evaluated through theoretical calculations.

## Results and discussion

### Synthesis of macrocyclic *gem*-diols and macrocyclic diketones

The precursors of the 2ketals-[1.1][*n*]PCPs were synthesized through platinum-mediated cyclization followed by reductive elimination (see SI). The macrocyclic *gem*-diol 2diols-[1.1][2]PCP was synthesized by subjecting 2ketals-[1.1][2]PCP to an excess of trifluoroacetic acid (TFA) (Table 1). After complete conversion of 2ketals-[1.1][2]PCP, the acid was neutralized with a saturated aqueous sodium bicarbonate solution. The resulting mixture was filtered, and the crude product was recrystallized to yield the pure macrocyclic *gem*-diol. The acid hydrolysis conditions were optimized using 2ketals-[1.1][2]PCP as a model compound. It was found that TFA proved to be a suitable acid for the efficient hydrolysis of 2ketals-[1.1][2]PCP. Parallel reactions were performed over 30 minutes at 70 °C, 25 °C, 0 °C, and −25 °C (Table 1), and yields were determined by ^1^H NMR spectroscopy. At 70 °C, the desired *gem*-diol product was not obtained; instead, the starting material was completely converted into the ring-opening product 1, identified as 4′-([1,1′-biphenyl]-4-carbonyl)-[1,1′-biphenyl]-4-carboxylic acid. At 25 °C, the desired 2diols-[1.1][2]PCP was obtained in 43% yield together with product 1 in 57% yield, whereas at 0 °C, the yield of 2diols-[1.1][2]PCP increased to 87%, with 13% of 1 formed. At −25 °C,the desired product was obtained in 96% yield, with only 4% of 1 observed. The reaction time also affected the product distribution. Thin-layer chromatography showed incomplete conversion at 20 min, while full conversion was achieved at 30 min with the highest yield of 2diols-[1.1][2]PCP. Prolonged reaction times led to increased formation of byproduct 1. Thus, TFA at −25 °C for 30 min was selected as the optimal condition for hydrolysis, affording 2diols-[1.1][2]PCP in 78% isolated yield after recrystallization. The hydrolysis of 2dbts-[1.1][2]PCP, in which methylene carbons are connected to 1,4-dioxabutan-1,4-diyl units, did not proceed under the optimized conditions, likely due to the enhanced stability of the five-membered rings (see SI).

**Table 1:** Dehydration of 2diols-[1,1][2]PCP^a^

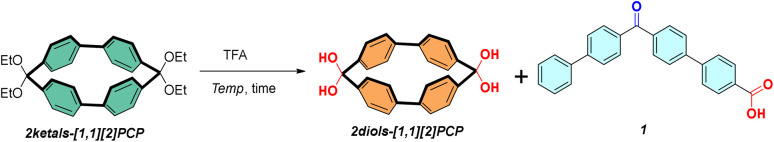				
Entry	Temp.	Time	Yield b of 2diols-[1.1][2]PCP	Yield b of 1
1	−25 °C	30 min	96% (78%)^c^	4%
2	0 °C	30 min	87%	13%
3	25 °C	30 min	43%	57%
4	70 °C	30 min	0%	100%

a All the reactions were carried out using 2ketals-[1.1][2]PCP (20 mg, 0.04 mmol) and trifluoroacetic acid (3 ml).

b Determined by ^1^H NMR spectroscopy unless otherwise noted.

c Isolated yield.

**Scheme 1:**
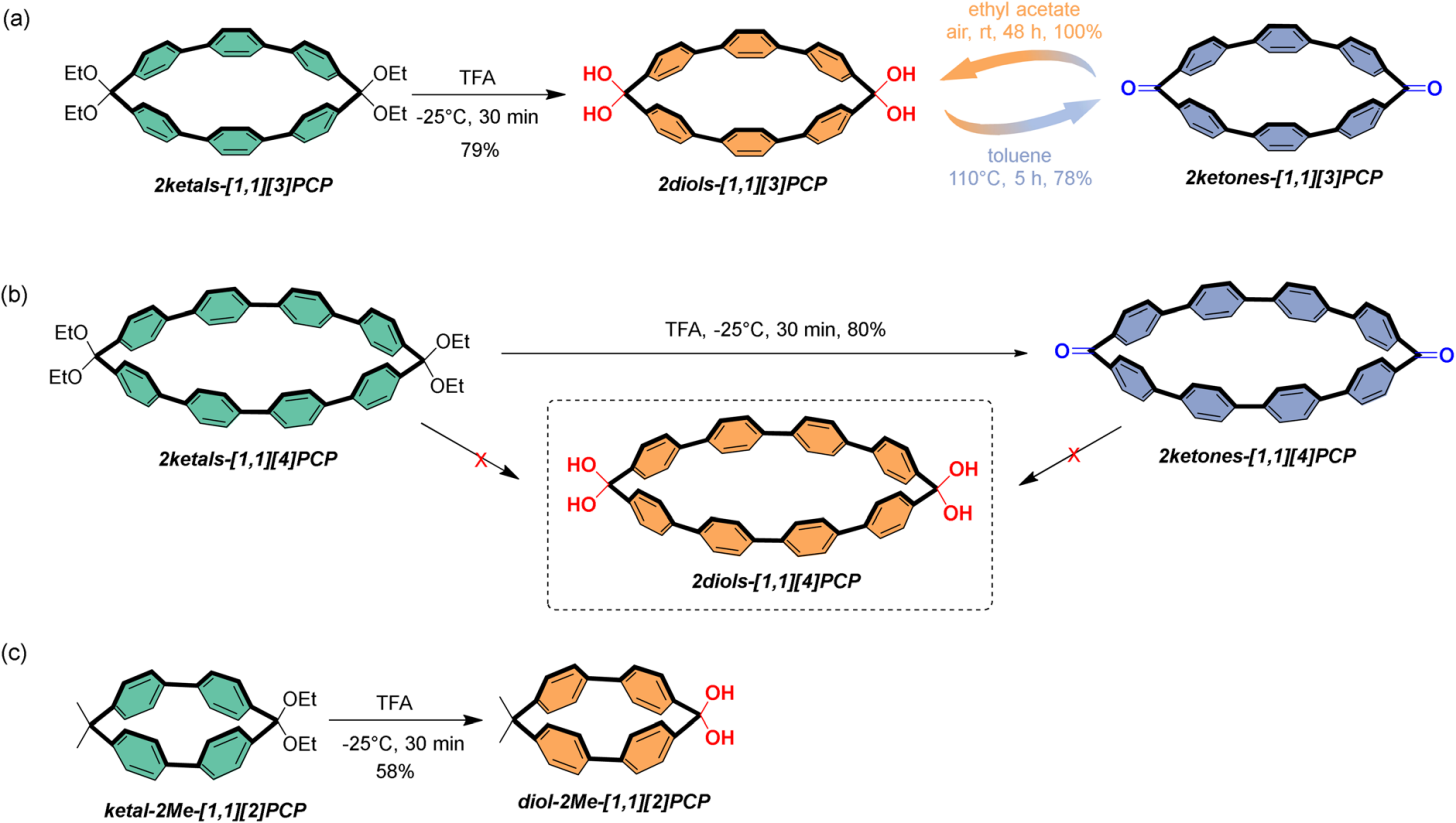
Synthesis of (a) 2diols-[1,1][3]PCP and 2ketones-[1,1][3]PCP, (b) 2ketones-[1,1][4]PCP (c) diol-2Me-[1,1][2]PCP.

Acid hydrolysis of the macrocyclic ketal precursors 2ketals-[1.1][3]PCP and ketal-2Me-[1.1][2]PCP under the optimized conditions afforded 2diols-[1.1][3]PCP and diol-2Me-[1.1][2]PCP in isolated yields of 79% and 58%, respectively (Scheme 1a and 1c). However, hydrolysis of 2ketals-[1.1][4]PCP afforded the macrocyclic diketone 2ketones-[1.1][4]PCP in 80% isolated yield, and no *gem*-diol product was detected (Scheme 1b). The transformation of 2diols-[1.1][2]PCP and 2diols-[1.1][3]PCP into their respective ketone counterparts was then attempted by heating in toluene under reflux for 5 h. While 2diols-[1.1][2]PCP remained unchanged, 2diols-[1.1][3]PCP was successfully converted into 2ketones-[1.1][3]PCP (78% isolated yield) (Scheme 1a). The characterization data of 2ketones-[1.1][3]PCP were consistent with those reported by Yamago group.^56^ Interestingly, when the ethyl acetate solution of 2ketones-[1.1][3]PCP was exposed to ambient air for 48 h, it quantitatively reverted to 2diols-[1.1][3]PCP (Scheme 1a). In contrast, 2ketones-[1.1][4]PCP remained unchanged under identical conditions (Scheme 1b).

### Structure of macrocyclic *gem*-diols and macrocyclic diketones

The ^1^H NMR spectra of 2diols-[1.1][2]PCP and diol-2Me-[1.1][2]PCP each exhibited a singlet at *δ* = 6.86 ppm, attributable to the geminal diol protons (see SI, Fig. S1a and S1c). The geminal diol carbon in both compounds appeared at *δ* = 97.48 ppm in the ^13^C NMR spectrum (see SI, Fig. S32 and S36). For 2diols-[1.1][3]PCP, a similar singlet appeared at *δ* = 6.96 ppm in the ^1^H NMR spectrum (see SI, Fig. S1b), and the corresponding carbon resonance was detected at *δ* = 96.64 ppm in the ^13^C NMR spectrum. In contrast, the ^13^C NMR spectra of 2ketones-[1.1][3]PCP and 2ketones-[1.1][4]PCP exhibited resonances at *δ* = 201.06 ppm and *δ* = 200.83 ppm, respectively, which were assigned to the carbonyl carbons. MALDI-TOF mass spectra of the three macrocyclic geminal diols (2diols-[1.1][2]PCP, 2diols-[1.1][3]PCP, and diol-2Me-[1.1][2]PCP) and two macrocyclic diketones (2ketones-[1.1][3]PCP and 2ketones-[1.1][4]PCP) showed molecular ion peaks at *m*/*z* = 396.138, *m*/*z* = 548.158, *m*/*z* = 392.176, *m*/*z* = 513.138 and *m*/*z* = 664.247, in agreement with their calculated values of C_26_H_20_O_4_^+^ [M]^+^: 396.136, C_38_H_28_O_4_^+^ [M + H]^+^: 548.199, C_28_H_24_O_2_^+^ [M]^+^: 392.178, C_38_H_25_O_2_^+^ [M + H]^+^: 513.184 and C_50_H_32_O_2_^+^ [M]^+^: 664.240, respectively.

**Fig. 2:**
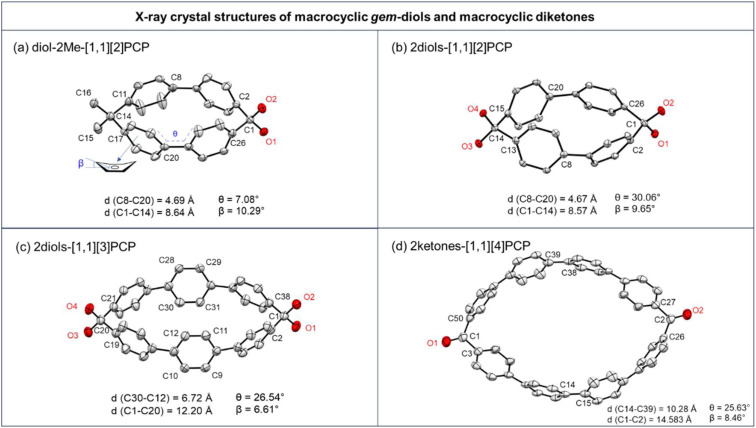
X-ray crystal structures of macrocyclic *gem*-diols (a) diol-2Me-[1,1][2]PCP, (b) 2diols-[1,1][2]PCP, (c) 2diols-[1,1][3]PCP, and macrocyclic diketones (d) 2ketones-[1,1][4]PCP. Carbon atoms are numbered in black according to the molecular symmetry. Hydrogen atoms and solvents are omitted for clarity. The thermal ellipsoids are scaled at the 50 % probability level. The average bending angles of the phenyls are defined as *β*. The average torsion angles are defined as *θ*.

To gain further insight into the structural characteristics of these macrocyclic geminal diols, single crystals were grown of 2diols-[1.1][2]PCP, 2diols-[1.1][3]PCP, and diol-2Me-[1.1][2]PCP, as well as the macrocyclic diketone 2ketones-[1.1][4]PCP and the ketal precursors ketal-2Me-[1.1][2]PCP, 2ketals-[1.1][2]PCP, 2ketals-[1.1][3]PCP, and 2ketals-[1.1][4]PCP. Their structures were unambiguously confirmed by single-crystal X-ray diffraction analysis (Fig. 2).

Each molecule exhibited an elliptical cavity, with the cavity size increasing as the ring size expanded. The exocyclic O–C–O bond angle (*α*_1_) in 2diols-[1.1][2]PCP (111.74°) is slightly larger than that in 2diols-[1.1][3]PCP (110.55°), whereas the endocyclic C–C–C angle (*α*_2_) in 2diols-[1.1][2]PCP (101.20°) is about 3° smaller than that in 2diols-[1.1][3]PCP (104.03°), indicating greater angular strain in the former due to its more constrained ring size. The *α*_2_ in 2diols-[1.1][2]PCP and 2diols-[1.1][3]PCP are close to those observed in 2ketals-[1.1][2]PCP (101.11°) and 2ketals-[1.1][3]PCP (103.44°), suggesting that the overall macrocyclic strain remains nearly unchanged before and after hydrolysis. For 2diols-[1.1][2]PCP, 2diols-[1.1][3]PCP, and diol-2Me-[1.1][2]PCP, the C–O bond lengths of the geminal hydroxyl groups (1.41–1.42 Å) are longer than those in hexafluoroacetone hydrate (1.38 Å), confirming that the stabilization of these macrocyclic geminal diols arises from ring strain rather than electron effects.^57^ In diol-2Me-[1.1][2]PCP, the intramolecular O⋯O distance (2.33 Å) and exocyclic O–C–O angle (111.05°) are nearly identical to those in 2diols-[1.1][2]PCP, indicating that despite its lower symmetry, the geminal diol geometry remains essentially unchanged.

**Fig. 3:**
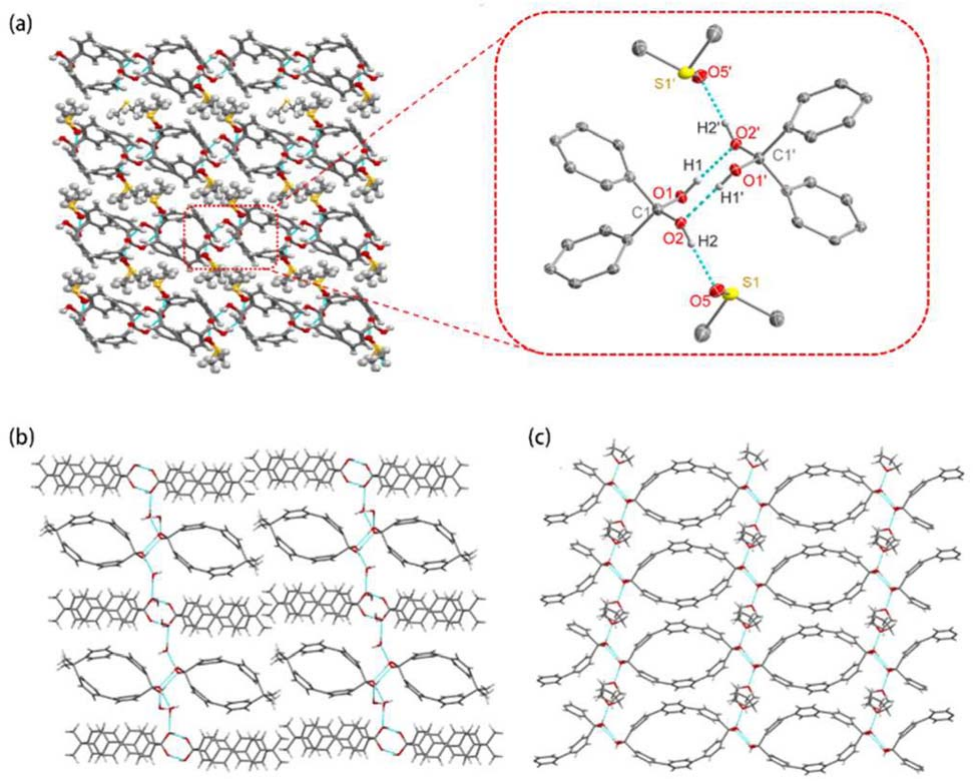
(a) Hydrogen-bonded network with eight-membered ring structures in 2diols-[1,1][2]PCP·2DMSO and the thermal ellipsoids are scaled at the 50 % probability level. Hydrogen-bonded network of (b) diol-2Me-[1,1][2]PCP·H_2_O and (c) 2diols-[1,1][3]PCP·2THF.

Geminal diols can easily form hydrogen bonds with solvent molecules,^58^ which may contribute to their stabilization. Extensive hydrogen-bonding networks are observed in the macrocyclic geminal diol 2diols-[1.1][2]PCP (Fig. 3a). The hydroxyl groups of the geminal diol moieties form eight-membered ring structures through intermolecular hydrogen bonding with adjacent macrocycles. In addition, one hydroxyl hydrogen of the geminal diol moiety forms a hydrogen bond with an oxygen atom of the solvent molecule dimethyl sulfoxide. These intermolecular hydrogen bonds (O(1)–H(1)⋯O(2′) and O(1′)–H(1′)⋯O(2)) exhibit identical O⋯O distances of 2.74 Å. Hydrogen bonds are observed between 2diols-[1.1][2]PCP and dimethyl sulfoxide solvent molecules, with O⋯O distances of 2.64 Å (O(2)–H(2)⋯O(5) and O(2′) –H(2′)⋯O(5′)). Similar hydrogen-bonding networks are also observed in 2diols-[1.1][3]PCP and diol-2Me-[1.1][2]PCP (Fig. 3b and c). It is worth mentioning that while 2diols-[1.1][2]PCP contains two geminal diol moieties, diol-2Me-[1.1][2]PCP contains only one. As a result, the density of the hydrogen-bonding network in diol-2Me-[1.1][2]PCP is lower, which may contribute to its reduced structural stability compared to 2diols-[1.1][2]PCP.

For the macrocyclic diketone 2ketones-[1.1][4]PCP, the distance between the oxygen atom and its closest carbon atom is 1.22 Å, consistent with a typical carbonyl double bond. The bond angles C(3)–C(1)–C(50) and C(26)–C(2)–C(27) are 112.84° and 113.19°, respectively, which are significantly smaller than the corresponding angle in benzophenone (116.90°),^59^ indicating considerable ring strain. Upon conversion from 2ketals-[1.1][4]PCP to 2ketones-[1.1][4]PCP, the hybridization of the bridging carbon changes from sp^3^ to sp^2^ due to the formation of carbonyl groups. This transformation leads to a substantial increase in the biphenyl dihedral angle *α* from 106.01° to 112.92°, as well as an increase in the bending angle *β* of the internal phenyl units from 5.47° to 8.46°. These changes indicate that the elliptical cavity of 2ketones-[1.1][4]PCP has a smaller eccentricity than that of 2ketals-[1.1][4]PCP, suggesting that 2ketones-[1.1][4]PCP accommodates greater ring strain (Table 2).

**Table 2:** X-ray single-crystal diffraction data of macrocyclic compounds

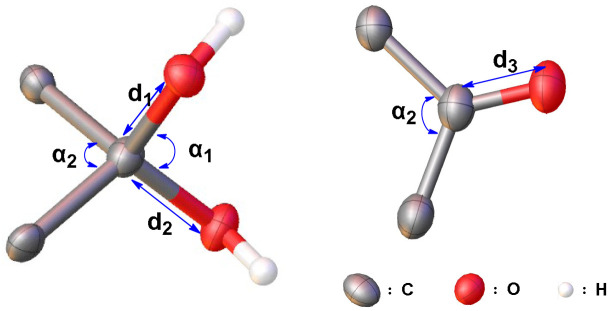					
Compound	*d*_1_ a (Å)	*d*_2_ b (Å)	*d*_3_ c (Å)	*α*_1_ d (°)	*α*_2_ e (°)
diol-2Me-[1,1][2]PCP	1.41	1.41	—f	111.05	101.45
2diols-[1,1][2]PCP	1.41	1.41	—f	111.74	101.20
2diols-[1,1][3]PCP	1.42	1.41	—f	110.55	104.03
2ketones-[1,1][4]PCP	—f	—f	1.22	—f	113.19

a Define as the C–O bond lengths *d*_1_.

b Define as the C–O bond lengths *d*_2_.

c The C=O bond lengths of the carbonyl group in the macrocyclic diketones.

d Define *α*_1_ as the exocyclic O–C–O bond angle.

e Define *α*_2_ as the intracyclic C–C–C bond angle.

f Not applicable.

### Thermal stability of the macrocyclic *gem*-diols

**Fig. 4:**
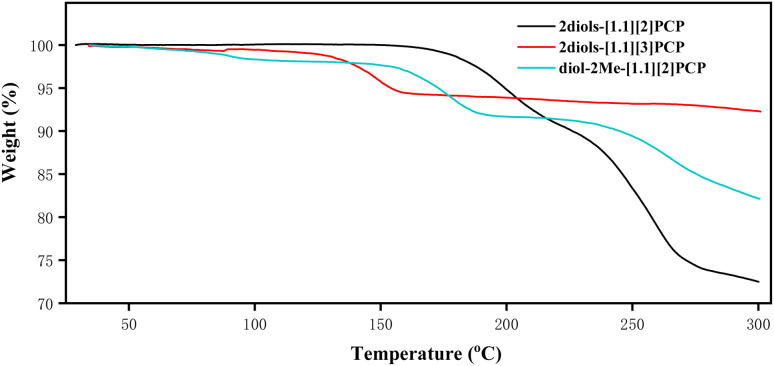
Thermogravimetric analysis of 2diols-[1,1][2]PCP, 2diols-[1,1][3]PCP and diol-2Me-[1,1][2]PCP, measured under nitrogen atmosphere.

The thermal stability of 2diols-[1.1][2]PCP, 2diols-[1.1][3]PCP, and diol-2Me-[1.1][2]PCP was evaluated by thermogravimetric analysis (TGA). The powders of 2diols-[1.1][2]PCP, 2diols-[1.1][3]PCP, and diol-2Me-[1.1][2]PCP used for TGA were prepared by recrystallization from ethyl acetate/*n*-hexane, followed by vacuum drying for 24 hours. As shown in Fig. 4, 2diols-[1.1][2]PCP (black line) exhibited no weight loss until 150 °C. Then, between 150 °C and 210 °C, an approximate 8% weight loss was observed, corresponding to the release of two water molecules. Upon continued heating above 205 °C, the sample gradually underwent complete decomposition. The results implied that, although 2diols-[1.1][2]PCP could dehydrate and likely form 2ketones-[1.1][2]PCP, the latter is not sufficiently stable to be isolated. This observation is consistent with the results of the heating experiments on 2diols-[1.1][2]PCP mentioned above. For diol-2Me-[1.1][2]PCP (Fig. 4, cyan line), an initial approximate 2% weight loss from 30 °C to 110 °C was observed, which can be attributed to the loss of solvent molecules engaged in hydrogen bonding with the geminal diols. Between 140 °C and 190 °C, an approximate 6% weight loss may be attributed to the release of a water molecule. In the case of 2diols-[1.1][3]PCP (Fig. 4, green line), a weight loss of approximately 4% between 100 °C and 160 °C corresponds to the elimination of two water molecules. Above 160 °C, the TGA curve exhibited a distinct plateau, indicating the formation of 2ketones-[1.1][3]PCP. 2ketones-[1.1][3]PCP remained thermally stable over a broad temperature range, consistent with the dehydration experiments described in the synthesis section.

TGA measurements indicate that 2diols-[1.1][2]PCP is more thermally stable than diol-2Me-[1.1][2]PCP, which may be attributed to the more extensive intermolecular hydrogen-bonding network present in 2diols-[1.1][2]PCP. 2diols-[1.1][2]PCP and diol-2Me-[1.1][2]PCP exhibit greater thermal stability than 2diols-[1.1][3]PCP. These results, along with the observation that acid hydrolysis of 2ketals-[1.1][4]PCP did not yield the macrocyclic geminal diol product, suggest that the stability of macrocyclic geminal diols depends on ring size; specifically, increasing the ring size leads to reduced thermal stability. This trend is likely attributed to the reduction of angular strain in larger macrocycles, which renders the geminal diol functionality less thermodynamically favored.

### DFT calculation

To further elucidate the structure–stability relationships, gas-phase geometry optimizations and strain energy calculations^60–62^ were performed at the WB97XD/6-311G(d) level for macrocyclic *gem*-diols—including diol-2Me-[1,1][2]PCP and 2diols-[1,1][*n*]PCPs—their corresponding macrocyclic diketones (ketone-2Me-[1,1][2]PCP and 2ketones-[1,1][*n*]PCPs), and the respective macrocyclic ketals (ketal-2Me-[1,1][2]PCP and 2ketals-[1,1][*n*]PCPs) (See SI, Fig. S74). The optimized core structures are in good agreement with the corresponding single-crystal X-ray structures (Table 3).

**Table 3:** Summary of the physical properties of macrocyclic *gem*-diols

Entry	Compound	Strain energy (kcal mol^−1^)	The inner angle (°)
1	ketal-2Me-[1,1][2]PCP	50.29	100.97
	diol-2Me-[1,1][2]PCP	45.96	101.86
	ketone-2Me-[1,1][2]PCP	57.51	104.11
2	2ketals-[1,1][2]PCP	43.89	100.90
	2diols-[1,1][2]PCP	45.33	101.82
	2ketones-[1,1][2]PCP	68.94	104.23
3	2ketals-[1,1][3]PCP	29.64	102.74
	2diols-[1,1][3]PCP	31.33	103.65
	2ketone-[1,1][3]PCP	52.98	108.58
4	2ketals-[1,1][4]PCP	25.70	104.10
	2diols-[1,1][4]PCP	26.12	105.96
	2ketones-[1,1][4]PCP	46.40	112.71

The strain energies of 2diols-[1.1][2]PCP, 2diols-[1.1][3]PCP, and 2diols-[1.1][4]PCP were calculated to be 45.33, 31.33, and 26.12 kcal mol^−1^, respectively, whereas those of 2ketones-[1.1][2]PCP, 2ketones-[1.1][3]PCP, and 2ketones-[1.1][4]PCP were 68.94, 52.98, and 46.40 kcal mol^−1^, respectively. In all three comparisons the strain energies of the *gem*-diol forms are more than 20 kcal mol^−1^ lower than those of the corresponding ketone forms. The strain energy of diol-2Me-[1.1][2]PCP is 11.55 kcal mol^−1^ lower than that of ketone-2Me-[1.1][2]PCP; this difference is approximately half of the aforementioned strain energy gaps, consistent with the fact that diol-2Me-[1.1][2]PCP contains only one *gem*-diol moiety. The strain energies of the macrocyclic ketals are similar to those of the corresponding macrocyclic *gem*-diols but are lower than those of the corresponding macrocyclic diketones.

The calculated internal angles for 2diols-[1.1][2]PCP, 2diols-[1.1][3]PCP, and 2diols-[1.1][4]PCP are 101.82°, 103.65°, and 105.96°, respectively, which are smaller than those of the corresponding diketones (2ketones-[1.1][2]PCP, 2ketones-[1.1][3]PCP, 2ketones-[1.1][4]PCP: 104.23°, 108.58°, 112.71°, respectively). The calculated internal angles of the ketals (2ketals-[1.1][2]PCP, 2ketals-[1.1][3]PCP, 2ketals-[1.1][4]PCP) are 101.86°, 102.74°, and 104.10°, respectively, showing an increasing trend with macrocycle size that aligns well with single-crystal X-ray data. On the basis of these computational and experimental results, we propose that the calculated internal angle of macrocyclic diketones can be used as a predictive indicator of relative stability: for internal angles ≈ 108° both ketone and *gem*-diol forms may be thermodynamically accessible; for internal angles ≤ 104° the *gem*-diol form is strongly favored; and for internal angles ≥ 112° the ketone form is strongly favored.

We compared the strain distribution of 2diols-[1,1][*n*]PCPs (*n* = 2, 3, 4) and 2ketones-[1,1][*n*]PCPs (*n* = 2, 3, 4) by strain visualization using StrainViz.^63^ For 2diols-[1,1][*n*]PCPs (*n* = 2, 3, 4) and 2ketones-[1,1][*n*]PCPs, the highest strain energy is located at the C_phenyl_–C_*gem*-diol_ and C_phenyl_–C_carbonyl_ bonds, respectively (see SI, Fig. S84). The highest strain energies in 2ketones-[1,1][*n*]PCPs are approximately 1.5 to 2.5 times higher than those in the corresponding 2diols-[1,1][*n*]PCPs.

These results indicate that an increase in macrocycle size leads to a pronounced decrease in ring strain energy, while dehydration of *gem*-diols to the corresponding sp^2^-hybridized carbonyl compounds significantly increases the ring strain energy across different macrocycle sizes. Thus, from the perspective of ring strain energy, the formation of geminal diols within strained macrocyclic frameworks enhances stability.

### Photophysical properties

**Fig. 5:**
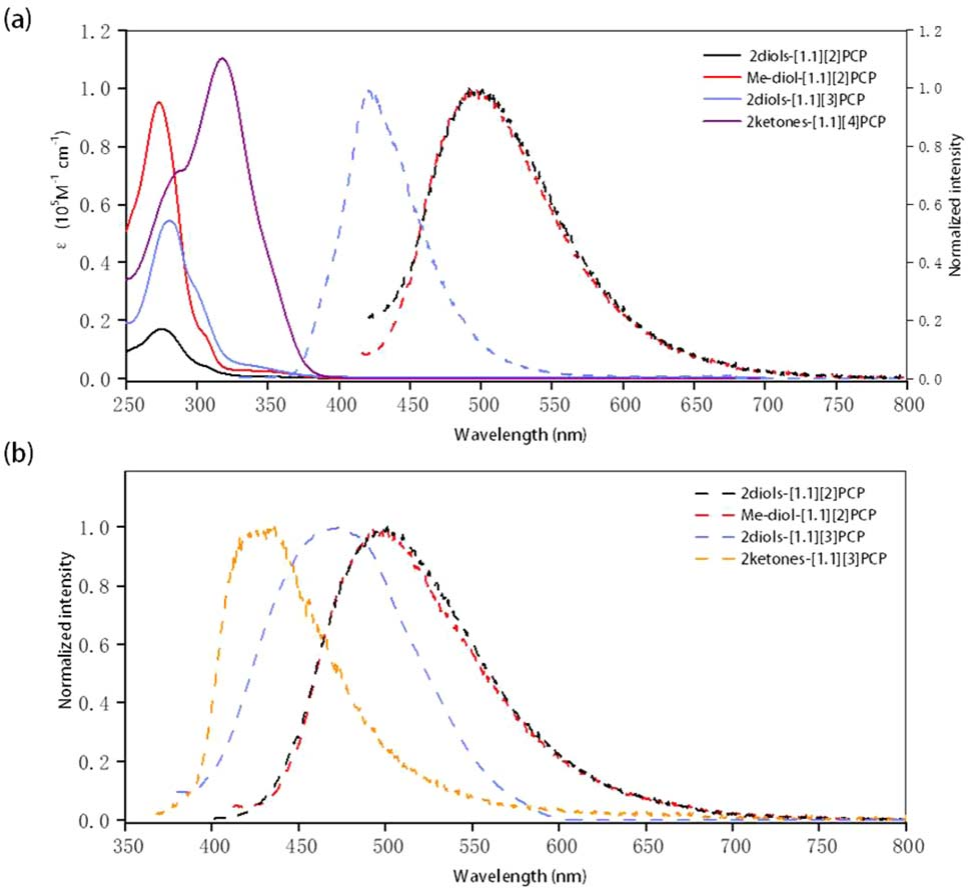
(a) UV-visible absorption (solid line) and fluorescence spectra (dashed line) of 2Me-diol-[1,1][2]PCP, 2diols-[1,1][2]PCP, 2diols-[1,1][3]PCP and 2ketones-[1,1][4]PCP in THF. (b) PL spectra of 2Me-diol-[1,1][2]PCP, 2diols-[1,1][2]PCP, 2diols-[1,1][3]PCP and 2ketones-[1,1][3]PCP in solid state.

The UV-vis absorption properties of these macrocycles in dilute THF solution were investigated, and the results are presented in Fig. 5, Tables 4 and S24. The absorption bands of the macrocyclic *gem*-diols, attributed to π–π* electronic transitions, were observed in the range of 273 to 280 nm. A distinct redshift in the maximum absorption wavelengths was observed with increasing macrocycle size, which is likely due to the extended conjugation of biphenyl units as the ring size increases. For macrocyclic diketones, the maximum absorption wavelength (*λ*_max_) of 2ketones-[1.1][4]PCP was measured to be 317 nm. However, due to the rapid conversion of 2ketones-[1.1][3]PCP to 2diols-[1.1][3]PCP in THF, the photophysical properties of 2ketones-[1.1][3]PCP could not be obtained in this solvent.

**Table 4:** Summary of the photophysical properties of macrocyclic *gem*-diols and macrocyclic diketones

Compound	*λ*_abs_ a (nm)	*λ*_PL,s_ b (nm)	*λ*_PL,sl_ c (nm)	*Φ*_PL,s_ d (%)	*Φ*_PL,sl_ e (%)
diol-Me-[1,1][2]PCP	273	503.5	491.5	10.13	12.73
2diols-[1,1][2]PCP	274	501	491.5	17.74	6.92
2diols-[1,1][3]PCP	280	425.5	420.5	20.55	38.23
2ketones-[1,1][3]PCP	—	474	—	6.72	—
2ketones-[1,1][4]PCP	317	—	—	—	—

a UV-visible maximum absorption in THF solution.

b PL maximum in solid state.

c PL maximum in THF solution.

d Absolute photoluminescence quantum yield in solid state.

e Absolute photoluminescence quantum yield in THF solution.

These macrocyclic *gem*-diols exhibit strong fluorescence in both THF solution and the solid state. In THF solution, the maximum emission wavelengths (*λ*_PL,sl_) of 2diols-[1.1][2]PCP, diol-2Me-[1.1][2]PCP, and 2diols-[1.1][3]PCP were determined to be 491.5 nm, 491.5 nm, and 420.5 nm, respectively, comparable to those of their corresponding ketal precursors (see SI). A blueshift in emission was observed with increasing macrocycle size, likely due to the relaxation of ring strain. The photoluminescence quantum yields (*Φ*_PL,sl_) of 2diols-[1.1][2]PCP, diol-2Me-[1.1][2]PCP, and 2diols-[1.1][3]PCP in THF were 6.92%, 12.73%, and 38.23%, respectively. Similar to their behavior in THF, 2diols-[1.1][2]PCP, diol-2Me-[1.1][2]PCP, and 2diols-[1.1][3]PCP exhibit solid-state maximum emission wavelengths (*λ*_PL,s_) of 501 nm, 503.5 nm, and 425.5 nm, respectively. Their corresponding solid-state *Φ*_PL,s_ values were 17.74%, 10.13%, and 20.55%. For macrocyclic diketones, no visible emission was detected for 2ketones-[1.1][4]PCP. In contrast, 2ketones-[1.1][3]PCP exhibits solid-state emission with a maximum emission wavelength of 474 nm and a *Φ*_PL,s_ of 6.72%. Such shifts and changes in quantum yields may be attributed to alterations in conjugation length and ring strain, which modulate the electronic structures and non-radiative decay pathways of these macrocycles.

## Conclusion

In summary, a series of crystalline, stable, and rigid macrocyclic *gem*-diols and macrocyclic diketones were successfully synthesized *via* acid hydrolysis of macrocyclic ketals at −25 °C. Single-crystal X-ray diffraction analysis of 2diols-[1.1][2]PCP, 2diols-[1.1][3]PCP, and diol-2Me-[1.1][2]PCP reveals O–C–O bond angles of approximately 111°. Notably, these macrocycles form a robust eight-membered hydrogen-bonded structure in the solid state, which likely contributes to their ordered packing and enhanced stability. Thermogravimetric analysis reveals a clear, ring size-dependent thermal stability. 2diols-[1.1][2]PCP remains stable up to 150 °C, and diol-2Me-[1.1][2]PCP begins to dehydrate at around 140 °C. 2diols-[1.1][3]PCP starts to dehydrate at approximately 100 °C, whereas 2diols-[1.1][4]PCP could not be obtained under similar conditions. The strain energies of macrocyclic *gem*-diols are consistently lower than those of their dehydrated ketone counterparts. This difference can be attributed to the sp^3^-hybridized *gem*-diol carbon atoms, which better accommodate angular strain than the planar sp^2^-hybridized carbons in the ketones. We propose that the calculated inner angles of macrocyclic ketones can be used to assess the relative stability of macrocyclic ketones *versus* their *gem*-diol forms. The successful synthesis of this new class of macrocyclic geminal diols not only expands the library of stable *gem*-diol compounds but also provides deeper insights into the structural features and stability factors of this important functional group, thereby laying a solid foundation for the design and synthesis of more diverse macrocyclic *gem*-diol molecules.

## Author contributions

H.-W. Jiang initiated and designed the project. Supervision of the research was provided by H.-W. Jiang, B. Xu, and J. Fan. B. Zou performed the primary experiments and conducted corresponding data analysis. X. Chen contributed to part of the experimental work. H. Wei was responsible for the majority of the photophysical measurements. H. Liu, S. Wen, and J. Huang aided in data collection and analysis. The initial draft of the manuscript was prepared by B. Zou. All authors participated in the discussion and revision of the manuscript.

## Conflicts of interest

The authors declare no competing financial interests.

## Supplementary Material

SC-OLF-D5SC08216A-s001

SC-OLF-D5SC08216A-s002

## Data Availability

CCDC 2479706 (diol-2Me-[1.1][2]PCP), 2479718 (2diols-[1.1][2]PCP), 2479720 (2diols-[1.1][3]PCP), 2479725 (2ketones-[1.1][4]PCP), 2479728 (2dbts-[1.1][2]PCP), 2479740 (ketal-2Me-[1.1][2]PCP), 2479742 (2ketals-[1.1][4]PCP), 2479743 (2ketals-[1.1][2]PCP) and 2479744 (2ketals-[1.1][3]PCP) contain the supplementary crystallographic data for this paper.^64*a*–*i*^ Supplementary information: experimental procedures, characterization data for compounds, NMR spectra, thermogravimetric analysis, and X-ray crystallographic data. See DOI: https://doi.org/10.1039/d5sc08216a.
