# Supplementary material for: Macrocyclic geminal diols: synthesis, structures, stability and photophysical properties
Source: Chem Sci. 2025 Nov 12;17(2):1127–36. doi: 10.1039/d5sc08216a (PMC12628340; doi:10.1039/d5sc08216a)
Supplement: SC-017-D5SC08216A-s001 [file SC-017-D5SC08216A-s001.pdf]

Supplementary Information (SI) for Chemical Science.
This journal is © The Royal Society of Chemistry 2025

Supporting Information for:

Macrocyclic Geminal Diols: Synthesis, Structures, Stability and Photophysical Properties

Bo Zou, [a] Xiaolin Chen, [a] Haoran Liu, [a] Sijie Wen, [a] Jieqing Huang, [a] Hengshan Wei, [a] Jinqing Huang, [b] Yucheng Gu, [c] Bingjia Xu, * [d] Jun Fan* [a] and Hua-Wei Jiang* [a]

a. School of Chemistry, South China Normal University, Guangzhou 510006, P. R. China; E-mail: fanj@scnu.edu.cn; jiang@m.scnu.edu.cn.
b. Department of Chemistry, Hong Kong University of Science and Technology, Clear Water Bay, Hong Kong, China
c. Syngenta Jealott's Hill International Research Centre, Bracknell, Berkshire, UK
d. School of Environmental and Chemical Engineering, Wuyi University, Jiangmen 529020, P. R. China; E-mail: bingjiaxu@m.scnu.edu.cn
* Corresponding authors.

Table of Contents

1. General Information 2

2. Experimental Details and Characterization 2

3. NMR Spectra 15

4. Mass Spectra 35

5.TGA spectra 44

5. X-Ray Crystallographic Analysis 46

6. DFT calculations 57

7. Photophysical Measurements 81

8. References 93

1. General Information

^1H NMR (600 MHz) and ^{13}C NMR (151 MHz) spectra were recorded on a Bruker AVANCE NEO 600 spectrometer. Chemical shifts were reported as the delta scale in ppm relative to $CDCl_3$ (δ = 7.26 ppm for ^1H NMR, and δ = 77.16 ppm for ^{13}C NMR), CD_2Cl_2 (δ = 5.32 ppm for ^1H NMR, and δ = 53.84 ppm for ^{13}C NMR), and DMSO-d_6 (δ = 2.50 ppm for ^1H NMR, and δ = 39.52 ppm for ^{13}C NMR). Mass spectrometry (MALDI MS) were obtained from the Waters Synapt G2 mass spectrometer. Semipreparative GPC was carried out on a Shimadzu recycling GPC system equipped with a LC-20 AD pump, SPD20A UV detector and a set of JAIGEL 2.5H (20 × 600 mm) columns in Chloroform as the eluent at a flow rate of 5.0 mL/min. Thermogravimetric analysis (TGA) was performed on a TGA209F1 instrument from NETZSCH. Absolute PL quantum yields and emission decay curves of the compounds were collected by using a spectrometer (FLS980) from Edinburgh Instruments equipped with an integrating sphere. Steady-state PL spectra were obtained by the spectrophotometers of QE65 Pro and FLS980. Measurement of UV-visible absorption spectra was conducted on a spectrometer from HITACHI Instruments (UV-3900H) and Shimadzu UV-2700 spectrometers. Unless otherwise noted, commercially available solvents and reagents were used without further purification. TLC analysis was performed on silica gel plates, and column chromatography was performed on silica gel (200-300 mesh) or neutral alumina (200-300 mesh).

2. Experimental Details and Characterization

2.1 Synthesis of 2dbts-[1.1][2]PCP

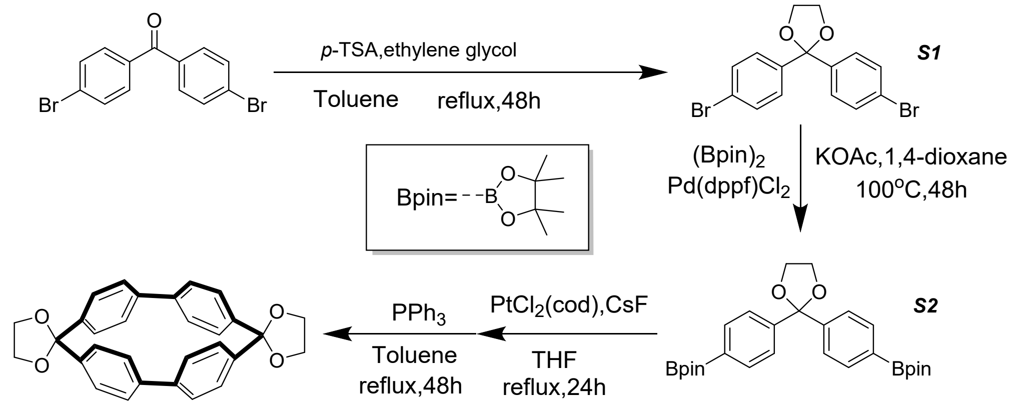

2dbts-[1.1][2]PCP

Synthesis of Compound S1

Compound **S1** was synthesized according to the previously reported method. [1]
A mixture of 4,4'-dibromobenzophenone (1000 mg, 2.94 mmol, 1.0 equiv.) and *p*-toluenesulfonic acid monohydrate (83.9 mg, 0.45 mmol, 0.15 equiv.) was placed in a two-necked round-bottom flask equipped with a Dean–Stark trap. The system was sealed, degassed, and flushed with nitrogen. Ethylene glycol (1824.65 mg, 29.4 mmol, 10 equiv.) and toluene (30 mL) were then added via syringe under a nitrogen atmosphere. The reaction mixture was heated to reflux and stirred for 48 h. After completion, the mixture was cooled to room temperature, and the pH was adjusted to 7 by the addition of saturated aqueous sodium bicarbonate. The solvent was removed under reduced pressure, and the residue was extracted with CH_2Cl_2 (10 mL). The organic phase was washed with saturated brine, dried over anhydrous sodium sulfate, and concentrated under reduced pressure. The crude product was purified by column chromatography on alumina, eluting with PE. After removal of the solvent under reduced pressure and standing for 30 min, compound **S1** was obtained as a white solid (1000.9 mg, 89%).
S1: (Known compound) [1] **^{1}H NMR** (600 MHz, CD_2Cl_2) δ 7.46 (d, *J* = 8.5 Hz, 4H), 7.36 (d, *J* = 8.5 Hz, 4H), 4.02 (s, 4H).

Synthesis of Compound S2

In a dry Schlenk flask with a magnetic stir bar, **S1** (500 mg, 1.3 mmol, 1.0 equiv.), bis(pinacolato)diboron (804.02 mg, 3.12 mmol, 2.4 equiv.), Pd(dppf)Cl_2 (62.71 mg, 0.13 mmol, 0.1 equiv.), and potassium acetate (766.47 mg, 7.81 mmol, 6.0 equiv.) were added sequentially. The tube was sealed and subjected to three vacuum–nitrogen cycles to remove air and ensure an inert atmosphere. Dry 1,4-dioxane (15 mL) was then added under nitrogen. The reaction mixture was stirred at 78 °C for 48 h. Upon completion, the mixture was cooled to room temperature and the solvent was removed under reduced pressure. The residue was diluted with CH_2Cl_2 (10 mL) and washed with saturated brine. The organic layer was separated, dried over anhydrous sodium sulfate, filtered, and concentrated under reduced pressure. The crude product was rapidly purified by column chromatography on alumina, using CH_2Cl_2/PE (v/v = 1:4) as the eluent. The desired fractions were collected and concentrated under reduced pressure to afford a white solid. The product was further purified by recrystallization from CH_2Cl_2/hexane to yield **S2** (519 mg, 83%) as a white solid.
S2: **^{1}H NMR** (600 MHz, CD_2Cl_2) δ 7.70 (d, *J* = 8.2 Hz, 4H), 7.48 (d, *J* = 8.2 Hz, 4H), 4.03 (s, 4H), 1.31 (s, 24H).

^{13}C NMR (151 MHz, CD_2Cl_2) δ 25.03, 65.38, 84.21, 109.54, 125.74, 134.87, 145.49.
MALDI-TOF mass: m/z 479.349 (calculated for $C_{27}H_{36}B_2O_6^+$ [M+H]$^+$: 479.277).

Synthesis of 2dbts-[1.1][2]PCP

In a dry Schlenk flask, **1** (300 mg, 0.628 mmol, 1.0 equiv.), $PtCl_2$(cod) (234.73 mg, 0.628 mmol, 1.0 equiv.), and CsF (571.76 mg, 3.76 mmol, 6.0 equiv.) were added, and subjected to three cycles of nitrogen swap. Dry THF (20 mL) was added via syringe through septum and the reaction mixture heated to 66 °C. After stirring for 24 h, the reaction mixture was cooled to room temperature, and the volatiles were removed under vacuum to afford a crude product containing cyclic intermediates. This crude mixture was then combined with PPh_3 (1547.51 mg, 5.9 mmol, 10.0 equiv.) in toluene (20 mL) and refluxed under a nitrogen atmosphere for 48 h. Afterward, the mixture was cooled to room temperature, and the volatiles were removed under vacuum. The residue was diluted with CH_2Cl_2 (50 mL) and washed with saturated brine. The organic phase was dried over Na_2SO_4 and concentrated under reduced pressure. The crude product was purified by short column chromatography (CH_2Cl_2/PE=1:5， v/v), before further purification by recycling GPC (Chloroform). Further purification by recrystallization from CH_2Cl_2/*n*-hexane gave pure **2dbts-[1.1][2]PCP (**25 mg, 18 %**)**.
2dbts-[1.1][2]PCP: **^{1}H NMR** (600 MHz, CD_2Cl_2) δ 7.26 (d, *J* = 8.7 Hz, 8H), 7.18 (d, *J* = 8.7 Hz, 8H), 4.27 (s, 8H).
^{13}C NMR (151 MHz, CD_2Cl_2) δ 65.29, 104.35, 125.91, 126.51.
UV/Vis (THF): λ_{max} (log ε) = 274 nm (4.62).
MALDI-TOF mass: m/z 448.144 (calculated for $C_{26}H_{18}H_2^+$ [M]+: 448.167).

2.2 Synthesis of 2ketals-[1.1][2]PCP

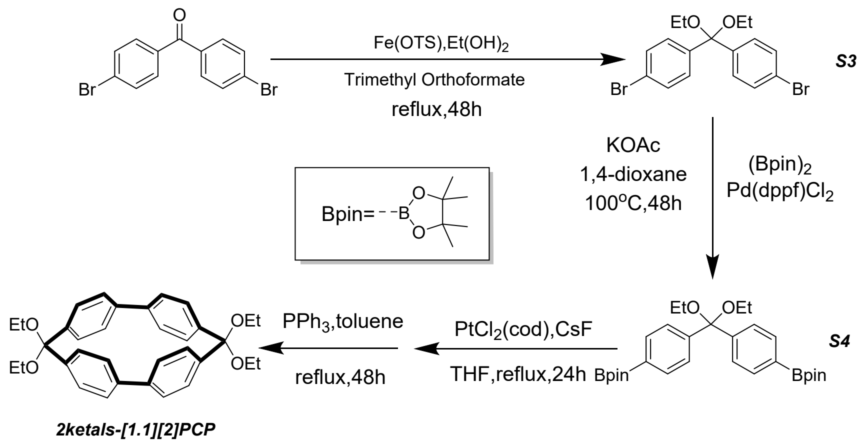

Synthesis of S3

Compound **S3** was synthesized according to the previously reported method. [2]
In a Schlenk flask, 4,4'-Dibromobenzophenone (340.01 mg, 1 mmol, 1.0 equiv), Iron(III) p-toluenesulfonate (17 mg, 0.03 mmol, 3 mol % equiv.), and subjected to three cycles of nitrogen swap. Triethyl orthoformate (0.32 mL, 3 mmol, 3.0 equiv.) and dry Ethanol (10 mL) was added via syringe through septum and the reaction mixture heated to 78 °C. After stirring for 24 h, the mixture was cooled to room temperature, and the volatiles were removed under vacuum. The residue was diluted with CH_2Cl_2 (10 mL) and washed with saturated brine. The organic phase was dried over

Na_2SO_4 and concentrated under reduced pressure. The crude product was Purified though column chromatography on alumina (100% PE) and follow-up reprecipitation (CH_2Cl_2/hexane). The product **S3** (340 mg, 82%) was obtained as a white solid.

S3: Known compound[2] **^{1}H NMR** (600 MHz, DMSO-d_6) δ 7.53 (d, J = 8.4 Hz, 4H), 7.36 (d, J = 8.4 Hz, 4H), 3.23 (q, J = 7.0 Hz, 4H), 1.17 (t, J = 7.0 Hz, 6H).

Synthesis of S4

In a dry Schlenk flask, **S3** (912 mg, 2.2 mmol, 1.0 equiv.), Bis(pinacolato)diboron (1363.23 mg, 5.29 mmol, 2.4 equiv.), and $PdCl_2$(dppf) (96.61 mg, 0.13 mmol, 6 mol % equiv.) and K_3PO_4 (1296.43 mg, 13.21 mmol, 6.0 equiv.) were added, and subjected to three cycles of nitrogen swap. Dry dioxane (15 mL) was added via syringe through septum and the reaction mixture heated to 80 °C. After stirring for 48 h, the mixture was cooled to room temperature, and the volatiles were removed under vacuum. The residue was diluted with CH_2Cl_2 (10 mL) and washed with saturated brine. The organic phase was dried over Na_2SO_4 and concentrated under reduced pressure. The crude product was Purified though column chromatography on alumina (PE/ CH_2Cl_2 = 100:1) and follow-up reprecipitation (CH_2Cl_2/hexane). The product **S4** (225 mg, 54%) was obtained as a white solid.

S4: ^{1}H NMR (600 MHz, CD_2Cl_2) δ 7.66 (d, J = 8.4 Hz, 4H), 7.50 (d, J = 8.4 Hz, 4H), 3.30 (q, J = 6.9 Hz, 4H), 1.30 (s, 24H), 1.20 (t, J = 6.9 Hz, 6H).

^{13}C NMR (151 MHz, CD_2Cl_2) δ 15.27, 25.03, 57.64, 84.13, 102.51, 126.45, 134.73, 146.63.

MALDI-TOF mass: m/z 463.283 (calculated for $C_{27}H_{37}B_2O_5^+$ [M-OEt$^-$]$^+$: 463.282).

Synthesis of compound 2ketals-[1.1][2]PCP

In a dry Schlenk flask, **S4** (300 mg, 0.59 mmol, 1.0 equiv.), $PtCl_2$(cod) (220.84mg, 0.59mmol, 1.0 equiv.), and CsF (537.72 mg, 3.54 mmol, 6.0 equiv.) were added, and subjected to three cycles of nitrogen swap. Dry THF (20 mL) was added via syringe through septum and the reaction mixture heated to 66 °C. After stirring for 24 h, the reaction mixture was cooled to room temperature, and the solvent were removed under vacuum to afford a crude product containing cyclic intermediates. This crude mixture was then combined with PPh_3 (1440.0 mg, 4.17 mmol) in toluene (20 mL) and refluxed under a nitrogen atmosphere for 48 h. Afterward, the mixture was cooled to room temperature, and the solvent were removed under vacuum. The residue was diluted with CH_2Cl_2 (50 mL) and washed with saturated brine. The organic phase was dried over Na_2SO_4 and concentrated under reduced pressure. The crude product was purified by short column chromatography (CH_2Cl_2/PE=6:1, v/v), before further purification by recycling GPC (Chloroform). Further purification by recrystallization from CH_2Cl_2/n-hexane gave pure **2katals-[1.1][2]PCP (**65 mg, 43 %, white solid**).**

2katals-[1.1][2]PCP: ^{1}H NMR (600 MHz, CD_2Cl_2) δ 7.24 (d, J = 8.4 Hz, 8H), 7.12 (d, J = 8.4 Hz, 8H), 3.80 (q, J = 7.0 Hz, 8H), 1.43 (t, J = 7.0 Hz, 12H).

^{13}C NMR (151 MHz, CD_2Cl_2) δ 25.03, 65.38, 84.21, 109.54, 125.74, 134.87, 145.49.

UV/Vis (THF): λ_{max} (log ε) = 272 nm (4.61).

MALDI-TOF MASS: m/z 508.281 (calculated for $C_{34}H_{35}O_4^+$ [M]$^+$: 508.261).

2.3 Synthesis of Compound ketal-2Me-[1,1][2]PCP

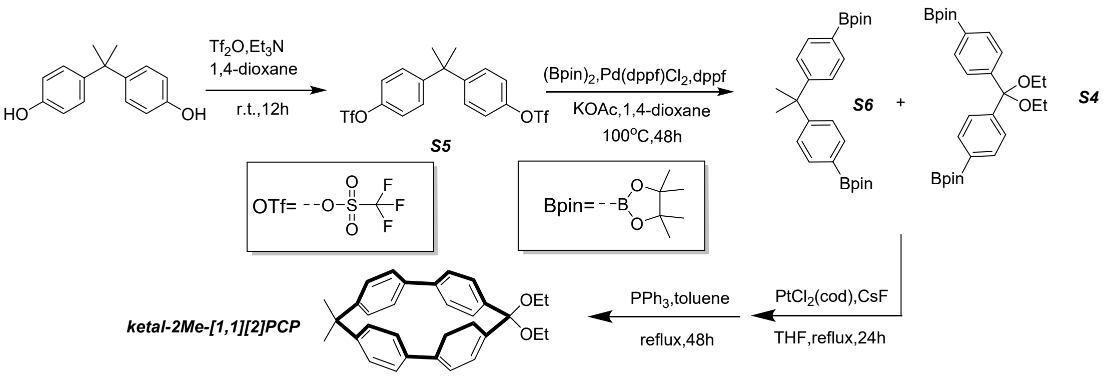

Synthesis of Compound S5

A mixture of 4,4'-dihydroxybenzyl alcohol (2000 mg, 8.76 mmol, 2.5 equiv.), triethylamine (7.3 mL, 53 mmol, 6.0 equiv.), and 1,4-dioxane (30 mL) was added to a dry round-bottom flask. The mixture was pre-cooled in an ice-water bath for 5 minutes. Under the ice-water bath conditions, trifluoromethanesulfonic anhydride (9 mL, 53 mmol, 6.0 equiv.) was added dropwise under 5 minutes. After the addition, the ice-water bath was removed, and the reaction mixture was stirred at room temperature overnight. The next day, deionized water (20 mL) was slowly added to quench the reaction, followed by dilution with CH_2Cl_2 (50 mL) and extraction. The organic phase was separated, dried over anhydrous sodium sulfate, filtered, and concentrated under reduced pressure to yield the crude product. The crude product was purified by silica gel column chromatography using PE as the eluent. The desired fractions were collected and concentrated under reduced pressure using a rotary evaporator to remove the solvent. The resulting colorless oil was dried under vacuum to obtain compound **S5** (2689 mg, 62%) as a white solid.

S5 (Known compound)[3]**: ^{1}H NMR** (600 MHz, $CDCl_3$) δ 7.27 (d, J = 8.9 Hz, 4H), 7.19 (d, J = 8.9 Hz, 4H), 1.69 (s, 6H).

Synthesis of Compound S6

In a dry Schlenk tube with a magnetic stir bar, **S5** (2650 mg, 5.38 mmol, 1.0 equiv.), bis(pinacolato)diboron (3007 mg, 11.84 mmol, 2.2 equiv.), Pd(dppf)Cl_2 (236 mg, 0.323 mmol, 6 mol % equiv.), 1,1-bis(diphenylphosphino)ferrocene (179 mg, 0.323 mmol, 6 mol % equiv.), and potassium acetate (3167 mg, 32.28 mmol, 6.0 equiv.) were added sequentially. The tube was sealed and subjected to three vacuum–nitrogen cycles to remove air and ensure an inert atmosphere. Anhydrous 1,4-dioxane (45 mL) was then added under nitrogen. The reaction mixture was stirred at 80°C for 48 h. Upon completion, the mixture was cooled to room temperature and the solvent was removed under reduced pressure. The residue was diluted with CH_2Cl_2 (20 mL) and washed with saturated brine. The organic layer was separated, dried over anhydrous sodium sulfate, filtered, and concentrated under reduced pressure. The crude product was rapidly purified by column chromatography on alumina, using CH_2Cl_2/PE (v/v = 1:5) as the eluent. The desired fractions were collected and concentrated under reduced pressure to afford a white solid. The product was further purified by recrystallization from CH_2Cl_2/hexane to yield compound **S6** (1574 mg, 65%) as a white solid.

S6: ^{1}H NMR (600 MHz, $CDCl_3$) δ 7.71 (d, J = 8.2 Hz, 4H), 7.23 (d, J = 8.2 Hz, 4H), 1.67 (s, 6H), 1.33 (s, 24H).

^{13}C NMR (151 MHz, $CDCl_3$) δ 153.77, 134.62, 126.29, 83.65, 43.48, 30.51, 24.85.

MALDI-TOF mass: m/z 471.373 (calculated for $C_{26}H_{18}H_2^+$ [M]$^+$: 471.285).

Synthesis of Compound ketal-2Me-[1,1][2]PCP

In a Schlenk flask, **S6** (113.40 mg, 0.22 mmol, 1.0 equiv.), compound **S4** (100 mg, 0.22 mmol, 1.0 equiv.), $PtCl_2$(cod) (166.87 mg, 0.44 mmol, 2.0 equiv.), and CsF (406.48 mg, 1.34 mmol, 6.0 equiv.) were added, and subjected to three cycles of nitrogen swap. Dry THF (15 mL) was added via syringe through septum and the reaction mixture heated to 66 °C. After stirring for 24 h, the reaction mixture was cooled to room temperature, and the solvent were removed under vacuum to afford a crude product containing cyclic intermediates. This crude mixture was then combined with PPh_3 (1169.81 mg, 4.46 mmol, 10.0 equiv.) in toluene (15 mL) and refluxed under a nitrogen atmosphere for 48 h. Afterward, the mixture was cooled to room temperature, and the solvent were removed under vacuum. The residue was diluted with CH_2Cl_2 (20 mL) and washed with saturated brine. The organic phase was dried over Na_2SO_4 and concentrated under reduced pressure. The crude product was purified by short column chromatography (CH_2Cl_2/PE=1:6, v/v), before further purification by recycling GPC (eluting with chloroform). Further purification by recrystallization from CH_2Cl_2/*n*-hexane gave pure **ketal-2Me-[1,1][2]PCP (**26.00 mg, 26%**)**.

ketal-2Me-[1,1][2]PCP: ^{1}H NMR (600 MHz, $CDCl_3$) δ 7.22 (d, *J* = 8.7 Hz, 4H), 7.16 (d, *J* = 8.7 Hz, 4H), 7.07 (d, *J* = 8.7 Hz, 4H), 7.03 (d, *J* = 8.7 Hz, 4H), 3.83 (qd, *J* = 7.0, 1.8 Hz, 4H), 1.94 (s, 6H), 1.43 (dd, *J* = 7.0, 1.8 Hz, 6H).

^{13}C NMR (151 MHz, $CDCl_3$) δ 15.56, 22.02, 45.30, 59.13, 106.24, 125.80, 125.88, 125.90, 127.33, 138.13, 139.47, 147.85, 154.32.

UV/Vis (THF): λ_{max} (log ε) = 272 nm (4.74).

MALDI-TOF mass: m/z 448.245 (calculated for $C_{32}H_{31}O_2^+$ [M]$^+$: 448.240).

2.4 Synthesis of Compound 2ketals-[1,1][3]PCP

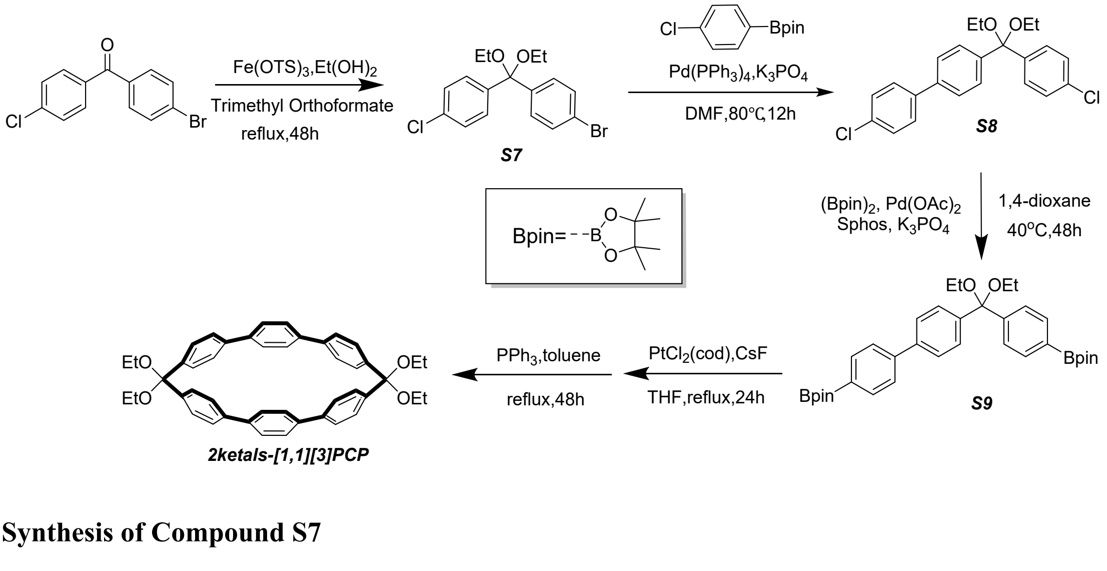

Synthesis of Compound S7

In a Schlenk flask, 4-Bromo-4-chlorobenzophenone (500 mg, 1.69 mmol, 1.0 equiv.) and ferric p-toluenesulfonate (28.9 mg, 0.08 mmol, 5 mol%) were added, and subjected to three cycles of nitrogen swap. Triethyl orthoformate (0.55 mL, 5.08 mmol, 3.0 equiv.) and dry Ethanol (10 mL)

was added via syringe through septum and the reaction mixture heated to 78 °C. After stirring for 48 h, the mixture was cooled to room temperature, saturated aqueous sodium bicarbonate was added to adjust the pH=7. and the volatiles were removed under vacuum. The residue was diluted with CH_2Cl_2 (10 mL) and washed with saturated brine. The organic phase was dried over Na_2SO_4 and concentrated under reduced pressure. The crude product was purified by short column chromatography on alumina (CH_2Cl_2/PE=1:6, v/v). After removal of the solvent under reduced pressure and standing for 30 min, the product **S7** (518 mg, 83%) was obtained as a white solid.

S7: ^{1}**H NMR** (600 MHz, CD_2Cl_2) δ 7.43 (dd, J = 8.7, 8.7 Hz, 4H), 7.38 (d, J = 8.7 Hz, 2H), 7.27 (d, J = 8.7 Hz, 2H), 3.28 (q, J = 7.1 Hz, 4H), 1.20 (t, J = 7.1 Hz, 6H).

^{13}**C NMR** (151 MHz, CD_2Cl_2) δ 15.19, 101.79, 121.88, 128.50, 128.71, 129.07, 131.48, 133.63, 142.07, 142.66.

MALDI-TOF mass: m/z 400.043 (calculated for $C_{26}H_{18}H_2^+$ $[M]^+$: 400.078).

Synthesis of Compound S8

Compound **S7** (400 mg, 1.08 mmol, 1.0 equiv.), $Pd(PPh_3)_4$ (125 mg, 0.108 mmol, 10 mol%), K_3PO_4 (1377.9 mg, 6.49 mmol, 6.0 equiv.), and 4-chlorophenylboronic acid pinacol ester (258.1 mg, 1.08 mmol, 1.0 equiv.) were added sequentially to a dried Schlenk flask with a magnetic stir bar. The tube was sealed and subjected to three freeze-pump-thaw cycles to ensure an inert nitrogen atmosphere. After degassing, DMF (10 mL) was added via syringe. The mixture was stirred and heated at 78 °C for 48 h under nitrogen. After completion of the reaction, the mixture was cooled to room temperature and then poured slowly into pre-cooled saturated brine solution, resulting in the formation of a pale-yellow solid. The precipitate was collected by filtration, dried, and then dissolved in dichloromethane. The crude product was purified by flash column chromatography on alumina, using petroleum ether as the eluent. After removal of the solvent under reduced pressure, compound **S8** (316 mg, 73% yield) was obtained as a colorless oil.

S8: ^{1}**H NMR** (600 MHz, CD_2Cl_2) δ 7.57 (d, J = 8.4 Hz, 2H), 7.54 – 7.48 (m, 6H), 7.40 (d, J = 8.4 Hz, 2H), 7.29 (d, J = 8.7 Hz, 2H), 3.33 (qd, J = 7.1, 4.4 Hz, 4H), 1.23 (t, J = 7.1 Hz, 6H).

^{13}**C NMR** (151 MHz, CD_2Cl_2) δ 15.26, 102.05, 126.95, 127.65, 128.47, 128.72, 129.24, 133.49, 133.66, 139.32, 139.60, 142.49, 142.90.

MALDI-TOF mass: m/z 355.062 (calculated for $C_{21}H_{17}Cl_2O^+$ $[M\text{-}OEt^-]^+$: 355.065).

Synthesis of Compound S9

Compound **S8** (500 mg, 1.25 mmol, 1.0 equiv.), bis(pinacolato)diboron (1898.4 mg, 7.48 mmol, 6.0 equiv.), $Pd(OAc)_2$ (16.83 mg, 0.07 mmol, 5 mol%), KOAc (734.09 mg, 7.48 mmol, 6.0 equiv.), and SPhos (61.58 mg, 0.15 mmol, 10 mol%) were added to a dried Schlenk flask. The tube was sealed and subjected to three cycles of nitrogen swap to ensure an inert nitrogen atmosphere. Dry 1,4-dioxane (15 mL) was then added via syringe. The mixture was stirred at 40 °C for 48 h. After reaction completed, the mixture was allowed to cool to room temperature, and the solvent was removed under reduced pressure. The residue was extracted with CH_2Cl_2 (20 mL), washed with saturated brine, and the organic layer was dried over anhydrous sodium sulfate. After filtration and concentration under reduced pressure, the crude product was purified by flash chromatography on an alumina column, using PE/ CH_2Cl_2 (3:1, v/v) as the eluent. The collected product was further

recrystallized from CH_2Cl_2/n-hexane to afford compound **S9** (489 mg, 67% yield) as a white solid.
S9: ^{1}**H NMR** (600 MHz, $CDCl_3$) δ 7.88 – 7.82 (m, 2H), 7.75 (d, J = 7.8 Hz, 2H), 7.59 – 7.54 (m, 6H), 7.51 (d, J = 8.1 Hz, 2H), 3.35 (qd, J = 7.1, 3.1 Hz, 4H), 1.35 (s, 12H), 1.31 (s, 12H), 1.23 (t, J = 7.1 Hz, 6H).
^{13}**C NMR** (151 MHz, $CDCl_3$) δ 15.10, 24.78, 24.81, 57.18, 83.64, 83.72, 102.09, 126.06, 126.31, 126.70, 127.14, 134.44, 135.12, 139.76, 142.50, 143.52, 146.27.
MALDI-TOF mass: m/z 400.043 (calculated for $C_{26}H_{18}H_2^+$ $[M]^+$: 400.078).

Synthesis of Compound 2ketals-[1,1][3]PCP

In a Schlenk flask, **S9** (300 mg, 0.513 mmol, 1.0 equiv.), $PtCl_2$(cod) (192.07 mg, 0.513 mmol, 1.0 equiv.), and CsF (467.54 mg, 3.08 mmol, 6.0 equiv.) were added, and subjected to three cycles of nitrogen swap. Dry THF (20 mL) was added via syringe through septum and the reaction mixture heated to 66 °C. After stirring for 24 h, the reaction mixture was cooled to room temperature, and the solvent were removed under vacuum to afford a crude product containing cyclic intermediates. This crude mixture was then combined with PPh_3 (1348.67 mg, 5.13 mmol, 10.0 equiv.) in toluene (20 mL) and refluxed under a nitrogen atmosphere for 48 h. Afterward, the mixture was cooled to room temperature, and the solvent were removed under vacuum. The residue was diluted with CH_2Cl_2 (50 mL) and washed with saturated brine. The organic phase was dried over Na_2SO_4 and concentrated under reduced pressure. The crude product was purified by short column chromatography (CH_2Cl_2/PE=1:6， v/v), before further purification by recycling GPC (Chloroform). Further purification by recrystallization from CH_2Cl_2/*n*-hexane gave pure **2ketals-[1,1][3]PCP (**25 mg, 18 %**)**.
2ketals-[1,1][3]PCP: ^{1}**H NMR** (600 MHz, CD_2Cl_2) δ 7.34 – 7.29 (m, 16H), 7.25 (d, J = 8.6 Hz, 8H), 3.81 (q, J = 7.0 Hz, 8H), 1.43 (t, J = 7.0 Hz, 12H).
^{13}**C NMR** (151 MHz, CD_2Cl_2) δ 15.47, 58.79, 105.40, 126.80, 127.00, 127.65, 127.75, 138.76, 138.87, 144.69.
UV/Vis (THF): λ_{max} (log ε) = 293 nm (4.98).
MALDI-TOF mass: m/z 660.329 (calculated for $C_{46}H_{44}O_4^+$ $[M]^+$: 660.324).

2.5 Synthesis of Compound 2ketals-[1,1][4]PCP

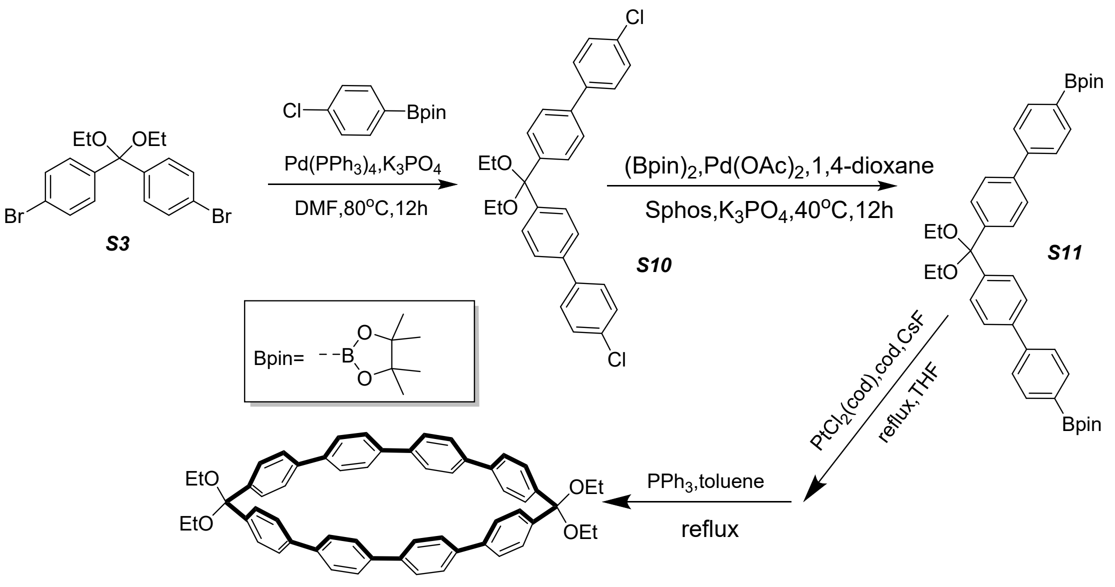

2ketals-[1,1][4]PCP

Synthesis of Compound S10

Compound **S3** (800 mg, 1.94 mmol, 1.0 equiv.), $Pd(PPh_3)_4$ (224.2 mg, 0.19 mmol, 10 mol%), K_3PO_4 (2470.7 mg, 11.64 mmol, 6.0 equiv.), and 4-chlorophenylboronic acid pinacol ester (925.72 mg, 3.90 mmol, 2.0 equiv.) were added to a dried Schlenk tube equipped with a magnetic stir bar. The reaction tube was sealed and degassed by three cycles of evacuation and nitrogen backfilling to ensure an inert atmosphere. Dry DMF (10 mL) was then added via syringe. The reaction mixture was heated to 78 °C and stirred at this temperature for 48 h. Upon completion, the reaction was allowed to cool to room temperature, and the mixture was slowly poured into pre-cooled saturated brine, resulting in the immediate formation of a pale-yellow precipitate. The solid was collected by filtration, dried, and dissolved in CH_2Cl_2 (20 ml). The resulting solution was purified by flash chromatography on an alumina column using petroleum ether as the eluent. The collected fractions containing the target compound were concentrated under reduced pressure to afford compound **S6** as a white solid (912.5 mg, 83% yield).

S10：**^{1}H NMR** (600 MHz, DMSO-d_6) δ 7.65 (dd, J = 8.6, 8.6 Hz, 8H), 7.56 (d, J = 8.6 Hz, 4H), 7.50 (d, J = 8.6 Hz, 4H), 3.31 (s, 4H), 1.22 (t, J = 7.0 Hz, 6H).

^{13}C NMR (151 MHz, DMSO) δ 14.93, 56.79, 101.52, 126.43, 126.85, 128.34, 128.79, 132.30, 137.85, 138.40, 142.21.

MALDI-TOF mass: m/z 483.944 (calculated for $C_{26}H_{18}H_2^+$ $[M+Na]^+$: 484.098).

Synthesis of Compound S11

Compound **S10** (400 mg, 0.706 mmol, 1.0 equiv.), bis(pinacolato)diboron (1076.14 mg, 7.48 mmol, 10.6 equiv.), $Pd(OAc)_2$ (9.51 mg, 0.07 mmol, 10 mol%), KOAc (416.11 mg, 7.48 mmol, 10.6 equiv.), and SPhos (34.78 mg, 0.15 mmol, 20 mol%) were added to a dried Schlenk tube. The sealed tube was subjected to three cycles of evacuation and nitrogen backfilling to ensure an inert atmosphere. Then 1,4-dioxane (15 mL) was added via syringe, and the reaction mixture was heated to 40 °C with stirring for 48 h. After reaction completion, the reaction mixture was cooled to room temperature, and the solvent was removed under reduced pressure. The residue was dissolved in CH_2Cl_2 (20 mL) and washed with saturated brine. The organic layer was dried over anhydrous sodium sulfate and concentrated under reduced pressure. The crude product was purified by column

chromatography on alumina using PE/ CH_2Cl_2 (3:1, v/v) as the eluent. The fractions containing the target compound were combined and concentrated. The residue was recrystallized from CH_2Cl_2 /n-hexane to afford compound **5** (406 mg, 87% yield) as a white solid.

S11: ^{1}**H NMR** (600 MHz, $CDCl_3$) δ 7.85 (d, J = 8.1 Hz, 4H), 7.62 (d, J = 8.4 Hz, 4H), 7.58 (d, J = 8.1 Hz, 4H), 7.55 (d, J = 8.4 Hz, 4H), 3.39 (q, J = 7.0 Hz, 4H), 1.35 (s, 24H), 1.26 (t, J = 7.0 Hz, 6H).

^{13}**C NMR** (151 MHz, $CDCl_3$) δ 0.00, 15.19, 24.88, 57.23, 83.80, 102.10, 126.39, 126.82, 127.22, 135.20, 139.87, 142.70, 143.56.

MALDI-TOF mass: m/z 615.348 (calculated for $C_{39}H_{45}B_2O_5^+$ [M-OEt $^-$]$^+$: 615.345).

Synthesis of Compound 2ketals-[1,1][4]PCP

Compound **5** (200 mg, 0.30 mmol, 1.0 equiv.), $PtCl_2$(cod) (113.30 mg, 0.30 mmol, 1.0 equiv.), and CsF (275.98 mg, 1.80 mmol, 6.0 equiv.) were added to a dried Schlenk tube. The tube was evacuated and refilled with nitrogen three times to ensure an inert atmosphere. Dry THF (15 mL) was then added, and the reaction mixture was stirred at 66 °C. After stirring for 24 h, the reaction mixture was cooled to room temperature, and the solvent were removed under vacuum to afford a crude product containing cyclic intermediates. This crude mixture was then combined with PPh_3 (794.25 mg, 3.0 mmol, 10 equiv.) in toluene (20 mL) and refluxed under a nitrogen atmosphere for 48 h. Afterward, the mixture was cooled to room temperature, and the volatiles were removed under vacuum. The residue was diluted with CH_2Cl_2 (50 mL) and washed with saturated brine. The organic phase was dried over Na_2SO_4 and concentrated under reduced pressure. The crude product was purified by column chromatography on alumina (PE/ CH_2Cl_2=6:1, v/v). The resulting pale-yellow solid was dissolved in chloroform and further purified by gel permeation chromatography (GPC). The collected white solid was recrystallized from CH_2Cl_2/n-hexane to afford compound **2ketals-[1,1][4]PCP** as a white solid (19.4 mg, 16% yield).

2ketals-[1,1][4]PCP: ^{1}**H NMR** (600 MHz, $CDCl_3$) δ 7.50 (d, J = 8.4 Hz, 8H), 7.43 (d, J = 8.4 Hz, 8H), 7.41-7.36 (m, 16H), 3.79 (q, J = 7.0 Hz, 8H), 1.44 (t, J = 7.0 Hz, 12H).

^{13}**C NMR** (151 MHz, $CDCl_3$) δ 15.37, 104.35, 126.35, 126.43, 126.97, 127.28, 137.99, 138.37, 138.78, 143.23.

UV/Vis (THF): λ_{max} (log ε) = 312 nm (5.08).

MALDI-TOF mass: m/z 812.380 (calculated for $C_{58}H_{52}O_4^+$ [M]$^+$: 812.387).

2.6 Hydrolysis Reactions of macrocyclic ketals

2.6.1.1 Optimization of the Hydrolysis Reaction Conditions

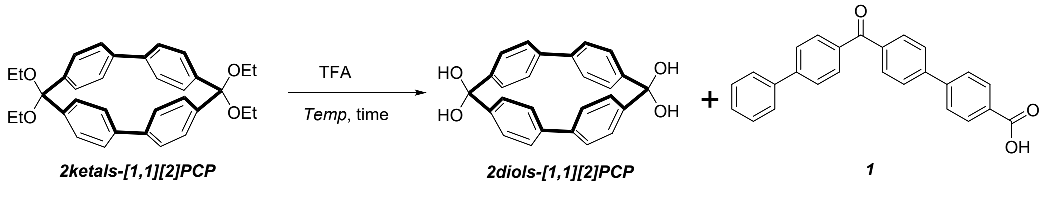

entry	Temp	Time	Yield[b] of **2diols-[1.1][2]PCP**	Yield[b] of **1**
1	-25 °C	30 min	96% (78 %)[c]	4%
2	0 °C	30 min	87 %	13 %
3	25 °C	30 min	43 %	57 %
4	70 °C	30 min	0 %	100 %

[a] All the reactions were carried out using **2ketals-[1.1][2]PCP** (20 mg, 0.04 mmol) and Trifluoroacetate (3ml). [b] Determined by ^{1}H NMR spectroscopy unless otherwise noted. [c] Yield of isolated product.

2ketals-[1,1][2]PCP (20 mg) was placed in a Schlenk tube equipped with a magnetic stir bar. The tube was sealed, evacuated, and backfilled with nitrogen, and then immersed in an ethanol/oil bath maintained at –25, 0, 25, or 70 °C. Trifluoroacetic acid (TFA, 3 mL) was added dropwise, and the mixture was stirred at the same temperature for 30 min. Upon completion, saturated aqueous sodium bicarbonate was rapidly added, and the pH was adjusted to 7. The mixture was filtered to collect the solid, and the solvent was removed. The crude product was directly analyzed by NMR to determine the yield. For entry 4 (70 °C, 30 min), the crude product was recrystallized from ethyl acetate/n-hexane to afford compound **1**.

1：Gray solid, 14.1 mg, 95 % isolated yield.
^{1}H NMR (600 MHz, DMSO-d_6) δ 13.08 (s, 1H), 8.08 (d, J = 8.4 Hz, 2H), 7.97 (d, J = 8.4 Hz, 2H), 7.94 – 7.88 (m, 8H), 7.82 – 7.78 (m, 2H), 7.54 (dd, J = 8.4, 7.2 Hz, 2H), 7.46 (t, J = 7.2 Hz, 1H).
^{13}C NMR (151 MHz, DMSO d_6) δ 126.76, 126.95, 127.08, 127.14, 128.35, 129.06, 129.98, 130.33, 130.36, 135.68, 136.54, 138.82, 142.79, 142.93, 144.16, 166.94, 194.80.
UV/Vis (THF): λ_{max} (log ε) = 298 nm (4.89).
MALDI-TOF mass: m/z 664.247 (calculated for $C_{50}H_{32}O_2^+$ [M]$^+$: 664.240).

2.6.1.2 Hydrolysis Reaction of 2dbts-[1,1][2]PCP

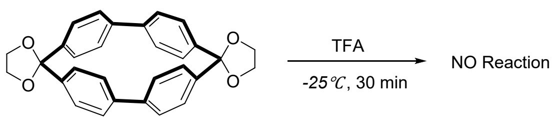

2dbts-[1,1][2]PCP

2.6.2 General Experimental Procedures for Hydrolysis Reactions.

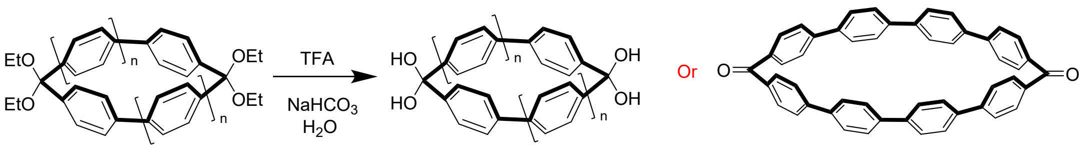

2ketals-[1,1][n]PCPs
n=2, 3, 4

2diols-[1,1][n]PCPs
n=2, 3

2ketones-[1,1][n]PCPs
n=4

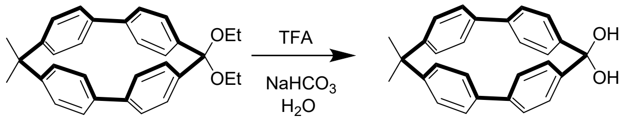

ketal-2Me-[1,1][2]PCP

diol-2Me-[1,1][2]PCP

Macrocyclic ketals (20 mg) was placed in a Schlenk tube equipped with a magnetic stir bar. The tube was sealed, evacuated, and backfilled with nitrogen. The reaction tube was then immersed in an ethanol bath pre-cooled to –25 °C. Cold trifluoroacetic acid (TFA, 3 mL, –25 °C) was added dropwise, and the resulting mixture was stirred at –25 °C for 30 min. After completion of the reaction, saturated aqueous sodium bicarbonate was added rapidly, and the pH was adjusted to 7. Then the reaction mixture was filtered, and the solid was collected and recrystallized from a mixture of ethyl acetate /*n*-hexane to afford the corresponding macrocyclic diols or macrocyclic diketones.

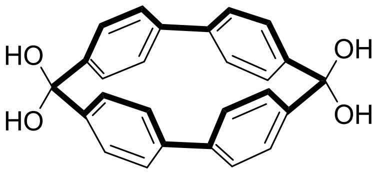

2diols-[1,1][2]PCP

2diols-[1,1][2]PCP: White solid, 12.2 mg, 78% isolated yield.
^{1}H NMR (600 MHz, DMSO-d_6) δ 7.31 (d, J = 8.8 Hz, 8H), 7.24 (d, J = 8.8 Hz, 8H), 6.86 (s, 4H).
^{13}C NMR (151 MHz, DMSO-d_6) δ 97.43, 123.98, 126.03, 136.66, 153.58.
UV/Vis (THF): λ_{max} (log ε) = 274 (4.06).
MALDI-TOF mass: m/z 479.349 (calculated for $C_{27}H_{36}B_2O_6^+$ [M+H]$^+$: 479.277).

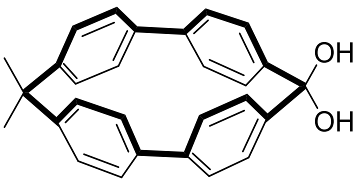

diol-2Me-[1,1][4]PCP

diol-2Me-[1,1][2]PCP： White solid, 10.1 mg, 58 % isolated yield.
^{1}H NMR (600 MHz, DMSO-d_6) δ 7.28 (d, J = 8.7 Hz, 4H), 7.25 (d, J = 8.8 Hz, 4H), 7.15 (dd, J = 8.7, 8.8 Hz, 8H), 6.86 (s, 2H), 1.88 (s, 6H).
^{13}C NMR (151 MHz, DMSO-d_6) δ 154.98, 152.75, 137.09, 136.57, 126.10, 125.37, 124.57, 124.32, 97.48, 44.59, 21.49.

UV/Vis (THF): λ_{max} (log ε) = 273 nm (4.98).
MALDI-TOF mass: m/z 508.281 (calculated for $C_{34}H_{35}O_{4+}$ $[M]^+$: 508.261) .

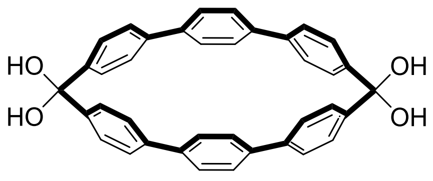

2diols-[1,1][3]PCP

2diols-[1,1][3]PCP: White solid, 13.1 mg, 79 % isolated yield.
^{1}H NMR (600 MHz, DMSO-d6) δ 7.38 (s, 8H), 7.36 (d, J = 8.7 Hz, 8H), 7.31 (d, J = 8.7 Hz, 8H), 6.96 (s, 4H).
^{13}C NMR (151 MHz, DMSO-d6) δ 96.64, 125.49, 125.52, 126.70, 136.40, 136.99, 148.81.
UV/Vis (THF): λ_{max} (log ε) = 280 (4.73).
MALDI-TOF mass: m/z 479.349 (calculated for $C_{27}H_{36}B_2O_6^+$ $[M+H]^+$: 479.277).

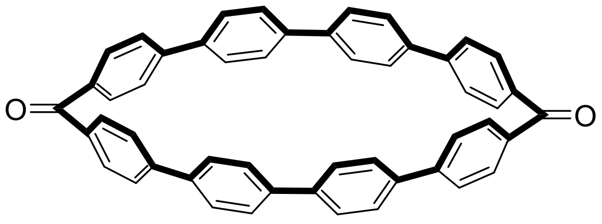

2ketones-[1,1][4]PCP

2ketones-[1,1][4]PCP: White solid, recrystallized from DCM, 13.1 mg, 80 % isolated yield.
^{1}H NMR (600 MHz, CD_2Cl_2) δ 7.63 (d, *J* = 8.4 Hz, 8H), 7.49 (d, *J* = 8.4 Hz, 8H), 7.40 (d, *J* = 8.4 Hz, 8H), 7.20 (d, *J* = 8.4 Hz, 8H).
^{13}C NMR (151 MHz, CD_2Cl_2) δ 127.36, 127.76, 127.96, 128.60, 138.97, 139.56, 140.71, 143.26, 200.83.
UV/Vis (THF): λ_{max} (log ε) = 317 (5.04).
MALDI-TOF mass: m/z 664.247 (calculated for $C_{50}H_{32}O_2^+$ $[M]^+$: 664.240).

2.7 Synthesis of Compound 2ketones-[1,1][3]PCP.

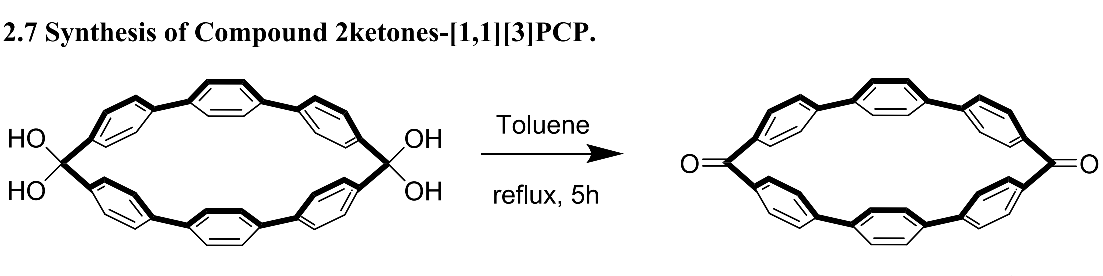

2diols-[1,1][3]PCP ***2ketones-[1,1][3]PCP***

Compound **2diols-[1,1][3]PCP** (20 mg, 0.03 mmol) was added to a reaction tube, which was then evacuated and refilled with nitrogen. Dry toluene was added to the tube, and the reaction mixture was stirred under reflux conditions for 5 h. After completion of the reaction, the mixture was cooled

to room temperature and filtered to obtain a solid. The resulting solid was recrystallized using a mixture of dichloromethane/*n*-hexane to afford compound **2ketones-[1,1][3]PCP** as a white solid (14.6 mg, 78 % isolated yield).

2ketones-[1,1][3]PCP: ^{1}**H NMR** (600 MHz, $CDCl_3$) δ 7.47 (s, 8H), 7.37 (d, *J* = 8.2 Hz, 8H), 7.16 (d, *J* = 8.2 Hz, 8H).

^{13}**C NMR** (151 MHz, $CDCl_3$) δ 126.21, 127.31, 128.07, 138.49, 140.92, 141.17, 201.20.

MALDI-TOF mass: m/z 513.138 (calculated for $C_{38}H_{25}O_2^+$ [M+H]$^+$: 513.184).

3. NMR Spectra

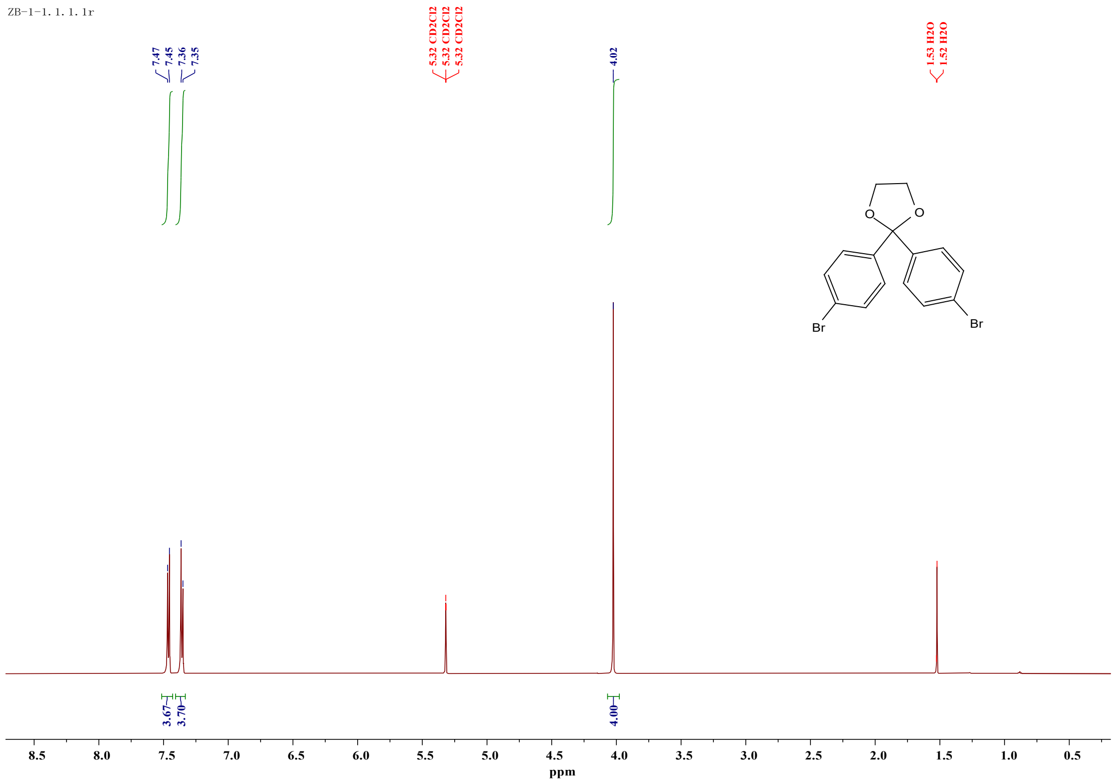

Figure S1. ^{1}H NMR spectrum of **S1** (600 MHz, CD_2Cl_2, 298 K).

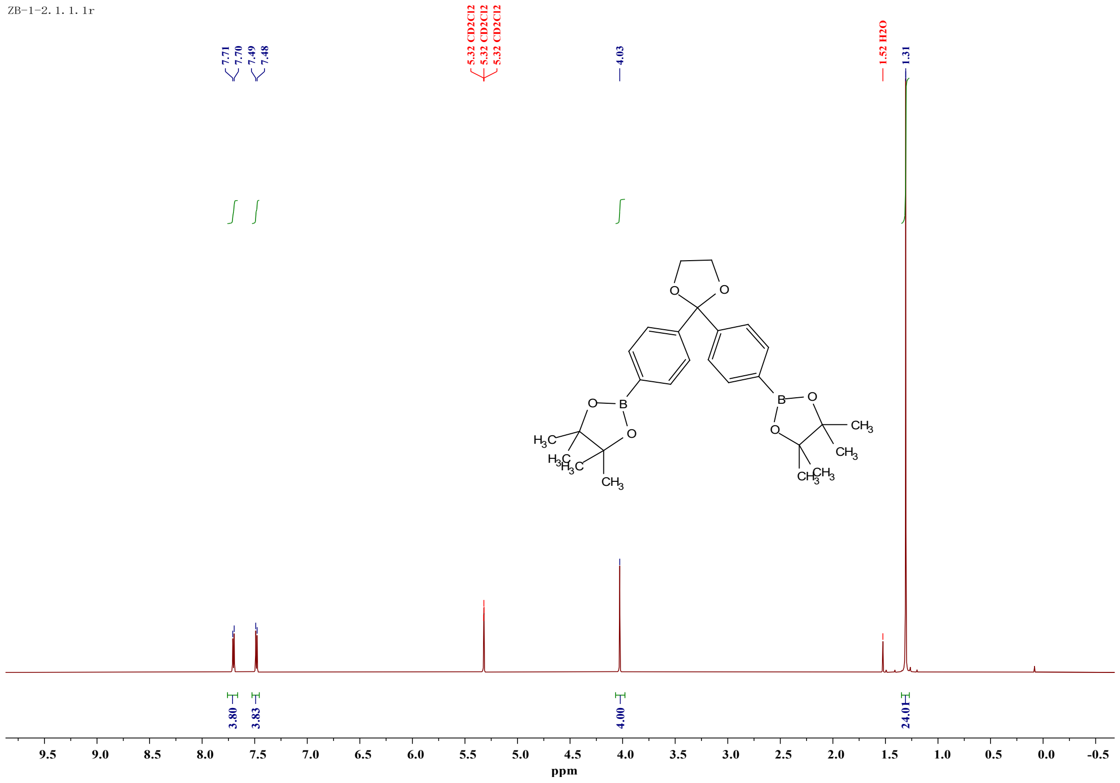

Figure S2. ^{1}H NMR spectrum of **S2** (600 MHz, CD_2Cl_2, 298 K).

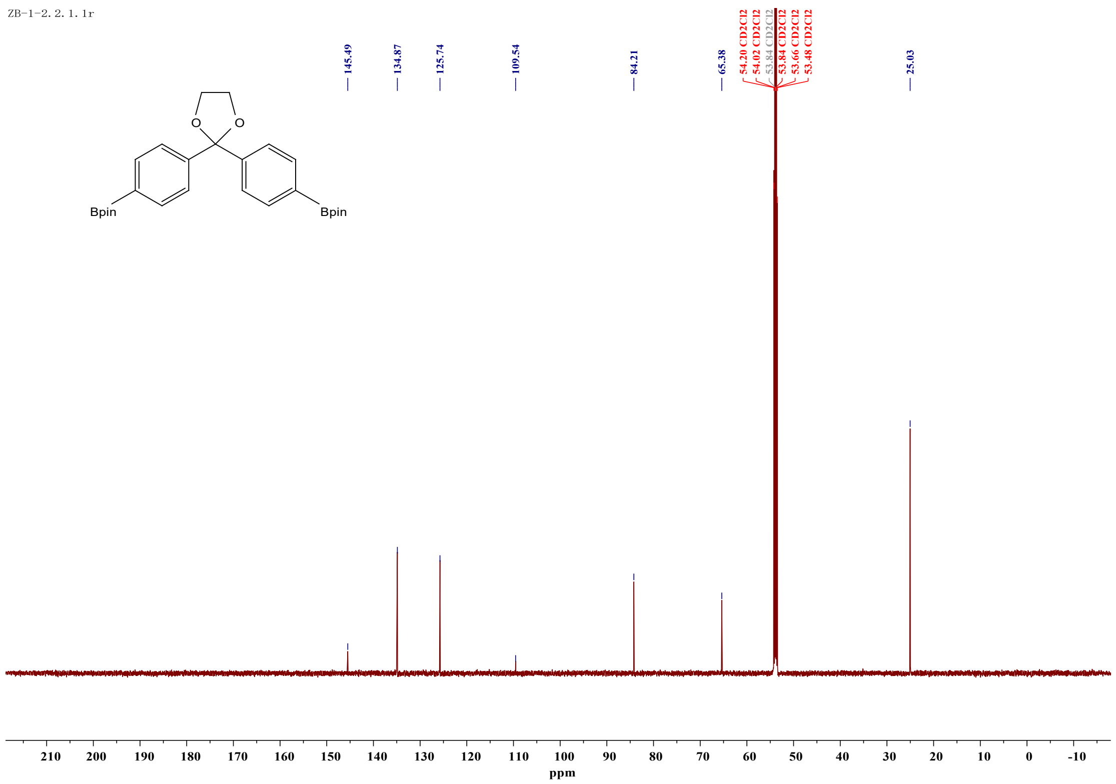

Figure S3. ^{13}C NMR spectrum of **S2** (151 MHz, CD_2Cl_2, 298 K).

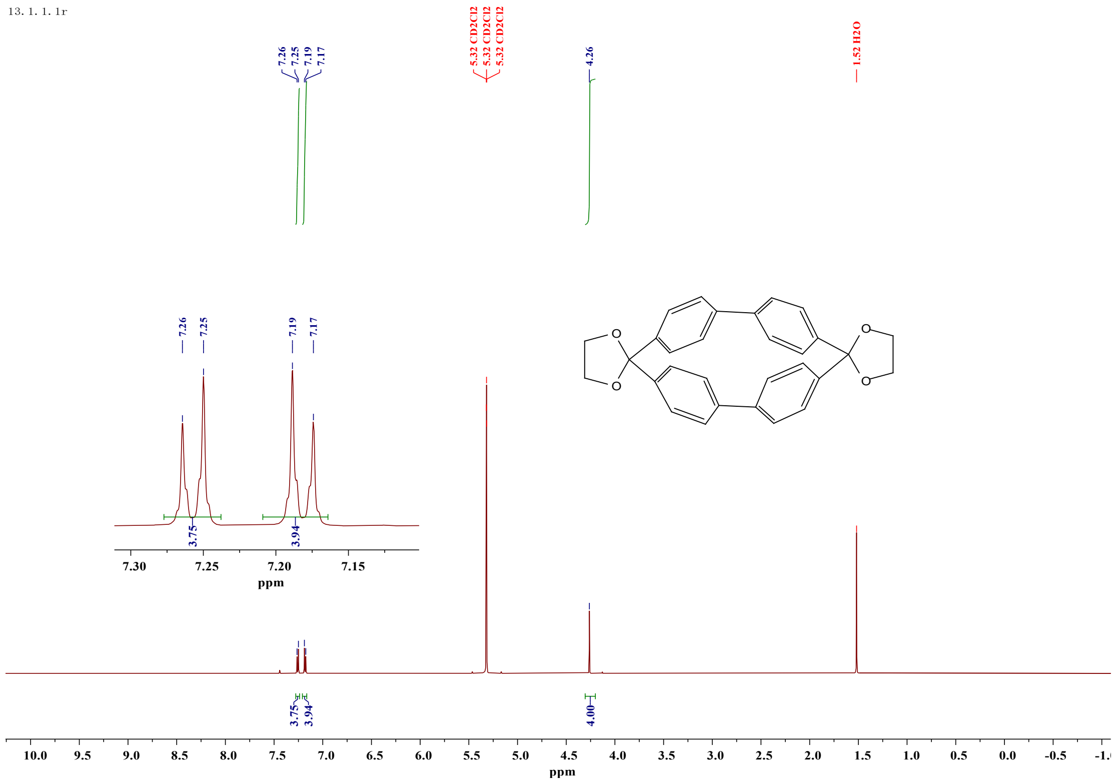

Figure S4. ^{1}H NMR spectrum of **2dbts-[1,1][2]PCP** (600 MHz, CD_2Cl_2, 298 K).

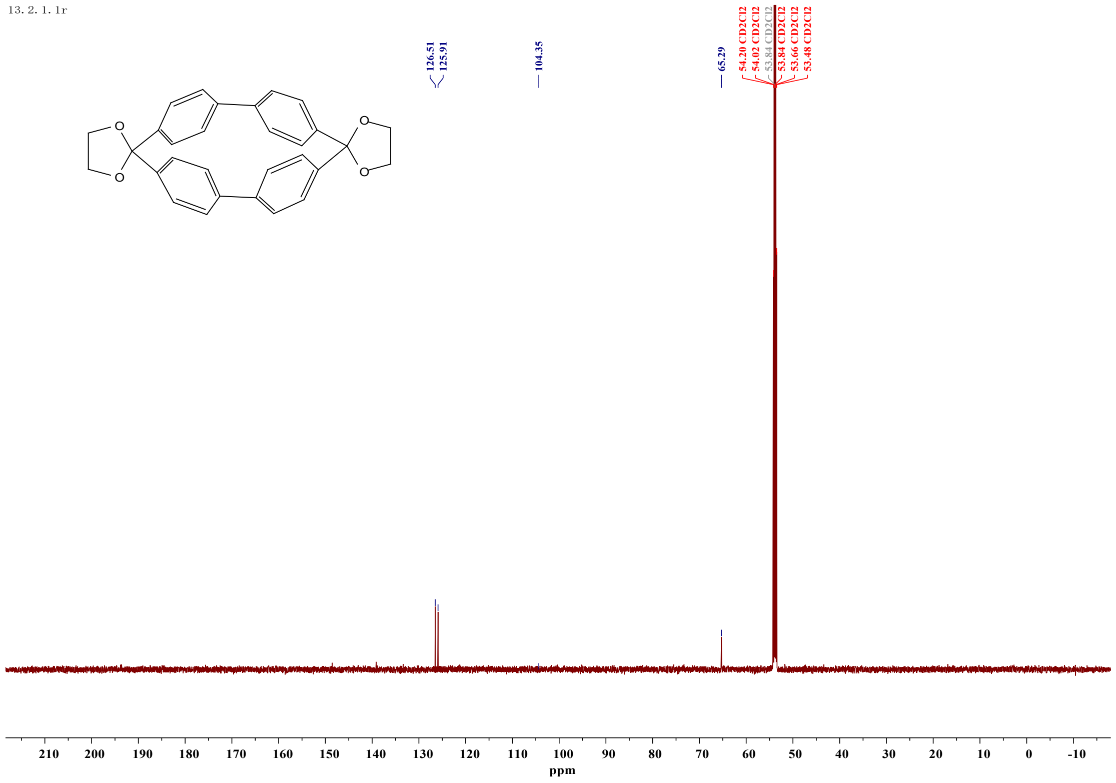

Figure S5. ^{13}C NMR spectrum of **2dbts-[1,1][2]PCP** (151 MHz, CD_2Cl_2, 298 K).

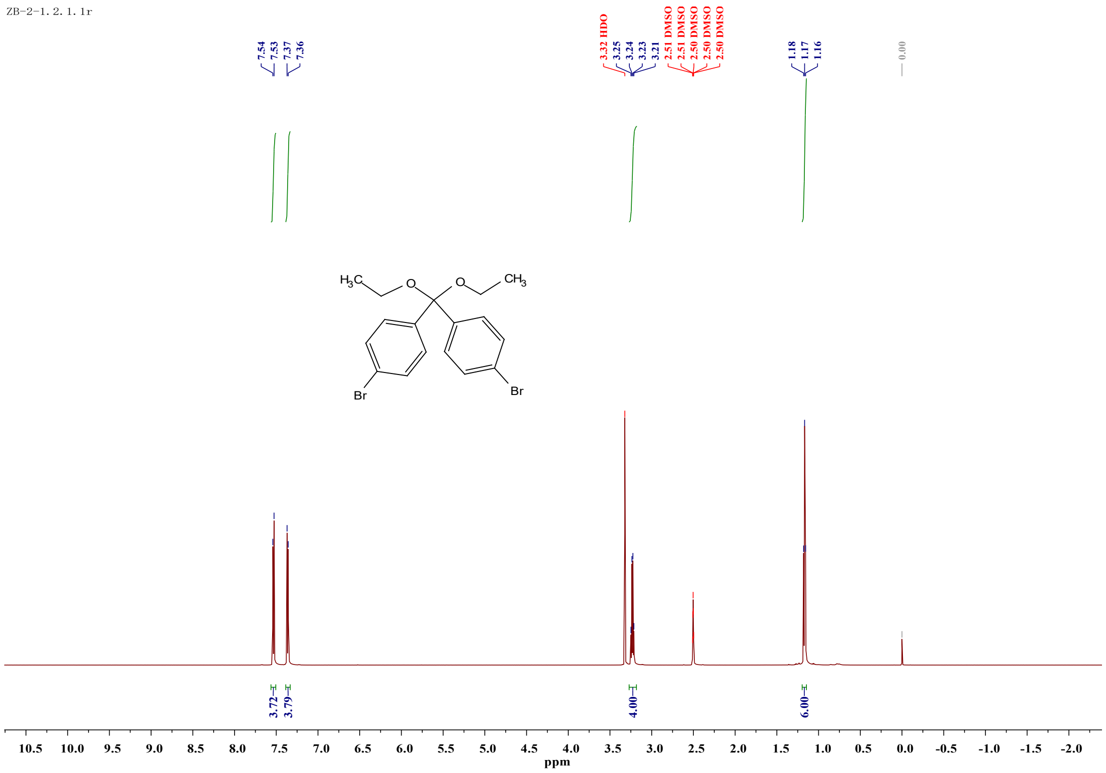

Figure S6. ¹H NMR spectrum of **S3** (600 MHz, DMSO-d_6, 298 K).

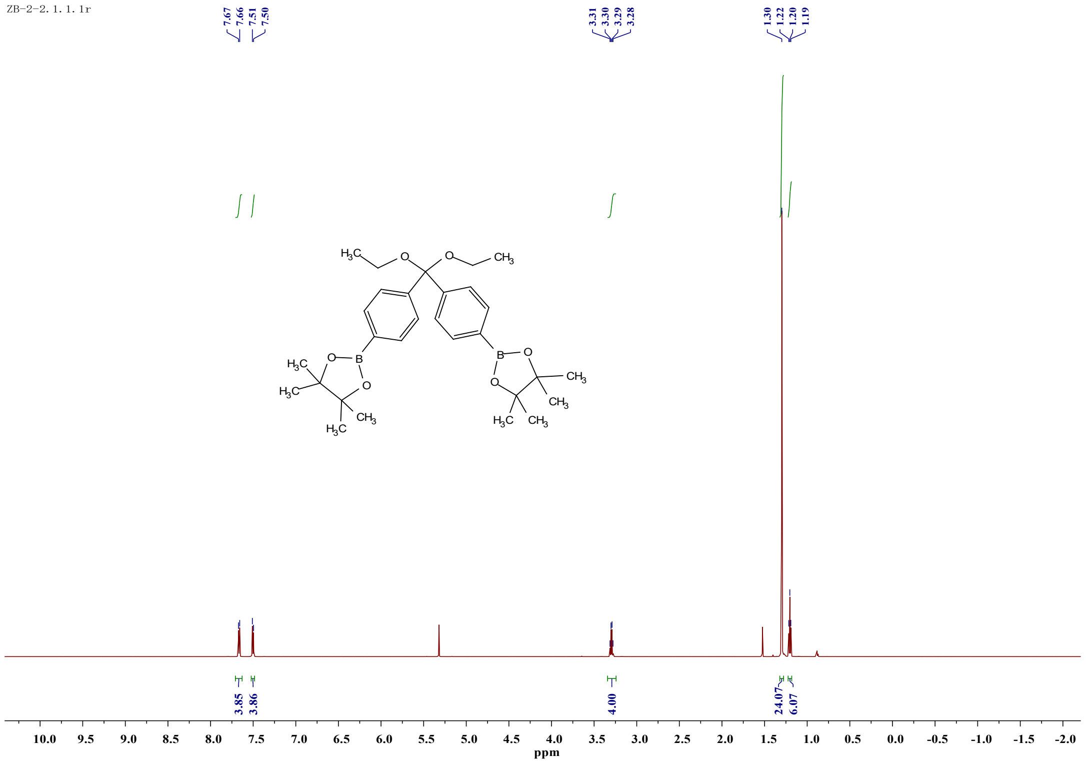

Figure S7. ¹H NMR spectrum of **S4** (151 MHz, CD_2Cl_2, 298 K).

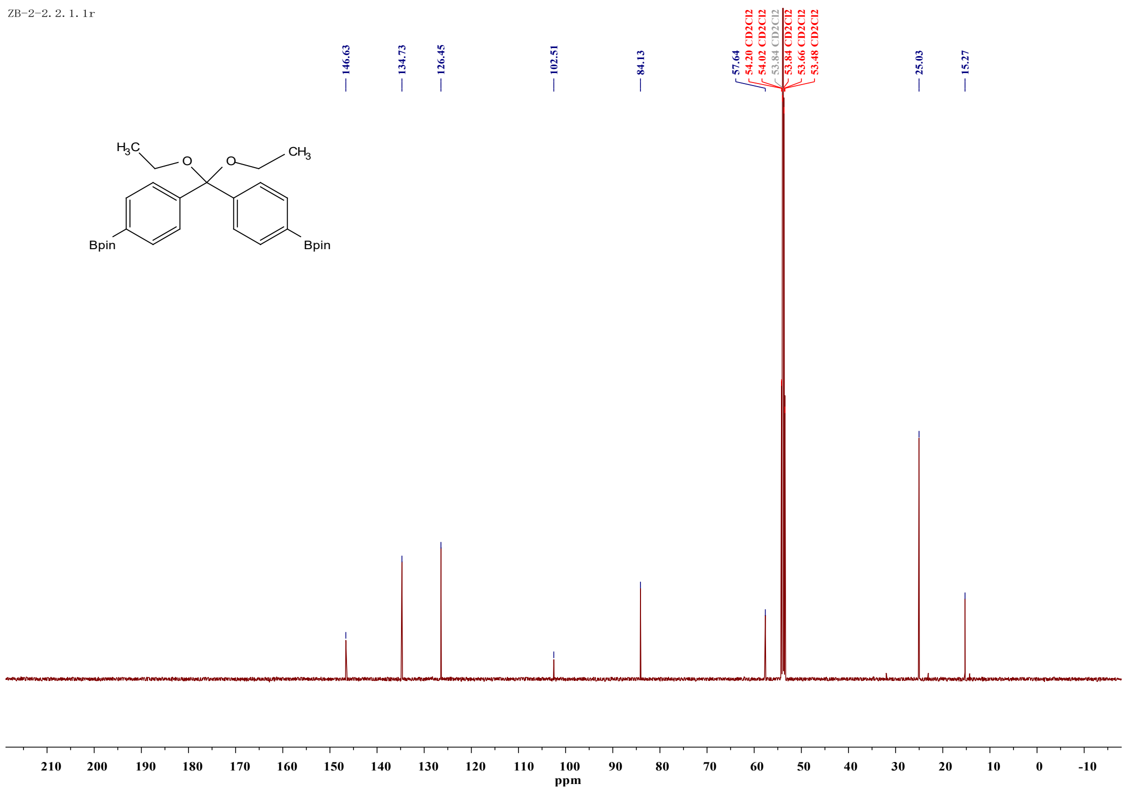

Figure S8. ^{13}C NMR spectrum of **S4** (151 MHz, CD_2Cl_2, 298 K).

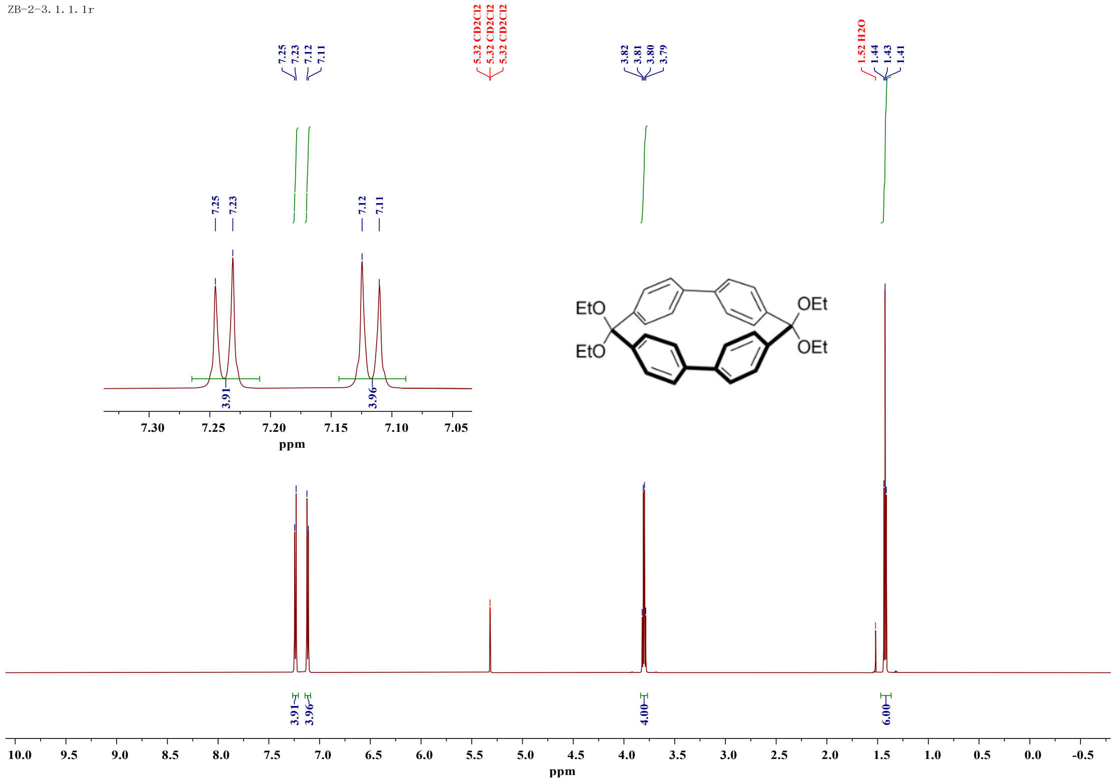

Figure S9. ^{1}H NMR spectrum of **2ketals-[1,1][2]PCP** (600 MHz, CD_2Cl_2, 298 K).

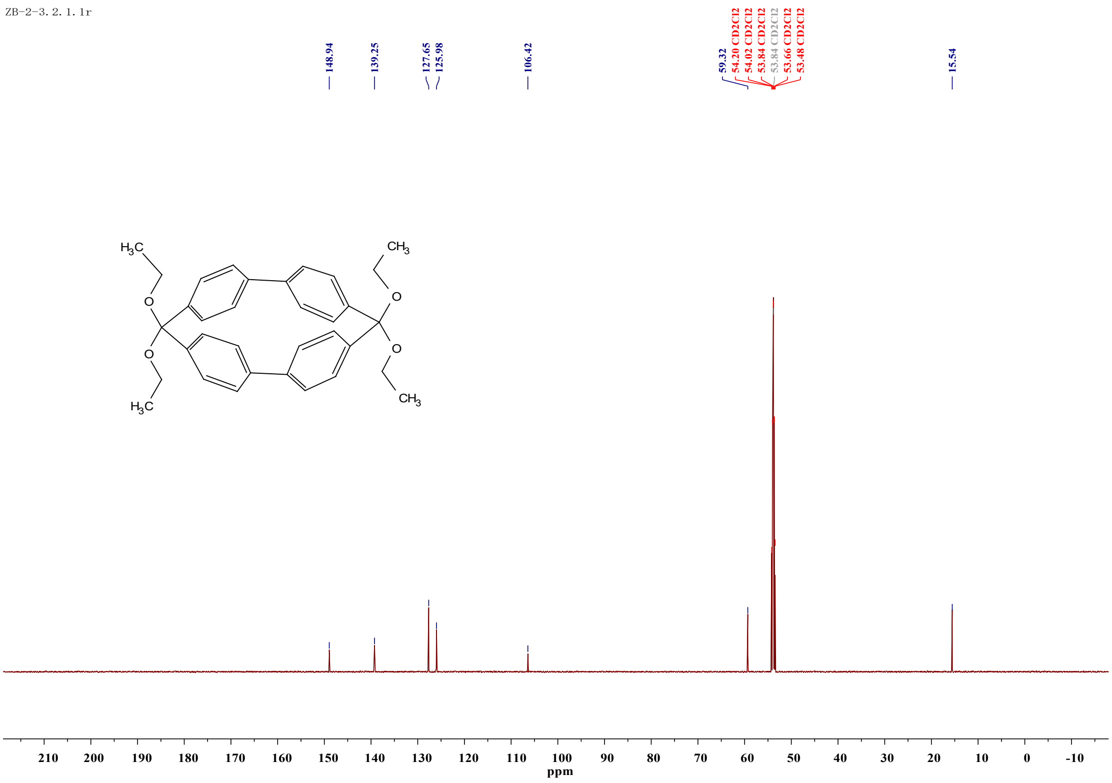

Figure S10. ^{13}C NMR spectrum of **2ketals-[1,1][2]PCP** (151 MHz, CD_2Cl_2, 298 K).

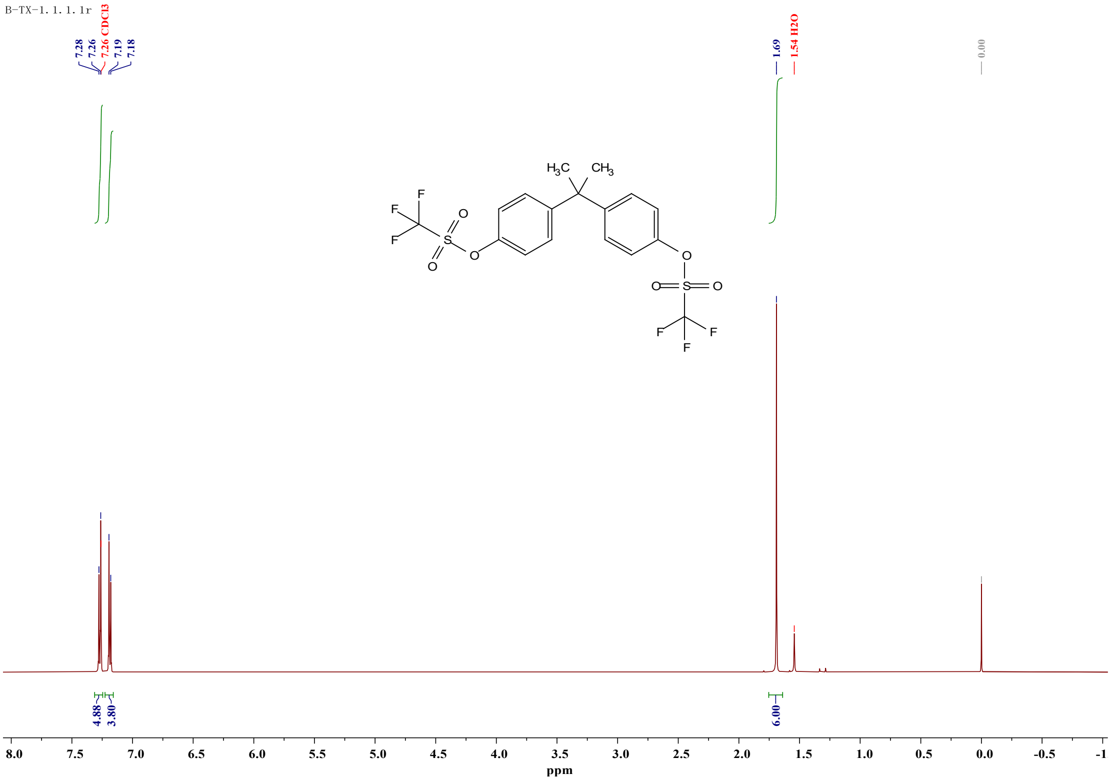

Figure S11. ^{1}H NMR spectrum of **S5** (600 MHz, $CDCl_3$, 298 K).

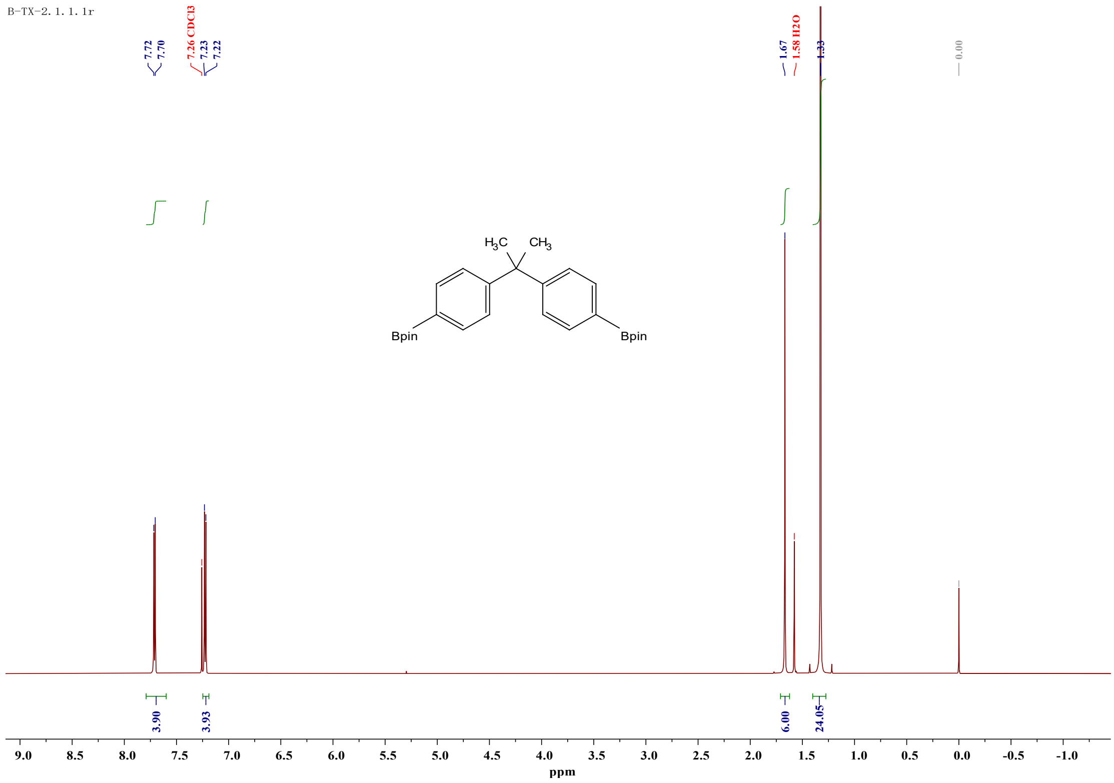

Figure S12. ^{1}H NMR spectrum of **S6** (151 MHz, $CDCl_3$, 298 K).

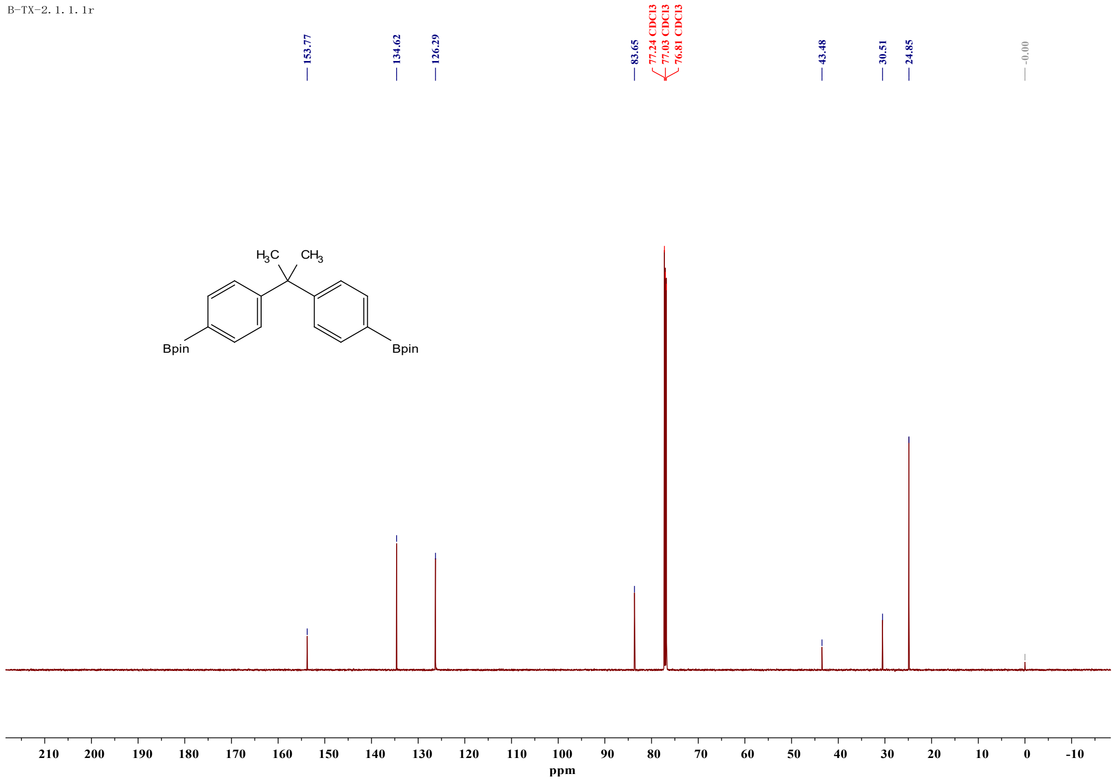

Figure S13. ^{13}C NMR spectrum of **S6** (600 MHz, $CDCl_3$, 298 K).

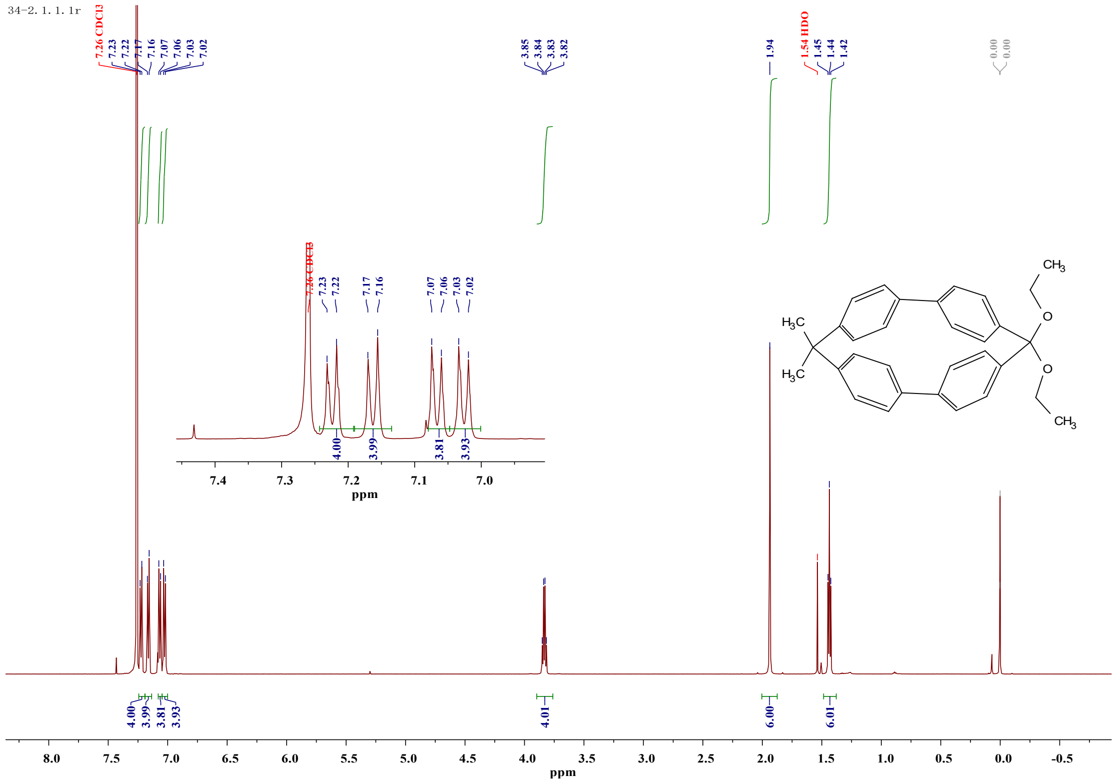

Figure S14. ^{1}H NMR spectrum of **ketal-Me-[1,1][2]PCP** (600 MHz, $CDCl_3$, 298 K).

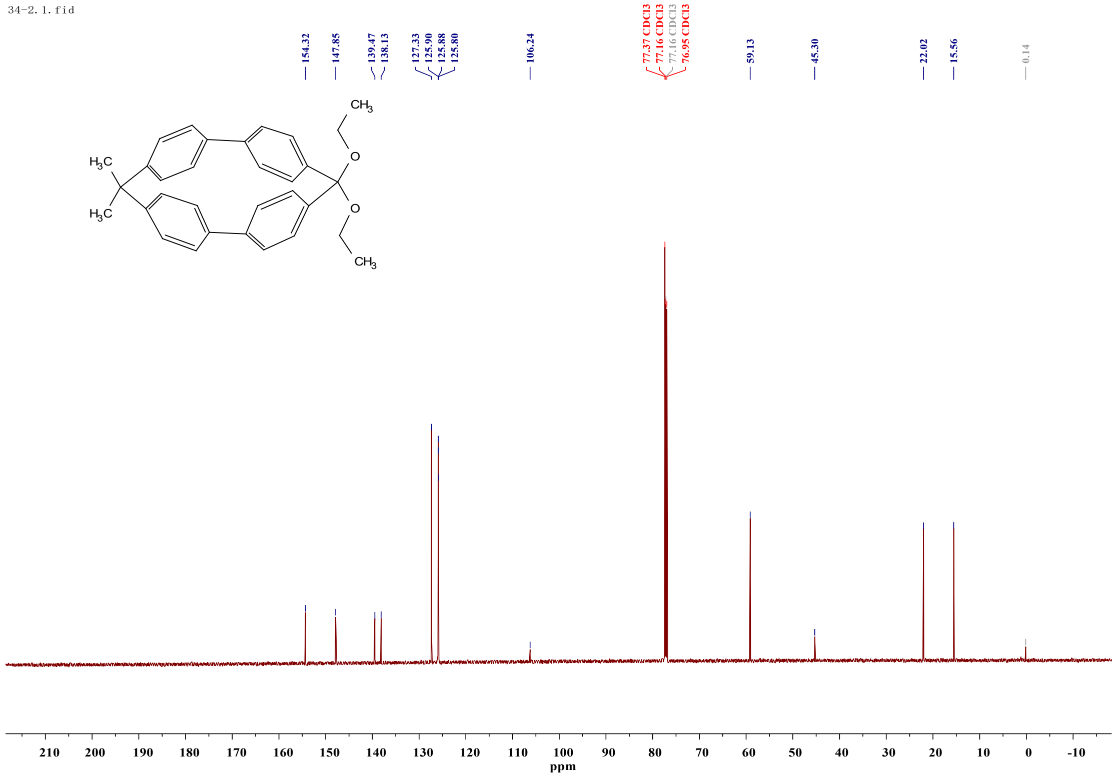

Figure S15. ^{13}C NMR spectrum of **ketal-Me-[1,1][2]PCP** (151 MHz, $CDCl_3$, 298 K).

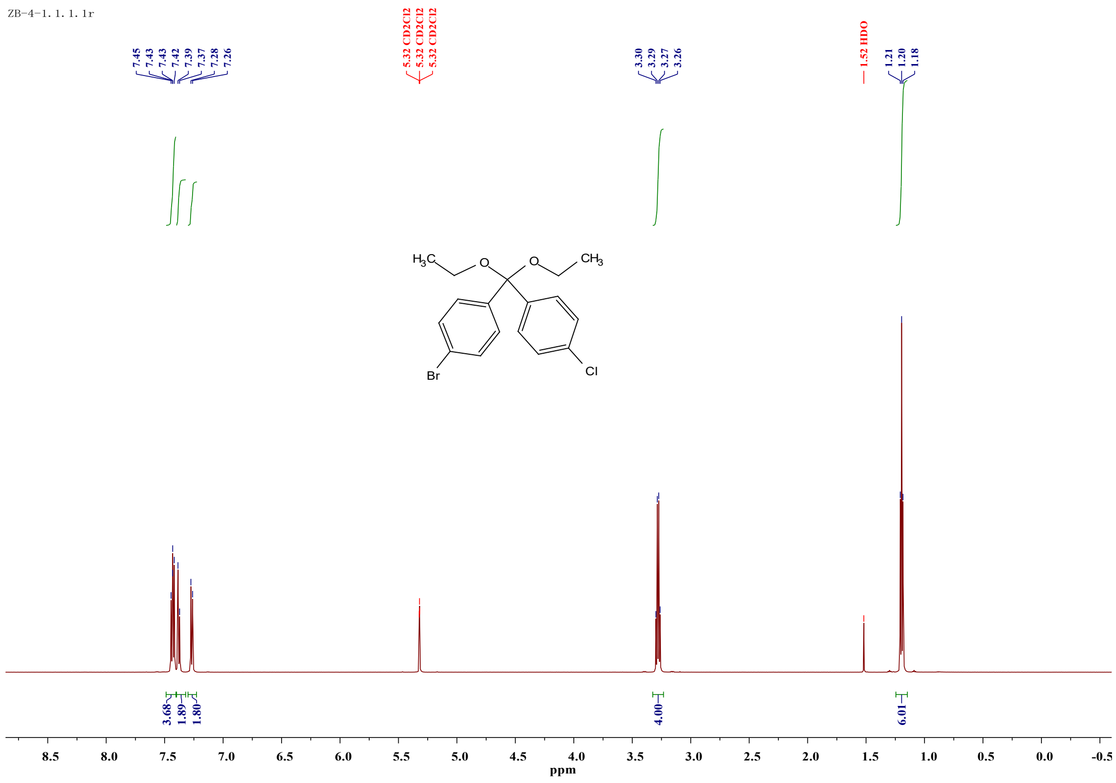

Figure S16. ^{1}H NMR spectrum of **S7** (600 MHz, CD_2Cl_2, 298 K).

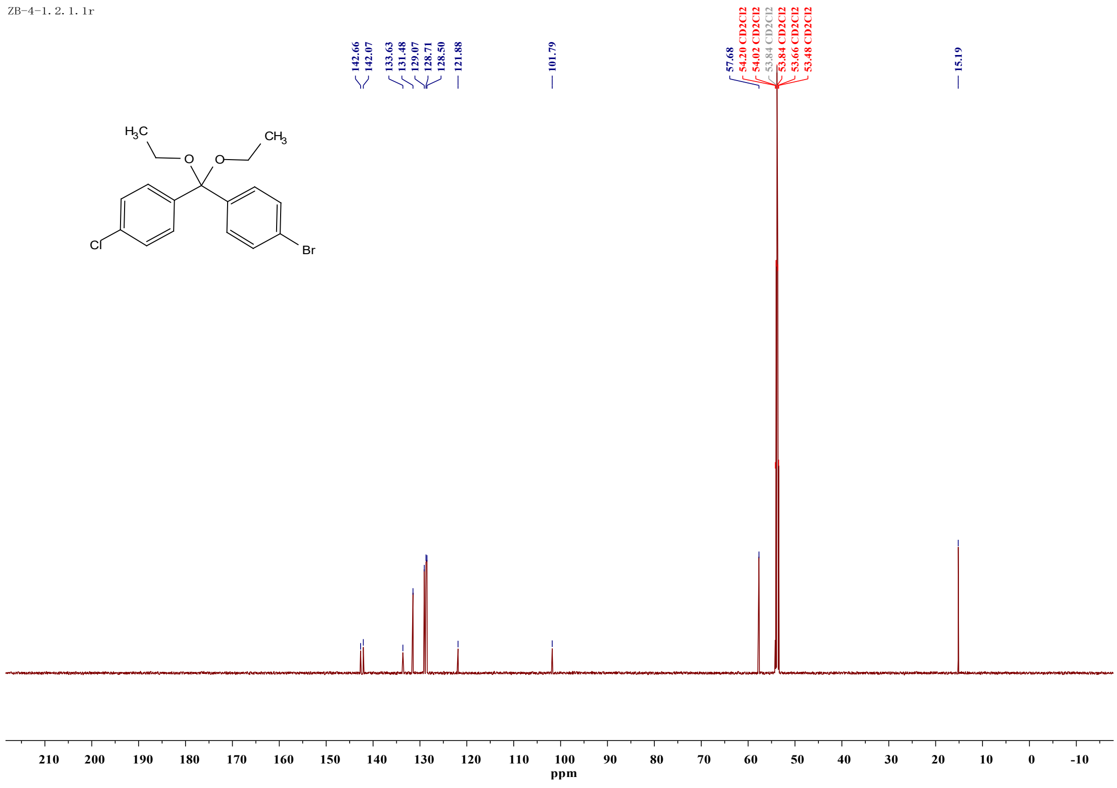

Figure S17. ^{13}C NMR spectrum of **S7** (151 MHz, CD_2Cl_2, 298 K).

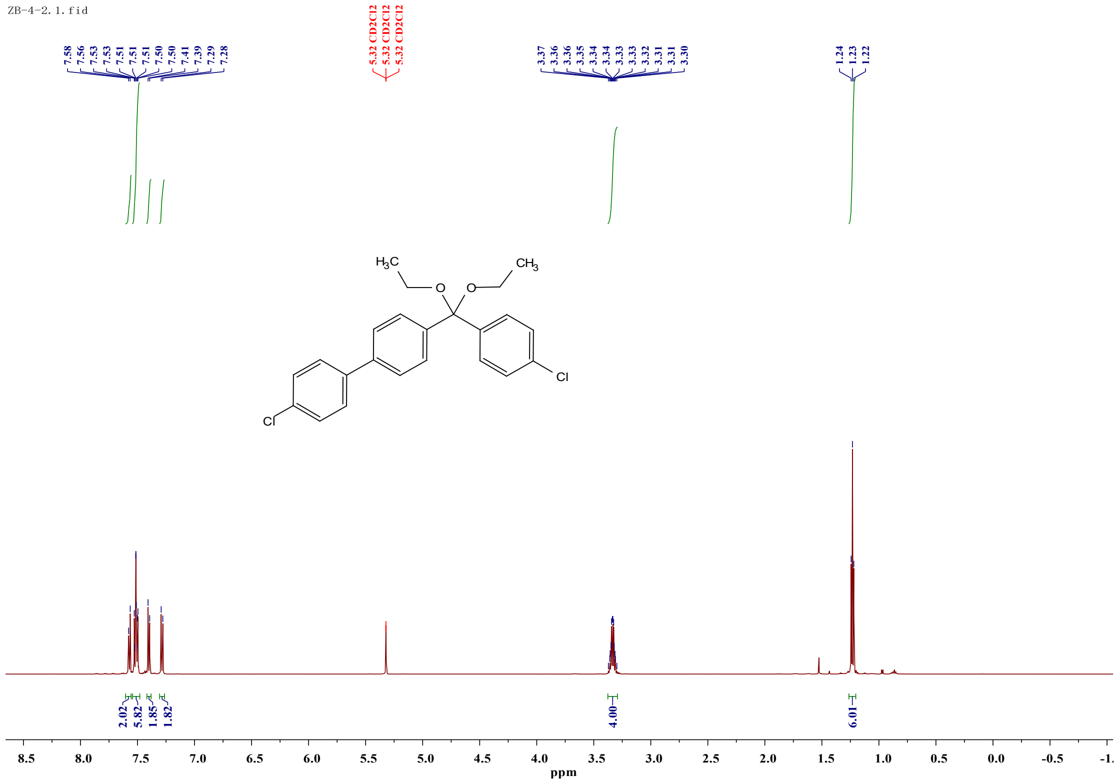

Figure S18. ^{1}H NMR spectrum of **S8** (600 MHz, CD_2Cl_2, 298 K).

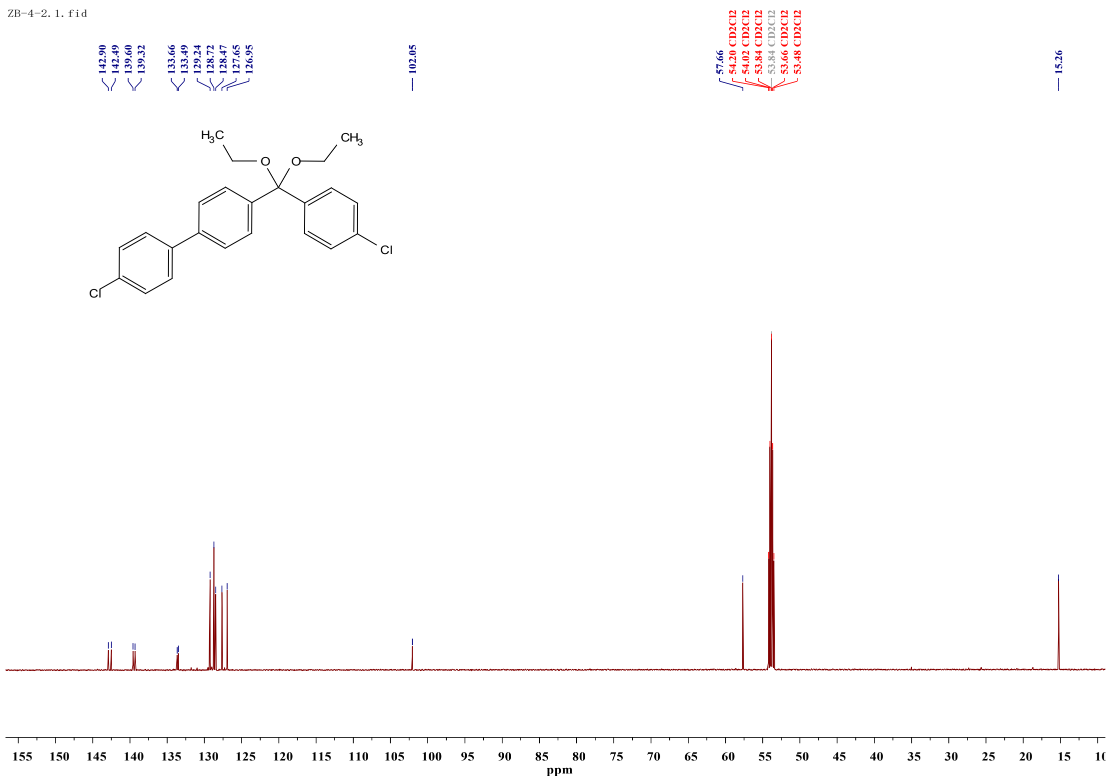

Figure S19. ^{13}C NMR spectrum of **S8** (151 MHz, CD_2Cl_2, 298 K).

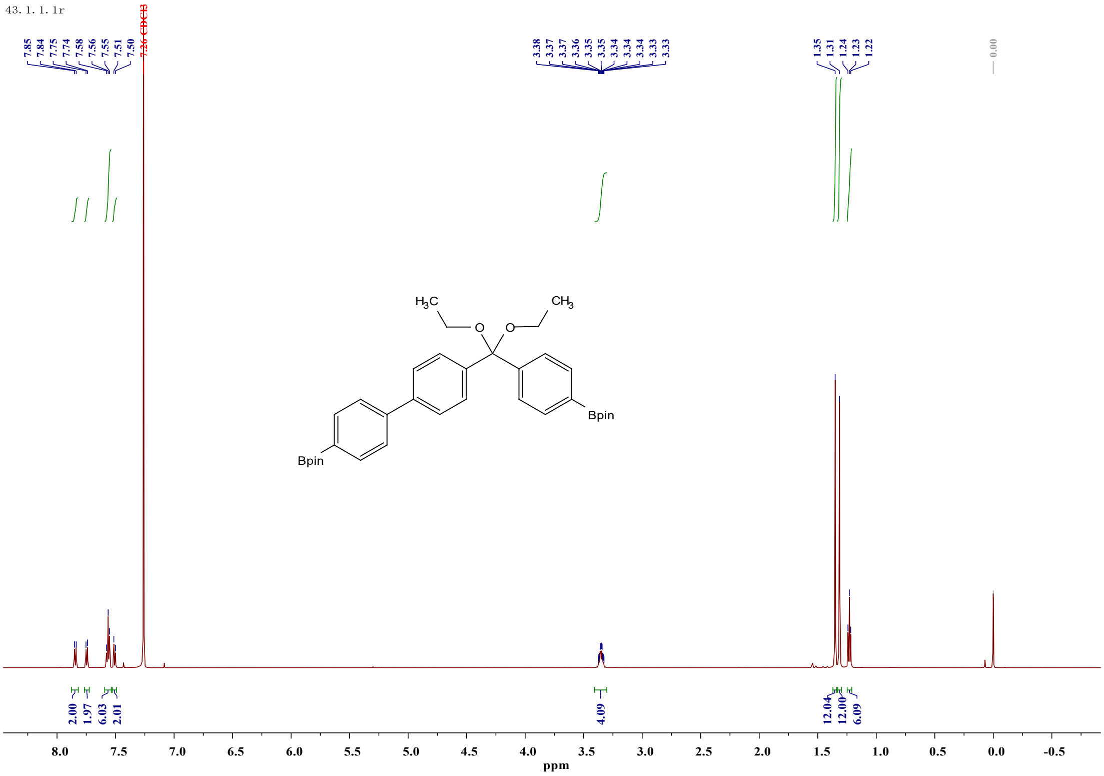

Figure S20. ^{1}H NMR spectrum of **S9** (600 MHz, $CDCl_3$, 298 K).

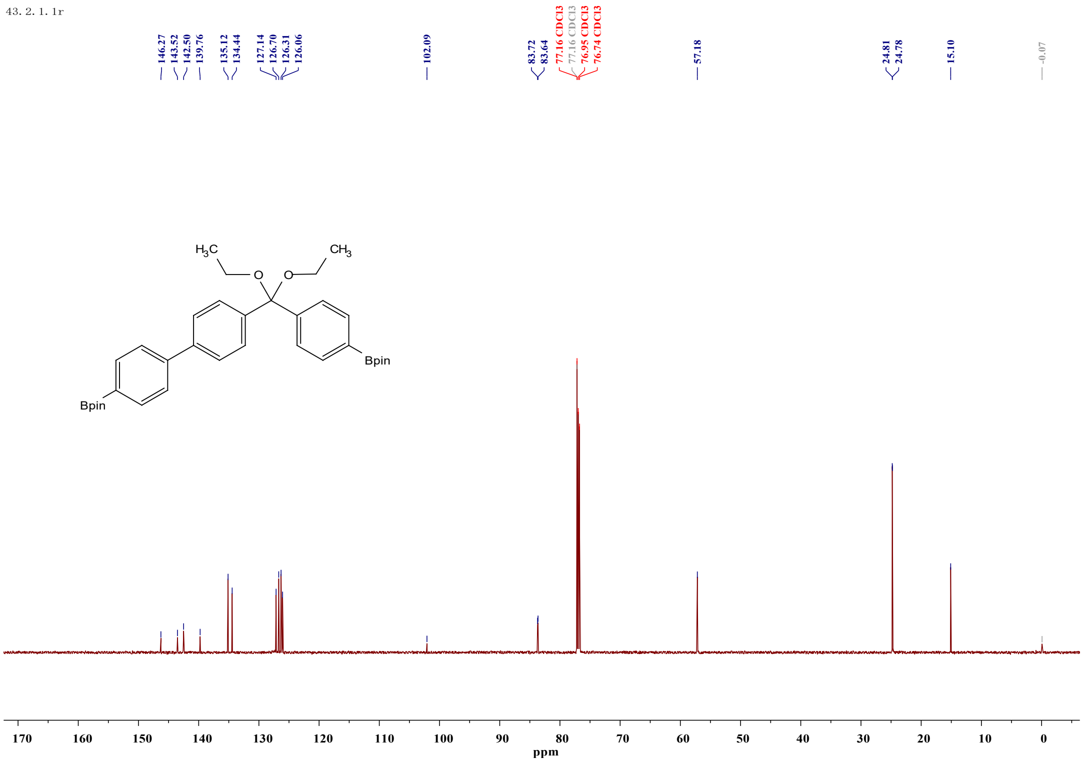

Figure S21. ^{13}C NMR spectrum of **S9** (151 MHz, $CDCl_3$, 298 K).

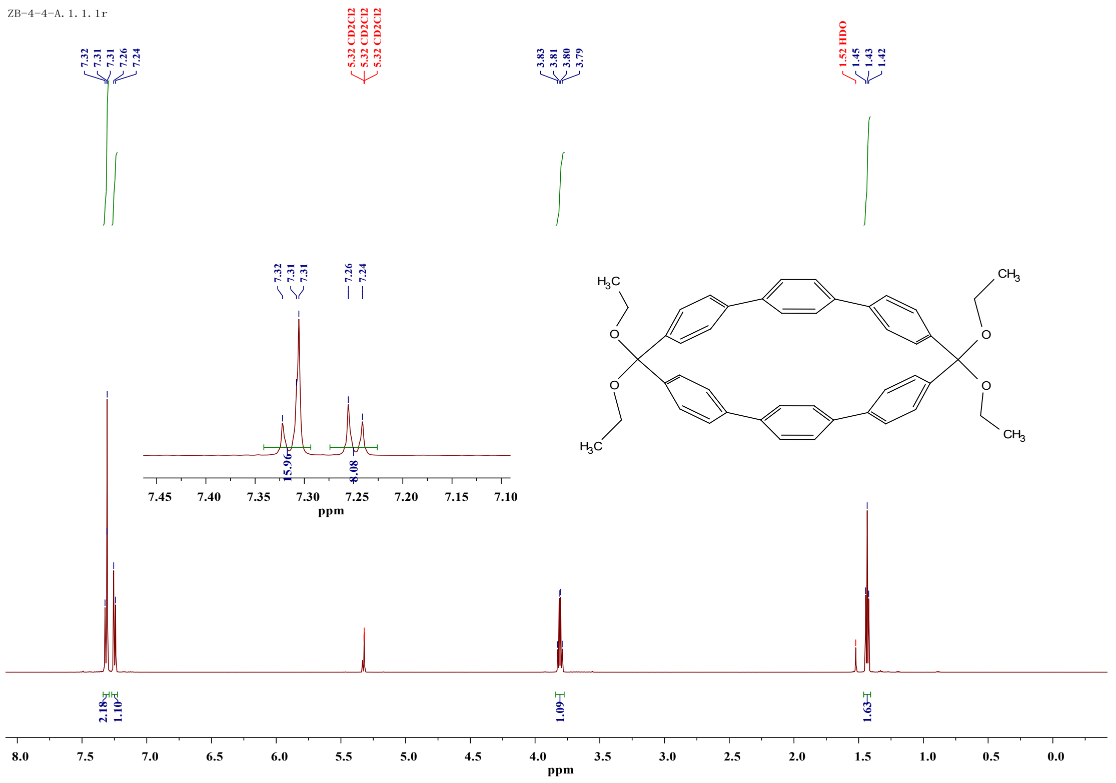

Figure S22. ^{1}H NMR spectrum of **2ketals-[1,1][3]PCP** (600 MHz, CD_2Cl_2, 298 K).

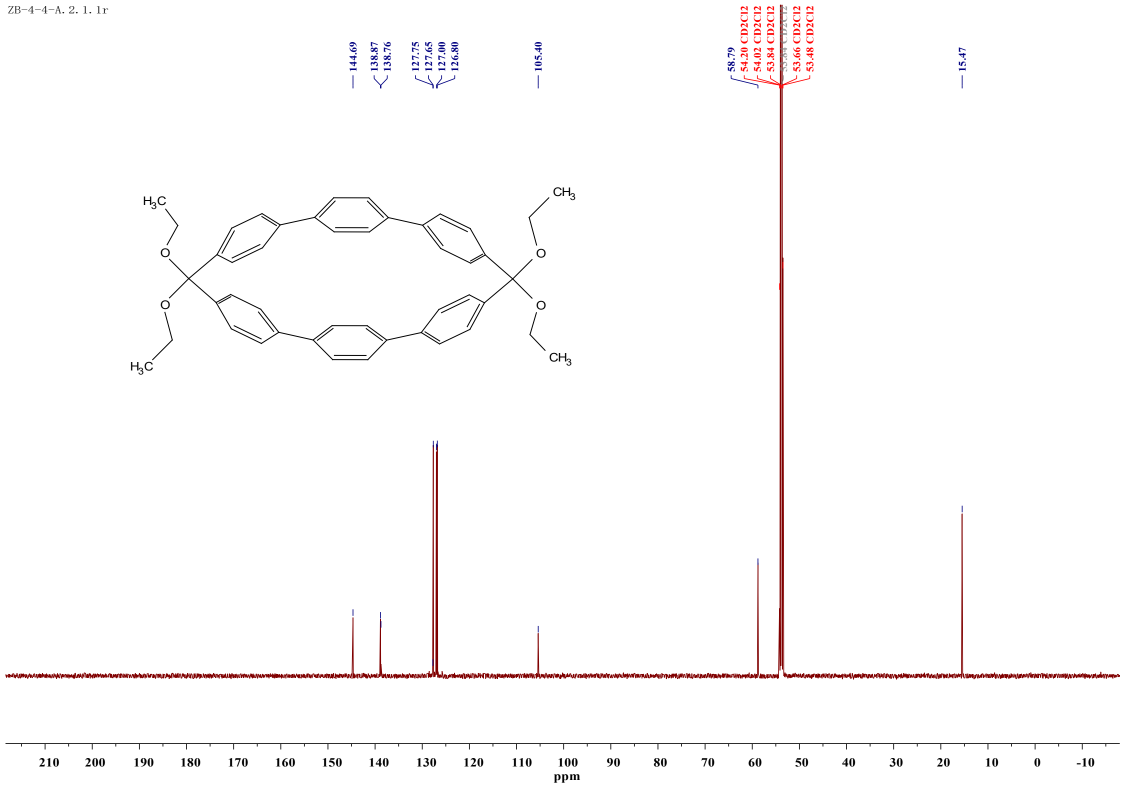

Figure S23. ^{13}C NMR spectrum of **2ketals-[1,1][3]PCP** (151 MHz, CD_2Cl_2, 298 K).

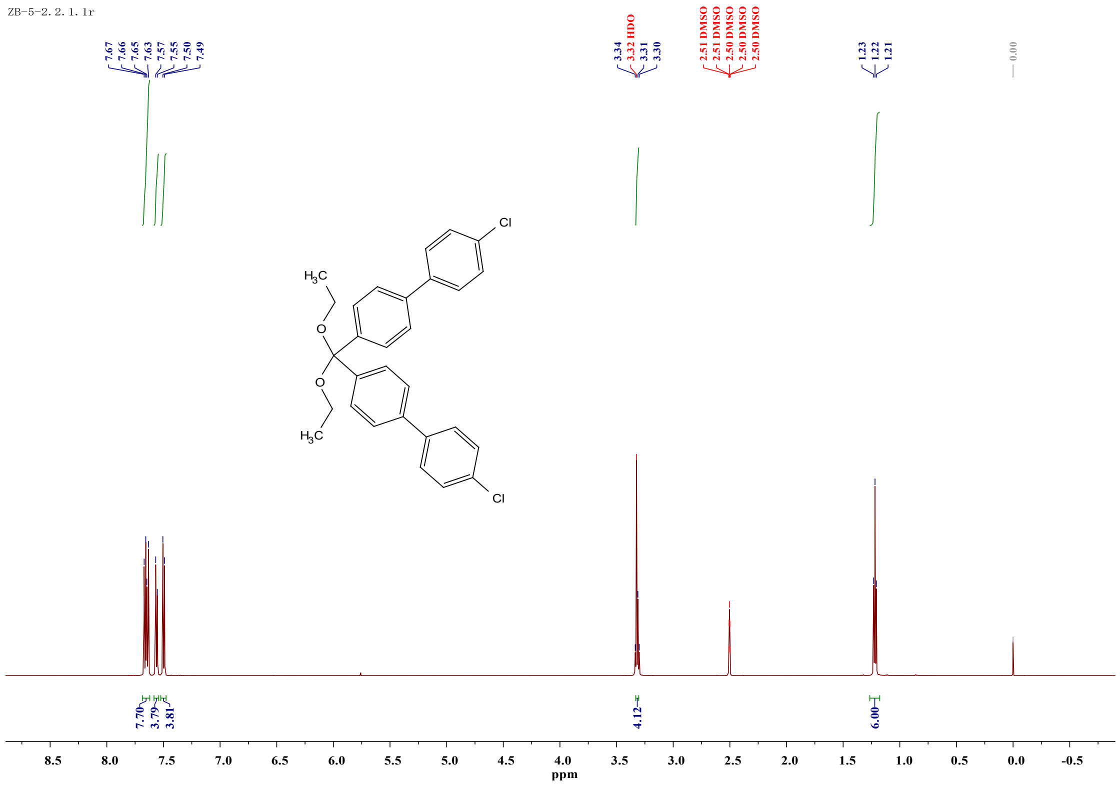

Figure S24. ^{1}H NMR spectrum of **S10** (600 MHz, DMSO-d_6, 298 K).

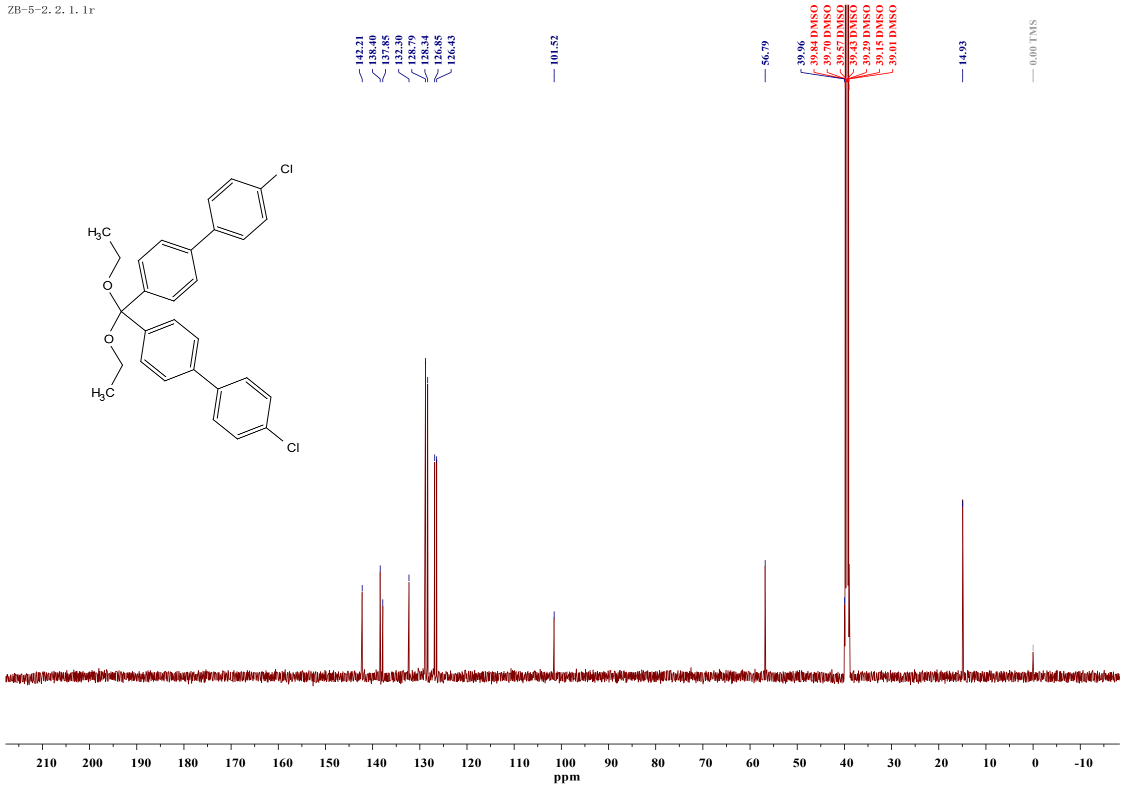

Figure S25. ^{13}C NMR spectrum of **S10** (600 MHz, DMSO-d_6, 298 K).

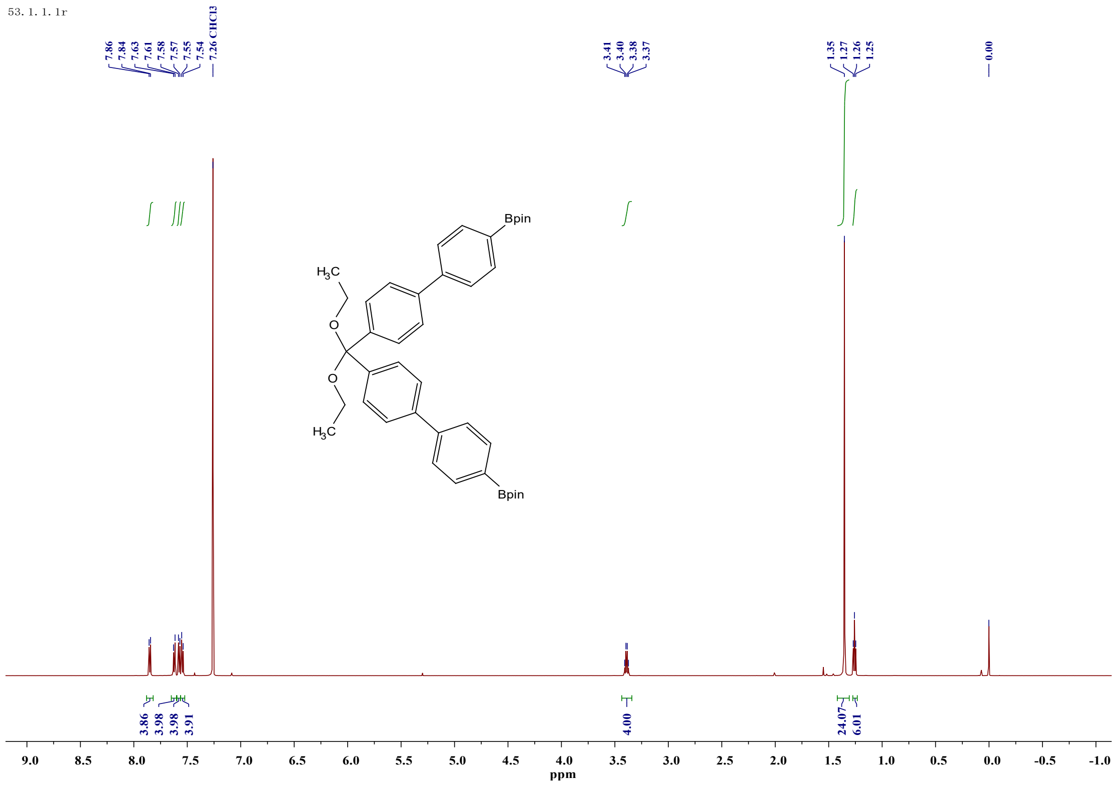

Figure S26. ^{1}H NMR spectrum of **S11** (600 MHz, $CDCl_3$, 298 K).

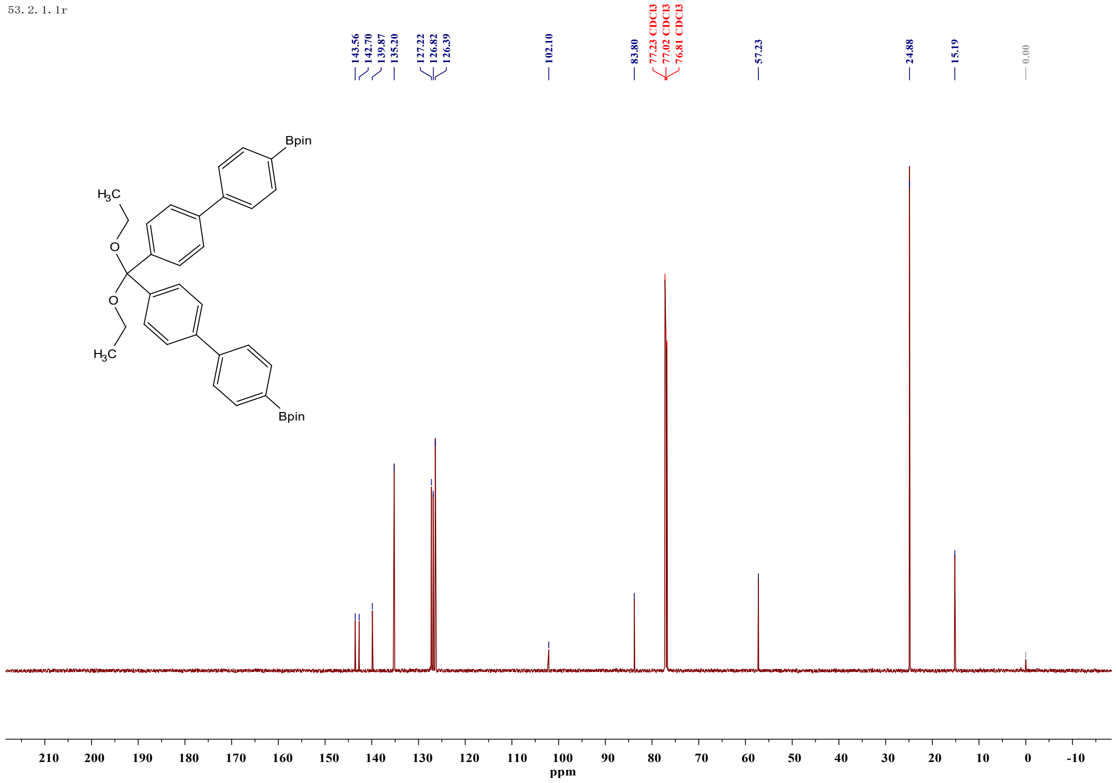

Figure S27. ^{13}C NMR spectrum of **S11** (151 MHz, $CDCl_3$, 298 K).

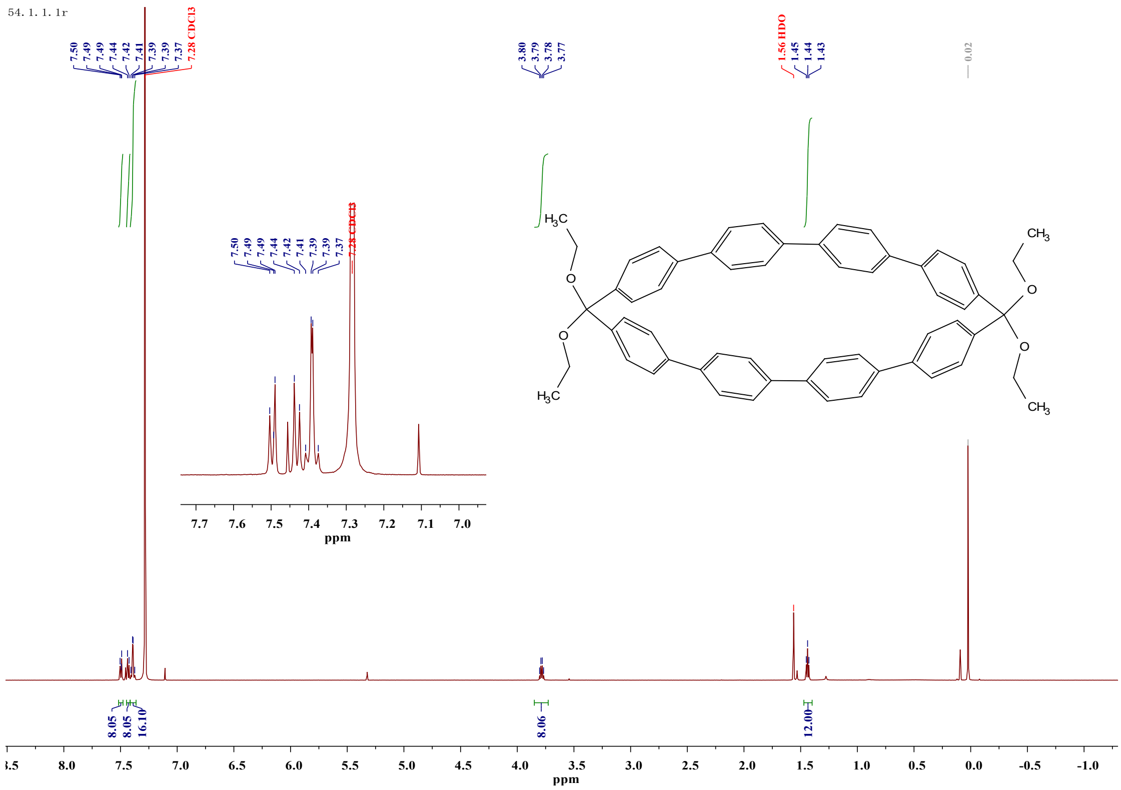

Figure S28. ^{1}H NMR spectrum of **2ketals-[1,1][4]PCP** (600 MHz, $CDCl_3$, 298 K).

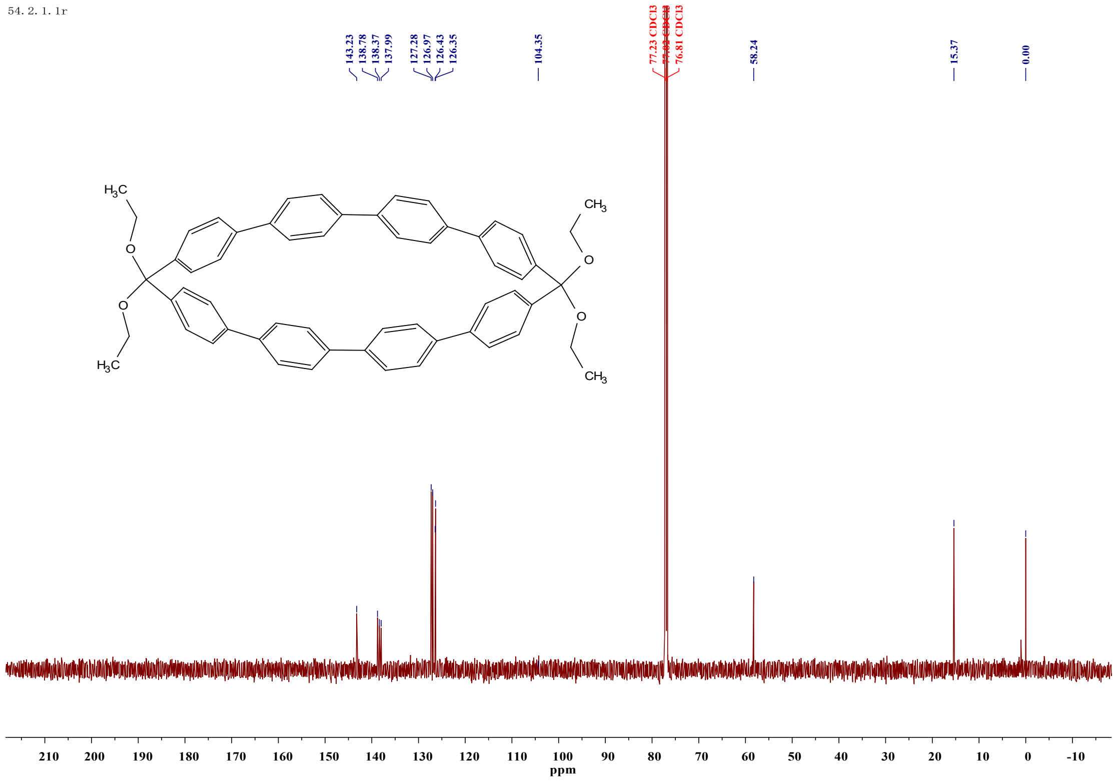

Figure S29. ^{13}C NMR spectrum of **2ketals-[1,1][4]PCP** (151 MHz, $CDCl_3$, 298 K).

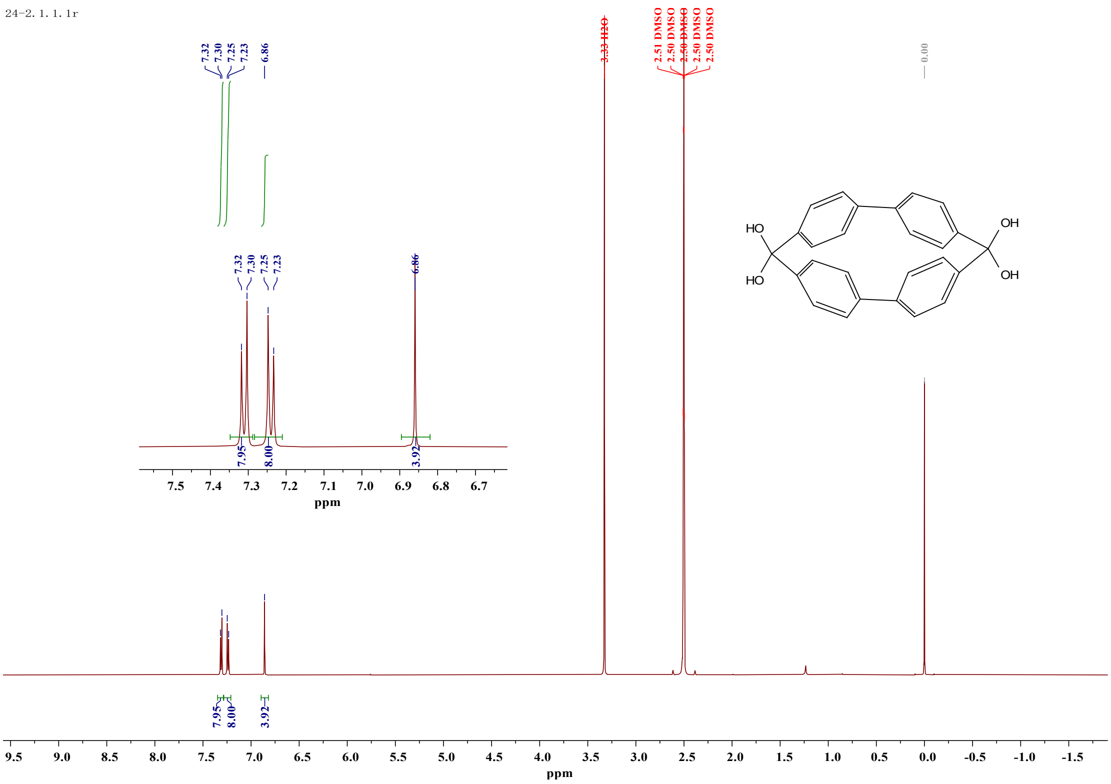

Figure S30. ^{1}H NMR spectrum of **2diols-[1,1][2]PCP** (600 MHz, DMSO-d_6, 298 K).

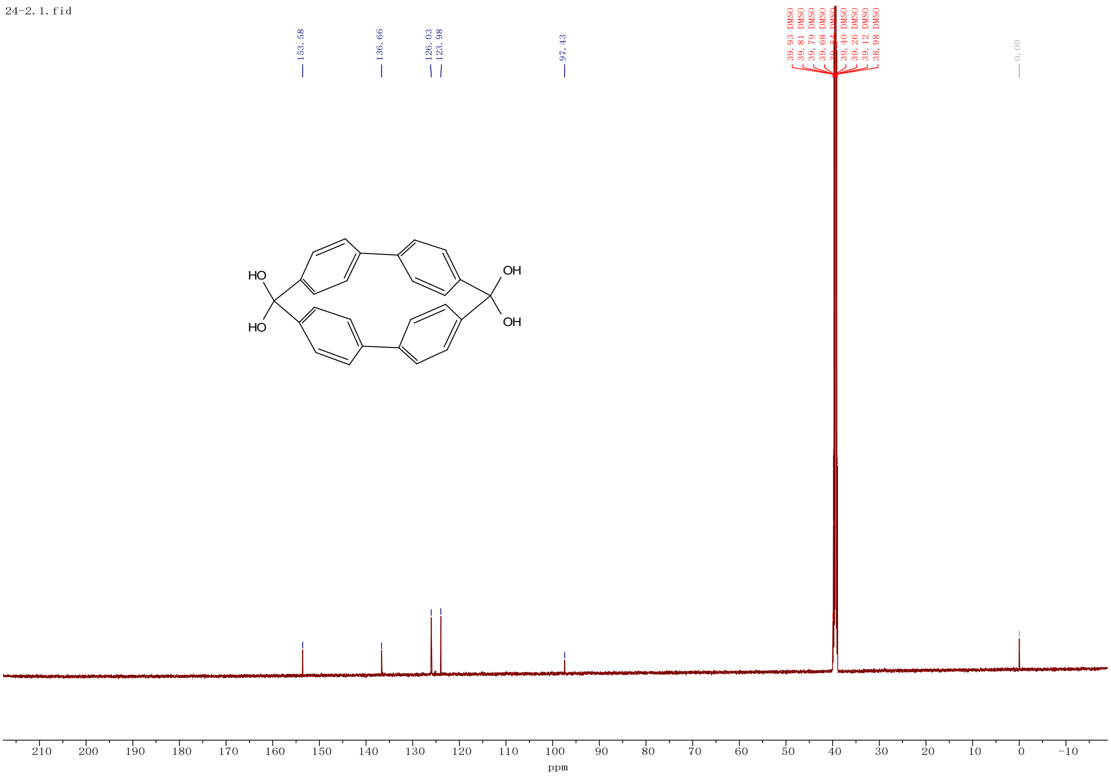

Figure S31. ^{13}C NMR spectrum of **2diols-[1,1][2]PCP** (151 MHz, DMSO-d_6, 298 K).

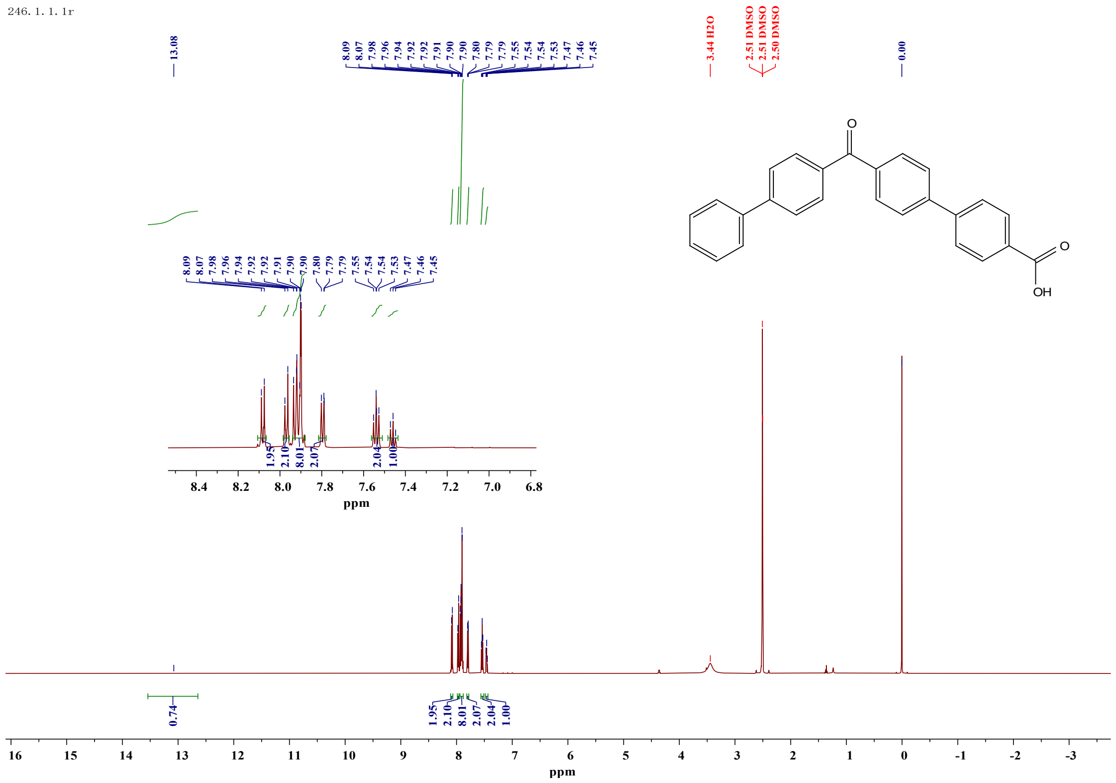

Figure S32. ^{1}H NMR spectrum of **1** (600 MHz, DMSO-d_6, 298 K).

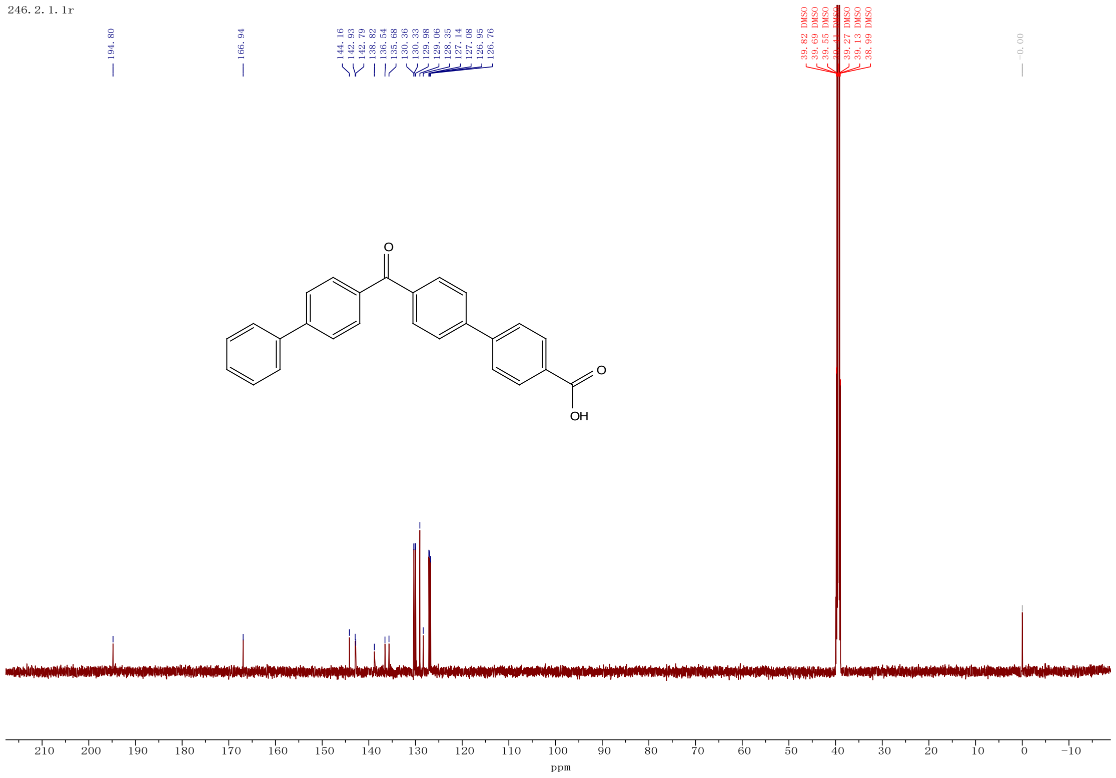

Figure S33. ^{13}C NMR spectrum of **1** (600 MHz, DMSO-d_6, 298 K).

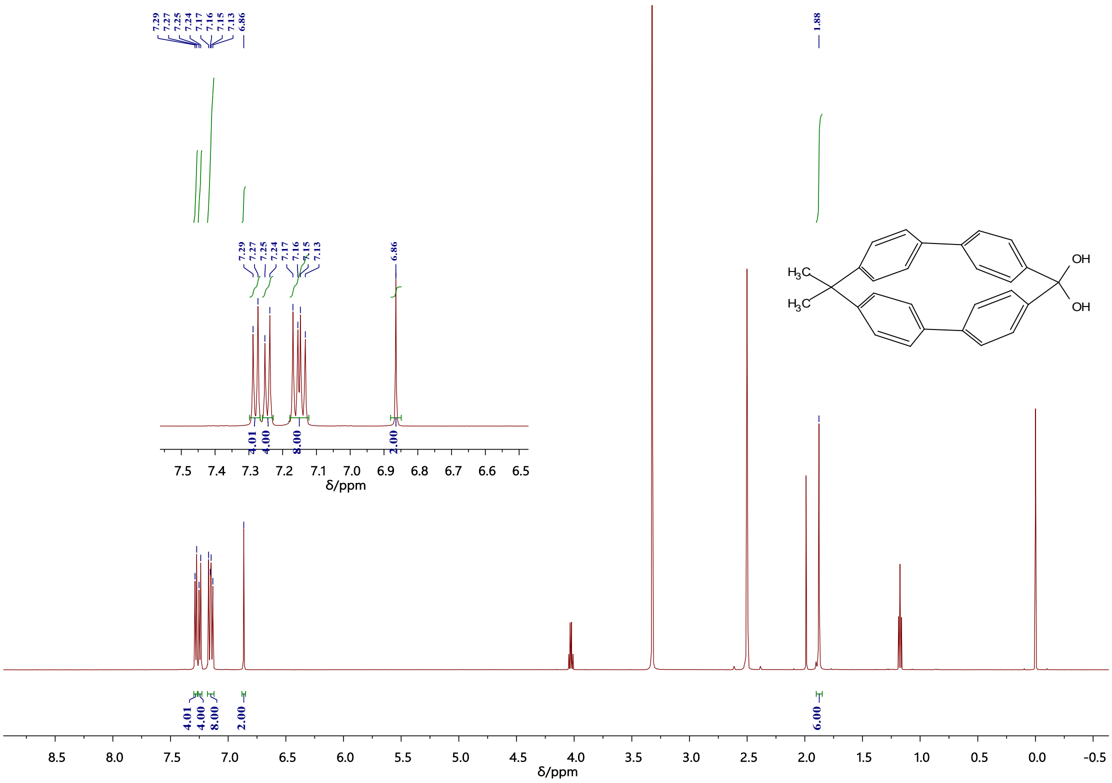

Figure S34. ^{1}H NMR spectrum of **diol-Me-[1,1][2]PCP** (600 MHz, DMSO-d_6, 298 K)

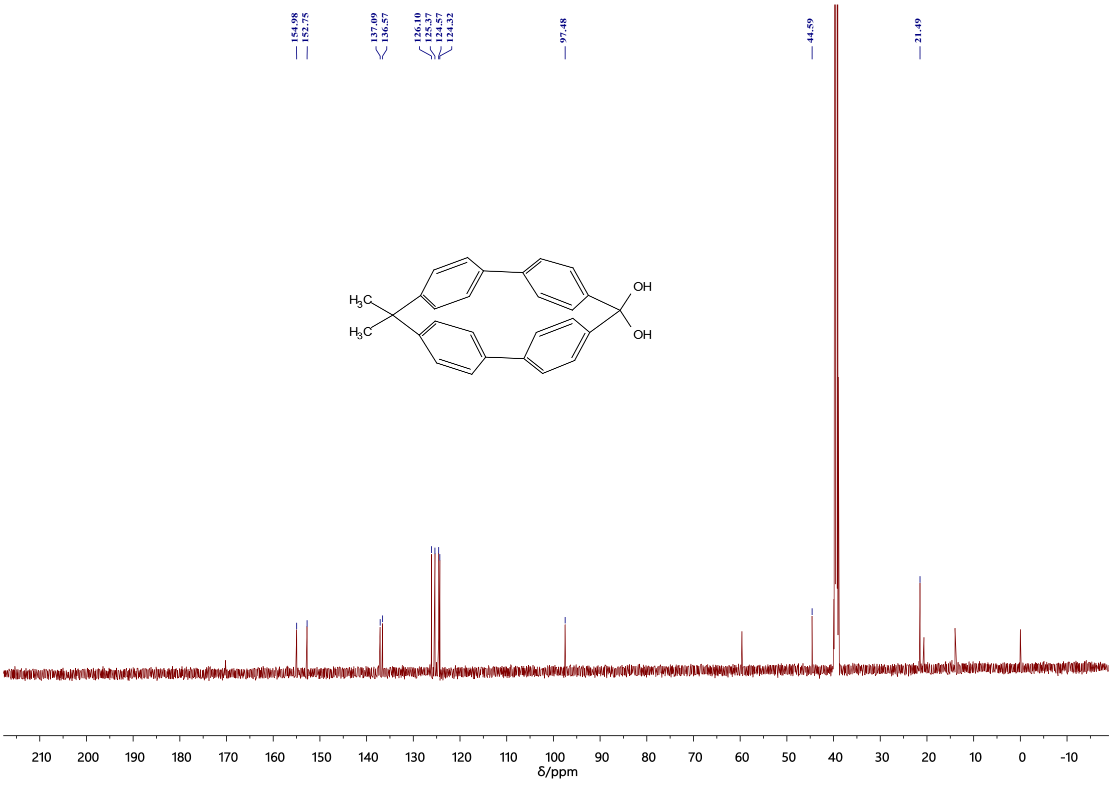

Figure S35. ^{13}C NMR spectrum of **diol-Me-[1,1][2]PCP** (600 MHz, DMSO-d_6, 298 K).

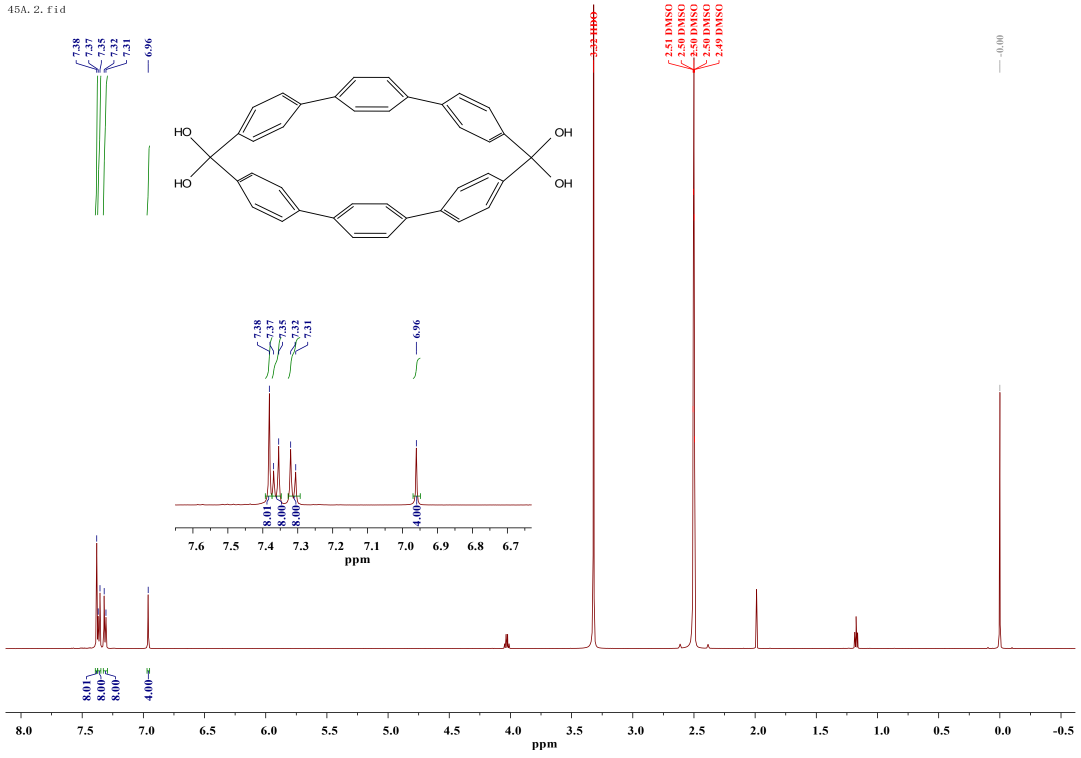

Figure S36. ^{1}H NMR spectrum of **2diols-[1,1][3]PCP** (600 MHz, DMSO-d_6, 298 K).

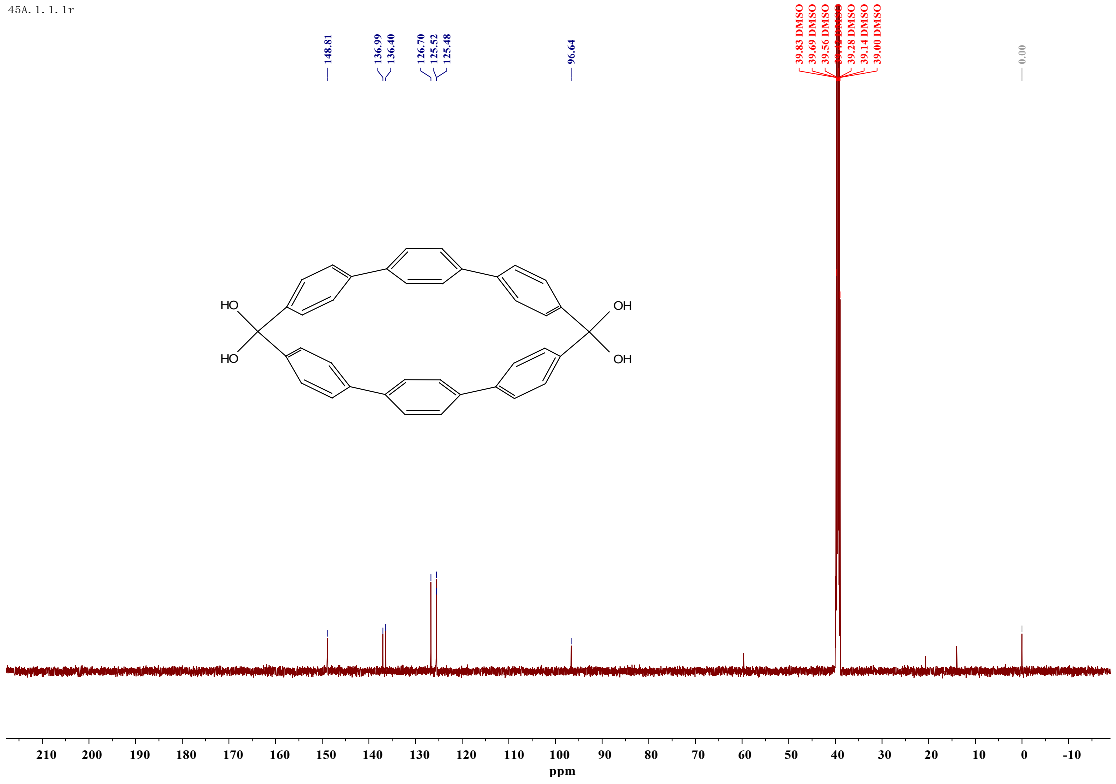

Figure S37. ^{13}C NMR spectrum of **2diols-[1,1][3]PCP** (151 MHz, DMSO-d_6, 298 K).

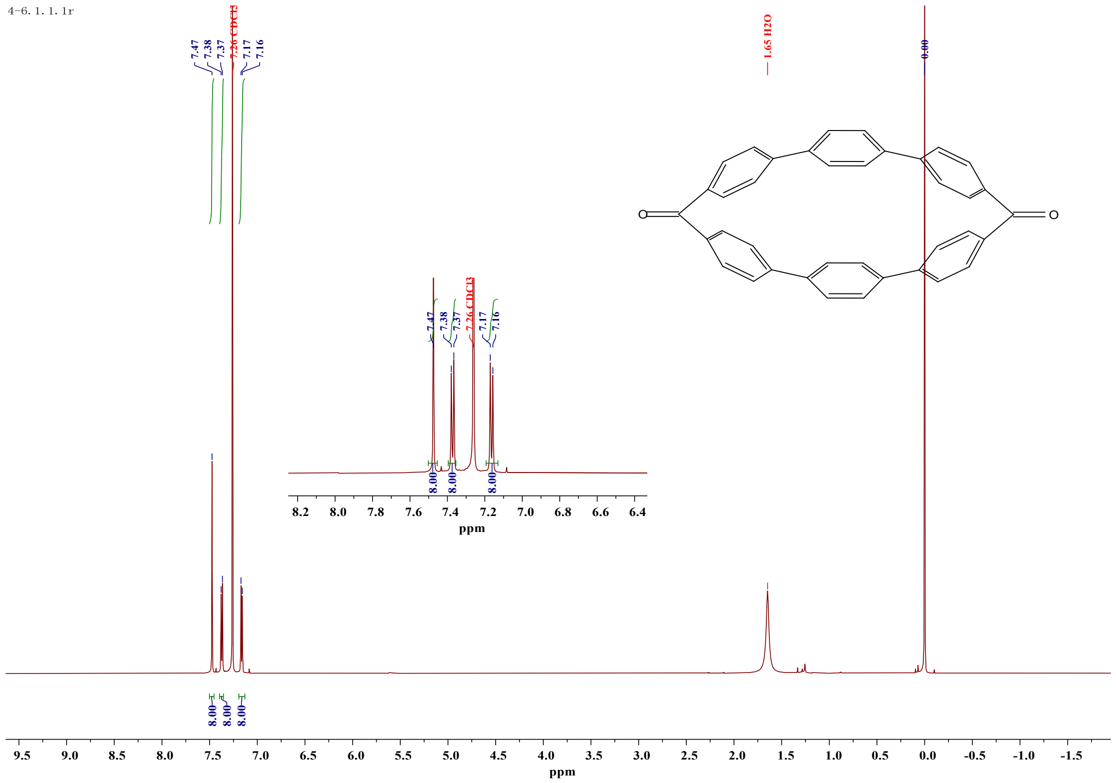

Figure S38. ^{1}H NMR spectrum of **2ketones-[1,1][3]PCP** (600 MHz, $CDCl_3$, 298 K).

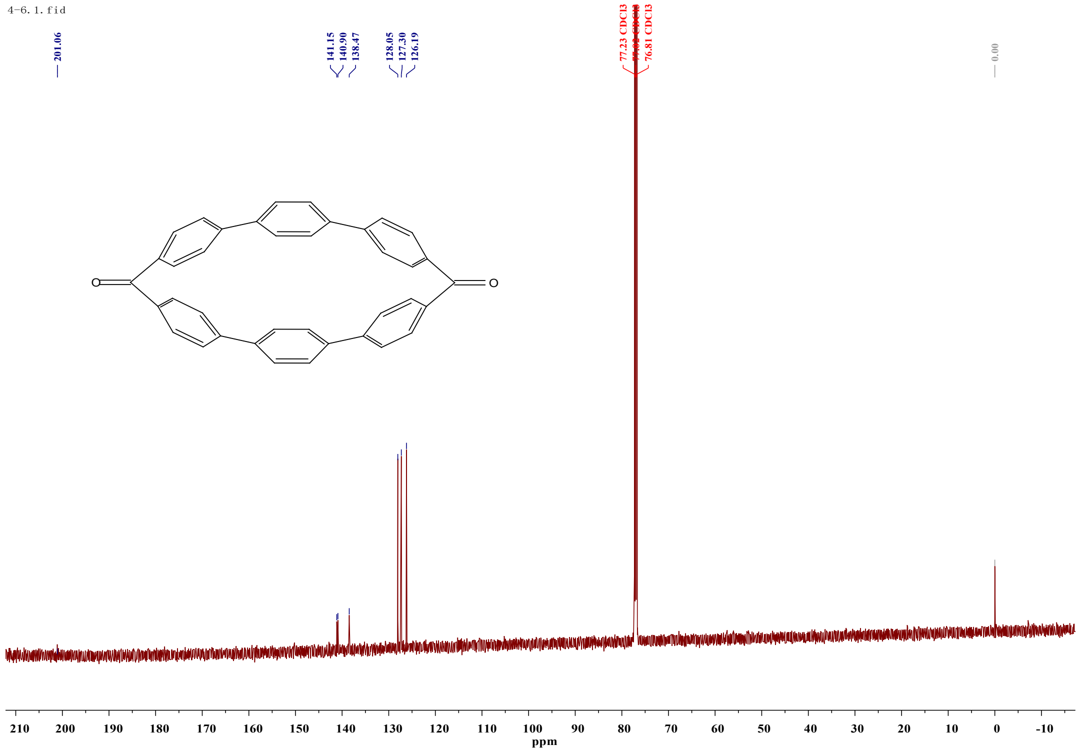

Figure S39. ^{13}C NMR spectrum of **2ketones-[1,1][3]PCP** (151 MHz, $CDCl_3$, 298 K).

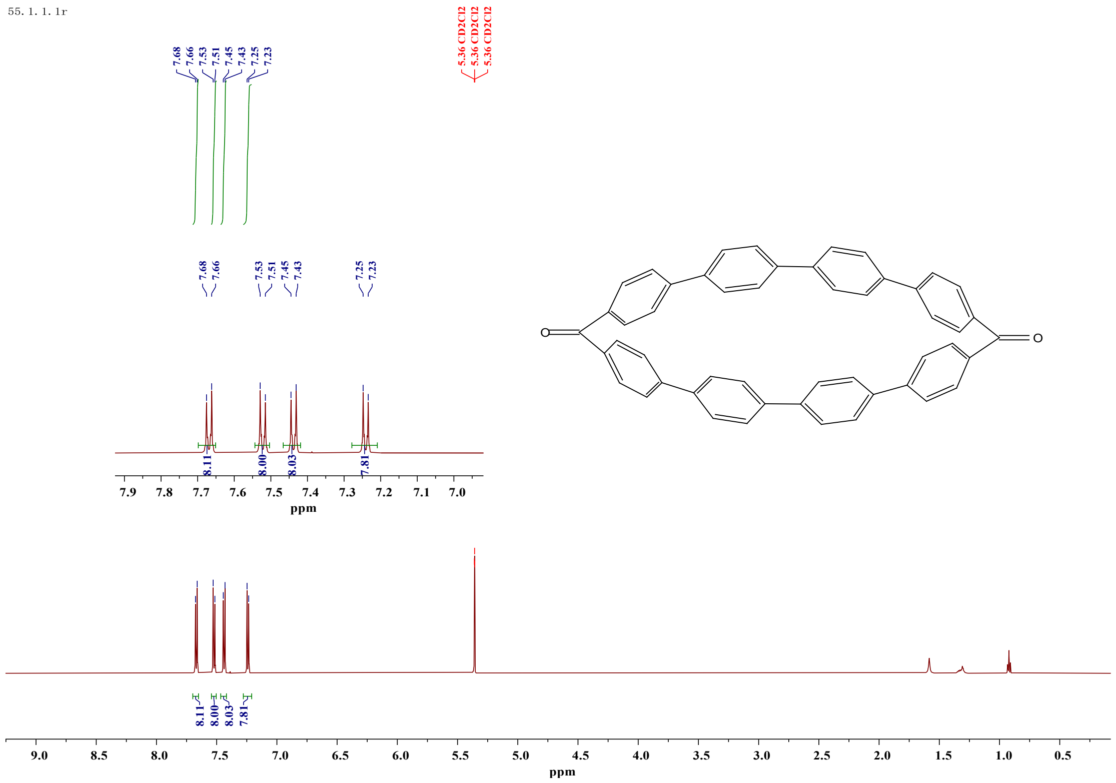

Figure S40. ^{1}H NMR spectrum of **2ketones-[1,1][4]PCP** (600 MHz, $CDCl_3$, 298 K).

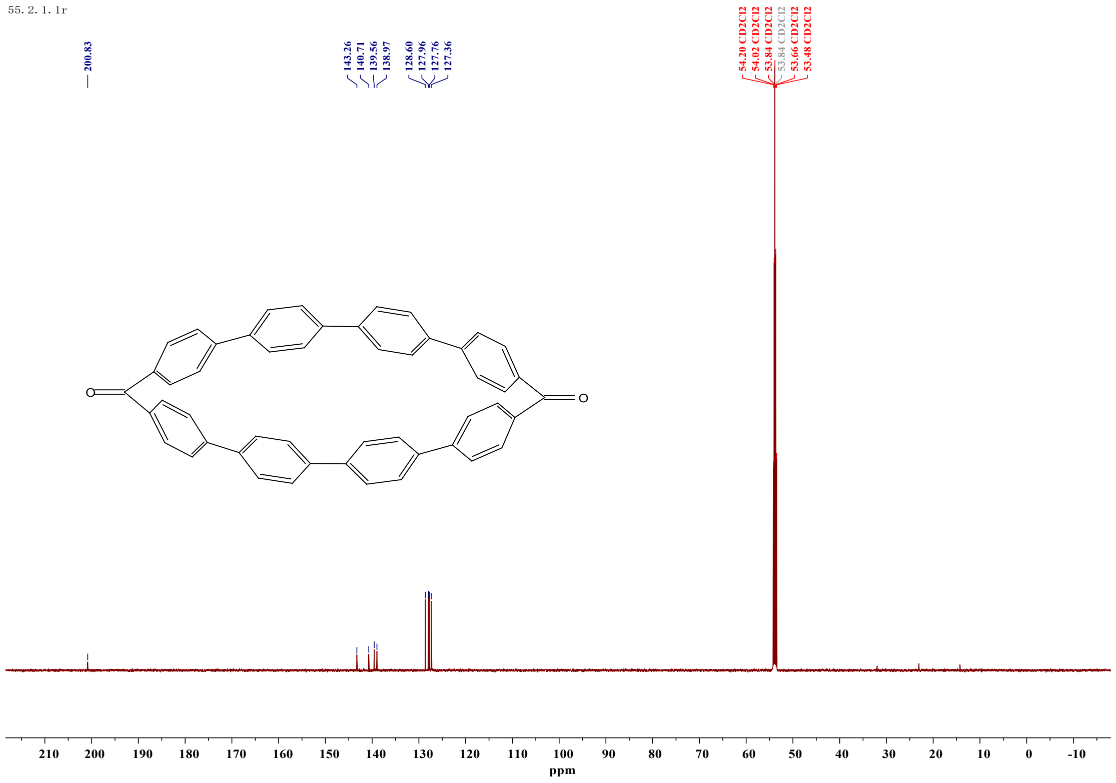

Figure S41. ^{13}C NMR spectrum of **2ketones-[1,1][4]PCP** (151 MHz, CD_2Cl_2, 298 K).

4. Mass Spectra

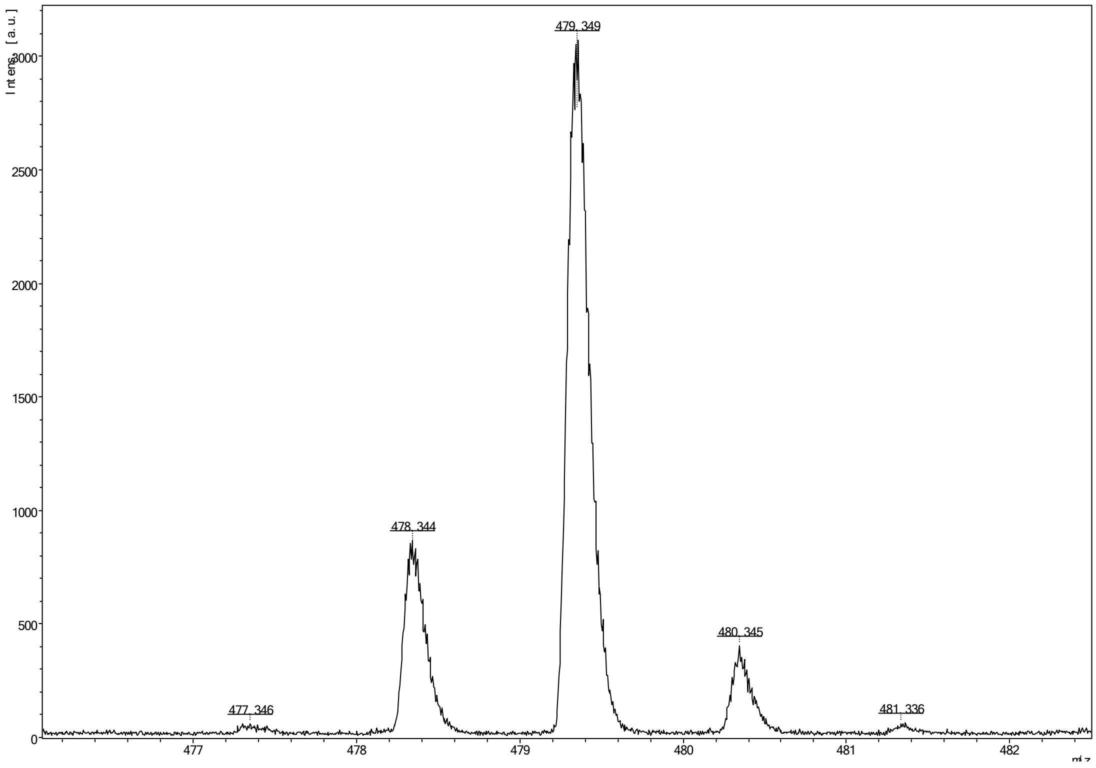

Figure S42. MALDI spectrum of compound **S2**

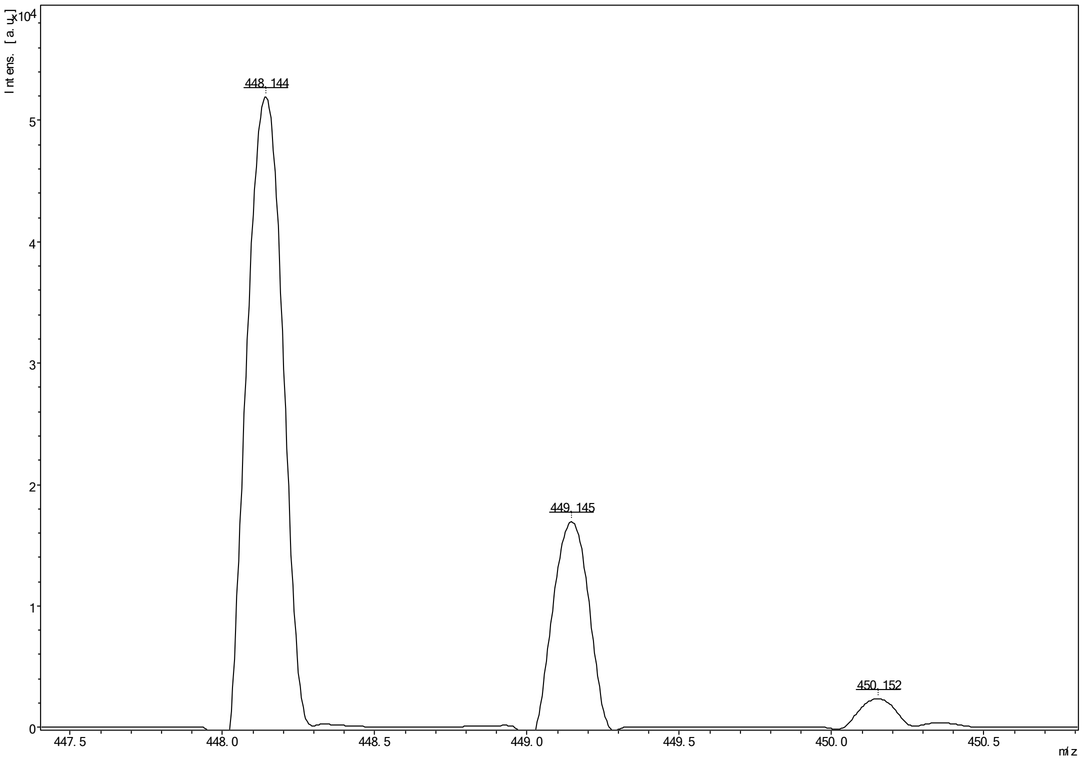

Figure S43. MALDI spectrum of compound **2EG-[1,1][2]PCP**

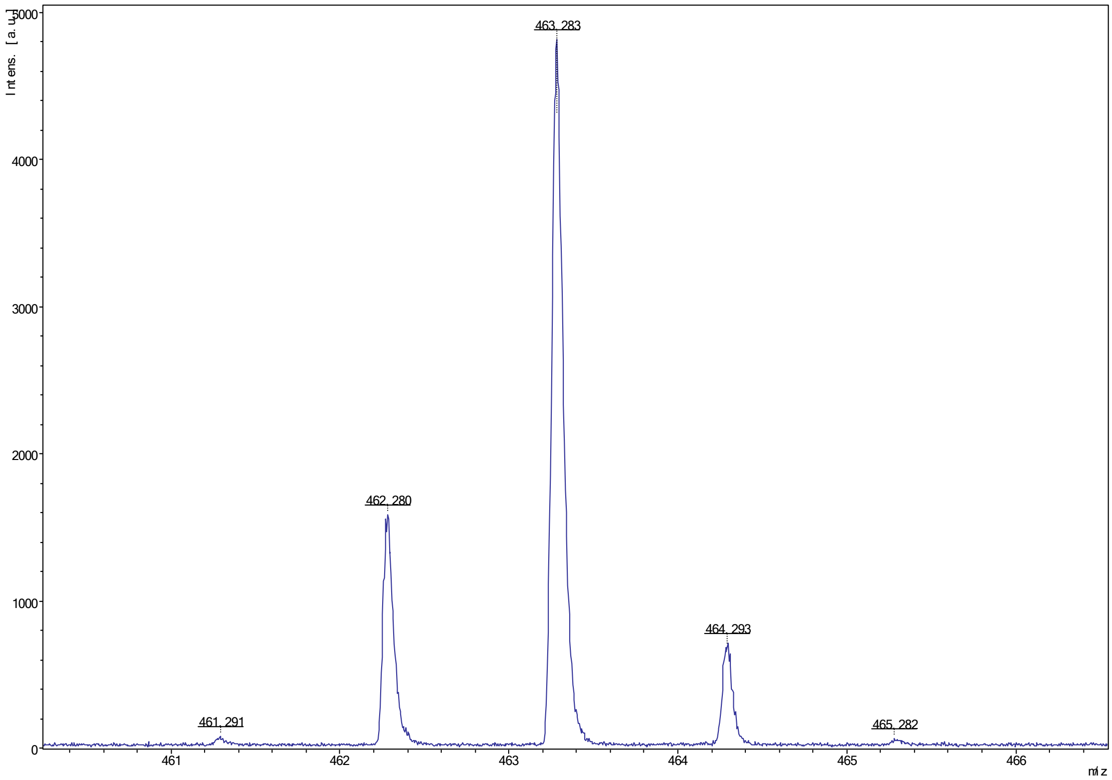

Figure S44. MALDI spectrum of compound **S4**

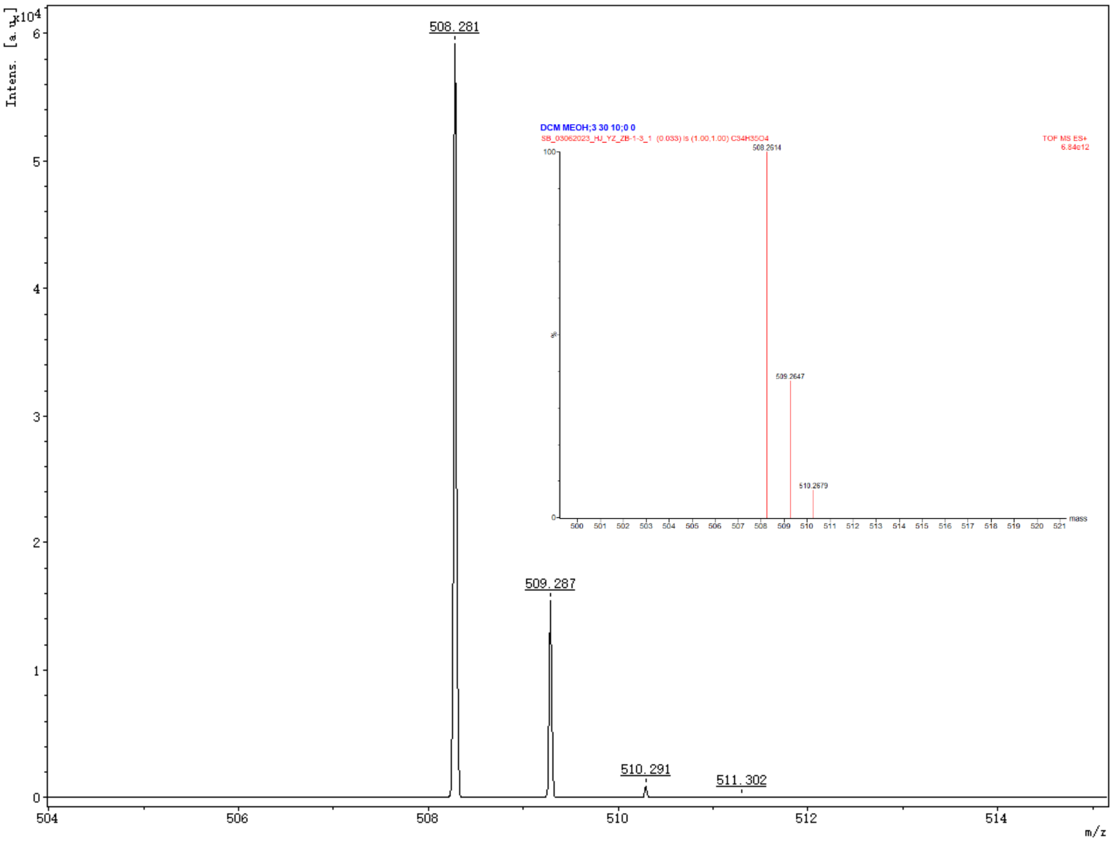

Figure S45. MALDI spectrum of compound **2ketals-[1,1][4]PCP**

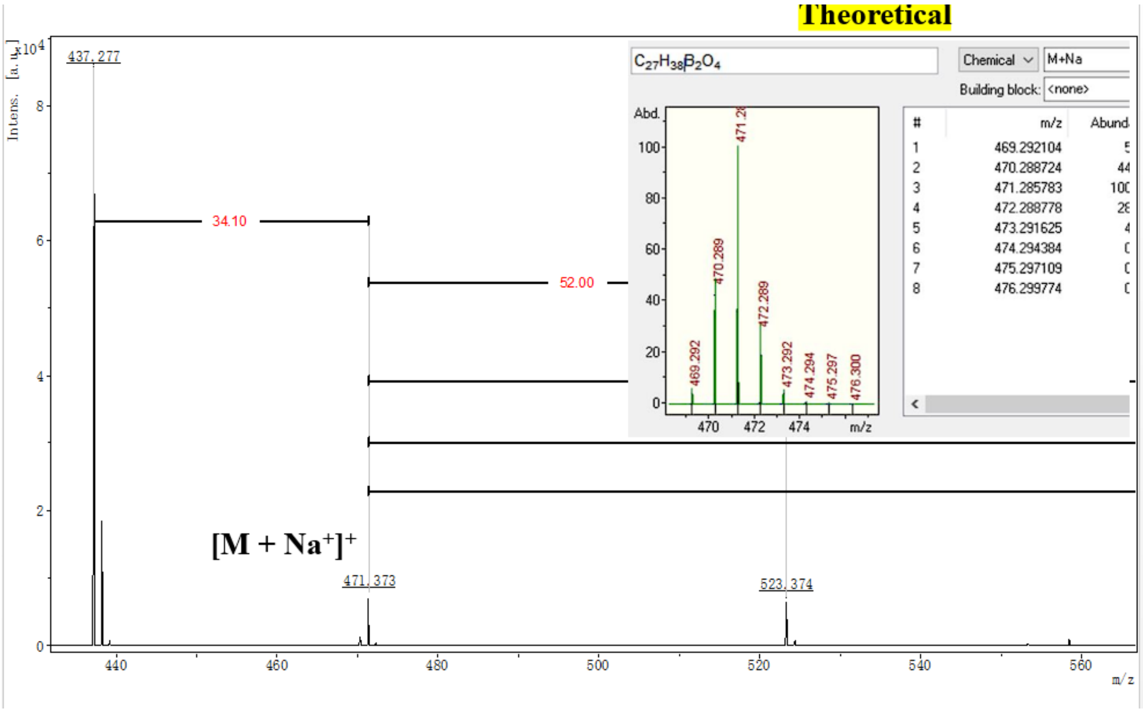

Figure S46. MALDI spectrum of compound **S6**

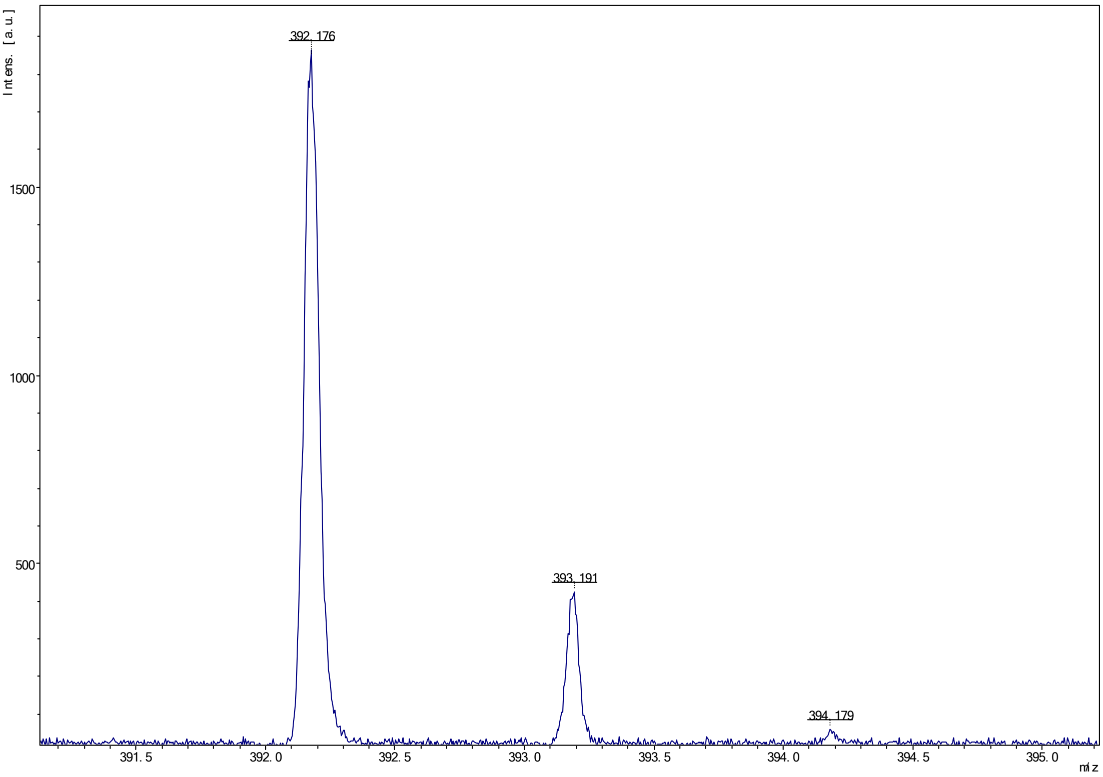

Figure S47. MALDI spectrum of compound **ketal-2Me-[1,1][2]PCP**

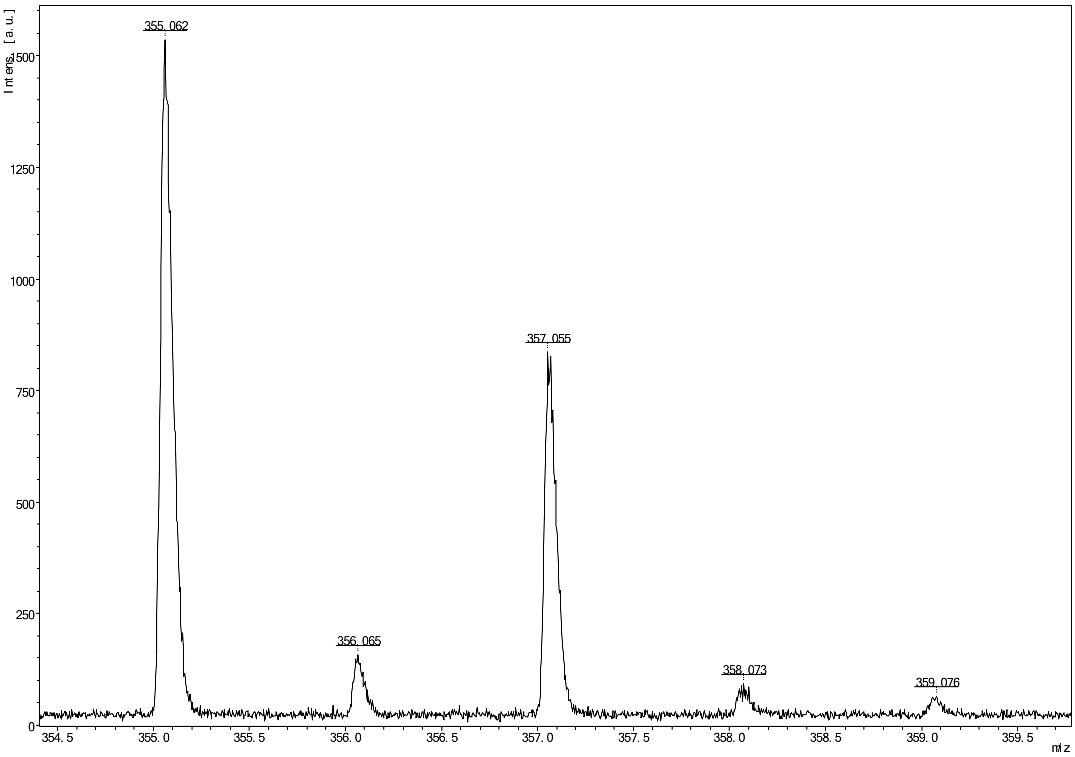

Figure S48. MALDI spectrum of compound **S8**

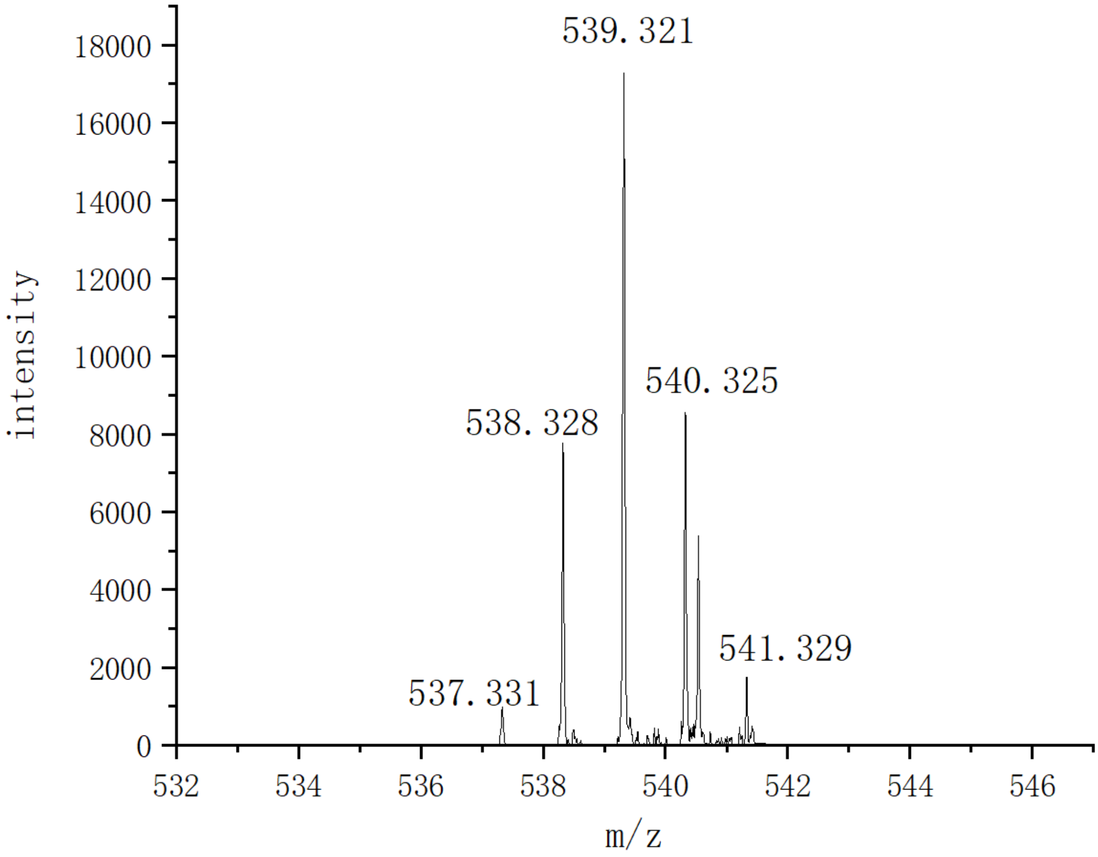

Figure S49. MALDI spectrum of compound **S9**

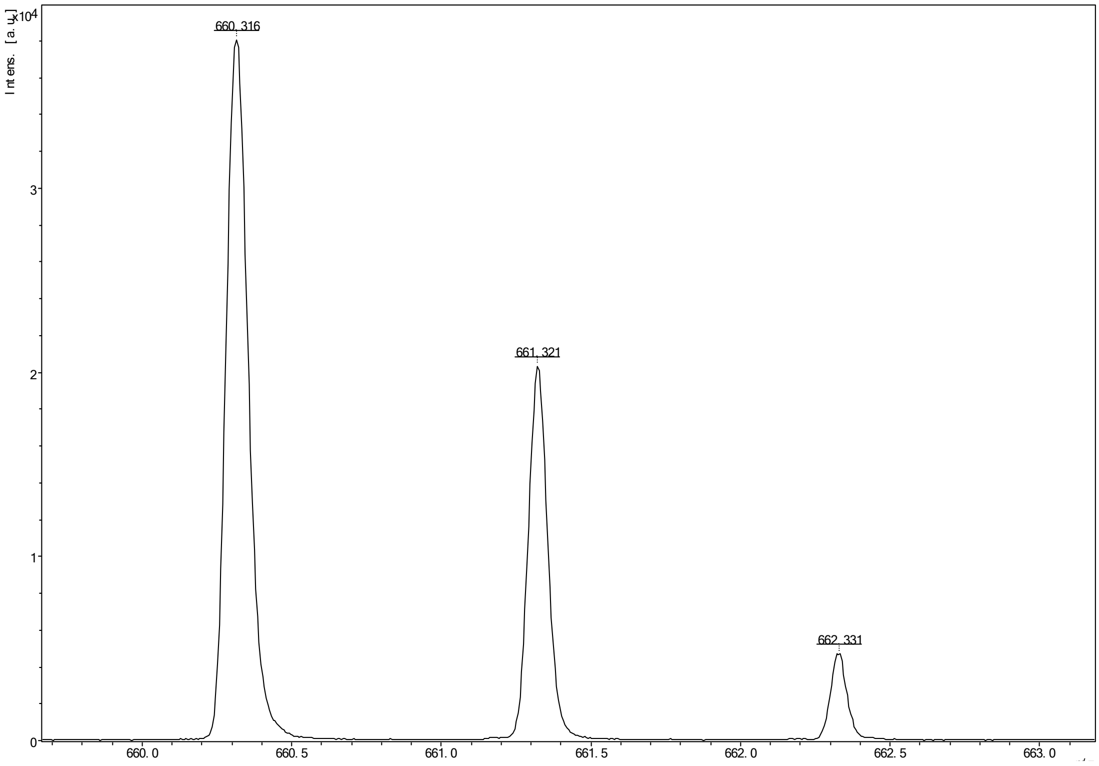

Figure S50. MALDI spectrum of compound **2ketals-[1,1][3]PCP**

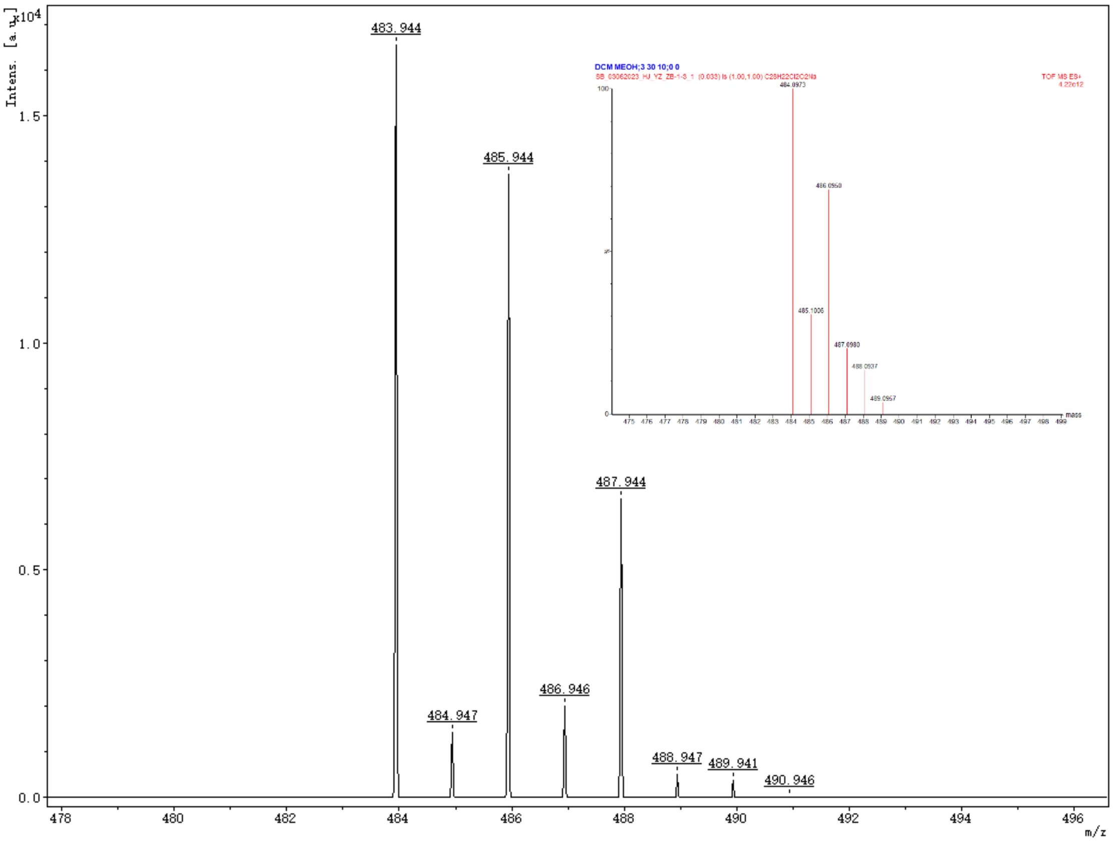

Figure S51. MALDI spectrum of compound **S10**

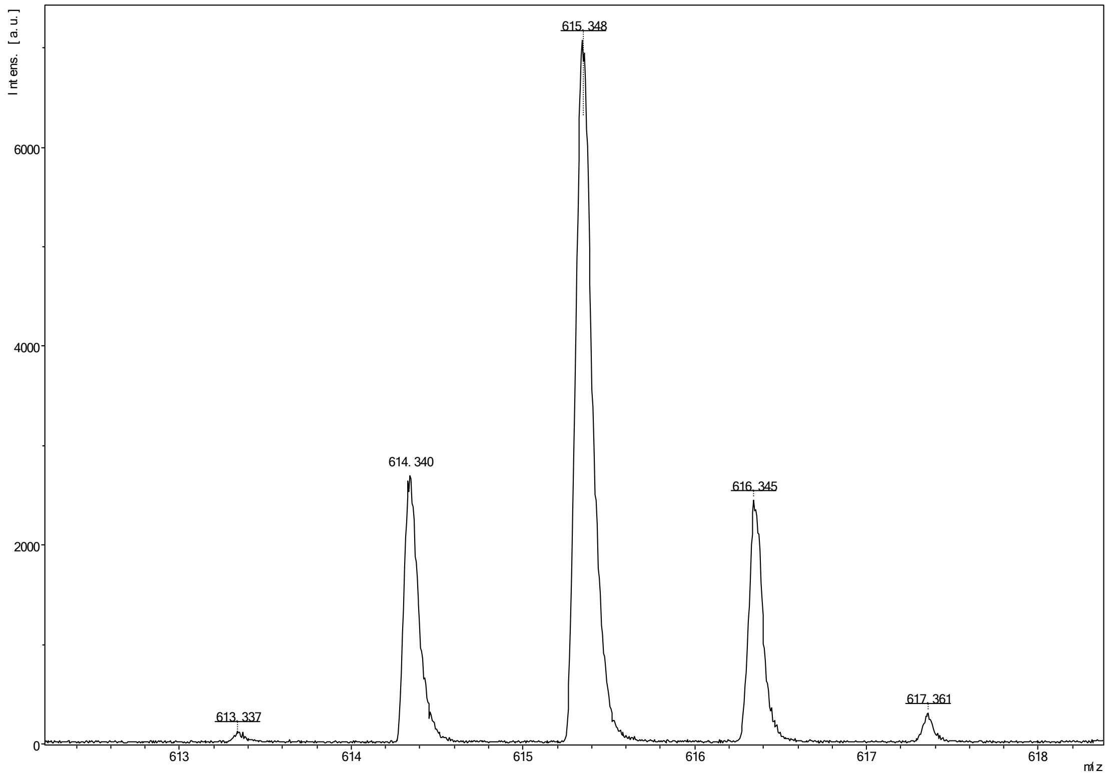

Figure S52. MALDI spectrum of compound **S11**

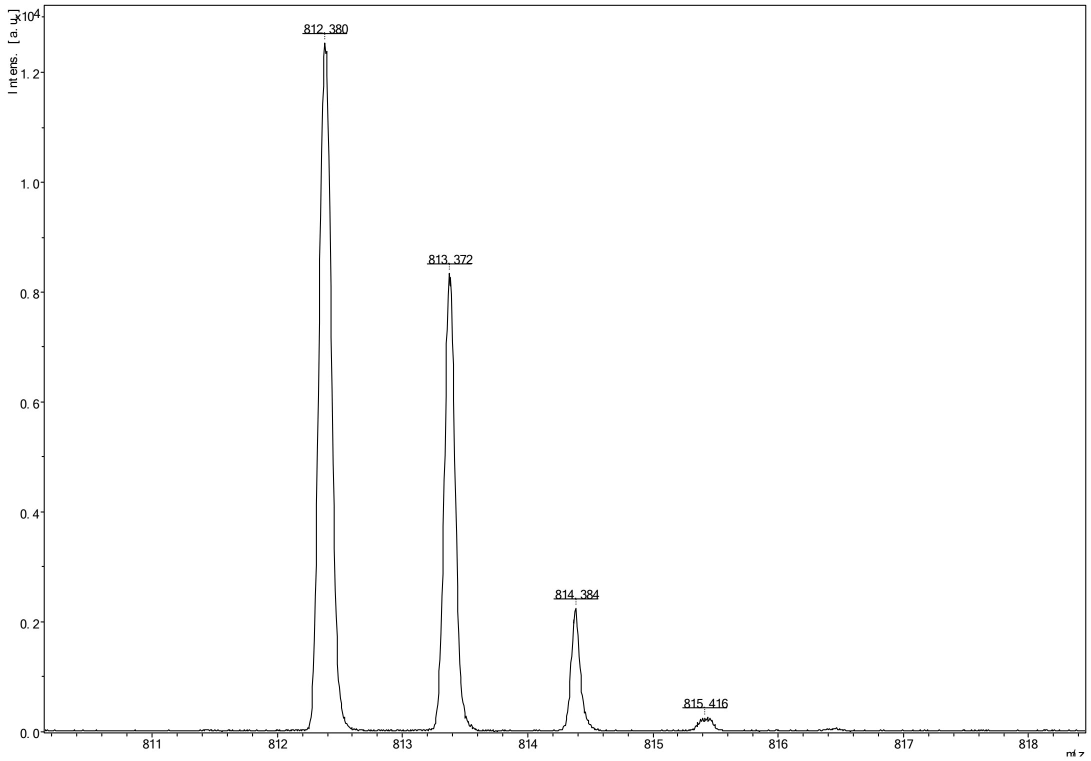

Figure S53. MALDI spectrum of compound **2ketals-[1,1][4]PCP**

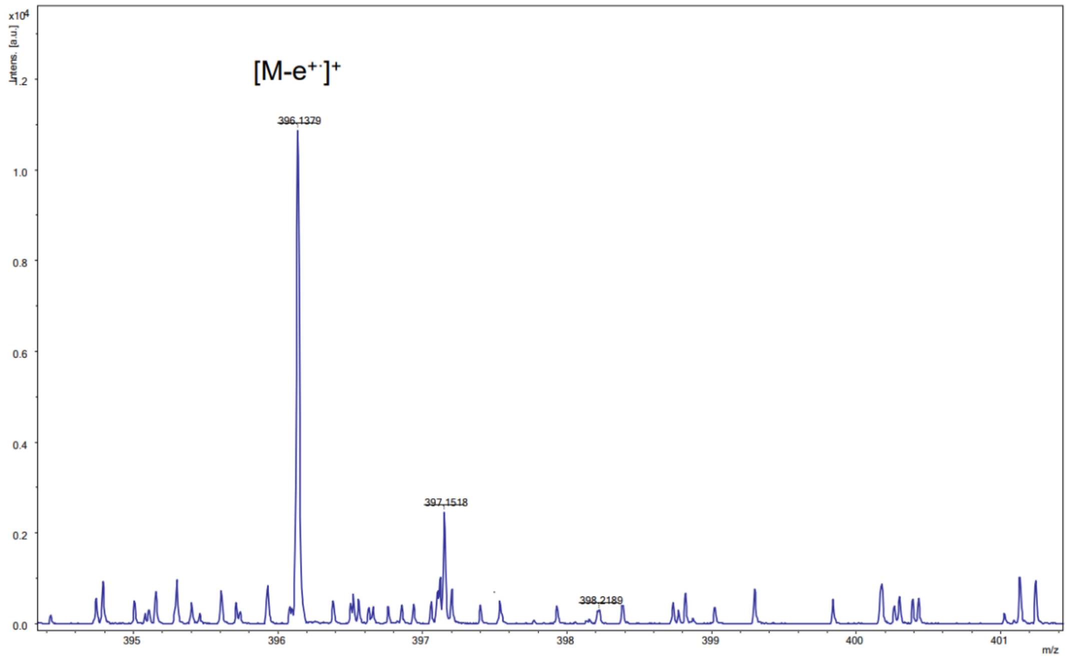

Figure S54. MALDI spectrum of compound **2diols-[1,1][2]PCP**

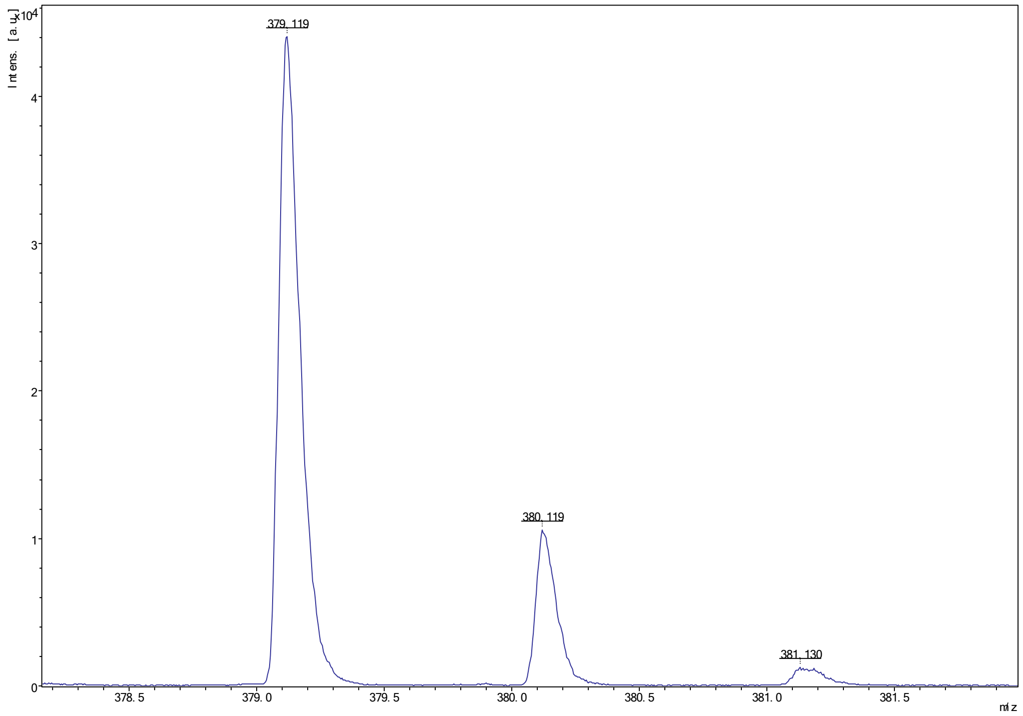

Figure S55. MALDI spectrum of compound **1**

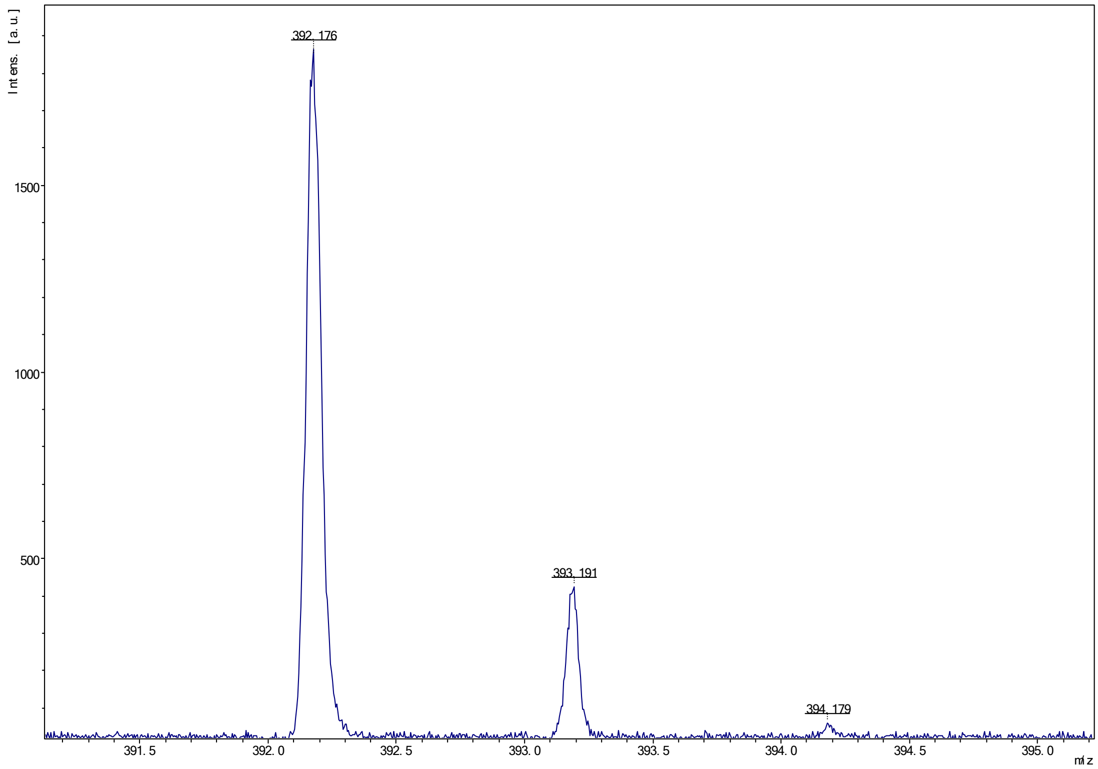

Figure S56. MALDI spectrum of compound **diols-2Me-[1,1][2]PCP**

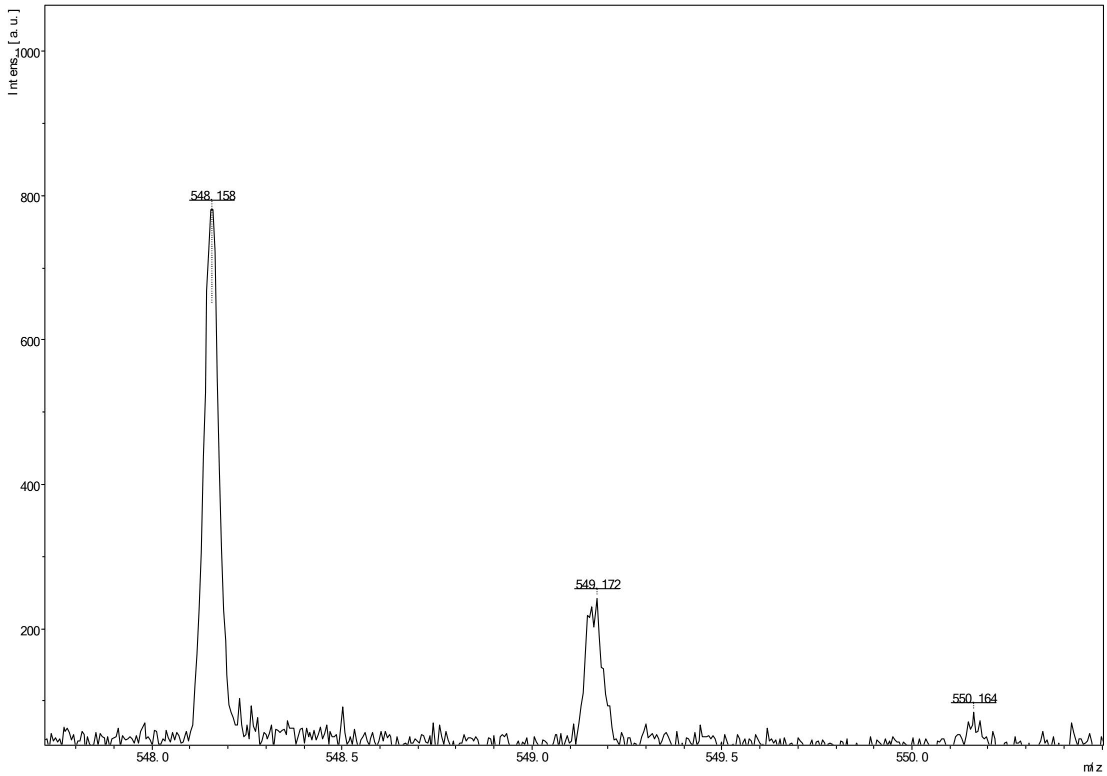

Figure S57. MALDI spectrum of compound **2diols-[1,1][3]PCP**

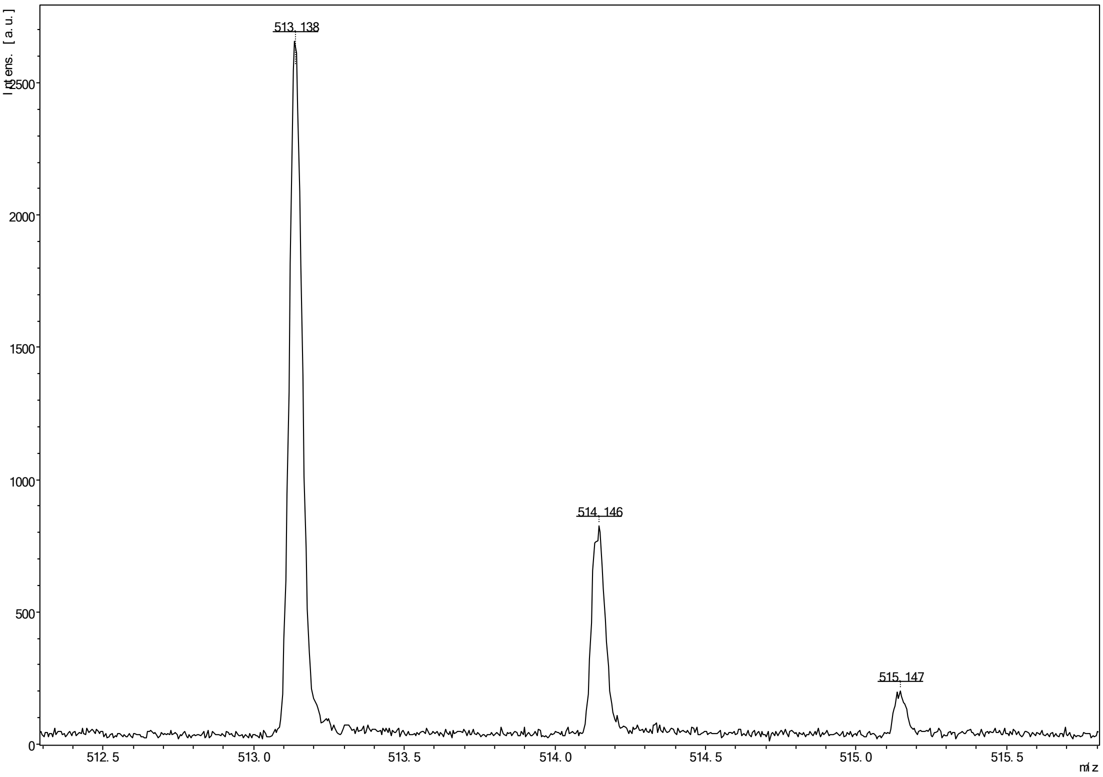

Figure S58. MALDI spectrum of compound **2ketones-[1,1][3]PCP**

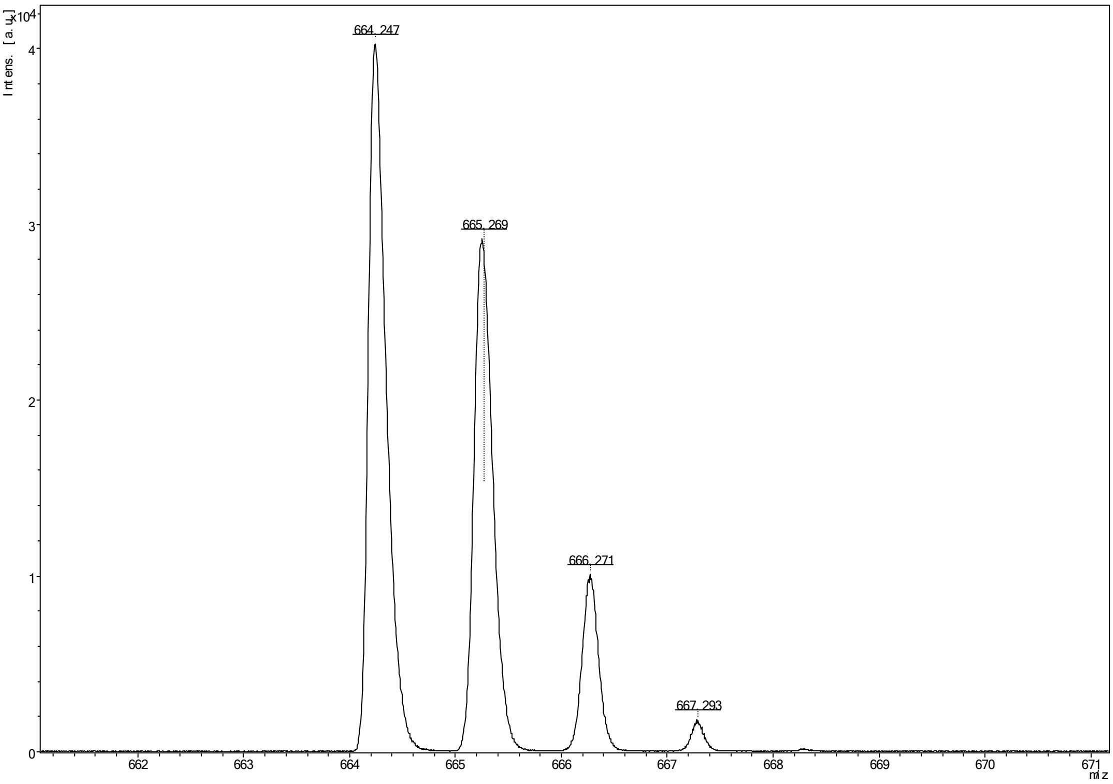

Figure S59. MALDI spectrum of compound **2ketones-[1,1][4]PCP**

5.TGA curves

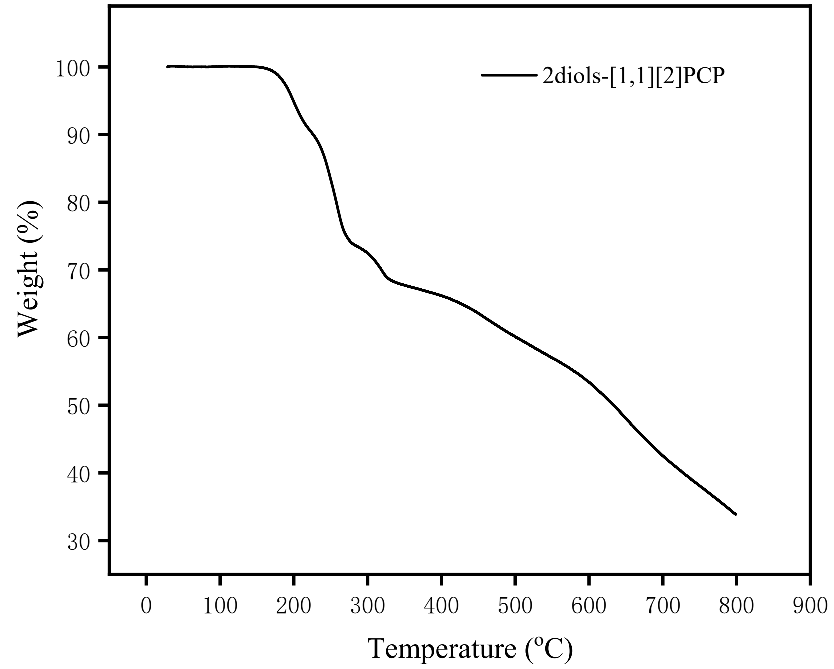

Figure S60. TGA curve of **2diols-[1,1][2]PCP.**

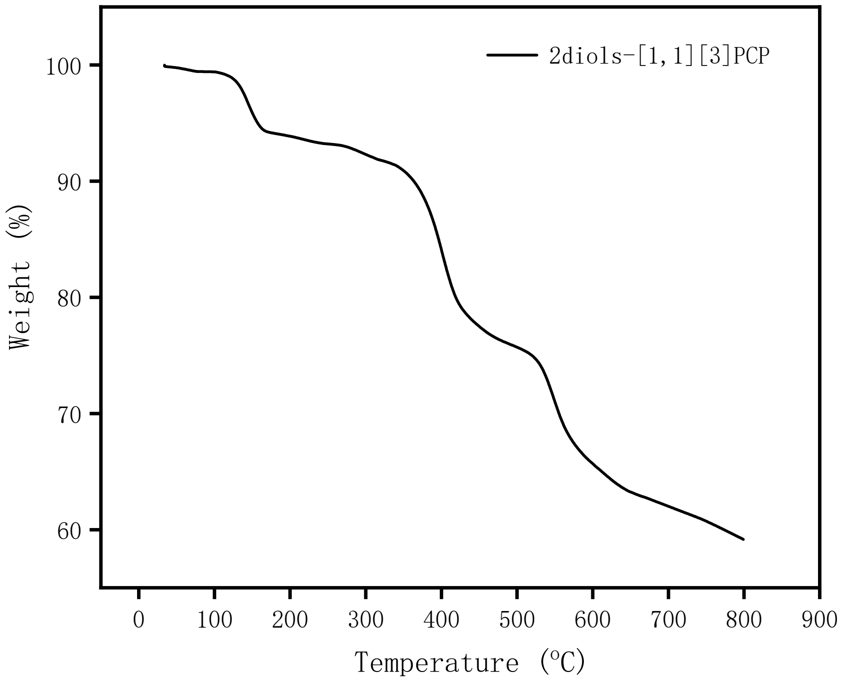

Figure S61. TGA curve of **2diols-[1,1][3]PCP**

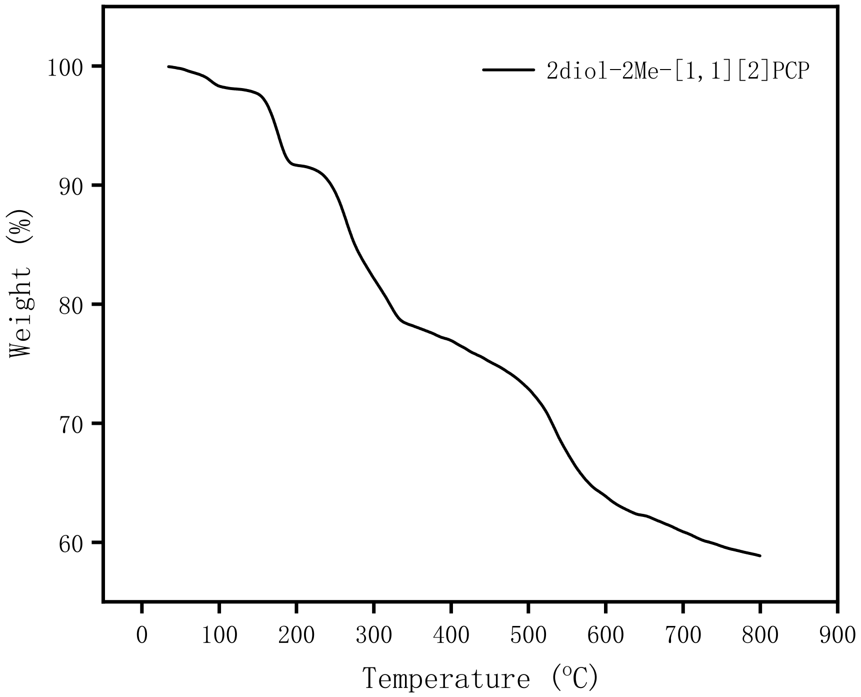

Figure S62. TGA curve of **diol-2Me-[1,1][2]PCP.**

5. X-Ray Crystallographic Analysis

Crystals suitable for single crystal X-ray diffraction analyses were obtained for compound **2dbts-[1,1][2]PCP**, **2ketals-[1,1][2]PCP**, **2diols-[1,1][2]PCP**, **ketal-2Me-[1,1][2]PCP**, **diol-2Me-[1,1][2]PCP**, **2ketals-[1,1][3]PCP**, **2diols-[1,1][3]PCP**, **2ketals-[1,1][4]PCP and 2ketones-[1,1][4]PCP**. Preliminary data on the space group and unit cell dimensions as well as intensity data were collected on a Bruker D8 Venture Diffraction with CCD plate detector under a flow of nitrogen gas, **2dbts-[1,1][2]PCP** using Mo Kα radiation (λ = 0.71073 Å) at 293(2) K, **2ketals-[1,1][2]PCP** using Mo Kα radiation (λ = 0.71073 Å) at 218 K, **2diols-[1,1][2]PCP** using Cu Kα radiation (λ = 1.54178) at 99.99(10) K, **ketal-2Me-[1,1][2]PCP** using Mo Kα radiation (λ = 0.71073 Å) at 298.15 K, **diol-2Me-[1,1][2]PCP** using Ga Kα radiation (λ = 1.34139 Å) at 150.00 K, **2ketals-[1,1][3]PCP** using Mo Kα radiation (λ = 0.71073 Å) at 298.15 K, **2diols-[1,1][3]PCP** using Ga Kα radiation (λ = 1.34139 Å) at 260.00 K, **2ketals-[1,1][4]PCP** using Ga Kα radiation (λ = 1.34139 Å) at 163.00 K, **2ketones-[1,1][4]PCP** using Mo Kα radiation (λ = 0.71073 Å) at 298.15 K, Reflection intensities were corrected for absorption by the multi-scan method. The structure was solved by direct methods using intrinsic phasing implemented in SHELXT (Sheldrick, 2015) with the Olex2[4]. The model was refined applying the full-matrix least-squares method using SHELXL (Sheldrick,

2018). All non-hydrogen atoms were refined with anisotropic displacement parameters. Hydrogen atoms were placed at calculated positions and refined using a riding model. Details of the crystal data and a summary of the intensity data collection parameters are listed in **Table S1-S9**.

Table S1. Selected single-crystal data of macrocycles.

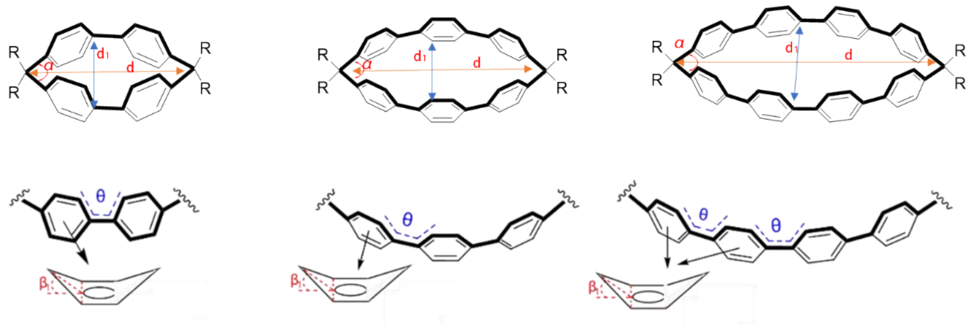

compound	d	d1	β	θ	α
2MEGs-[1,1][2]PCP	8.493	4.801	10.28	5.68	102.29
Ketal-Me-[1,1][2]PCP	8.644	4.649	9.83	23.06	99.85
2ketals-[1,1][2]PCP	8.613	4.617	8.61	26.54	100.97
2ketals-[1,1][3]PCP	12.303	6.330	6.52	27.15	103.44
2ketals-[1,1][4]PCP	15.834	8.755	5.47	34.12	106.01
Me-diol-[1,1][2]PCP	8.644	4.687	10.33	7.08	101.44/98.65
2diols-[1,1][2]PCP	8.573	4.675	9.65	30.06	101.10
2diols-[1,1][3]PCP	12.20	6.60	6.61	26.54	103.01
2ketones-[1,1][4]PCP	14.58	10.24	8.46	25.63	112.92

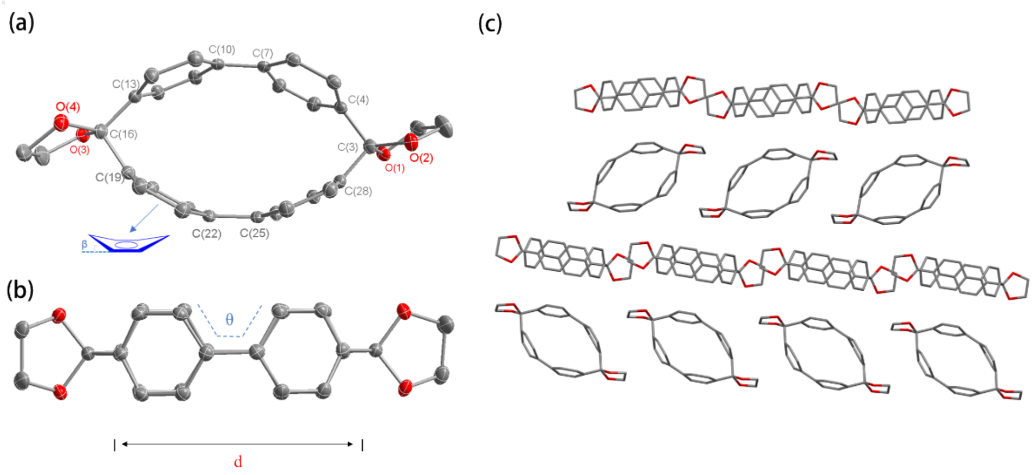

Figure S63. X-ray single-crystal structure of **2dbts-[1,1][2]PCP**: (a) top view, (b) side view, and (c) crystal packing diagram.

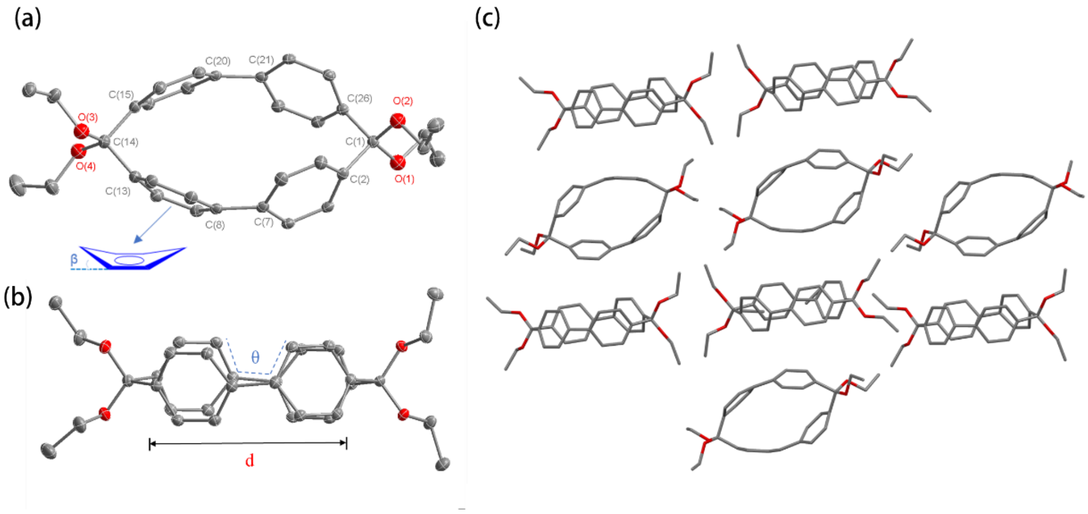

Figure S64. X-ray single-crystal structure of **2ketals-[1,1][2]PCP**: (a) top view, (b) side view, and (c) molecular packing.

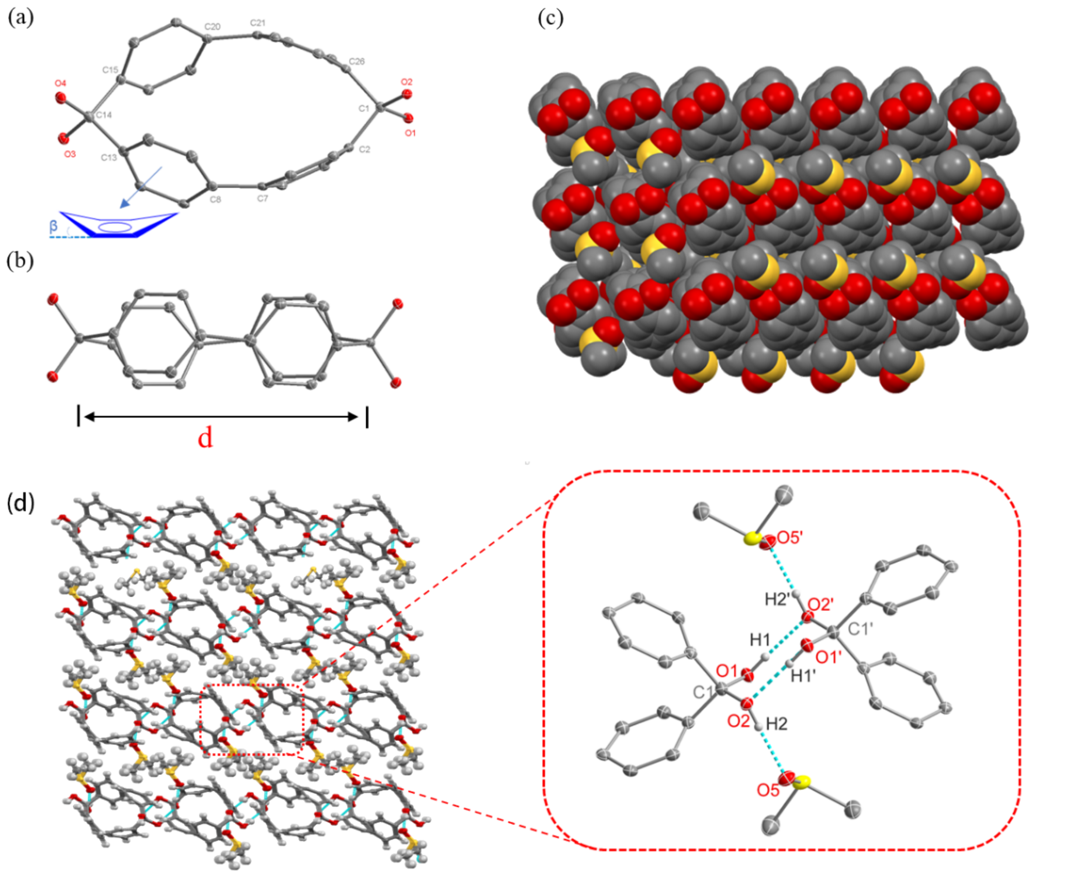

Figure S65. X-ray single-crystal structure of **2diols-[1,1][2]PCP**: (a) top view, (b) side view, (c) molecular packing, and (d) Weak intermolecular interactions in the crystal structure, highlighting O–H···O hydrogen bonds .

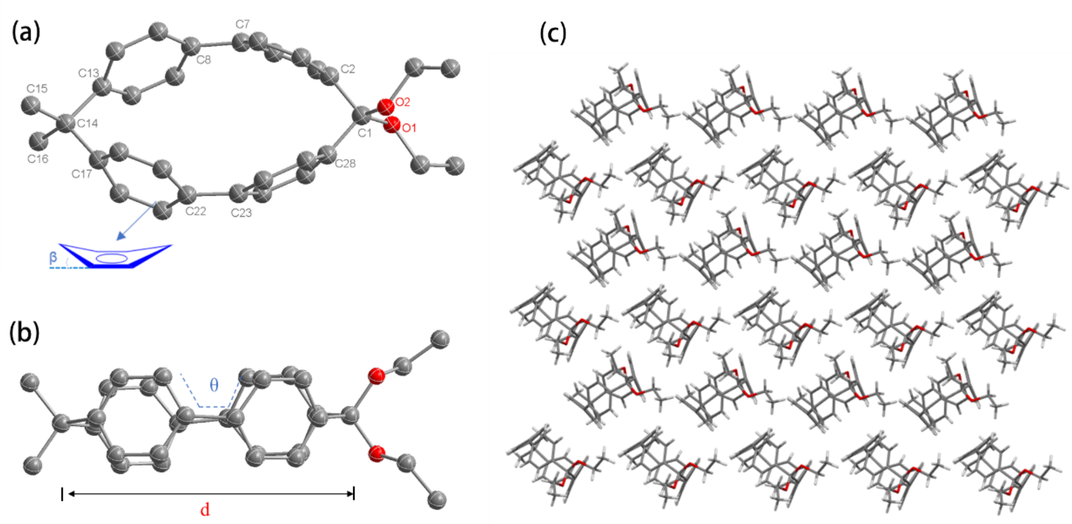

Figure S66. X-ray single-crystal structure of **ketal-2Me-[1,1][2]PCP**: (a) top view, (b) side view, and (c) molecular packing.

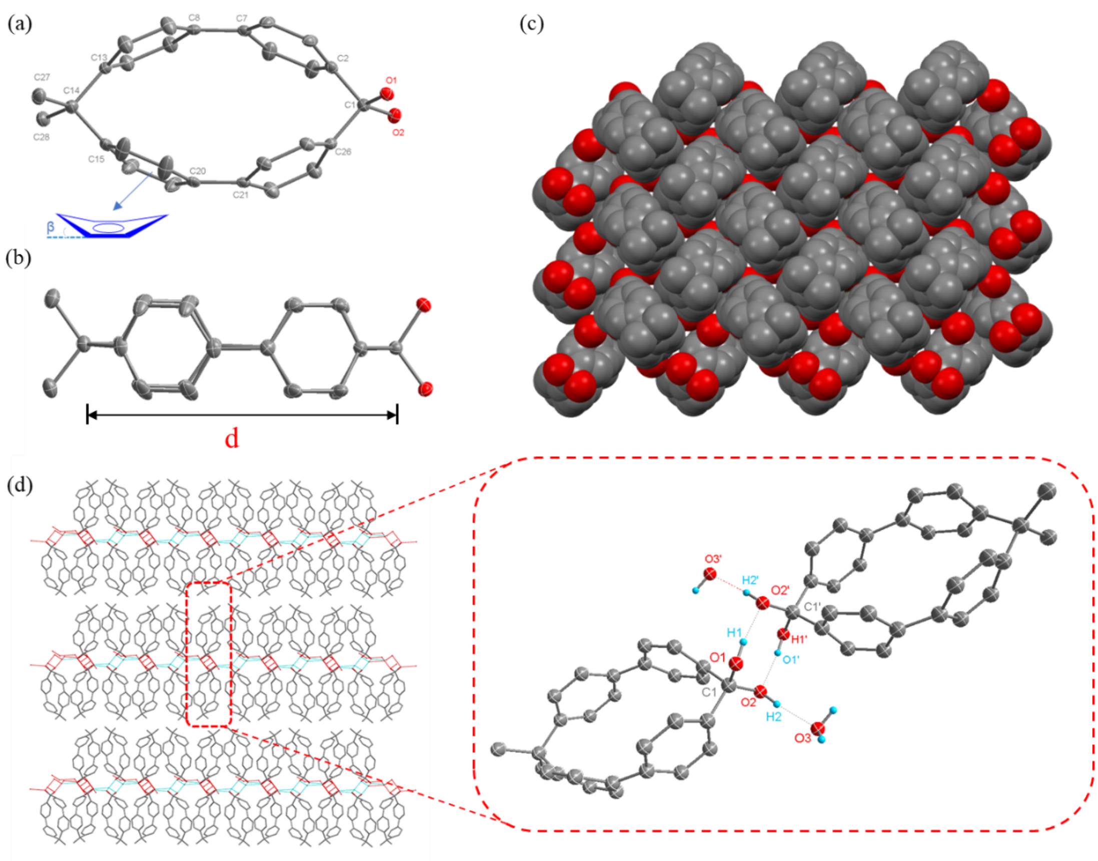

Figure S67. X-ray single-crystal structure of **diol-2Me-[1,1][2]PCP**: (a) top view, (b) side view, (c) molecular packing, and (d) Weak intermolecular interactions in the crystal structure, highlighting O–H···O hydrogen bonds .

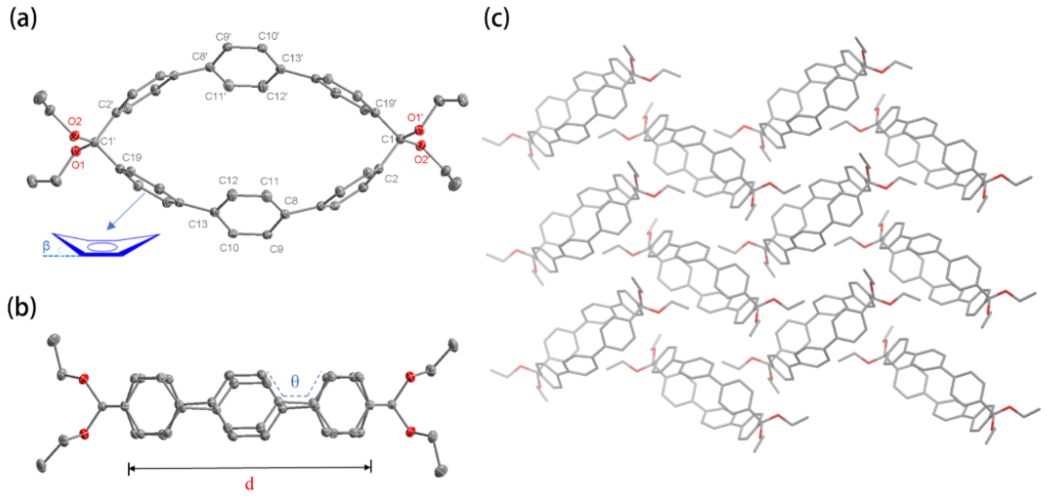

Figure S68. X-ray single-crystal structure of **2ketals-[1,1][3]PCP**: (a) top view, (b) side view, and (c) molecular packing.

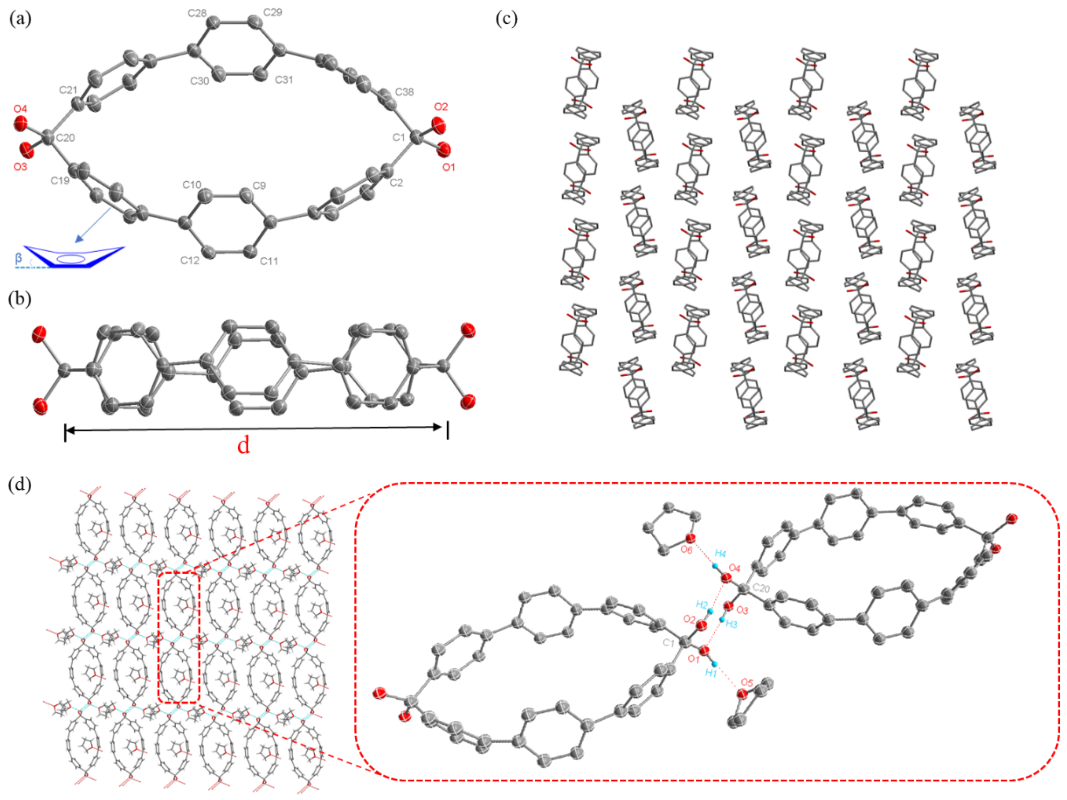

Figure S69. X-ray single-crystal structure of **2diols-[1,1][3]PCP**: (a) top view, (b) side view, (c) molecular packing, and (d) Weak intermolecular interactions in the crystal structure, highlighting O–H···O hydrogen bonds .

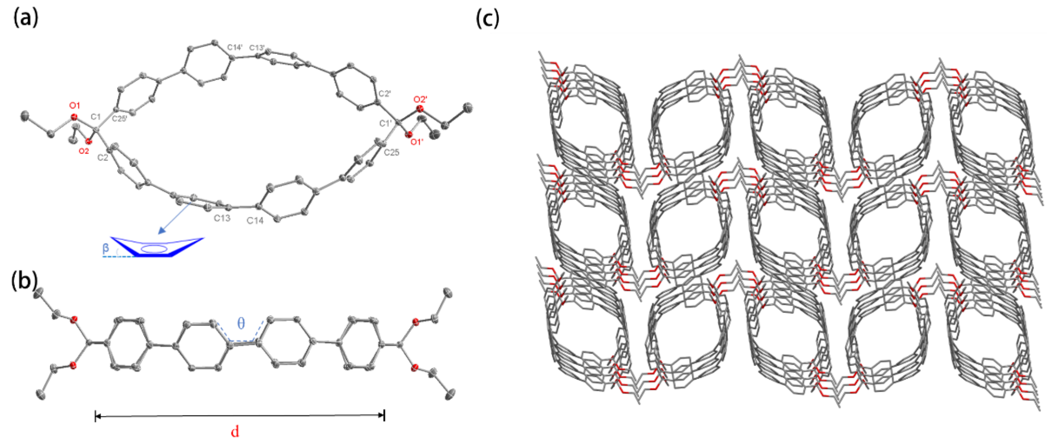

Figure S70. X-ray single-crystal structure of **2ketals-[1,1][4]PCP**: (a) top view, (b) side view, and (c) molecular packing.

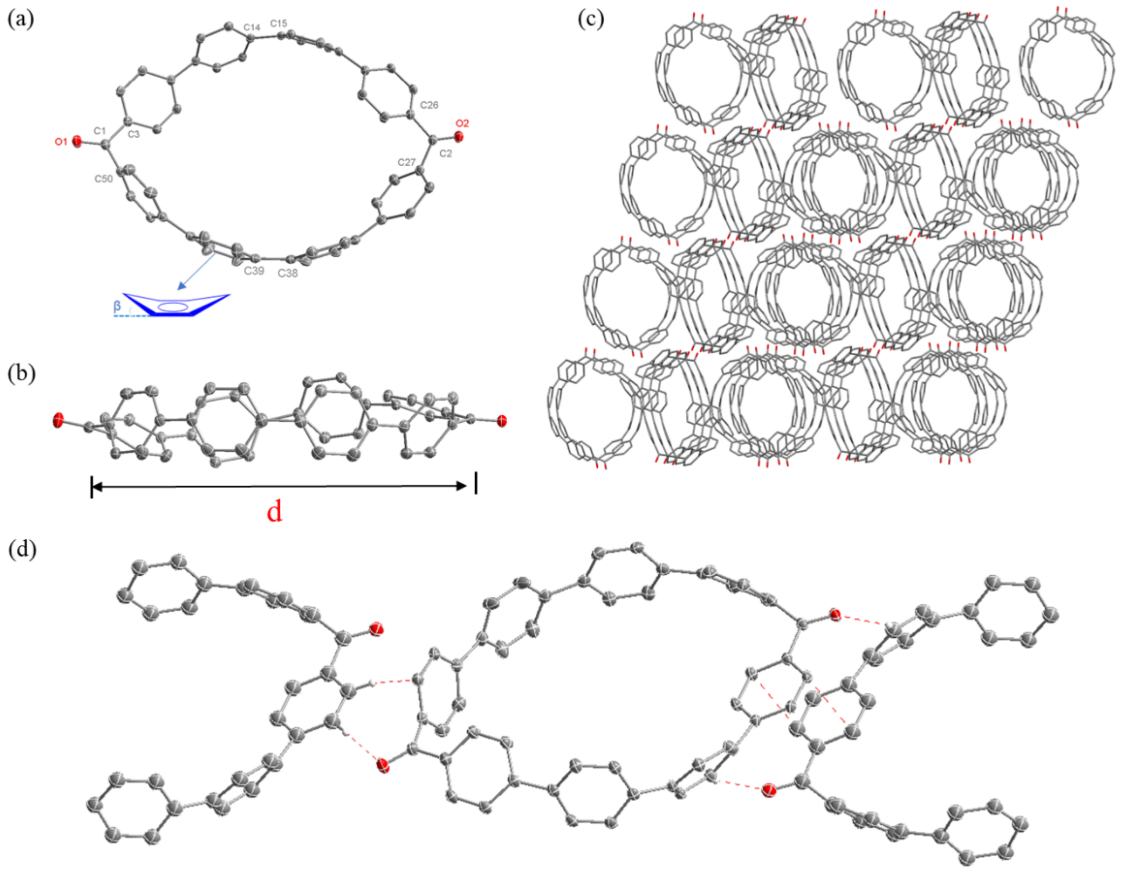

Figure S71. X-ray single-crystal structure of **2ketones-[1,1][4]PCP**: (a) top view, (b) side view, (c) molecular packing, and (d) Weak intermolecular interactions in the crystal structure.

Table S2. Crystallographic data and structure refinement for **2dbts-[1,1][2]PCP**

CCDC	2479728
Empirical formula	$C_{30} H_{24} O_4$
Formula weight	448.49
Temperature	293(2) K
Wavelength	0.71073 Å
Crystal system	Orthorhombic
Space group	P c a 21
	a = 15.7797(4) Å
Unit cell dimensions	b = 11.4128(3) Å
	c = 12.1013(2) Å
Volume	2179.33(9) $Å^3$
Z	4
Density (calculated)	1.367 Mg/m^3
Absorption coefficient	0.090 mm^{-1}
F(000)	944
Crystal size	0.170 x 0.140 x 0.120 mm^3
Theta range for data collection	1.784 to 25.999°.
Index ranges	-19<=h<=19, -14<=k<=14, -14<=l<=14
Reflections collected	18843
Independent reflections	4249 [R(int) = 0.0323]
Completeness to theta = 25.242°	99.3 %
Absorption correction	Semi-empirical from equivalents
Max. and min. transmission	0.7456 and 0.6908
Refinement method	Full-matrix least-squares on F^2
Data / restraints / parameters	4249 / 1 / 307
Goodness-of-fit on F^2	1.044
Final R indices [I>2sigma(I)]	R1 = 0.0372, wR2 = 0.0944
R indices (all data)	R1 = 0.0420, wR2 = 0.0990
Absolute structure parameter	0.4(4)
Extinction coefficient	n/a
Largest diff. peak and hole	0.209 and -0.146 $e.Å^{-3}$

Table S3. Crystallographic data and structure refinement for **2ketals-[1,1][2]PCP**

CCDC	2479743
Empirical formula	$C_{34}H_{36}O_4$
Formula weight	508.63
Temperature/K	218.00
Crystal system	monoclinic
Space group	P21/c
a/Å	25.4477(16)
b/Å	11.3272(7)
c/Å	9.7211(6)
α/°	90
β/°	98.216(2)
γ/°	90
Volume/Å3	2773.4(3)
Z	4

ρcalcg/cm3	1.218
μ/mm-1	0.078
F(000)	1088.0
Crystal size/mm3	0.12 × 0.12 × 0.09
Radiation	Mo Kα (λ = 0.71073)
2Θ range for data collection/°	3.234 to 54.968
Index ranges	-30 ≤ h ≤ 33, -14 ≤ k ≤ 12, -12 ≤ l ≤ 12
Reflections collected	29296
Independent reflections	6099 [Rint = 0.0502, Rsigma = 0.0439]
Data/restraints/parameters	6099/0/347
Goodness-of-fit on F2	1.044
Final R indexes [I>=2σ (I)]	R1 = 0.0466, wR2 = 0.1224
Final R indexes [all data]	R1 = 0.0577, wR2 = 0.1302
Largest diff. peak/hole / e Å-3	0.25/-0.25

Table S4. Crystallographic data and structure refinement for **2diols-[1,1][2]PCP**

CCDC	2479718
Empirical formula	$C_{30}H_{32}O_6S_2$
Formula weight	552.67
Temperature/K	99.99(10)
Crystal system	triclinic
Space group	P-1
a/Å	8.8569(3)
b/Å	9.1083(3)
c/Å	16.7941(6)
α/°	92.790(3)
β/°	91.558(3)
γ/°	93.496(2)
Volume/Å3	1350.06(8)
Z	2
ρcalcg/cm3	1.360
μ/mm-1	2.145
F(000)	584.0
Crystal size/mm3	0.12 × 0.11 × 0.08
Radiation	Cu Kα (λ = 1.54178)
2Θ range for data collection/°	5.27 to 133.75
Index ranges	-10 ≤ h ≤ 10, -10 ≤ k ≤ 10, -19 ≤ l ≤ 19
Reflections collected	13412
Independent reflections	4654 [Rint = 0.0258, Rsigma = 0.0282]
Data/restraints/parameters	4654/0/351
Goodness-of-fit on F2	1.071
Final R indexes [I>=2σ (I)]	R1 = 0.0416, wR2 = 0.1110
Final R indexes [all data]	R1 = 0.0464, wR2 = 0.1147
Largest diff. peak/hole / e Å-3	0.80/-0.59

Table S5. Crystallographic data and structure refinement for **ketal-2Me-[1,1][2]PCP**

CCDC	2479740
Empirical formula	$C_{32}H_{32}O_2$
Formula weight	448.57
Temperature/K	298.15
Crystal system	monoclinic
Space group	$P2_1/c$
a/Å	9.2334(3)
b/Å	11.3728(3)
c/Å	47.2982(14)
α/°	90
β/°	91.977(2)
γ/°	90
Volume/Å^3	4963.8(3)
Z	8
ρ_{calc}g/cm^3	1.200
μ/mm^{-1}	0.073
F(000)	1920.0
Crystal size/mm^3	0.12 × 0.12 × 0.11
Radiation	Mo Kα (λ = 0.71073)
2Θ range for data collection/°	1.724 to 50.054
Index ranges	$-9 \le h \le 10$, $-13 \le k \le 12$, $-56 \le l \le 56$
Reflections collected	39114
Independent reflections	8707 [R_{int} = 0.0766, R_{sigma} = 0.0817]
Data/restraints/parameters	8707/0/622
Goodness-of-fit on F^2	1.069
Final R indexes [I>=2σ (I)]	R_1 = 0.0650, wR_2 = 0.1719
Final R indexes [all data]	R_1 = 0.1315, wR_2 = 0.2070
Largest diff. peak/hole / e Å^{-3}	0.26/-0.21

Table S6. Crystallographic data and structure refinement for **diol-2Me-[1,1][2]PCP**

CCDC	2479706
Empirical formula	$C_{28}H_{26}O_3$
Formula weight	410.49
Temperature/K	150.00
Crystal system	monoclinic
Space group	$P2_1/c$
a/Å	22.488(3)
b/Å	11.1001(12)
c/Å	8.8428(13)
α/°	90
β/°	99.154(7)
γ/°	90
Volume/Å^3	2179.2(5)
Z	4

ρ_{calc}g/cm^3	1.251
μ/mm^{-1}	0.407
F(000)	872.0
Crystal size/mm^3	0.15 × 0.11 × 0.11
Radiation	Ga Kα (λ = 1.34139)
2Θ range for data collection/°	6.928 to 114.124
Index ranges	-26 ≤ h ≤ 28, -13 ≤ k ≤ 13, -11 ≤ l ≤ 10
Reflections collected	17304
Independent reflections	4417 [R_{int} = 0.1133, R_{sigma} = 0.0717]
Data/restraints/parameters	4417/0/290
Goodness-of-fit on F^2	1.054
Final R indexes [I>=2σ (I)]	R_1 = 0.0487, wR_2 = 0.1344
Final R indexes [all data]	R_1 = 0.0833, wR_2 = 0.1482
Largest diff. peak/hole / e Å^{-3}	0.25/-0.26

Table S7. Crystallographic data and structure refinement for **2ketals-[1,1][3]PCP**

CCDC	2479744
Empirical formula	$C_{46}H_{44}O_4$
Formula weight	660.81
Temperature/K	298.15
Crystal system	monoclinic
Space group	P2$_1$/c
a/Å	11.3613(3)
b/Å	9.1856(3)
c/Å	18.8517(5)
α/°	90
β/°	106.6800(10)
γ/°	90
Volume/Å^3	1884.59(9)
Z	2
ρ_{calc}g/cm^3	1.164
μ/mm^{-1}	0.073
F(000)	704.0
Crystal size/mm^3	0.11 × 0.11 × 0.1
Radiation	Mo Kα (λ = 0.71073)
2Θ range for data collection/°	3.742 to 55.048
Index ranges	-14 ≤ h ≤ 14, -7 ≤ k ≤ 11, -24 ≤ l ≤ 20
Reflections collected	15481
Independent reflections	4278 [R_{int} = 0.0527, R_{sigma} = 0.0405]
Data/restraints/parameters	4278/0/228
Goodness-of-fit on F^2	1.090
Final R indexes [I>=2σ (I)]	R_1 = 0.0422, wR_2 = 0.1136
Final R indexes [all data]	R_1 = 0.0514, wR_2 = 0.1198
Largest diff. peak/hole / e Å^{-3}	0.25/-0.19

Table S8. Crystallographic data and structure refinement for **2diols-[1,1][3]PCP**

CCDC	2479720
Empirical formula	$C_{46}H_{44}O_6$
Formula weight	692.81
Temperature/K	260.00
Crystal system	monoclinic
Space group	$P2_1$
a/Å	9.6955(8)
b/Å	12.4871(9)
c/Å	15.6271(11)
α/°	90
β/°	90.093(3)
γ/°	90
Volume/Å^3	1892.0(2)
Z	2
ρ_{calc}g/cm^3	1.216
μ/mm^{-1}	0.403
F(000)	736.0
Crystal size/mm^3	0.12 × 0.11 × 0.08
Radiation	Ga Kα (λ = 1.34139)
2Θ range for data collection/°	4.92 to 105.952
Index ranges	$-11 \leq h \leq 11$, $-14 \leq k \leq 14$, $-17 \leq l \leq 18$
Reflections collected	13626
Independent reflections	6185 [R_{int} = 0.0847, R_{sigma} = 0.0861]
Data/restraints/parameters	6185/1/474
Goodness-of-fit on F^2	1.131
Final R indexes [I>=2σ (I)]	R_1 = 0.0653, wR_2 = 0.1657
Final R indexes [all data]	R_1 = 0.0760, wR_2 = 0.1771
Largest diff. peak/hole / e Å^{-3}	0.30/-0.34

Table S9. Crystallographic data and structure refinement for **2ketals-[1,1][4]PCP**

CCDC	2479742
Empirical formula	$C_{64}H_{66}O_4$
Formula weight	899.16
Temperature/K	163.00
Crystal system	monoclinic
Space group	$P2_1/c$
a/Å	12.0868(5)
b/Å	23.5414(9)
c/Å	8.7707(3)
α/°	90
β/°	99.2060(10)
γ/°	90
Volume/Å^3	2463.47(16)
Z	2
ρ_{calc}g/cm^3	1.212
μ/mm^{-1}	0.363
F(000)	964.0
Crystal size/mm^3	0.12 × 0.09 × 0.09

Radiation	Ga Kα (λ = 1.34139)
2Θ range for data collection/°	6.446 to 113.904
Index ranges	-15 ≤ h ≤ 15, -29 ≤ k ≤ 22, -10 ≤ l ≤ 10
Reflections collected	19042
Independent reflections	4997 [R_{int} = 0.0345, R_{sigma} = 0.0310]
Data/restraints/parameters	4997/0/310
Goodness-of-fit on F^2	1.030
Final R indexes [I>=2σ (I)]	R_1 = 0.0615, wR_2 = 0.1683
Final R indexes [all data]	R_1 = 0.0671, wR_2 = 0.1736
Largest diff. peak/hole / e $Å^{-3}$	0.71/-0.53

Table S10. Crystallographic data and structure refinement for **2ketones-[1,1][4]PCP**

CCDC	2479725
Empirical formula	$C_{154}H_{104}Cl_8O_6$
Formula weight	2333.97
Temperature/K	298.15
Crystal system	triclinic
Space group	P-1
a/Å	10.2450(7)
b/Å	14.4312(10)
c/Å	22.1091(16)
α/°	108.872(2)
β/°	94.103(3)
γ/°	98.397(2)
Volume/$Å^3$	3035.2(4)
Z	1
$\rho_{calc}g/cm^3$	1.277
μ/mm^{-1}	0.246
F(000)	1212.0
Crystal size/mm^3	0.14 × 0.08 × 0.08
Radiation	Mo Kα (λ = 0.71073)
2Θ range for data collection/°	3.002 to 57.624
Index ranges	-13 ≤ h ≤ 13, -19 ≤ k ≤ 18, -29 ≤ l ≤ 29
Reflections collected	49183
Independent reflections	14931 [R_{int} = 0.0424, R_{sigma} = 0.0408]
Data/restraints/parameters	14931/171/840
Goodness-of-fit on F^2	1.057
Final R indexes [I>=2σ (I)]	R_1 = 0.0657, wR_2 = 0.1974
Final R indexes [all data]	R_1 = 0.0805, wR_2 = 0.2117
Largest diff. peak/hole / e $Å^{-3}$	1.07/-0.82

6. DFT calculations

We carried out the computational studies by using the density functional theory (DFT) in Gaussian 16 program package[5] (revision C. 01). All geometry optimizations of minima were conducted by

using wb97xd/6-311g(d) in gas phase at 298.15 K. Frequency analyses were carried out at the same level to evaluate the zero-point vibrational energy and thermal corrections at 298.15 K. The nature of the stationary points was determined in each case according to the appropriate number of negative eigenvalues of the Hessian matrix. Calculations of single point energies, molecular orbitals and TD-DFT for all DFT-optimized structures were obtained by using the B3LYP/TZVP theory level. Visualization of molecular orbitals was performed by the use of VMD software with 0.02 of iso value. [6-8] The strain visualization was calculated by using the method developed by Jasti et al. [9]

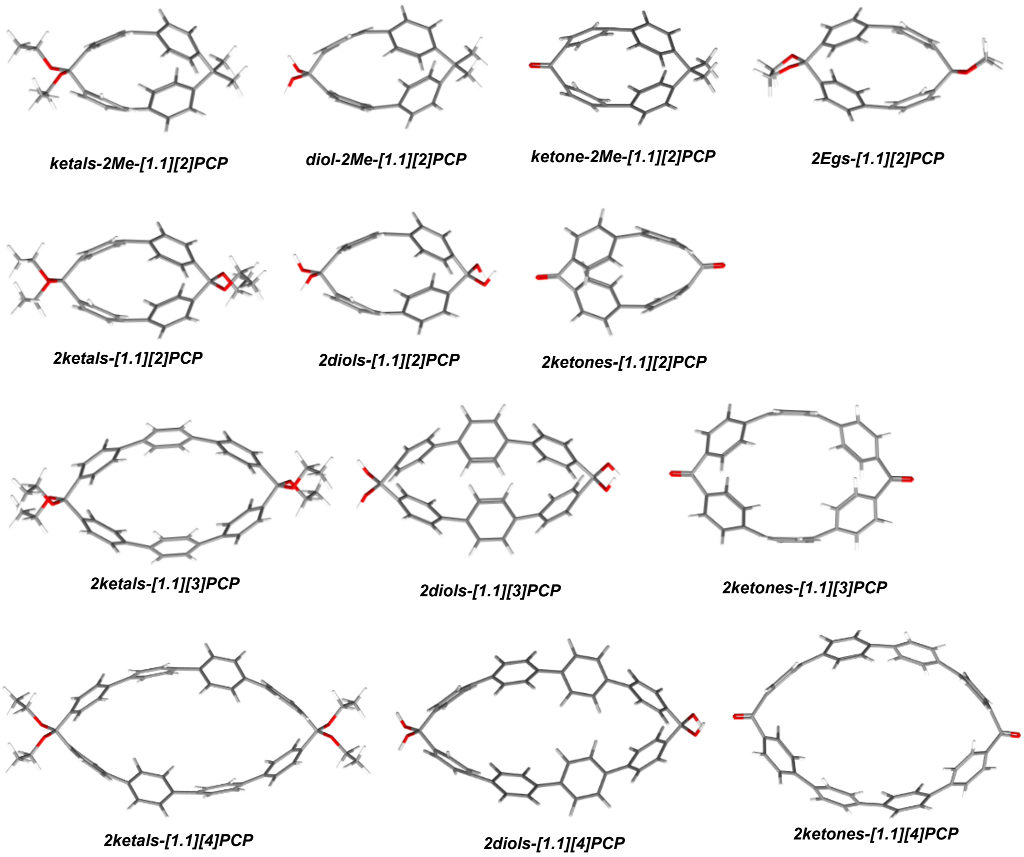

Figure S72. Optimized structures of **ketal-2Me-[1,1][2]PCP**, **diol-2Me-[1,1][2]PCP**, **ketone-2Me-[1,1][2]PCP**, **2dbts-[1,1][2]PCP**, **2ketal-[1,1][n]PCPs (n=2, 3, 4)**, **2diols-[1,1][n]PCPs (n=2, 3, 4)** and **2ketones-[1,1][n]PCPs (n=2, 3, 4)**.

Table S11. Uncorrected and thermal-corrected (298K) energies of stationary points (Hartree).[a]

compound	E	E + ZPE	H	G	HOMO /LUMO	HOMO /LUMO Gaps

**ketal-2Me-[1,1][2]PCP**	-1388.911828	-1388.350662	-1388.320722	--1388.407956	-7.32/ 0.27	7.59
**diol-2Me -[1,1][2]PCP**	-1231.682893	-1231.234561	-1231.210566	-1231.283123	-7.37/ 0.18	7.55
**ketone-2Me -[1,1][2]PCP**	-1155.238382	-1154.819353	-1154.796379	-1154.868319	-7.67/ 0.05	7.72
**2ketal-[1,1][2]PCP**	-1617.958807	-1617.331665	-1617.296464	-1617.397157	-7.33/ 0.21	7.54
**2ketal-[1,1][3]PCP**	-2080.025690	-2079.235205	-2079.190561	-2079.313775	-7.44/ 0.25	7.69
**2ketal-[1,1][4]PCP**	-2542.075926	-2541.122207	-2541.068223	-2541.213463	-7.46/ 0.19	7.65
**2diols-[1,1][2]PCP**	-1303.500877	-1303.099871	-1303.076492	-1303.147997	-7.38/ 0.13	7.51
**2diols-[1,1][3]PCP**	-1765.567178	-1765.002555	-1764.969928	-1765.062518	-7.52/ 0.10	7.62
**2diols-[1,1][4]PCP**	-2227.619466	-2226.892076	-2226.849905	-2226.965231	-7.60/ 0.10	7.69
**2ketones-[1,1][2]PCP**	-1150.611086	-1150.268567	-1150.247264	-1150.316619	-8.05/ -0.26	7.79
**2ketones-[1,1][3]PCP**	-1612.681289	-1612.174391	-1612.144077	-1612.234747	-7.99/ -0.31	7.68
**2ketones-[1,1][4]PCP**	-2074.735692	-2074.065528	-2074.025847	-2074.138768	-7.86/ -0.43	7.43
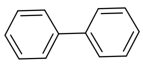	-463.230470	-463.047631	-463.037868	-463.082188	-	-
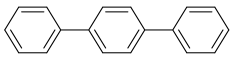	-694.252393	-693.987956	-693.973564	-694.029372	-	-
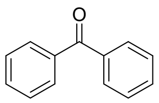	-576.549922	-576.356591	-576.345036	-576.394009	-	-
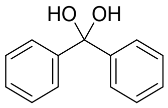	-652.974958	-652.753992	-652.740845	-652.793699	-	-
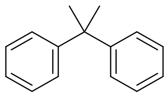	-581.157933	-580.889509	-580.875937	-580.928777	-	-
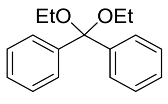	-810.202538	-809.868791	-809.849680	-809.917690	-	-

[a]E = electronic energy; ZPE = zero-point-energy; H (= E+ZPE+E_{vib}+E_{rot}+E_{trans}+RT): sum of electronic and thermal enthalpies; G(=H-TS): sum of electronic and thermal free energies.

Table S12. Strain energies (**SE**, kcal mol^{-1}, **SE** (ΔH) = n $\boldsymbol{H}$(**TPE**) – n $\boldsymbol{H}$(**BTPE**) + $\boldsymbol{H}$(**[n]*c*-CTPE**)) of **[n]*c*-CTPEs** based on Homodesmotic Reaction.[10-12]

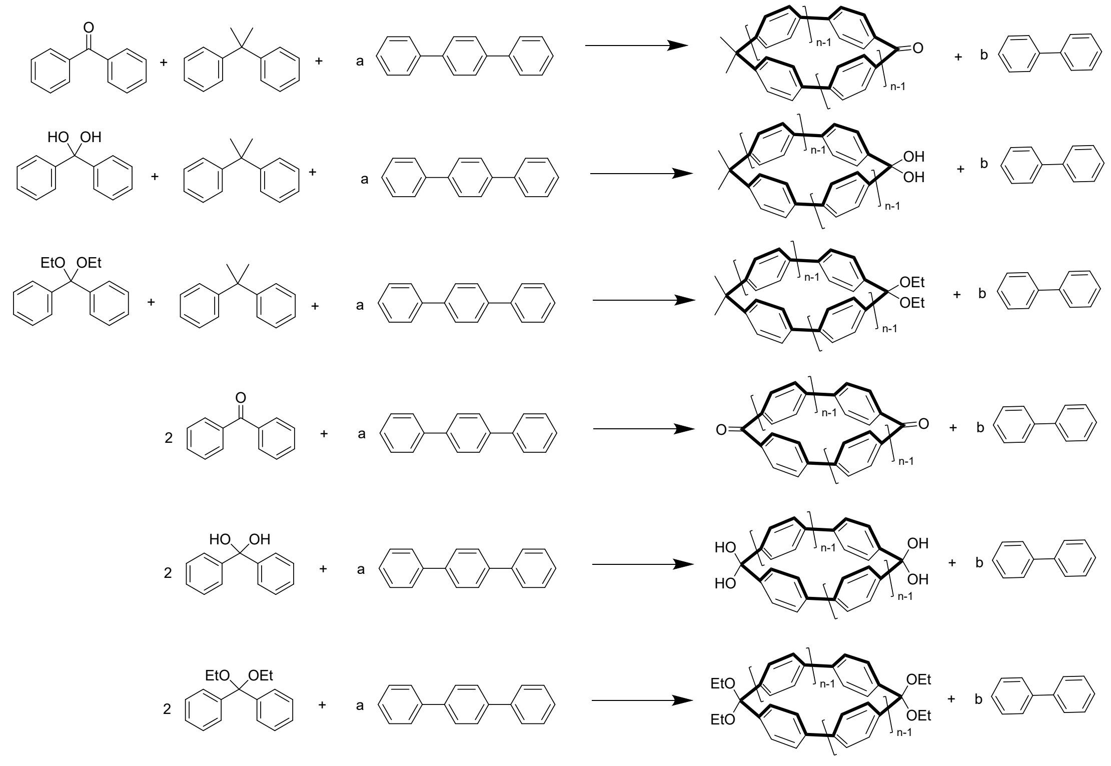

	n	a	b	Strain energies (kcal/mol)
**ketal-2Me-[1,1][2]PCP**	2	4	6	50.29
**diol-2Me -[1,1][2]PCP**	2	4	6	45.96
**ketone-2Me -[1,1][2]PCP**	2	4	6	57.51
**2ketal-[1,1][2]PCP**	2	4	6	43.89
**2ketal-[1,1][3]PCP**	3	6	8	29.64
**2ketal-[1,1][4]PCP**	4	8	10	25.70
**2diols-[1,1][2]PCP**	2	4	6	45.33
**2diols-[1,1][3]PCP**	3	6	8	31.33
**2diols-[1,1][4]PCP**	4	8	10	26.12

**2ketones-[1,1][2]PCP**	2	4	6	68.94
**2ketones-[1,1][3]PCP**	3	6	8	52.98
**2ketones-[1,1][4]PCP**	4	8	10	46.40

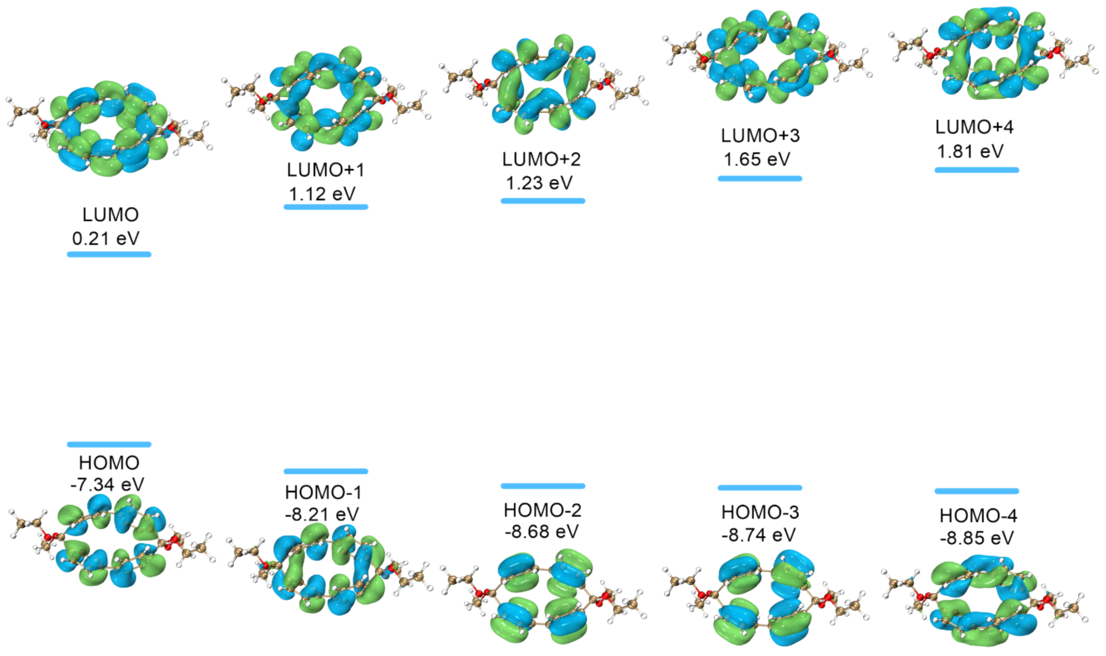

Figure S73. Frontier molecular orbitals of **2ketals-[1,1][2]PCP**.

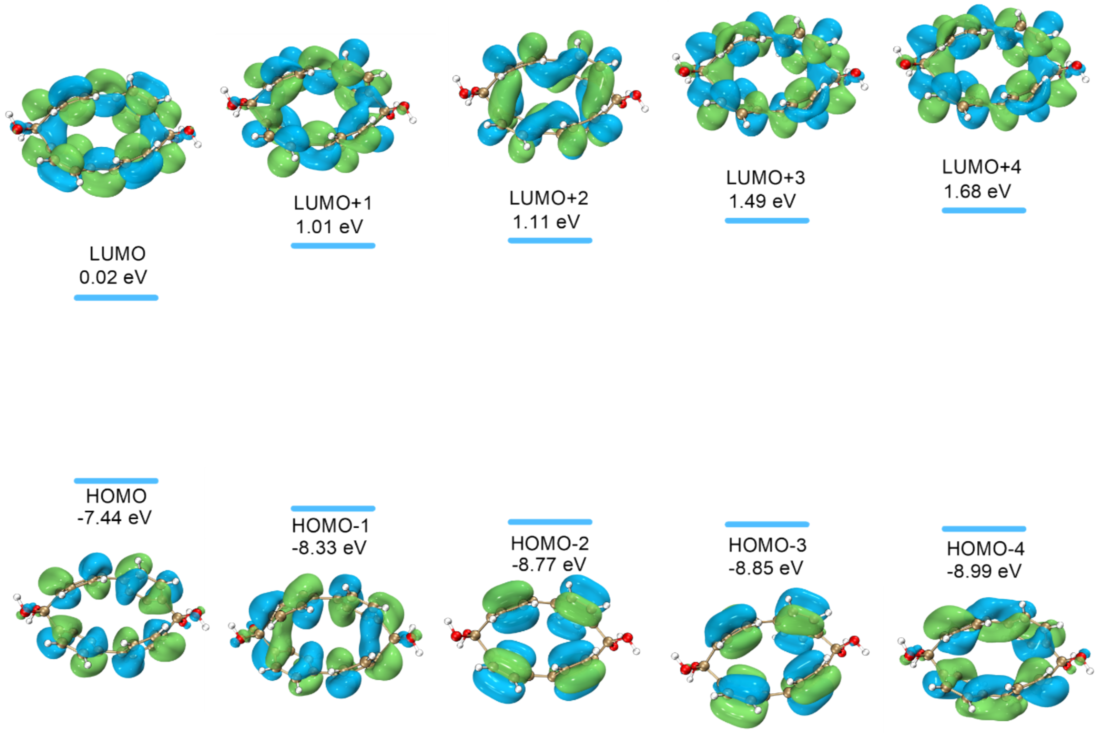

Figure S74. Frontier molecular orbitals of **2diols-[1,1][2]PCP**.

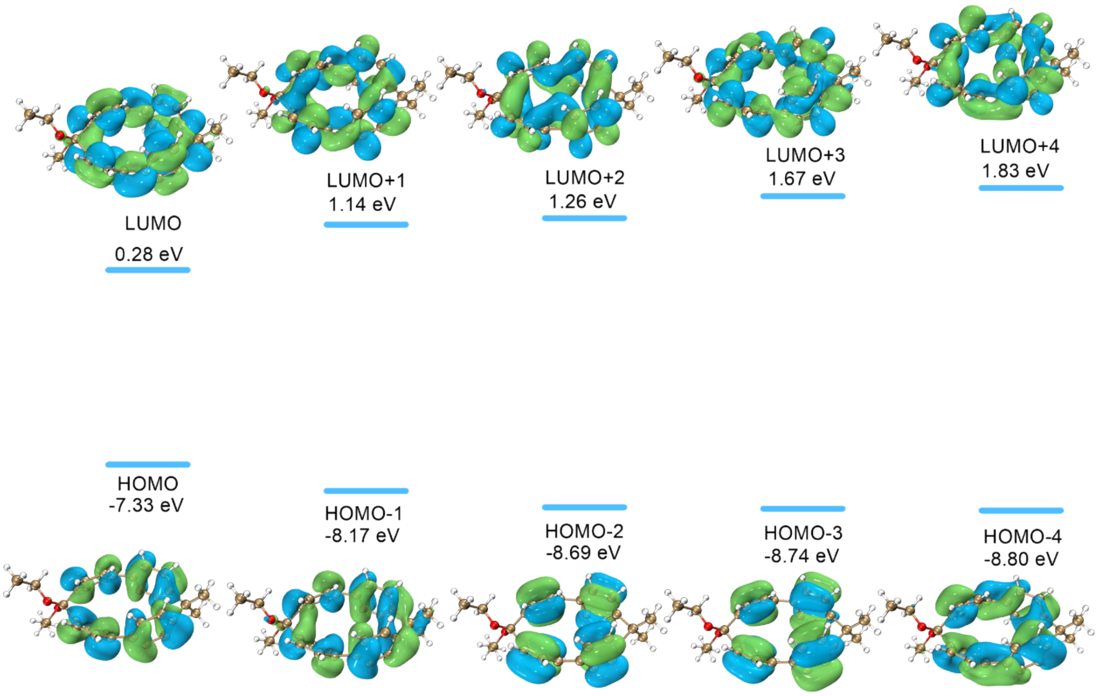

Figure S75. Frontier molecular orbitals of **ketal-2Me-[1,1][2]PCP**.

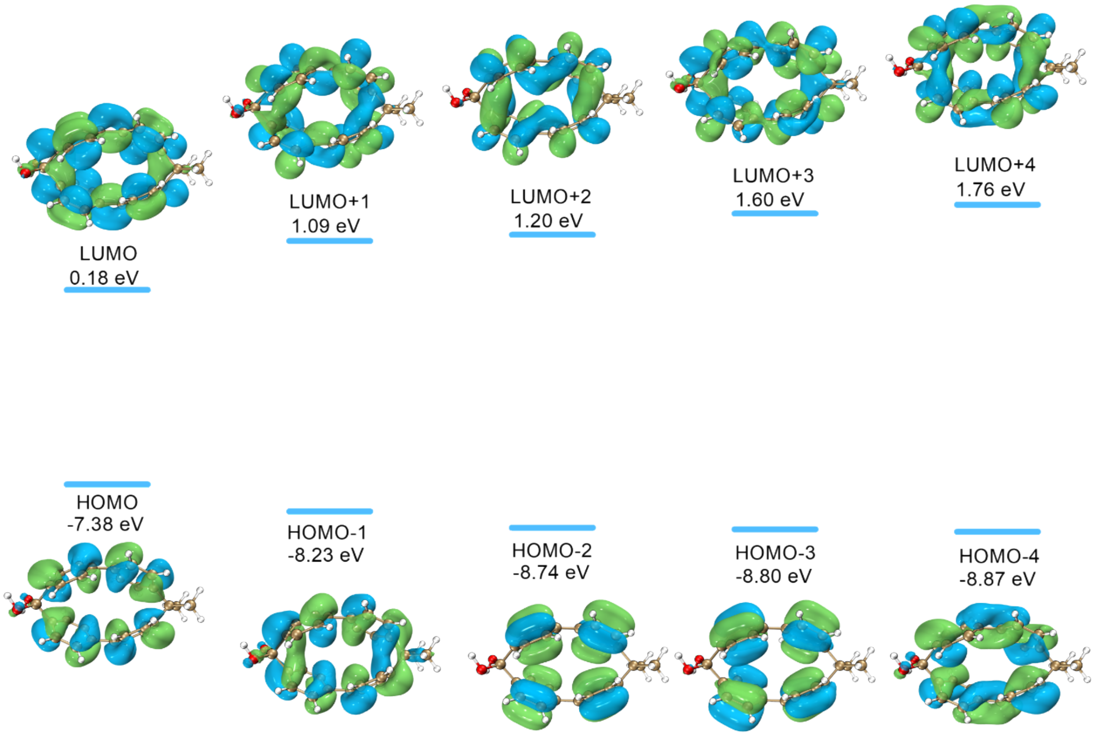

Figure S76. Frontier molecular orbitals of **diol-2Me-[1,1][2]PCP**..

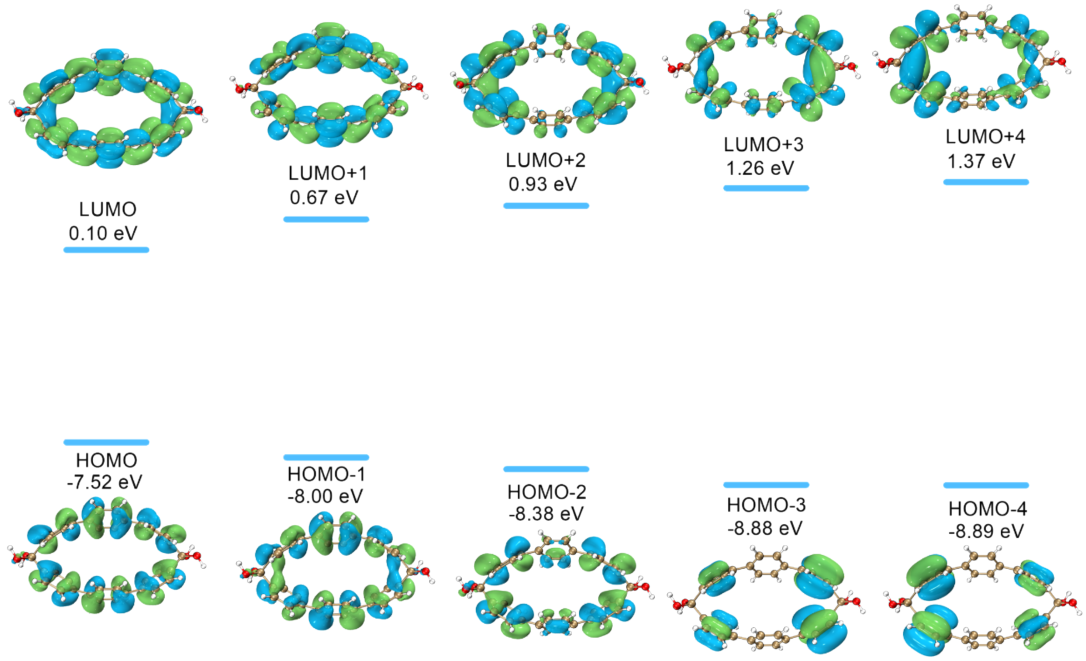

Figure S77. Frontier molecular orbitals of **2diols-[1,1][3]PCP**..

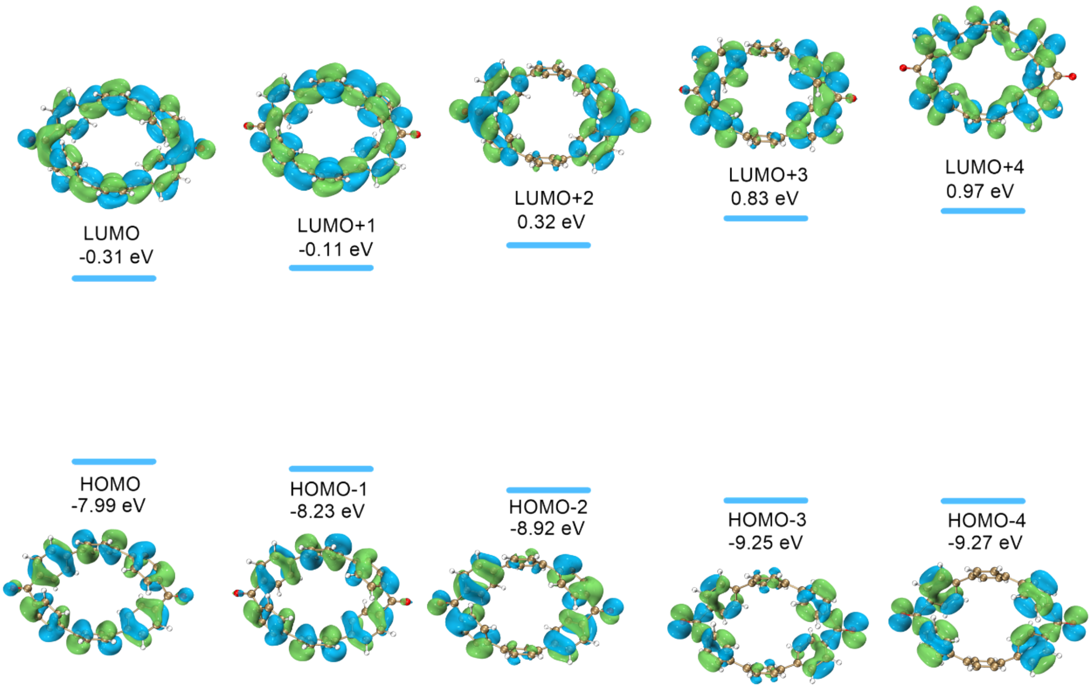

Figure S78. Frontier molecular orbitals of **2ketones-[1,1][3]PCP**..

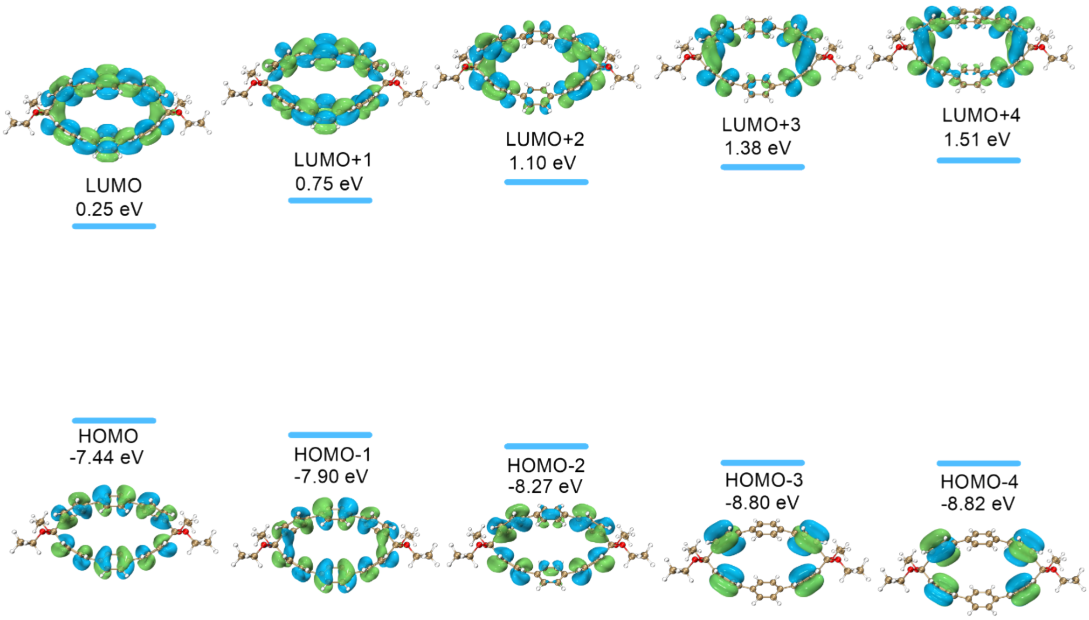

Figure S79. Frontier molecular orbitals of **2ketals-[1,1][3]PCP**.

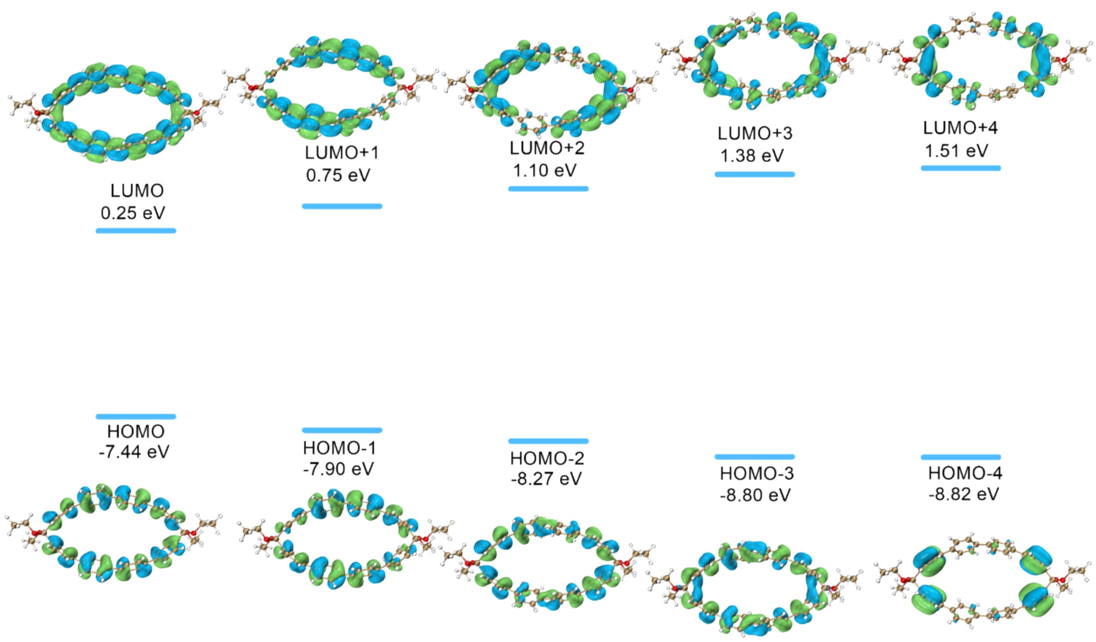

Figure S80. Frontier molecular orbitals of **2ketals-[1,1][4]PCP**.

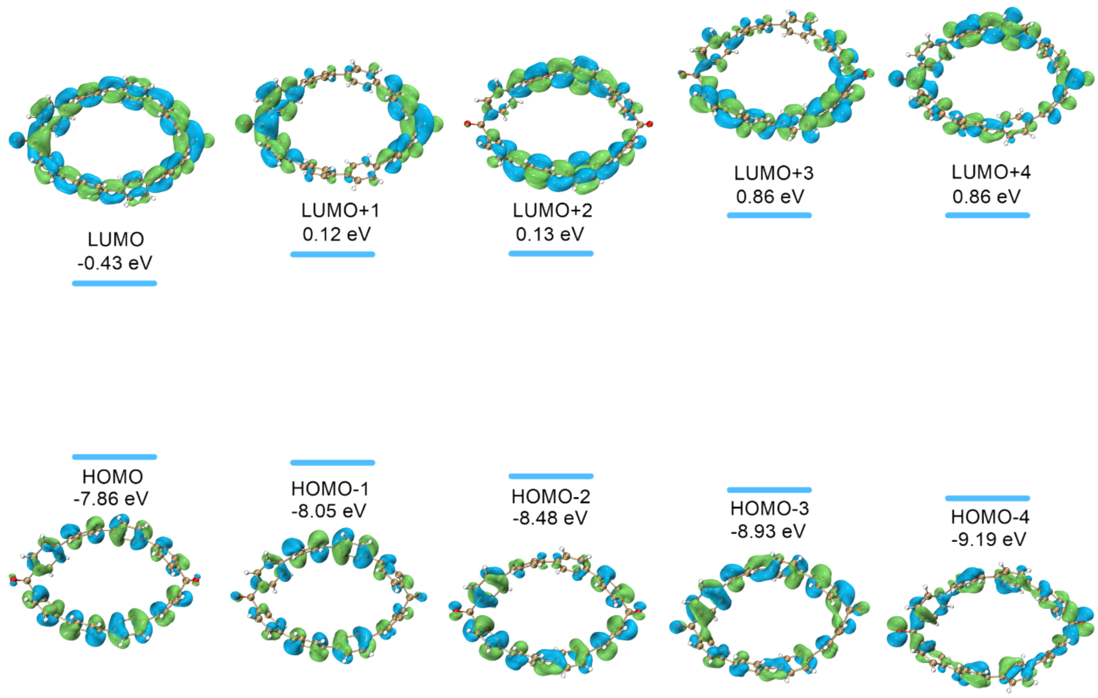

Figure S81. Frontier molecular orbitals of **2ketones-[1,1][2]PCP**.

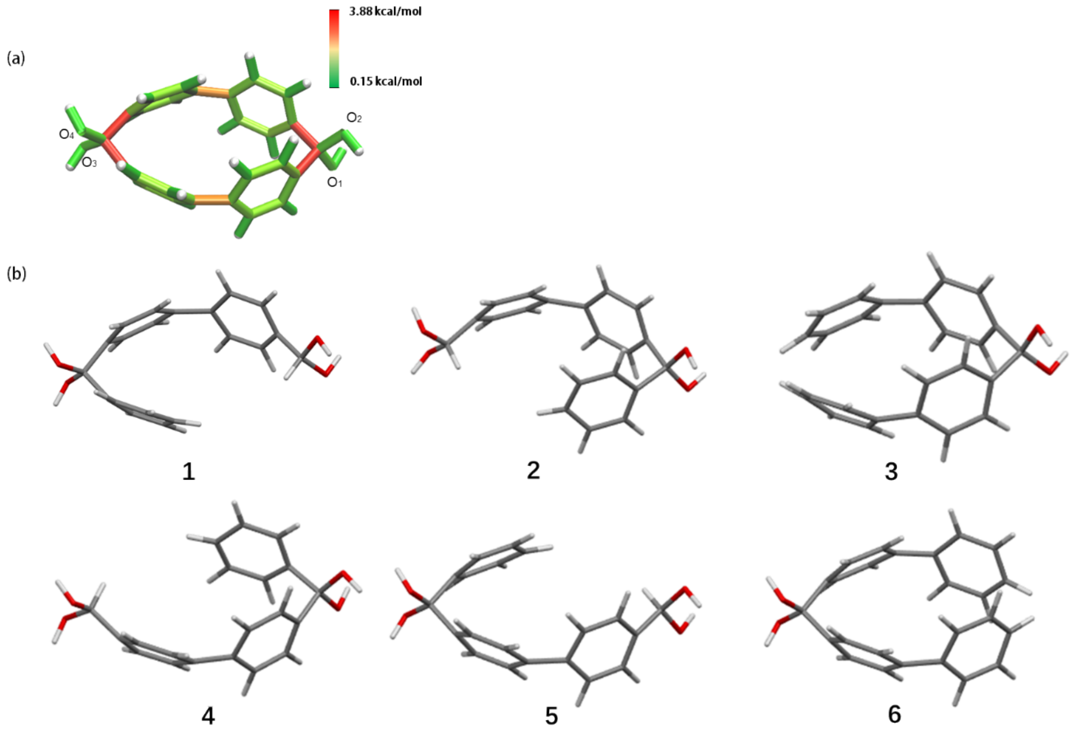

Figure S82. (a) Strainvis analysis of **2diols-[1,1][2]PCP**. (b) Molecule fragments for use in StrainViz.

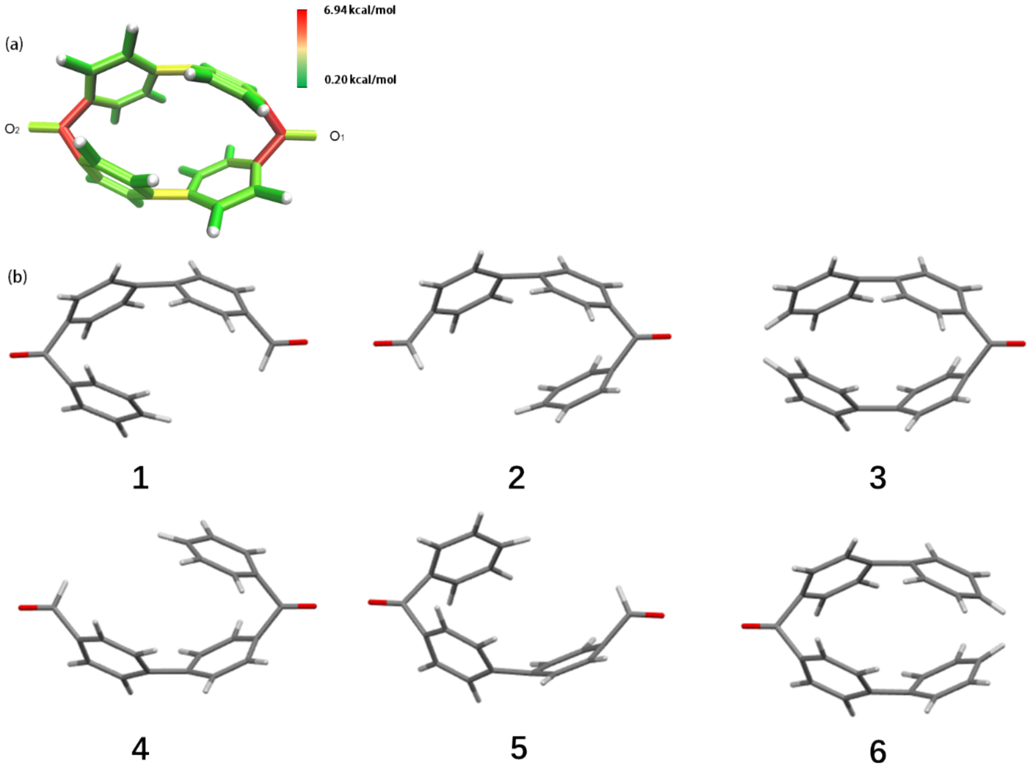

Figure S83. (a) Strainvis analysis of **2ketones-[1,1][2]PCP**. (b) Molecule fragments for use in StrainViz.

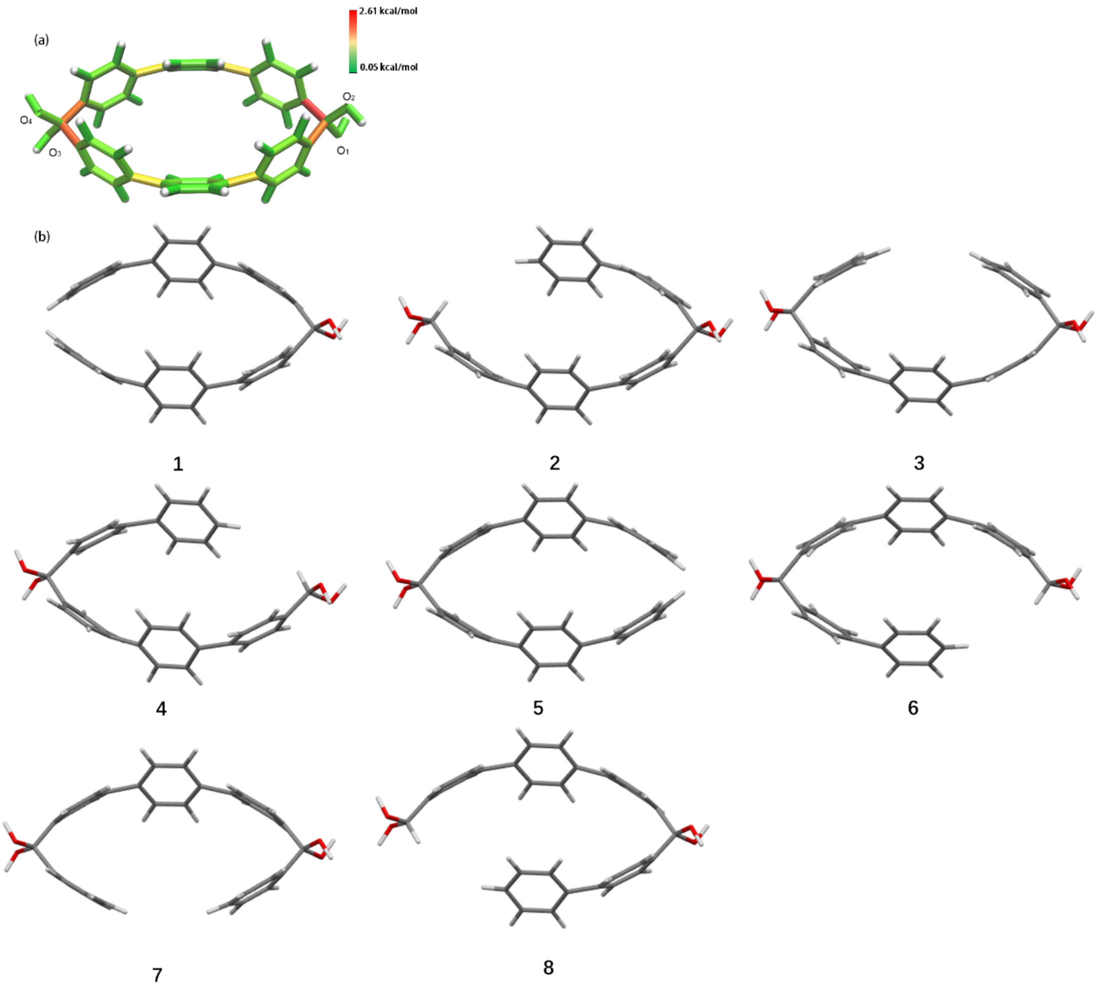

Figure S84. (a) Strainvis analysis of **2diols-[1,1][3]PCP**. (b) Molecule fragments for use in StrainViz.

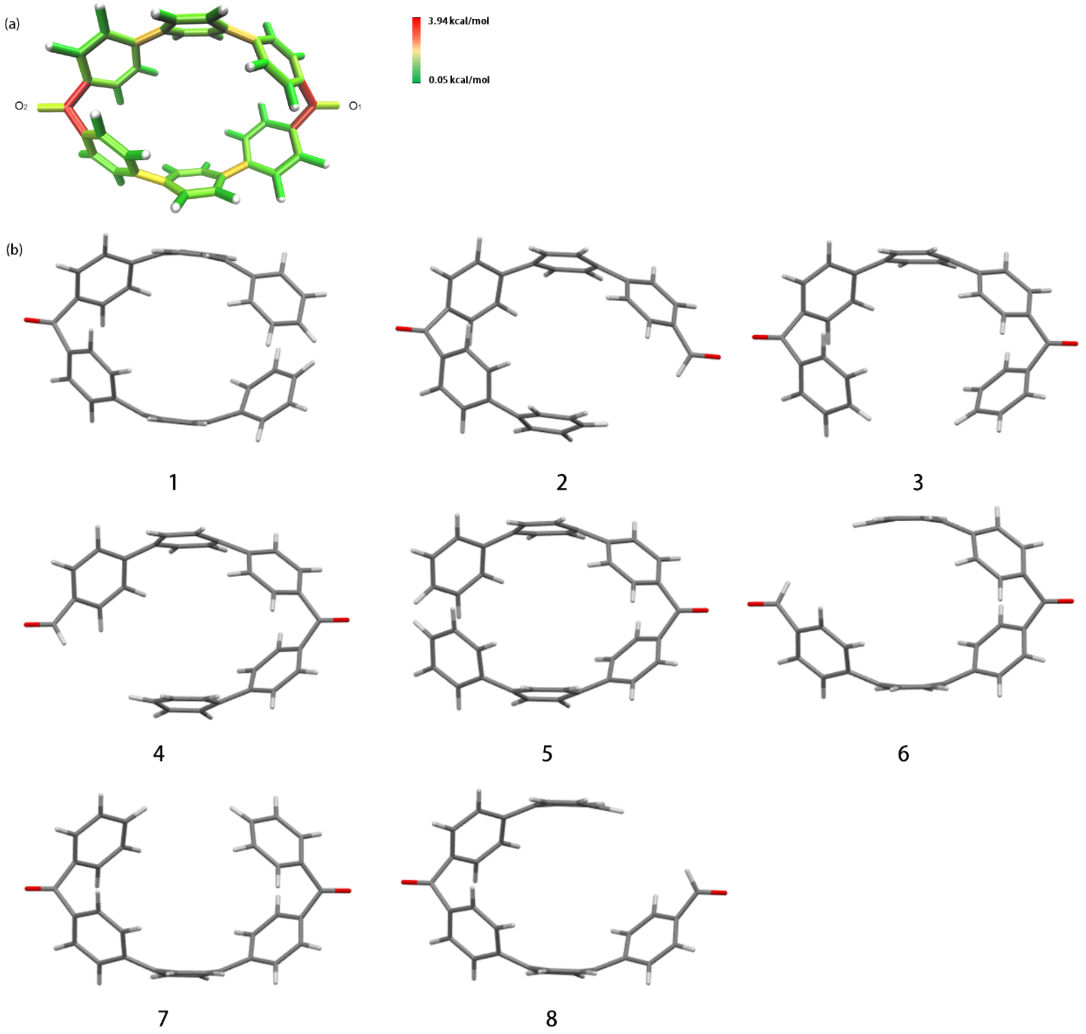

Figure S85. (a) Strainvis analysis of **2ketones-[1,1][3]PCP**. (b) Molecule fragments for use in StrainViz.

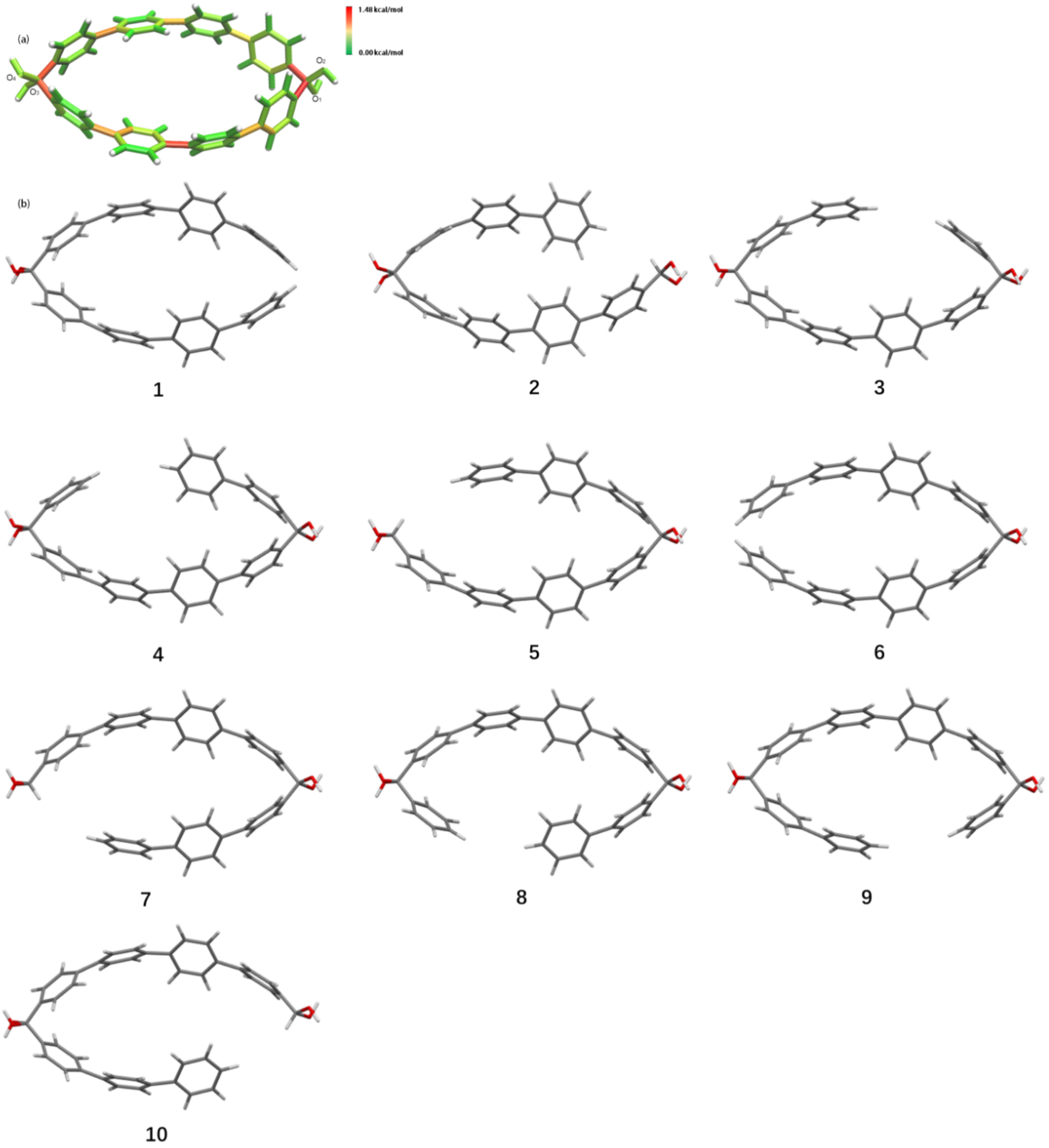

Figure S86. (a) Strainvis analysis of **2diols-[1,1][4]PCP**. (b) Molecule fragments for use in StrainViz.

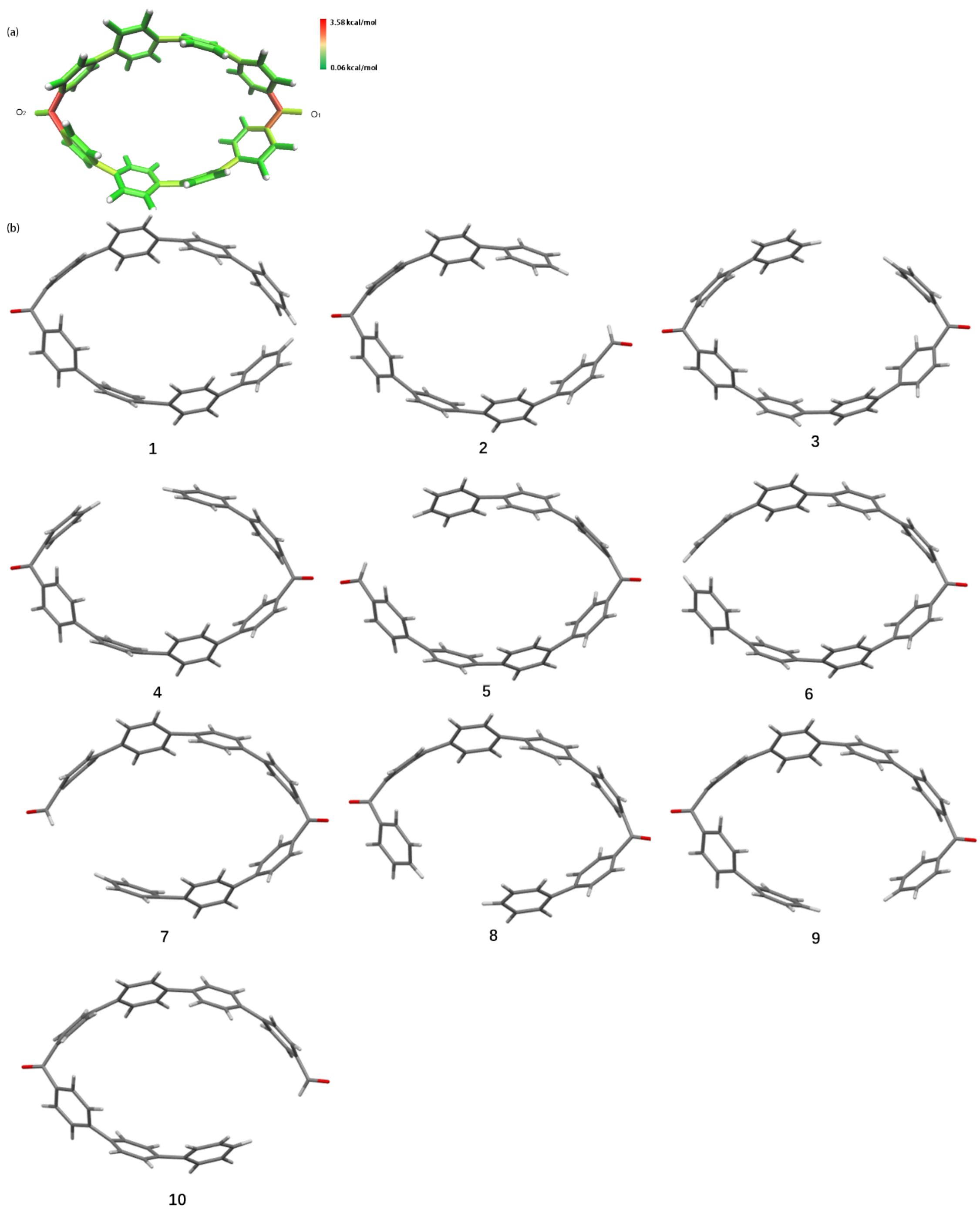

Figure S87. (a) Strainvis analysis of **2ketones-[1,1][4]PCP**. (b) Molecule fragments for use in StrainViz.

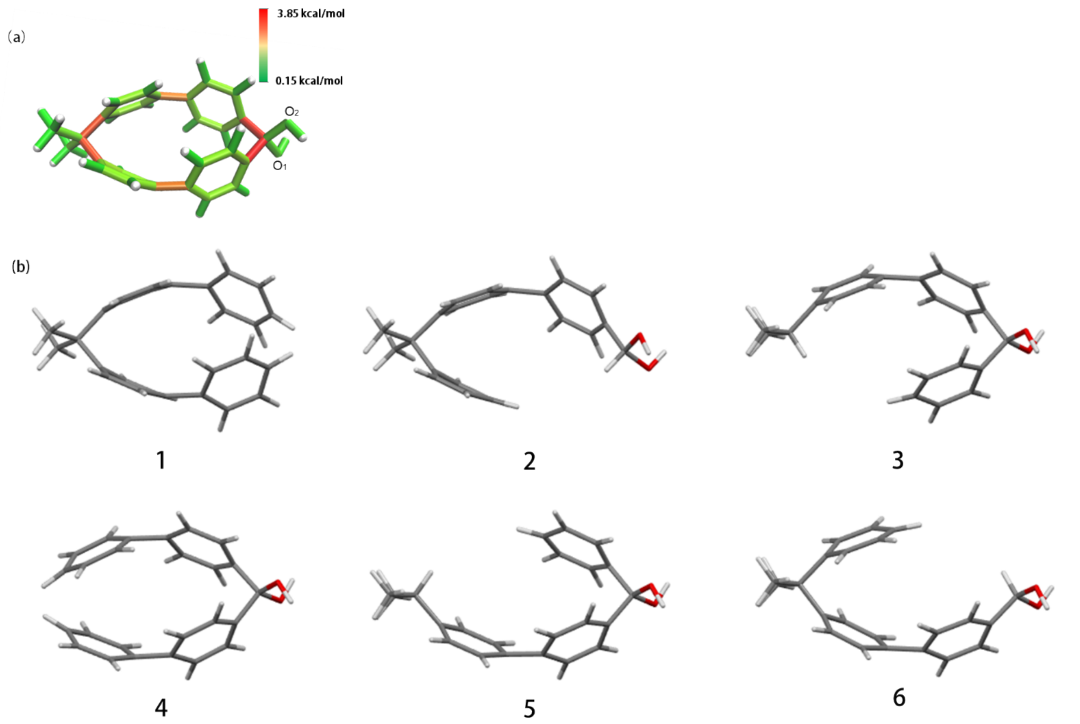

Figure S88. (a) Strainvis analysis of **diol-2Me-[1,1][2]PCP**. (b) Molecule fragments for use in StrainViz.

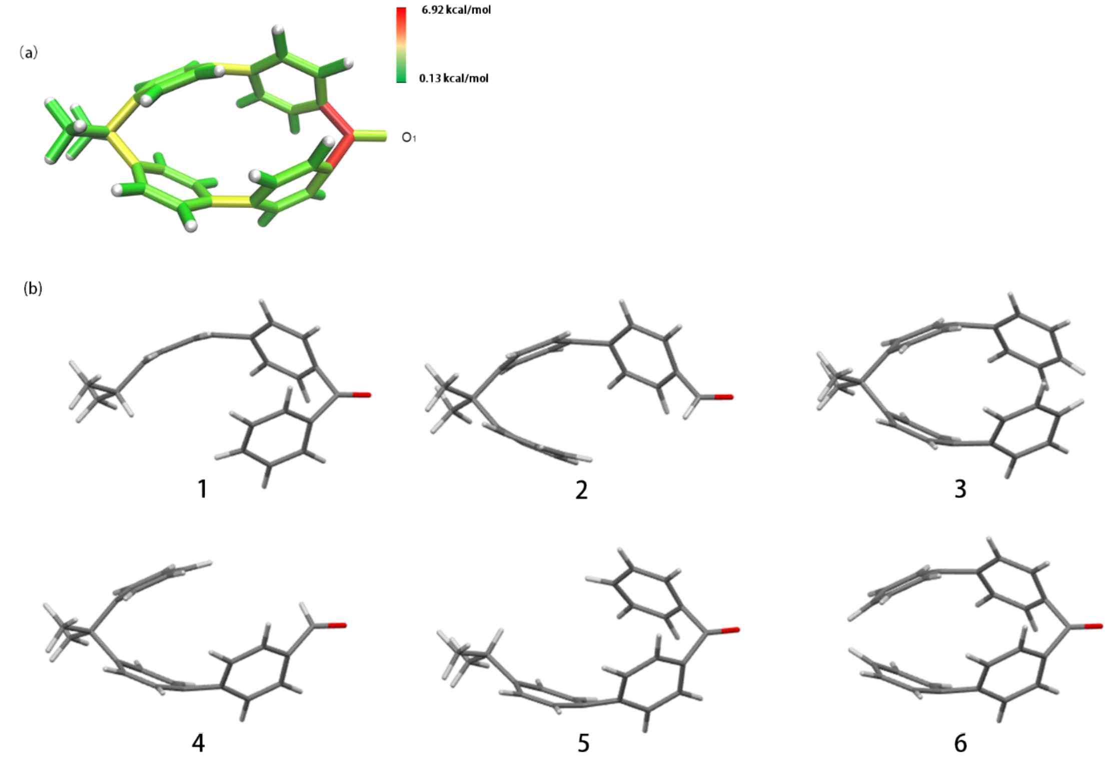

Figure S89. (a) Strainvis analysis of **ketone-2Me-[1,1][2]PCP**. (b) Molecule fragments for use in StrainViz.

Table S13. Electronic transitions for 2**ketals-[1,1][2]PCP** determined by TD-DFT methods at B3LYP/ TZVP level.

ExcitedState	Energy(eV)	Wavelength(nm)	f	Transitions
$S_0 \to S_1$	3.2655	379.67	0.0002	HOMO→LUMO (0.70593)
$S_0 \to S_2$	4.0662	304.91	0.0000	HOMO–2→LUMO (–0.29077)<br>HOMO→LUMO+2 (0.63352)
$S_0 \to S_3$	4.1401	299.47	0.0002	HOMO–3→LUMO (–0.14975)<br>HOMO–1→LUMO (–0.44035)<br>HOMO→LUMO+1 (0.52891)
$S_0 \to S_4$	4.2503	291.71	0.2048	HOMO–3→LUMO (–0.34448)<br>HOMO–1→LUMO (0.46645)<br>HOMO→LUMO+1 (0.28236)<br>HOMO→LUMO+4 (0.25391)
$S_0 \to S_5$	4.5387	273.17	0.0000	HOMO–2→LUMO (0.63003)<br>HOMO→LUMO+2 (0.29773)
$S_0 \to S_6$	4.5418	272.99	0.0126	HOMO–5→LUMO (–0.30041)<br>HOMO–4→LUMO (0.37468)<br>HOMO–2→LUMO+1 (0.14086)<br>HOMO–1→LUMO+2 (–0.26460)<br>HOMO→LUMO+3 (0.13664)<br>HOMO→LUMO+6 (0.38302)
$S_0 \to S_7$	4.6078	269.07	0.0082	HOMO–5→LUMO (–0.11868)<br>HOMO–4→LUMO (–0.35035)<br>HOMO→LUMO+3 (0.59465)
$S_0 \to S_8$	4.6351	267.49	0.5351	HOMO–3→LUMO (0.51610)<br>HOMO–1→LUMO (0.24758)<br>HOMO→LUMO+1 (0.35621)<br>HOMO→LUMO+4 (–0.14338)
$S_0 \to S_9$	4.7217	262.59	0.0013	HOMO–6→LUMO (0.45064)<br>HOMO–3→LUMO+1 (0.13058)<br>HOMO→LUMO+1 (–0.34197)<br>HOMO→LUMO+4 (–0.11234)<br>HOMO→LUMO+6 (0.34672)
$S_0 \to S_{10}$	4.8052	258.02	0.0598	HOMO–5→LUMO (0.19854)<br>HOMO–4→LUMO (0.42455)<br>HOMO–3→LUMO+2 (0.10805)<br>HOMO→LUMO+3 (0.26990)<br>HOMO→LUMO+6 (–0.41111)

Table S14. Electronic transitions for **ketal-2Me-[1,1][2]PCP** determined by TD-DFT methods at B3LYP/ TZVP level.

ExcitedState	Energy(eV)	Wavelength(nm)	f	Transitions
**$S_0 \to S_1$**	3.3188	373.58	0.0002	HOMO→LUMO (0.70590)
**$S_0 \to S_2$**	4.0940	302.84	0.0001	HOMO–2→LUMO (0.26351)<br>HOMO→LUMO+2 (0.64199)
**$S_0 \to S_3$**	4.1563	298.30	0.0014	HOMO–3→LUMO (–0.16617)<br>HOMO–1→LUMO (–0.38863)<br>HOMO→LUMO+1 (0.55893)
**$S_0 \to S_4$**	4.2709	290.30	0.2053	HOMO–3→LUMO (–0.29515)<br>HOMO–1→LUMO (0.51469)<br>HOMO→LUMO+1 (0.24809)

				HOMO→LUMO+4 (0.25619)
**$S_0→S_5$**	4.5631	271.71	0.0179	HOMO–5→LUMO (0.27980)<br>HOMO–4→LUMO (–0.33503)<br>HOMO–2→LUMO+1 (0.14386)<br>HOMO–1→LUMO+2 (0.27368)<br>HOMO→LUMO+3 (–0.23115)<br>HOMO→LUMO+5 (0.37235)
**$S_0→S_6$**	4.6024	269.39	0.0020	HOMO–2→LUMO (0.63731)<br>HOMO→LUMO+2 (–0.26922)
**$S_0→S_7$**	4.6256	268.04	0.0059	HOMO–4→LUMO (–0.39821)<br>HOMO→LUMO+3 (0.56892)
**$S_0→S_8$**	4.6752	265.19	0.4602	HOMO–3→LUMO (0.52656)<br>HOMO–1→LUMO (0.23548)<br>HOMO–1→LUMO+6 (–0.10445)<br>HOMO→LUMO+1 (0.32779)<br>HOMO→LUMO+4 (–0.17567)
**$S_0→S_9$**	4.7241	262.45	0.0005	HOMO–6→LUMO (–0.36836)<br>HOMO–5→LUMO (–0.14067)<br>HOMO–3→LUMO+1 (–0.13580)<br>HOMO–1→LUMO+1 (0.39325)<br>HOMO→LUMO+6 (0.35990)
**$S_0→S_{10}$**	4.8277	256.82	0.0532	HOMO–5→LUMO (0.15239)<br>HOMO–4→LUMO (0.40104)<br>HOMO–3→LUMO+2 (0.10382)<br>HOMO–1→LUMO+2 (–0.10775)<br>HOMO→LUMO+3 (0.24271)<br>HOMO→LUMO+5 (0.45693)

Table S15. Electronic transitions for **2ketals-[1,1][3]PCP** determined by TD-DFT methods at B3LYP/ TZVP level.

**ExcitedState**	**Energy(eV)**	**Wavelength(nm)**	**f**	**Transitions**
$S_0→S_1$	3.5230	351.92	0.0000	HOMO→LUMO (0.70130)
$S_0→S_2$	4.0712	304.54	0.0288	HOMO–1→LUMO (0.55433)<br>HOMO→LUMO+1 (0.43446)
$S_0→S_3$	4.1994	295.24	1.1835	HOMO–1→LUMO (–0.42335)<br>HOMO→LUMO+1 (0.53105)<br>HOMO→LUMO+4 (0.11187)
$S_0→S_4$	4.3653	284.02	0.1235	HOMO–2→LUMO (0.69265)
$S_0→S_5$	4.3804	283.04	0.1123	HOMO→LUMO+3 (0.69090)
$S_0→S_6$	4.4077	281.29	0.0000	HOMO–4→LUMO+3 (0.12336)<br>HOMO–3→LUMO (–0.29060)<br>HOMO–2→LUMO+4 (–0.13331)<br>HOMO→LUMO+2 (0.58498)
$S_0→S_7$	4.4765	276.97	0.0000	HOMO–1→LUMO+1 (0.68549)
$S_0→S_8$	4.5155	274.57	0.2468	HOMO–4→LUMO (0.35431)<br>HOMO–3→LUMO+2 (–0.12622)<br>HOMO–2→LUMO+3 (–0.14664)<br>HOMO→LUMO+1 (–0.14527)<br>HOMO→LUMO+4 (0.53365)
$S_0→S_9$	4.5577	272.03	0.0076	HOMO–6→LUMO (–0.31507)<br>HOMO–5→LUMO+1 (0.16136)<br>HOMO–1→LUMO+2 (–0.11976)<br>HOMO–1→LUMO+5 (0.19478)<br>HOMO→LUMO+6 (0.54527)

$S_0 \to S_{10}$	4.5820	270.59	0.0000	HOMO–9→LUMO (0.15740)<br>HOMO–6→LUMO+1 (0.17339)<br>HOMO–5→LUMO (–0.28616)<br>HOMO–3→LUMO (–0.17904)<br>HOMO–1→LUMO+5 (0.20972)<br>HOMO→LUMO+6 (0.50622)

Table S16. Electronic transitions for **2ketals-[1,1][4]PCP** determined by TD-DFT methods at B3LYP/ TZVP level.

ExcitedState	Energy(eV)	Wavelength(nm)	f	Transitions
$S_0 \to S_1$	3.5600	348.27	0.0000	HOMO→LUMO (0.68728)<br>HOMO-1→LUMO+1 (0.11181)<br>HOMO-2→LUMO+2 (0.10965)
$S_0 \to S_2$	4.0102	309.17	0.8564	HOMO-1→LUMO (0.70110)
$S_0 \to S_3$	4.0664	304.90	0.6727	HOMO→LUMO (0.29180)<br>HOMO-2→LUMO+1 (0.62199)<br>HOMO-2→LUMO+2 (0.14450)
$S_0 \to S_4$	4.0946	302.80	0.7928	HOMO→LUMO (0.49203)<br>HOMO-2→LUMO+1 (–0.32004)<br>HOMO-2→LUMO+2 (0.38353)
$S_0 \to S_5$	4.1559	298.33	0.0417	HOMO→LUMO (–0.40546)<br>HOMO-2→LUMO+2 (0.56736)
$S_0 \to S_6$	4.3162	287.26	0.0000	HOMO-2→LUMO+2 (0.16541)<br>HOMO-1→LUMO+1 (0.66293)<br>HOMO→LUMO+3 (–0.13563)
$S_0 \to S_7$	4.3556	284.65	0.0000	HOMO-1→LUMO+1 (–0.43281)<br>HOMO-1→LUMO+2 (0.53081)<br>HOMO-2→LUMO+3 (–0.11473)
$S_0 \to S_8$	4.3959	282.04	0.0000	HOMO-3→LUMO+2 (–0.12785)<br>HOMO-3→LUMO+1 (0.24274)<br>HOMO→LUMO+4 (–0.20327)<br>HOMO-1→LUMO+1 (0.13899)<br>HOMO-1→LUMO+4 (–0.10422)<br>HOMO-1→LUMO+5 (0.10545)<br>HOMO→LUMO+3 (0.53204)<br>HOMO→LUMO+5 (0.12019)
$S_0 \to S_9$	4.4192	280.56	0.0049	HOMO-3→LUMO+2 (0.27481)<br>HOMO-3→LUMO+3 (–0.14068)<br>HOMO-1→LUMO+2 (–0.21864)<br>HOMO-1→LUMO+3 (–0.12316)<br>HOMO→LUMO+4 (0.53291)
$S_0 \to S_{10}$	4.5150	274.60	0.0000	HOMO→LUMO+1 (0.41175)<br>HOMO→LUMO+2 (0.45611)<br>HOMO-1→LUMO+1 (0.29216)

Table S17. Electronic transitions for **2dbts-[1,1][2]PCP** determined by TD-DFT methods at B3LYP/ TZVP level.

ExcitedState	Energy(eV)	Wavelength(nm)	f	Transitions
$S_0 \to S_1$	3.2357	383.17	0.0001	HOMO→LUMO (0.70584)
$S_0 \to S_2$	4.0222	308.25	0.0000	HOMO–2→LUMO (0.29148)

				HOMO→LUMO+1 (0.63362)
$S_0 \rightarrow S_3$	4.1218	300.80	0.0089	HOMO–3→LUMO (–0.11023)<br>HOMO–1→LUMO (0.50954)<br>HOMO→LUMO+2 (0.47373)
$S_0 \rightarrow S_4$	4.2404	292.39	0.1701	HOMO–3→LUMO (0.38520)<br>HOMO–1→LUMO (0.38415)<br>HOMO→LUMO+2 (–0.33112)<br>HOMO→LUMO+4 (0.25350)
$S_0 \rightarrow S_5$	4.4940	275.89	0.0000	HOMO–2→LUMO (0.63028)<br>HOMO→LUMO+1 (–0.29759)
$S_0 \rightarrow S_6$	4.5196	274.32	0.0147	HOMO–5→LUMO (–0.29274)<br>HOMO–4→LUMO (0.34823)<br>HOMO–3→LUMO (–0.14984)<br>HOMO–2→LUMO+2 (0.13586)<br>HOMO–1→LUMO+1 (–0.28065)<br>HOMO→LUMO+3 (0.14798)<br>HOMO→LUMO+5 (0.36209)
$S_0 \rightarrow S_7$	4.5959	269.77	0.2399	HOMO–5→LUMO (–0.12651)<br>HOMO–4→LUMO (–0.19645)<br>HOMO–3→LUMO (0.31433)<br>HOMO–1→LUMO (–0.18213)<br>HOMO→LUMO+2 (0.26553)<br>HOMO→LUMO+3 (0.47037)
$S_0 \rightarrow S_8$	4.6331	267.61	0.2748	HOMO–4→LUMO (0.32545)<br>HOMO–3→LUMO (0.34995)<br>HOMO–1→LUMO (–0.17907)<br>HOMO→LUMO+2 (0.27837)<br>HOMO→LUMO+3 (–0.34262)<br>HOMO→LUMO+4 (0.15905)
$S_0 \rightarrow S_9$	4.7356	261.81	0.0022	HOMO–6→LUMO (0.44920)<br>HOMO–3→LUMO+2 (–0.13737)<br>HOMO–1→LUMO+2 (–0.36041)<br>HOMO–1→LUMO+4 (0.12102)<br>HOMO→LUMO+6 (–0.32053)
$S_0 \rightarrow S_{10}$	4.8039	258.09	0.0788	HOMO–4→LUMO (–0.21808)<br>HOMO–3→LUMO (–0.20203)<br>HOMO–1→LUMO (–0.10072)<br>HOMO–1→LUMO+1 (0.13935)<br>HOMO→LUMO+4 (0.58078)<br>HOMO→LUMO+5 (0.12548)

Table S18. Electronic transitions for **diol-2Me-[1,1][2]PCP** determined by TD-DFT methods at B3LYP/ TZVP level.

ExcitedState	Energy(eV)	Wavelength(nm)	f	Transitions
$S_0 \rightarrow S_1$	3.2768	378.37	0.0002	HOMO→LUMO (0.70597)
$S_0 \rightarrow S_2$	4.0780	304.03	0.0001	HOMO–2→LUMO (0.27134)<br>HOMO→LUMO+1 (0.10181)<br>HOMO→LUMO+2 (0.63079)
$S_0 \rightarrow S_3$	4.1417	299.35	0.0011	HOMO–3→LUMO (–0.13294)<br>HOMO–1→LUMO (–0.45937)<br>HOMO→LUMO+1 (0.50919)
$S_0 \rightarrow S_4$	4.2545	291.42	0.1750	HOMO–3→LUMO (–0.33176)<br>HOMO–1→LUMO (0.44994)<br>HOMO→LUMO+1 (0.30212)

				HOMO→LUMO+4 (0.26448)
$S_0 \rightarrow S_5$	4.5465	272.71	0.0200	HOMO–5→LUMO (–0.30065)<br>HOMO–4→LUMO (–0.31951)<br>HOMO–2→LUMO+1 (0.14273)<br>HOMO–1→LUMO+2 (0.26697)<br>HOMO→LUMO+3 (0.27639)<br>HOMO→LUMO+5 (0.35098)
$S_0 \rightarrow S_6$	4.5661	271.53	0.0005	HOMO–2→LUMO (0.63243)<br>HOMO→LUMO+2 (–0.28503)
$S_0 \rightarrow S_7$	4.6035	269.33	0.0041	HOMO–4→LUMO (0.42902)<br>HOMO→LUMO+3 (0.54588)
$S_0 \rightarrow S_8$	4.6491	266.68	0.4431	HOMO–3→LUMO (0.50923)<br>HOMO–1→LUMO (0.24255)<br>HOMO→LUMO+1 (0.35364)<br>HOMO→LUMO+4 (–0.16833)
$S_0 \rightarrow S_9$	4.7228	262.52	0.0015	HOMO–6→LUMO (0.41510)<br>HOMO–3→LUMO+1 (–0.13181)<br>HOMO–1→LUMO+1 (0.37383)<br>HOMO–1→LUMO+4 (0.10780)<br>HOMO→LUMO+6 (–0.35322)
$S_0 \rightarrow S_{10}$	4.8137	257.57	0.0747	HOMO–3→LUMO (0.28744)<br>HOMO–1→LUMO (–0.11818)<br>HOMO→LUMO+4 (0.61640)

Table S19. Electronic transitions for **2diols-[1,1][2]PCP** determined by TD-DFT methods at B3LYP/ TZVP level.

ExcitedState	Energy(eV)	Wavelength(nm)	f	Transitions
$S_0 \rightarrow S_1$	3.1907	388.58	0.0002	HOMO→LUMO (0.70613)
$S_0 \rightarrow S_2$	4.0332	307.41	0.0000	HOMO–2→LUMO (0.32239)<br>HOMO→LUMO+2 (0.61798)
$S_0 \rightarrow S_3$	4.1079	301.82	0.0117	HOMO–1→LUMO (0.53143)<br>HOMO→LUMO+1 (–0.45245)
$S_0 \rightarrow S_4$	4.2265	293.35	0.1258	HOMO–3→LUMO (0.41593)<br>HOMO–1→LUMO (–0.34656)<br>HOMO→LUMO+1 (–0.33771)<br>HOMO→LUMO+4 (0.26827)
$S_0 \rightarrow S_5$	4.4748	277.07	0.0000	HOMO–2→LUMO (0.61683)<br>HOMO→LUMO+2 (–0.32851)
$S_0 \rightarrow S_6$	4.5112	274.84	0.0167	HOMO–5→LUMO (0.34156)<br>HOMO–4→LUMO (–0.30921)<br>HOMO–2→LUMO+1 (0.14283)<br>HOMO–1→LUMO+2 (0.25611)<br>HOMO→LUMO+3 (–0.26304)<br>HOMO→LUMO+5 (0.34807)
$S_0 \rightarrow S_7$	4.5604	271.87	0.0048	HOMO–4→LUMO (–0.41934)<br>HOMO→LUMO+3 (0.55396)
$S_0 \rightarrow S_8$	4.5969	269.71	0.5006	HOMO–3→LUMO (0.47323)<br>HOMO–1→LUMO (0.26671)<br>HOMO→LUMO+1 (0.40738)<br>HOMO→LUMO+4 (0.12280)
$S_0 \rightarrow S_9$	4.7185	262.76	0.0035	HOMO–6→LUMO (0.49397)<br>HOMO–3→LUMO+1 (–0.12654)

				HOMO–1→LUMO+1 (0.29745)<br>HOMO–1→LUMO+4 (–0.13277)<br>HOMO→LUMO+7 (–0.33317)
$S_0 \rightarrow S_{10}$	4.7858	259.07	0.0633	HOMO–3→LUMO (–0.27418)<br>HOMO–1→LUMO (0.11560)<br>HOMO→LUMO+4 (0.62403)

Table S20. Electronic transitions for **2diols-[1,1][3]PCP** determined by TD-DFT methods at B3LYP/ TZVP level.

ExcitedState	Energy(eV)	Wavelength(nm)	f	Transitions
$S_0 \rightarrow S_1$	3.4627	358.06	0.0002	HOMO→LUMO (0.70176)
$S_0 \rightarrow S_2$	4.0437	306.61	0.0920	HOMO–1→LUMO (0.60808)<br>HOMO→LUMO+1 (0.35597)
$S_0 \rightarrow S_3$	4.2052	294.84	0.9168	HOMO–4→LUMO (–0.11008)<br>HOMO–1→LUMO (–0.33543)<br>HOMO→LUMO+1 (0.57201)<br>HOMO→LUMO+4 (–0.13583)
$S_0 \rightarrow S_4$	4.3119	287.54	0.0875	HOMO–2→LUMO (0.12716)<br>HOMO→LUMO+2 (0.68716)
$S_0 \rightarrow S_5$	4.3341	286.07	0.1681	HOMO–2→LUMO (0.68496)<br>HOMO→LUMO+2 (–0.11533)
$S_0 \rightarrow S_6$	4.3594	284.41	0.0219	HOMO–4→LUMO+2 (0.10676)<br>HOMO–3→LUMO (0.30670)<br>HOMO–2→LUMO+4 (–0.12150)<br>HOMO→LUMO+3 (0.57441)
$S_0 \rightarrow S_7$	4.4637	277.76	0.2550	HOMO–4→LUMO (0.34843)<br>HOMO–2→LUMO+3 (–0.12106)<br>HOMO→LUMO+1 (0.18189)<br>HOMO→LUMO+4 (0.52165)
$S_0 \rightarrow S_8$	4.5100	274.91	0.0000	HOMO–1→LUMO+1 (0.66547)<br>HOMO→LUMO+5 (0.10964)
$S_0 \rightarrow S_9$	4.5442	272.84	0.0055	HOMO–6→LUMO (0.34640)<br>HOMO–5→LUMO+1 (0.15510)<br>HOMO–1→LUMO+1 (–0.14836)<br>HOMO–1→LUMO+3 (0.13989)<br>HOMO–1→LUMO+6 (–0.17460)<br>HOMO→LUMO+5 (0.51248)
$S_0 \rightarrow S_{10}$	4.5765	270.91	0.0000	HOMO–8→LUMO (0.12967)<br>HOMO–6→LUMO+1 (0.17143)<br>HOMO–5→LUMO (0.35477)<br>HOMO–3→LUMO (0.18529)<br>HOMO–1→LUMO+5 (–0.20526)<br>HOMO→LUMO+6 (0.46474)

Table S21. Electronic transitions for **2ketones-[1,1][2]PCP** determined by TD-DFT methods at B3LYP/ TZVP level.

ExcitedState	Energy(eV)	Wavelength(nm)	f	Transitions
$S_0 \rightarrow S_1$	3.4965	354.59	0.0002	HOMO→LUMO (0.70467)
$S_0 \rightarrow S_2$	3.7136	333.87	0.0883	HOMO-3→LUMO+1 (–0.14851)<br>HOMO-2→LUMO+2 (0.19458)

				HOMO→LUMO+1 (0.63602)
$S_0→S_3$	3.7939	326.80	0.0000	HOMO-3→LUMO+2 (–0.16464)<br>HOMO-2→LUMO+1 (0.24733)<br>HOMO→LUMO+2 (0.61403)
$S_0→S_4$	4.0968	302.64	0.0129	HOMO-6→LUMO+2 (–0.16619)<br>HOMO-3→LUMO+1 (0.45333)<br>HOMO-3→LUMO+5 (–0.15821)<br>HOMO-2→LUMO+2 (–0.35095)<br>HOMO-2→LUMO+3 (–0.13145)<br>HOMO→LUMO+1 (0.23678)
$S_0→S_5$	4.0993	302.45	0.0000	HOMO-7→LUMO+1 (–0.11382)<br>HOMO-6→LUMO+1 (0.15782)<br>HOMO-4→LUMO+2 (–0.10668)<br>HOMO-3→LUMO+1 (–0.35720)<br>HOMO-3→LUMO+2 (–0.14547)<br>HOMO-2→LUMO+1 (0.40866)<br>HOMO-2→LUMO+3 (–0.11928)<br>HOMO→LUMO+2 (–0.30978)
$S_0→S_6$	4.3238	286.75	0.0003	HOMO-2→LUMO+2 (0.68350)
$S_0→S_7$	4.3432	285.47	0.1282	HOMO-5→LUMO+1 (0.18130)<br>HOMO-2→LUMO+2 (0.63303)<br>HOMO→LUMO+4 (0.20700)
$S_0→S_8$	4.4256	280.15	0.0000	HOMO-4→LUMO+1 (–0.10511)<br>HOMO-2→LUMO+1 (0.59435)<br>HOMO-2→LUMO+3 (–0.29272)<br>HOMO→LUMO+2 (0.11384)<br>HOMO→LUMO+5 (0.11067)
$S_0→S_9$	4.4698	277.38	0.0003	HOMO-6→LUMO+2 (0.27568)<br>HOMO-4→LUMO+1 (0.14780)<br>HOMO-2→LUMO+1 (0.32788)<br>HOMO-2→LUMO+3 (0.42350)<br>HOMO→LUMO+3 (–0.28067)<br>HOMO→LUMO+5 (–0.10702)
$S_0→S_{10}$	4.4924	275.99	0.0009	HOMO-3→LUMO+1 (0.68639)

Table S22. Electronic transitions for **2ketones-[1,1][3]PCP** determined by TD-DFT methods at B3LYP/ TZVP level.

ExcitedState	Energy(eV)	Wavelength(nm)	f	Transitions
$S_0→S_1$	3.6568	339.05	0.3323	HOMO–3→LUMO (–0.17093)<br>HOMO–2→LUMO+2 (0.14483)<br>HOMO–1→LUMO+1 (0.10819)<br>HOMO→LUMO (0.64200)
$S_0→S_2$	3.6636	338.43	0.0012	HOMO–1→LUMO (0.56674)<br>HOMO→LUMO+1 (–0.41173)
$S_0→S_3$	3.8136	325.11	0.0003	HOMO–1→LUMO (0.40990)<br>HOMO→LUMO+1 (0.56910)
$S_0→S_4$	3.8203	324.54	0.0000	HOMO–9→LUMO (0.13332)<br>HOMO–4→LUMO (0.31595)<br>HOMO–4→LUMO+8 (0.10423)<br>HOMO–3→LUMO+2 (–0.36856)<br>HOMO–2→LUMO (0.37344)<br>HOMO–2→LUMO+8 (0.10259)<br>HOMO→LUMO+2 (0.21667)

$S_0 \rightarrow S_5$	3.8979	318.08	0.0526	HOMO–9→LUMO+2 (–0.12810)<br>HOMO–4→LUMO+2 (–0.27260)<br>HOMO–3→LUMO (0.47645)<br>HOMO–3→LUMO+8 (0.13910)<br>HOMO–2→LUMO+2 (–0.24255)<br>HOMO→LUMO (0.25467)
$S_0 \rightarrow S_6$	4.1361	299.76	0.7206	HOMO–1→LUMO+1 (0.68905)<br>HOMO→LUMO (–0.12234)
$S_0 \rightarrow S_7$	4.1402	299.46	0.0000	HOMO–4→LUMO (–0.12520)<br>HOMO–3→LUMO+1 (0.11928)<br>HOMO–2→LUMO (–0.12960)<br>HOMO→LUMO+1 (0.66084)
$S_0 \rightarrow S_8$	4.2203	293.78	0.0529	HOMO–7→LUMO (–0.11508)<br>HOMO–2→LUMO+1 (0.17300)<br>HOMO–1→LUMO+2 (0.65415)<br>HOMO→LUMO+3 (0.10312)
$S_0 \rightarrow S_9$	4.4348	279.57	0.0000	HOMO–4→LUMO (–0.42982)<br>HOMO–3→LUMO+1 (0.13011)<br>HOMO–2→LUMO (0.50524)<br>HOMO–1→LUMO+6 (0.12867)
$S_0 \rightarrow S_{10}$	4.4732	277.17	0.0000	HOMO–10→LUMO (0.11180)<br>HOMO–6→LUMO (0.27411)<br>HOMO–5→LUMO+1 (–0.22832)<br>HOMO–1→LUMO+5 (–0.23177)<br>HOMO→LUMO+4 (0.49828)<br>HOMO→LUMO+7 (0.11745)

Table S23. Electronic transitions for **2ketones-[1,1][4]PCP** determined by TD-DFT methods at B3LYP/ TZVP level.

ExcitedState	Energy(eV)	Wavelength(nm)	f	Transitions
$S_0 \rightarrow S_1$	3.3189	373.57	0.0008	HOMO–2→LUMO+1 (–0.17081)<br>HOMO–0→LUMO (0.66677)
$S_0 \rightarrow S_2$	3.5904	345.32	0.2204	HOMO–5→LUMO (–0.23607)<br>HOMO–4→LUMO+1 (–0.27197)<br>HOMO–2→LUMO (–0.30604)<br>HOMO–0→LUMO+1 (0.47679)
$S_0 \rightarrow S_3$	3.6699	337.84	0.5002	HOMO–5→LUMO+1 (0.15681)<br>HOMO–4→LUMO (0.18120)<br>HOMO–1→LUMO (0.64049)
$S_0 \rightarrow S_4$	3.7319	332.23	0.1523	HOMO–5→LUMO+1 (0.33836)<br>HOMO–4→LUMO (0.40715)<br>HOMO–4→LUMO+3 (0.13459)<br>HOMO–2→LUMO+1 (0.19452)<br>HOMO–1→LUMO (–0.28072)<br>HOMO–0→LUMO (0.16038)
$S_0 \rightarrow S_5$	3.7844	327.62	0.0810	HOMO–5→LUMO (0.28521)<br>HOMO–5→LUMO+3 (0.10141)<br>HOMO–4→LUMO+1 (0.29388)<br>HOMO–2→LUMO (0.17424)<br>HOMO–0→LUMO+1 (0.50342)
$S_0 \rightarrow S_6$	3.9175	316.49	0.0116	HOMO–1→LUMO+1 (0.68911)
$S_0 \rightarrow S_7$	4.0471	306.35	0.1680	HOMO–5→LUMO (–0.25485)<br>HOMO–4→LUMO+1 (–0.16839)<br>HOMO–2→LUMO (0.59853)

				HOMO–0→LUMO+1 (0.10454)
$S_0 \rightarrow S_8$	4.0494	306.18	0.8300	HOMO–1→LUMO+2 (–0.12879)<br>HOMO–0→LUMO+2 (0.68488)
$S_0 \rightarrow S_9$	4.1653	297.66	0.0476	HOMO–5→LUMO+1 (0.10235)<br>HOMO–2→LUMO+1 (–0.36206)<br>HOMO–1→LUMO+2 (0.54623)<br>HOMO–0→LUMO (–0.14921)<br>HOMO–0→LUMO+2 (0.13403)
$S_0 \rightarrow S_{10}$	4.2587	291.13	0.0129	HOMO–5→LUMO+1 (–0.13856)<br>HOMO–4→LUMO (–0.13917)<br>HOMO–2→LUMO+1 (0.50885)<br>HOMO–1→LUMO+2 (0.39498)<br>HOMO–0→LUMO+3 (–0.10130)

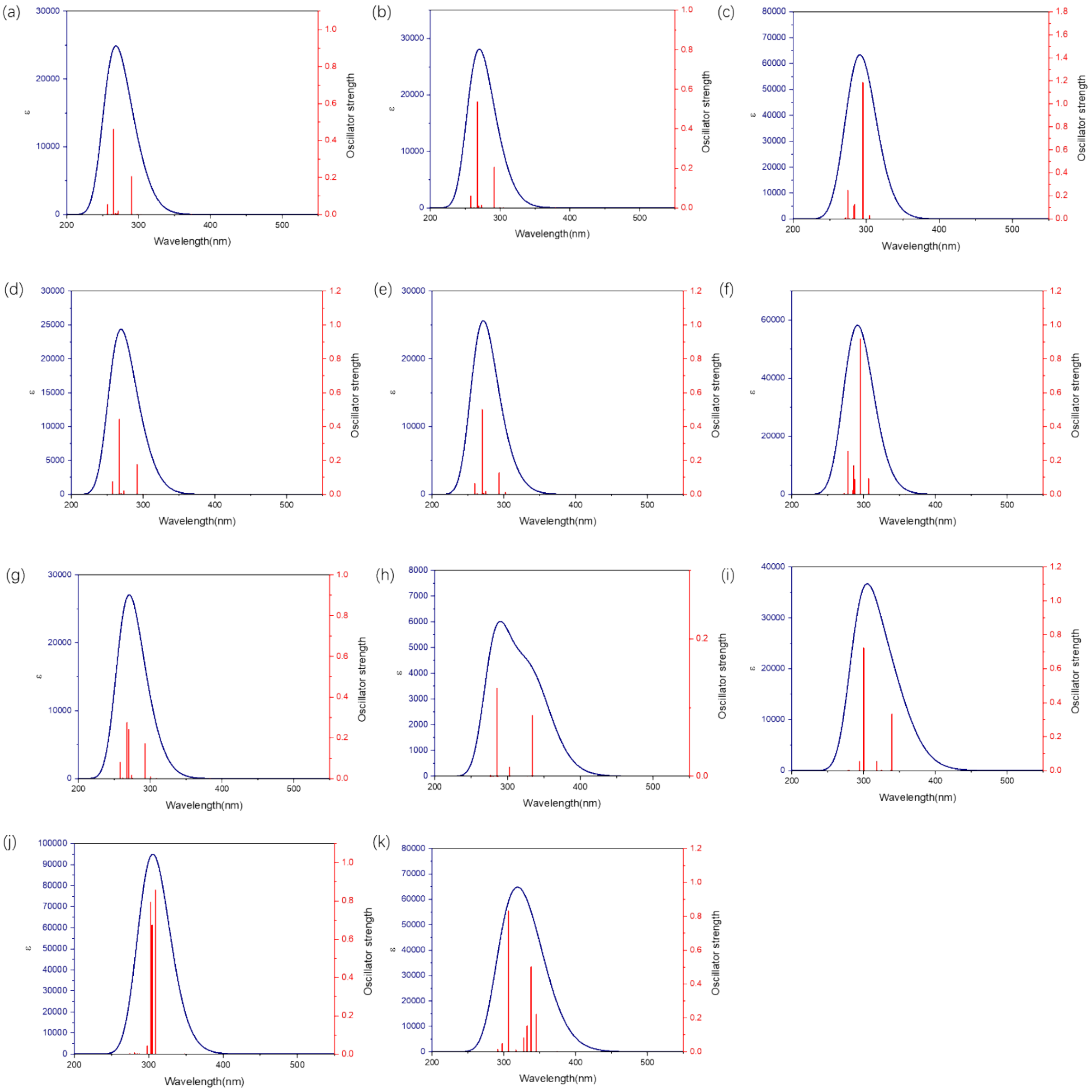

Figure S90. Calculated electronic absorption spectrum (B3LYP/TZVP) of (a) **Ketal-2Me-[1,1][2]PCP**, (b) **2ketals-[1,1][2]PCP**, (c) **2ketals-[1,1][3]PCP**, (d) **diol-2Me-[1,1][2]PCP**, (e)

2diols-[1,1][2]PCP, (f) **2diols-[1,1][3]PCP**, (g) **2dbts-[1,1][2]PCP**, (h) **2ketones-[1,1][2]PCP**, (i) **2ketones-[1,1][3]PCP**, (j) **2ketals-[1,1][4]PCP** and (k) **2ketones-[1,1][4]PCP**.

7. Photophysical Measurements

Table S24. Photophysical properties of the compounds in the solution and solid states.

molecule	$\lambda_{abs.}$ (nm)	$\lambda_{PL,s}$ (nm)	$\lambda_{PL,sl}$ (nm)	$\Phi_{PL,s}$ (%)	$\Phi_{PL,sl}$ (%)	FLT_s (ns)	FLT_{sl} (ns)
**2dbts-[1,1][2]PCP**	274	491	488.5	39.79	4.63	41.59	18.37
**ketal-2Me-[1,1][2]PCP**	272	491	499.5	39.71	4.58	36.51	13.87
**2ketals-[1,1][2]PCP**	272	490.5	500	37.12	4.48	37.59	14.63
**2ketals-[1,1][3]PCP**	293	429.5	423.5	73.68	36.72	9.60	6.08
**2ketals-[1,1][4]PCP**	312	407	406	90.42	58.74	1.99	0.50
**diol-2Me-[1,1][2]PCP**	273	503.5	491.5	10.13	12.73	37.73	13.87
**2diols-[1,1][2]PCP**	274	501	491.5	17.74	6.92	54.62	14.69
**2diols-[1,1][3]PCP**	280	425.5	420.5	20.55	38.23	6.83	5.85
**2ketones-[1,1][3]PCP**	-	474	-	6.72	-	8.88	-
**2ketones-[1,1][4]PCP**	317	-	-	-	-	-	-
**Compound 1**	298	-	-	-	-	-	-

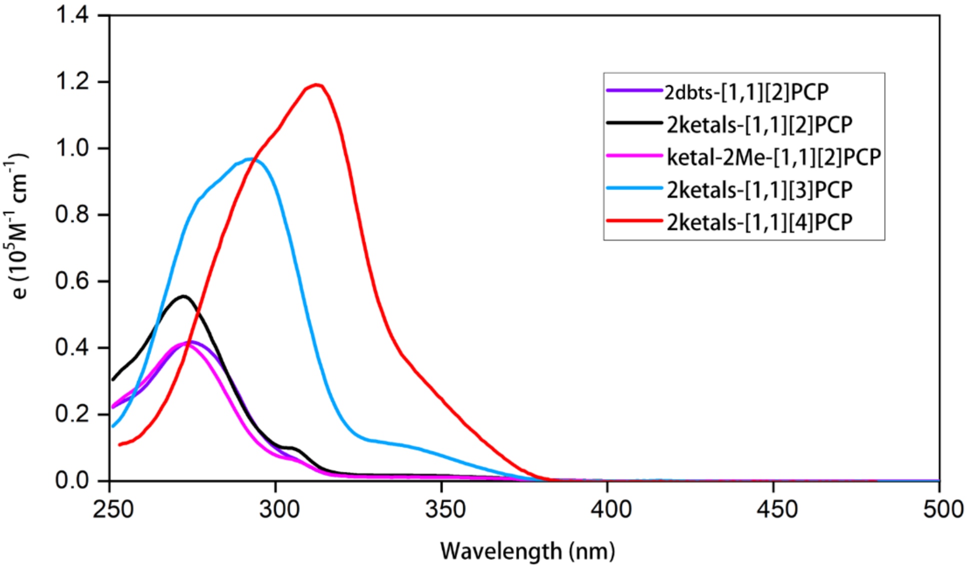

Figure S91. UV–vis absorption spectra of **2dbts-[1,1][2]PCP**, **ketal-2Me-[1,1][2]PCP**, **2ketals-[1,1][2]PCP**, **2ketals-[1,1][3]PCP**, and **2ketals-[1,1][4]PCP** in THF.

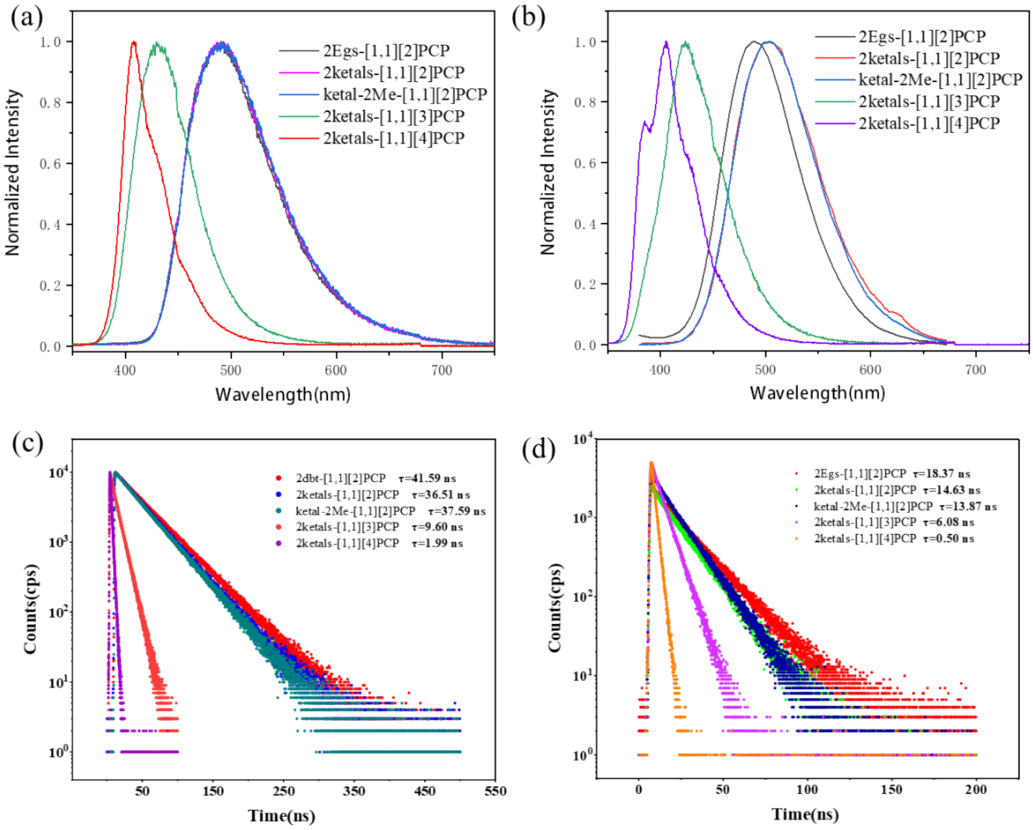

Figure S92. (a) Solid-state photoluminescence spectra; (b) photoluminescence spectra in tetrahydrofuran (THF) at a concentration of 1.0×10^{-4} mol·L^{-1}; (c) solid-state fluorescence decay curves; and (d) fluorescence decay curves in THF (1.0×10^{-4} mol·L^{-1}) of **2dbts-[1,1][2]PCP, ketal-2Me-[1,1][2]PCP, 2ketals-[1,1][2]PCP, 2ketals-[1,1][3]PCP, and 2ketals-[1,1][4]PCP.**

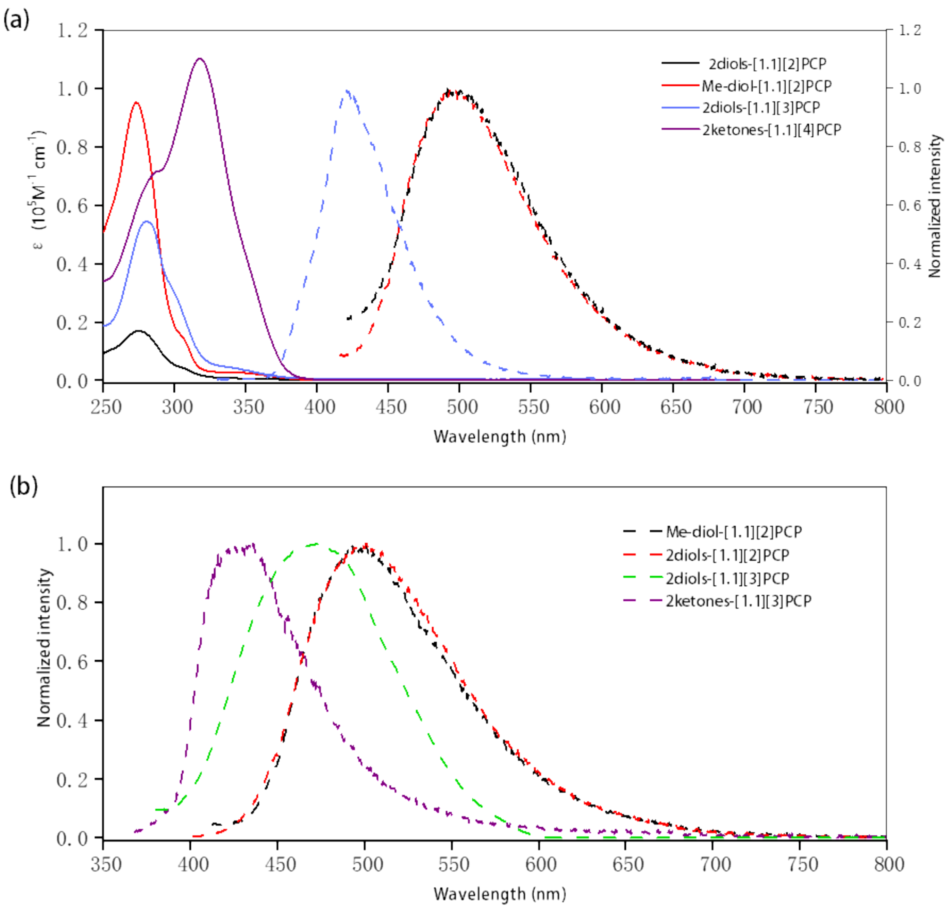

Figure S93. (a) UV/Vis absorption (solid line) and fluorescence spectra (dashed line) of **2Me-diol-[1,1][n]PCP**, **2diols-[1,1][2]PCP** and **2diols-[1,1][3]PCP** in THF. (b) PL spectra of **2Me-diol-[1,1][2]PCP**, **2diols-[1,1][2]PCP**, **2diols-[1,1][3]PCP** and **2ketones-[1,1][3]PCP** in solid state.

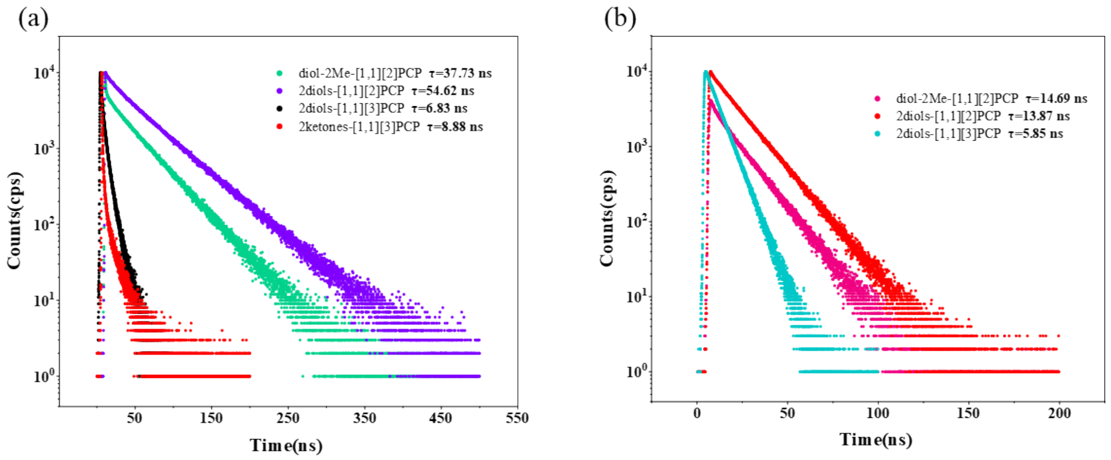

Figure 94. (a) solid-state fluorescence decay curves of **2Me-diol-[1,1][n]PCP**, **2diols-[1,1][2]PCP**, **2diols-[1,1][3]PCP and 2ketones-[1,1][3]PCP**; and (b) fluorescence decay curves in THF (1.0×10^{-4} mol·L^{-1}) of **2Me-diol-[1,1][n]PCP**, **2diols-[1,1][2]PCP**, **2diols-**

[1,1][3]PCP.

Cartesian coordinates of optimized structures

2ketals-[1,1][2]PCP			
O	-5.059699	0.217731	-1.13953
O	-5.059807	-0.21761	1.13952
O	5.059782	0.217775	1.139524
O	5.059721	-0.21786	-1.13954
C	-4.281665	0.000066	0.000029
C	-3.300798	1.179145	0.143259
C	-2.523031	1.229129	1.297558
H	-2.843489	0.684453	2.179486
C	-1.258602	1.789147	1.267322
H	-0.612684	1.66265	2.1295
C	-2.889368	1.921043	-0.95943
H	-3.511849	1.943653	-1.84622
C	-1.623762	2.497218	-0.98394
H	-1.286138	2.988934	-1.8912
C	-0.739072	2.327583	0.08464
C	0.739164	2.327537	-0.08486
C	1.623865	2.497268	0.983701
H	1.286265	2.989123	1.890894
C	2.889449	1.921046	0.959255
H	3.511931	1.943757	1.846044
C	1.258673	1.78892	-1.26747
H	0.612752	1.662331	-2.12963
C	2.523085	1.228853	-1.29764
H	2.843523	0.684059	-2.1795
C	3.300865	1.179017	-0.14335
C	4.281707	-4.9E-05	0.000004
C	3.300826	-1.17908	0.14339
C	2.523055	-1.22887	1.297692
H	2.843525	-0.68408	2.179543
C	1.258627	-1.78891	1.267551
H	0.612719	-1.66229	2.129719
C	2.889375	-1.92112	-0.95919
H	3.511848	-1.94387	-1.84599
C	1.623773	-2.4973	-0.98362
H	1.286145	-2.98916	-1.8908
C	0.739092	-2.32753	0.084959
C	-0.739142	-2.32755	-0.08453
C	-1.623825	-2.49712	0.984075
H	-1.286202	-2.98882	1.891345
C	-2.889413	-1.92092	0.959552
H	-3.511881	-1.94348	1.846354
C	-1.258679	-1.78915	-1.26722
H	-0.612771	-1.6627	-2.12942
C	-2.523098	-1.22911	-1.29747
H	-2.843562	-0.68448	-2.17942
C	-3.300839	-1.17905	-0.14316
C	-5.930901	-0.85073	-1.48562
H	-6.687622	-0.97996	-0.70368
H	-5.362271	-1.78597	-1.54978
C	-6.571259	-0.50943	-2.8121
H	-5.813144	-0.40617	-3.59192
H	-7.12189	0.431645	-2.74373
H	-7.268481	-1.29586	-3.11171
C	-5.931045	0.850833	1.485575
H	-6.687359	0.980448	0.703307
H	-5.362339	1.785988	1.550377
C	-6.572104	0.509108	2.811602
H	-5.814366	0.405273	3.591721
H	-7.122974	-0.43178	2.742563
H	-7.26923	1.295601	3.111264
C	5.930796	-0.85075	1.485911
H	6.687046	-0.98084	0.703665
H	5.36186	-1.78574	1.551074
C	6.571989	-0.50871	2.811792
H	7.268898	-1.29529	3.111732
H	5.814296	-0.40437	3.591887
H	7.123124	0.431996	2.742389
C	5.93073	0.850665	-1.48593
H	6.687176	0.980547	-0.70384
H	5.361847	1.785709	-1.55079
C	6.571599	0.508838	-2.81203
H	7.12252	-0.43202	-2.74297
H	7.268654	1.295326	-3.11187
H	5.81375	0.404892	-3.59202

2ketals-[1,1][3]PCP			
O	-6.96535	0.016345	1.160068
C	-6.18373	0.000086	-4E-06
O	-6.96541	-0.01618	-1.16005
C	-5.22531	1.197884	0.060293
C	-4.4753	1.494586	-1.07512
H	-4.75482	1.055568	-2.02722
C	-3.30245	2.222147	-0.97376
H	-2.67387	2.336912	-1.85091
C	-4.86808	1.787341	1.267589
H	-5.47177	1.601953	2.14788
C	-3.69083	2.519155	1.367994

H	-3.39312	2.916865	2.333425
C	-2.84451	2.673065	0.268013
C	-1.41341	3.038252	0.417148
C	-0.69421	3.725122	-0.5651
H	-1.22623	4.227171	-1.36757
C	0.694317	3.725109	-0.5651
H	1.226354	4.22715	-1.36756
C	-0.6921	2.467974	1.468299
H	-1.21811	1.911268	2.236668
C	0.692174	2.467963	1.468304
H	1.218169	1.911251	2.236681
C	1.413504	3.038225	0.417157
C	2.844596	2.67301	0.268029
C	3.302534	2.222082	-0.97374
H	2.67396	2.336869	-1.8509
C	4.475365	1.49449	-1.0751
H	4.754881	1.055466	-2.0272
C	3.690902	2.519069	1.368014
H	3.393193	2.916785	2.333444
C	4.868129	1.787221	1.267615
H	5.47181	1.601813	2.147907
C	5.225353	1.197761	0.06032
C	-7.84626	1.089054	-1.31212
H	-7.28345	2.026561	-1.23521
H	-8.5886	1.085087	-0.5062
C	-8.51007	0.961923	-2.66467
H	-7.76622	0.990784	-3.46431
H	-9.21539	1.781956	-2.82095
H	-9.05606	0.01862	-2.73938
C	-7.84637	-1.08877	1.312034
H	-7.28387	-2.02636	1.233937
H	-8.58939	-1.08398	0.506743
C	-8.50902	-0.96251	2.665235
H	-9.05461	-0.01907	2.741166
H	-7.76454	-0.9923	3.464234
H	-9.21453	-1.7824	2.821418
O	6.965378	-0.01635	-1.16004
C	6.183735	-8.2E-05	0.000018
O	6.965383	0.016182	1.160081
C	5.225306	-1.19788	-0.0603
C	4.475292	-1.49459	1.075112
H	4.754808	-1.05557	2.027214
C	3.302443	-2.22215	0.973734
H	2.673856	-2.33692	1.850884
C	4.868081	-1.78733	-1.2676
H	5.471777	-1.60193	-2.14788
C	3.690835	-2.51914	-1.36801
H	3.393128	-2.91685	-2.33345
C	2.84451	-2.67306	-0.26804
C	1.413414	-3.03825	-0.41718
C	0.694208	-3.72512	0.565071
H	1.22623	-4.22716	1.367538
C	-0.69432	-3.72511	0.565065
H	-1.22636	-4.22714	1.367526
C	0.692102	-2.46798	-1.46834
H	1.218113	-1.91127	-2.23671
C	-0.69217	-2.46797	-1.46834
H	-1.21817	-1.91126	-2.23672
C	-1.4135	-3.03822	-0.41719
C	-2.8446	-2.673	-0.26806
C	-3.30253	-2.22209	0.973715
H	-2.67395	-2.33689	1.850869
C	-4.47536	-1.4945	1.075088
H	-4.75487	-1.05549	2.027189
C	-3.6909	-2.51905	-1.36804
H	-3.3932	-2.91676	-2.33348
C	-4.86813	-1.7872	-1.26763
H	-5.47181	-1.60178	-2.14792
C	-5.22535	-1.19775	-0.06033
C	7.846226	-1.08906	1.31217
H	7.283415	-2.02656	1.235222
H	8.588604	-1.08508	0.506288
C	8.509974	-0.96195	2.664754
H	7.766095	-0.99083	3.464354
H	9.215292	-1.78199	2.821051
H	9.055963	-0.01865	2.739508
C	7.846412	1.088758	-1.31198
H	7.283921	2.026358	-1.23391
H	8.589408	1.083965	-0.50667
C	8.509101	0.962479	-2.66517
H	9.054681	0.019032	-2.74107
H	7.764638	0.992274	-3.46419
H	9.214616	1.782369	-2.82133

2ketals-[1,1][4]PCP

O	-8.77966	-0.0506	1.229312
O	-8.87501	0.039011	-1.08767
C	-8.04404	-0.00643	0.037585
C	-7.09856	1.198649	-0.02732
C	-6.36725	1.522909	1.112226
H	-6.61103	1.047934	2.056317
C	-5.27356	2.364565	1.033833
H	-4.66564	2.519416	1.91871
C	-6.78214	1.830639	-1.22383

H	-7.36685	1.614094	-2.10981
C	-5.68539	2.682622	-1.30017
H	-5.4355	3.138832	-2.25312
C	-4.87402	2.915465	-0.18796
C	-3.5303	3.538413	-0.28908
C	-2.95886	4.234394	0.780807
H	-3.57633	4.524446	1.625051
C	-1.60054	4.511323	0.808866
H	-1.1698	5.000006	1.677505
C	-2.71666	3.260572	-1.38992
H	-3.12382	2.711899	-2.23284
C	-1.36132	3.55433	-1.37083
H	-0.73507	3.239031	-2.19959
C	-0.76818	4.121718	-0.24261
C	0.708252	4.081371	-0.09728
C	1.237633	3.404316	1.002381
H	0.567029	3.034794	1.771546
C	2.583504	3.089783	1.066405
H	2.932174	2.467192	1.882621
C	1.594843	4.523113	-1.08038
H	1.216399	5.084503	-1.92932
C	2.950342	4.229171	-1.00171
H	3.611015	4.582528	-1.78698
C	3.464947	3.45951	0.046628
C	4.833535	2.88198	0.027687
C	5.474432	2.495821	1.209733
H	5.075763	2.808924	2.168878
C	6.577761	1.659363	1.183192
H	7.00092	1.294394	2.113263
C	5.445118	2.532613	-1.17869
H	4.986769	2.819362	-2.11936
C	6.574402	1.726067	-1.20686
H	7.000125	1.42318	-2.15579
C	7.105734	1.218511	-0.02698
C	-9.63785	1.05997	1.460821
H	-10.3179	1.186981	0.611687
H	-9.04627	1.97851	1.546247
C	-10.4024	0.784451	2.735929
H	-11.0855	1.608725	2.955739
H	-9.71807	0.670832	3.579909
H	-10.9866	-0.13415	2.644674
C	-9.82013	-1.0189	-1.18982
H	-10.5983	-0.89464	-0.42835
H	-9.32972	-1.98039	-1.00161
C	-10.4103	-0.9722	-2.58125
H	-11.172	-1.74717	-2.6981
H	-9.63672	-1.13398	-3.3357
H	-10.8746	-0.00161	-2.77057
O	8.779712	0.050652	-1.22924
O	8.874955	-0.03904	1.087727
C	8.044026	0.006426	-0.03755
C	7.098553	-1.19866	0.027292
C	6.3672	-1.52284	-1.11225
H	6.610953	-1.04781	-2.05632
C	5.273513	-2.3645	-1.03388
H	4.665564	-2.5193	-1.91875
C	6.782155	-1.83072	1.223775
H	7.366882	-1.61423	2.109748
C	5.685403	-2.6827	1.300086
H	5.435528	-3.13897	2.253017
C	4.873996	-2.91548	0.187881
C	3.53028	-3.53843	0.289005
C	2.95883	-4.23437	-0.7809
H	3.576285	-4.5244	-1.62516
C	1.600507	-4.51131	-0.80895
H	1.169755	-4.99996	-1.6776
C	2.716654	-3.26062	1.389859
H	3.123829	-2.71197	2.232794
C	1.361319	-3.55438	1.37078
H	0.735074	-3.2391	2.19955
C	0.768166	-4.12173	0.242548
C	-0.70827	-4.08139	0.097233
C	-1.23767	-3.40433	-1.00242
H	-0.56708	-3.03481	-1.77159
C	-2.58354	-3.0898	-1.06642
H	-2.93222	-2.46721	-1.88264
C	-1.59485	-4.52313	1.080342
H	-1.21639	-5.08452	1.929276
C	-2.95035	-4.22919	1.00169
H	-3.61101	-4.58255	1.78697
C	-3.46497	-3.45953	-0.04664
C	-4.83356	-2.882	-0.02767
C	-5.47449	-2.49586	-1.20971
H	-5.07585	-2.80899	-2.16886
C	-6.57781	-1.6594	-1.18316
H	-7.001	-1.29446	-2.11323
C	-5.44511	-2.5326	1.178709
H	-4.98673	-2.81932	2.119373
C	-6.57439	-1.72604	1.206892
H	-7.00008	-1.42313	2.155825
C	-7.10575	-1.21852	0.027016
C	9.638074	-1.05981	-1.46067
H	10.3182	-1.18664	-0.61153
H	9.046646	-1.97845	-1.54601
C	10.4026	-0.78427	-2.7358
H	11.08582	-1.60845	-2.95554

H	9.718225	-0.67085	-3.57979
H	10.98664	0.134436	-2.64463
C	9.820109	1.018847	1.189897
H	10.59823	0.89456	0.428427
H	9.329732	1.980356	1.001687
C	10.41029	0.972119	2.581329
H	11.17198	1.747065	2.698197
H	9.636681	1.133915	3.335775
H	10.87454	0.00151	2.770649

2dbts-[1,1][2]PCP

C	-2.60308	1.100335	-1.2866
C	-1.37977	1.746587	-1.24923
C	-0.88801	2.291172	-0.05683
C	-1.77363	2.373056	1.021921
C	-2.99021	1.700804	0.992641
C	-3.36086	0.962225	-0.12695
C	-2.75071	-2.09237	-0.94508
C	-1.45485	-2.59521	-0.96849
C	-0.58823	-2.3891	0.108739
C	-1.14827	-1.90517	1.296819
C	-2.44468	-1.42235	1.32969
C	-3.21263	-1.39821	0.168914
C	-4.24823	-0.29532	0.015307
C	0.888022	-2.29116	-0.05679
C	0.588239	2.389126	0.108724
C	1.379814	-1.74664	-1.2492
C	2.603118	-1.10039	-1.28657
C	3.360864	-0.96222	-0.12692
C	2.990206	-1.70076	0.992697
C	1.773626	-2.37301	1.021977
C	1.454879	2.595242	-0.96849
C	2.750737	2.092409	-0.94506
C	3.212633	1.39824	0.168936
C	2.444682	1.422395	1.329708
C	1.14827	1.905214	1.296815
C	4.248228	0.295346	0.015307
H	-2.89622	0.561024	-2.18119
H	-0.73495	1.686648	-2.11922
H	-1.46593	2.866684	1.938481
H	-3.59533	1.643695	1.891622
H	-3.36397	-2.13733	-1.83794
H	-1.08516	-3.05292	-1.88078
H	-0.51943	-1.75673	2.167809
H	-2.80614	-0.92333	2.221911
H	0.735008	-1.68674	-2.11922
H	2.896274	-0.56111	-2.18118
H	3.595305	-1.64362	1.891691
H	1.465904	-2.86661	1.938546
H	1.085195	3.052959	-1.88079
H	3.364013	2.137369	-1.83791
H	2.806129	0.923379	2.221936
H	0.519414	1.756779	2.167797
O	5.068706	0.507601	-1.09997
O	5.115331	0.211056	1.115555
O	-5.06872	-0.50759	-1.09997
O	-5.11529	-0.21101	1.115571
C	6.191518	-0.33002	-0.89132
C	6.27754	-0.45599	0.64691
C	-6.19158	0.329942	-0.8913
C	-6.27763	0.455848	0.646933
H	-6.03189	1.305638	-1.36276
H	-7.05813	-0.15375	-1.34131
H	-7.14508	-0.04827	1.074655
H	-6.27451	1.503754	0.964301
H	7.058104	0.153614	-1.34132
H	6.03176	-1.30569	-1.36281
H	6.274227	-1.50393	0.9642
H	7.145091	0.047919	1.074669

ketal-2Me-[1,1][2]PCP

O	-4.2267	0.058271	1.15863
O	-4.22674	-0.05839	-1.158626
C	-3.44837	-4.1E-05	-0.000016
C	-2.46832	1.180015	-0.140202
C	-1.68761	1.502441	0.967555
H	-2.00488	1.180898	1.954071
C	-0.42426	2.041984	0.802121
H	0.226239	2.125346	1.666476
C	-2.0618	1.640727	-1.388358
H	-2.68697	1.451894	-2.253151
C	-0.79724	2.195607	-1.552231
H	-0.4607	2.456837	-2.550978
C	0.090191	2.284224	-0.476319
C	1.566497	2.23653	-0.649944
C	2.471603	2.68345	0.313944
H	2.153351	3.404385	1.061225
C	3.740722	2.12007	0.416756
H	4.367079	2.41652	1.251265
C	2.069662	1.395182	-1.646891
H	1.41062	1.040195	-2.432106
C	3.334035	0.845655	-1.546169

H	3.610749	0.083449	-2.265514
C	4.145335	1.092736	-0.436656
C	5.15334	0.000029	-0.000006
C	6.063595	-0.44474	-1.150622
H	5.52263	-0.71507	-2.05777
H	6.661884	-1.31035	-0.852305
H	6.748206	0.365629	-1.412594
C	6.06358	0.444869	1.15059
H	6.6618	1.31053	0.852261
H	6.748258	-0.36544	1.412556
H	5.52261	0.715173	2.057745
C	4.145411	-1.09274	0.43666
C	3.334112	-0.8457	1.546193
H	3.610836	-0.08353	2.265567
C	2.069747	-1.39524	1.646896
H	1.410706	-1.04029	2.432128
C	3.740819	-2.12007	-0.416758
H	4.367189	-2.41651	-1.251259
C	2.471699	-2.68346	-0.313966
H	2.153464	-3.40439	-1.061259
C	1.56658	-2.23655	0.649914
C	0.090281	-2.28422	0.476255
C	-0.79717	-2.19567	1.552151
H	-0.46065	-2.45694	2.550895
C	-2.06175	-1.64081	1.388279
H	-2.68693	-1.45203	2.25307
C	-0.42415	-2.04191	-0.802181
H	0.22638	-2.12518	-1.666523
C	-1.68751	-1.50238	-0.967614
H	-2.00475	-1.18078	-1.954121
C	-2.46825	-1.18005	0.140139
C	-5.09819	1.17803	1.241428
H	-5.85526	1.117511	0.451509
H	-4.53	2.101966	1.081314
C	-5.73787	1.161382	2.611332
H	-4.97953	1.247974	3.392966
H	-6.28696	0.23021	2.769224
H	-6.4364	1.995407	2.715415
C	-5.09819	-1.17818	-1.241394
H	-5.85492	-1.11797	-0.451122
H	-4.52987	-2.10214	-1.081828
C	-5.73847	-1.16114	-2.61101
H	-4.98036	-1.24665	-3.392994
H	-6.28835	-0.23031	-2.768116
H	-6.43637	-1.99566	-2.715341

2diols-[1,1][2]PCP

C	3.29247	1.18316	-0.13632
C	2.87599	1.910616	0.975873
C	1.618127	2.500859	0.996339
C	0.739618	2.351374	-0.08159
C	1.265644	1.829039	-1.26806
C	2.526909	1.259489	-1.29679
C	1.618184	-2.50102	-0.99598
C	0.739837	-2.35121	0.082091
C	1.266214	-1.82887	1.268376
C	2.527516	-1.25939	1.296798
C	3.29282	-1.18318	0.136133
C	2.876048	-1.91081	-0.97587
C	4.260036	0.000105	-0.00032
C	-3.2926	-1.18327	-0.13614
C	-2.52725	-1.25993	-1.29669
C	-1.2659	-1.82929	-1.26798
C	-0.73959	-2.35122	-0.08145
C	-1.61798	-2.50055	0.996626
C	-2.87589	-1.91042	0.9762
C	-1.26616	1.829041	1.268399
C	-0.73983	2.351375	0.0821
C	-1.61813	2.500955	-0.99602
C	-2.87598	1.910687	-0.97593
C	-3.29266	1.183061	0.136103
C	-2.52741	1.259407	1.29674
C	-4.26002	-9.9E-05	-0.00031
O	-5.05169	0.206497	-1.13682
O	-5.05217	-0.20672	1.135848
O	5.052215	0.206811	1.135895
O	5.051744	-0.2065	-1.13673
H	3.489241	1.913586	1.869427
H	1.278759	2.984694	1.906995
H	0.627331	1.718287	-2.13782
H	2.850707	0.726413	-2.18417
H	1.27866	-2.9853	-1.90634
H	0.628164	-1.71794	2.138303
H	2.851578	-0.72614	2.183979
H	3.489273	-1.9142	-1.86945
H	-2.85136	-0.72733	-2.18425
H	-0.62779	-1.71858	-2.13787
H	-1.27851	-2.98441	1.907234
H	-3.48913	-1.91347	1.869766
H	-0.62819	1.71835	2.138407
H	-1.2785	2.984927	-1.90652
H	-3.48904	1.914083	-1.8696
H	-2.85147	0.726246	2.183991
H	-5.53574	-0.60731	-1.29968

H	-5.53556	0.607451	1.299101
H	5.535771	-0.60723	1.299155
H	5.534947	0.60772	-1.30025

2diols-[1,1][3]PCP			
C	-1.41735	3.041217	-0.06972
C	-0.69095	3.654446	0.954752
C	0.698573	3.651309	0.946951
C	1.411443	3.031058	-0.08328
C	0.680835	2.521956	-1.15966
C	-0.70209	2.528914	-1.15425
C	0.680867	-2.52189	1.159691
C	1.411471	-3.03116	0.083392
C	0.698634	-3.65159	-0.94675
C	-0.69089	-3.6547	-0.95458
C	-1.41728	-3.04126	0.069762
C	-0.70205	-2.52881	1.154236
C	5.23977	-1.19853	-0.06429
C	4.8151	-1.81204	1.10948
C	3.64345	-2.55545	1.133007
C	2.8502	-2.67614	-0.0098
C	3.373486	-2.19964	-1.21603
C	4.555224	-1.47663	-1.24491
C	3.643358	2.55539	-1.133
C	2.850178	2.676076	0.009855
C	3.373639	2.199742	1.216081
C	4.555416	1.476799	1.24491
C	5.239844	1.198628	0.064218
C	4.815059	1.812054	-1.10954
C	6.189348	0.000036	-5.6E-05
O	6.986687	-0.00801	-1.15236
C	-2.85475	2.685935	0.037391
C	-2.85467	-2.68592	-0.0374
O	6.986768	0.008028	1.152213
C	-3.33796	2.138579	1.228614
C	-4.51333	1.404881	1.244916
C	-5.23626	1.200575	0.072366
C	-3.33776	-2.1384	-1.2286
C	-4.51312	-1.4047	-1.24492
C	-5.23615	-1.20052	-0.07242
C	-4.85582	-1.8905	1.074811
C	-3.68153	-2.63046	1.08778
O	-6.97691	0.139476	-1.1459
C	-4.85584	1.890471	-1.07489
C	-3.68154	2.630396	-1.08784
C	-6.18174	-1.6E-05	-3.2E-05
O	-6.97689	-0.13961	1.145846
H	-1.21729	4.105262	1.790728
H	1.234947	4.102884	1.776028
H	1.197657	2.007996	-1.96272
H	-1.23341	2.022711	-1.95307
H	1.197721	-2.00794	1.962743
H	1.23505	-4.10328	-1.77574
H	-1.21725	-4.10566	-1.79048
H	-1.23341	-2.02252	1.952982
H	5.350522	-1.62975	2.034925
H	3.301577	-2.97691	2.072932
H	2.79149	-2.29352	-2.12727
H	4.893064	-1.03062	-2.1732
H	3.301452	2.97687	-2.07291
H	2.791745	2.293664	2.127382
H	4.893438	1.03094	2.173202
H	5.350471	1.629774	-2.03499
H	7.50726	0.800712	-1.14359
H	7.507282	-0.80072	1.143391
H	-2.72642	2.178616	2.124324
H	-4.81122	0.887406	2.150512
H	-2.72612	-2.17832	-2.12425
H	-4.81091	-0.88712	-2.1505
H	-5.43883	-1.77854	1.981061
H	-3.36468	-3.10877	2.009403
H	-7.45829	-0.68409	-1.26103
H	-5.43881	1.778474	-1.98116
H	-3.36464	3.108672	-2.00947
H	-7.45847	0.68386	1.260874

2ketones-[1,1][3]PCP			
C	1.393197	-3.54585	-0.21444
C	0.86675	-3.65892	1.075333
C	-0.50382	-3.66865	1.285731
C	-1.39332	-3.54581	0.214489
C	-0.86687	-3.65886	-1.07531
C	0.50368	-3.66863	-1.28571
C	-0.86674	3.658927	1.07534
C	-1.3932	3.545856	-0.21443
C	-0.50369	3.668643	-1.28571
C	0.86686	3.658864	-1.07532
C	1.393321	3.545817	0.21448
C	0.503826	3.66866	1.285728
C	-4.92774	1.191042	-0.3008
C	-4.92354	2.341602	0.482777
C	-3.83447	3.211654	0.443936

C	-2.74839	2.962843	-0.3987
C	-2.85182	1.897835	-1.3021
C	-3.90885	1.014709	-1.23739
C	-3.83454	-3.21155	-0.44396
C	-2.74851	-2.96277	0.398744
C	-2.852	-1.89783	1.30222
C	-3.90902	-1.0147	1.237482
C	-4.92784	-1.19097	0.300813
C	-4.92361	-2.3415	-0.48282
C	-5.81068	0.000052	-0.00007
O	-7.01334	0.000071	-0.00019
C	2.748392	-2.96284	-0.39871
C	2.748506	2.962774	0.398731
C	3.834464	-3.21166	0.443923
C	4.923539	-2.34162	0.482775
C	4.927745	-1.19105	-0.30079
C	3.834543	3.211537	-0.44397
C	4.923605	2.341477	-0.48282
C	4.927827	1.190959	0.300823
C	3.909006	1.014704	1.23749
C	2.85199	1.897842	1.302217
C	3.908864	-1.01471	-1.23738
C	2.85183	-1.89783	-1.30211
C	5.810676	-6.1E-05	-3.4E-05
O	7.013343	-5.8E-05	-0.0002
H	1.530037	-3.60851	1.933003
H	-0.8819	-3.66189	2.303079
H	-1.53017	-3.6084	-1.93297
H	0.881805	-3.66189	-2.30304
H	-1.53002	3.60851	1.933013
H	-0.88182	3.661892	-2.30304
H	1.530154	3.608396	-1.93298
H	0.88192	3.661897	2.303073
H	-5.72937	2.512584	1.18954
H	-3.80701	4.058353	1.122836
H	-2.02717	1.678372	-1.97058
H	-3.8928	0.123631	-1.85663
H	-3.80703	-4.05822	-1.1229
H	-2.02741	-1.67841	1.970767
H	-3.89301	-0.12365	1.856767
H	-5.72941	-2.51242	-1.18965
H	3.807004	-4.05837	1.122818
H	5.729373	-2.51261	1.189542
H	3.807038	4.0582	-1.12292
H	5.729404	2.512394	-1.18965
H	3.892997	0.123667	1.856788
H	2.027396	1.678434	1.970767
H	3.89282	-0.12362	-1.85661
H	2.027187	-1.67835	-1.97058

2ketones-[1,1][4]PCP

C	-5.75577	1.321813	-1.24498
C	-4.80825	2.328564	-1.25171
C	-4.7151	3.23524	-0.19261
C	-5.70143	3.196489	0.794867
C	-6.6835	2.213687	0.776814
C	-6.67846	1.220194	-0.204
H	-5.72945	0.588094	-2.03884
H	-4.05855	2.345676	-2.03567
H	-5.67616	3.914987	1.608198
H	-7.42484	2.166321	1.567265
C	-3.45973	4.016781	-0.06572
C	-2.72715	3.92043	1.117775
C	-2.85375	4.63707	-1.16151
C	-1.39091	4.286681	1.157258
H	-3.17199	3.44097	1.983892
C	-1.52063	5.016832	-1.1163
H	-3.41922	4.778396	-2.07751
C	-0.74225	4.773684	0.019951
H	-0.81839	4.079644	2.055161
H	-1.06518	5.459853	-1.99637
C	0.742126	4.773713	-0.01986
C	1.390822	4.286854	-1.15721
C	1.52049	5.01677	1.116428
C	2.727069	3.920625	-1.11774
H	0.818322	4.079903	-2.05515
C	2.853611	4.637026	1.161621
H	1.065012	5.459694	1.99653
C	3.45962	4.016858	0.065778
H	3.171938	3.441272	-1.9839
H	3.419069	4.778258	2.077642
C	4.714976	3.23529	0.192642
C	4.807987	2.32842	1.251585
C	5.70143	3.196715	-0.79472
C	5.755511	1.321679	1.244806
H	4.058175	2.345387	2.035443
C	6.683511	2.213925	-0.77671
H	5.676249	3.915349	-1.60794
C	6.678358	1.220269	0.203945
H	5.729075	0.587805	2.038513
H	7.424964	2.166704	-1.56706
C	-6.20371	-1.78774	-1.25029
C	-5.21409	-2.75693	-1.19388
C	-4.77727	-3.27534	0.029202

C	-5.49444	-2.91713	1.173946
C	-6.47841	-1.94122	1.121676
C	-6.78343	-1.3026	-0.07989
H	-6.48379	-1.37838	-2.21473
H	-4.70904	-3.03924	-2.11135
H	-5.24485	-3.36315	2.130957
H	-6.97122	-1.61996	2.033415
C	-3.47298	-3.98128	0.11376
C	-2.93847	-4.71552	-0.95001
C	-2.6407	-3.73189	1.208187
C	-1.59365	-5.05777	-0.97851
H	-3.56931	-4.9875	-1.79037
C	-1.30324	-4.09016	1.188983
H	-3.01047	-3.15195	2.046545
C	-0.73907	-4.69579	0.065631
H	-1.19208	-5.57667	-1.84355
H	-0.66211	-3.79111	2.011971
C	0.739204	-4.69578	-0.06557
C	1.593798	-5.05774	0.978554
C	1.303352	-4.09014	-1.18893
C	2.938622	-4.71548	0.950032
H	1.192252	-5.57665	1.843607
C	2.640803	-3.73186	-1.20816
H	0.662202	-3.7911	-2.01191
C	3.473108	-3.98124	-0.11375
H	3.569477	-4.98746	1.790383
H	3.010547	-3.15191	-2.04653
C	4.777396	-3.27529	-0.02923
C	5.49448	-2.91701	-1.17401
C	5.214305	-2.75694	1.193841
C	6.478418	-1.94107	-1.12177
H	5.244842	-3.363	-2.13102
C	6.203903	-1.78772	1.250228
H	4.70933	-3.03931	2.111339
C	6.78351	-1.3025	0.079808
H	6.971138	-1.61974	-2.03354
H	6.484053	-1.3784	2.214664
C	7.53556	0.002394	-0.03726
C	-7.53557	0.002247	0.037176
O	8.640671	0.079029	-0.51484
O	-8.6407	0.07879	0.514722

2ketones-[1,1][2]PCP

C	1.170473	2.443971	-1.32123
C	1.769425	1.199161	-1.28012
C	2.376331	0.736258	-0.1073

C	2.587641	1.659532	0.919427
C	1.999681	2.921143	0.873201
C	1.186326	3.275413	-0.20103
C	-2.00023	2.921371	-0.87309
C	-2.58812	1.659721	-0.91905
C	-2.37654	0.736575	0.107727
C	-1.7696	1.199708	1.28044
C	-1.1706	2.444505	1.321248
C	-1.1866	3.275742	0.2009
C	-5.3E-05	4.211864	-0.00029
C	-2.37633	-0.73626	-0.1073
C	2.37654	-0.73658	0.107727
C	-1.76943	-1.19916	-1.28012
C	-1.17047	-2.44397	-1.32123
C	-1.18633	-3.27541	-0.20103
C	-1.99968	-2.92114	0.873201
C	-2.58764	-1.65953	0.919427
C	2.58812	-1.65972	-0.91905
C	2.000226	-2.92137	-0.87309
C	1.186598	-3.27574	0.2009
C	1.170595	-2.44451	1.321248
C	1.769598	-1.19971	1.28044
C	0.000053	-4.21186	-0.00029
H	0.567411	2.708506	-2.18371
H	1.618293	0.517667	-2.11045
H	3.124719	1.35856	1.813605
H	2.075073	3.578091	1.734378
H	-2.07574	3.578179	-1.73436
H	-3.12526	1.358577	-1.81314
H	-1.61848	0.518412	2.110929
H	-0.56729	2.709125	2.183532
H	-1.61829	-0.51767	-2.11045
H	-0.56741	-2.70851	-2.18371
H	-2.07507	-3.57809	1.734378
H	-3.12472	-1.35856	1.813605
H	3.125259	-1.35858	-1.81314
H	2.07574	-3.57818	-1.73436
H	0.56729	-2.70913	2.183532
H	1.618482	-0.51841	2.110929
O	0.000053	5.409267	-0.00072
O	-5.3E-05	-5.40927	-0.00072

diol-2Me-[1,1][2]PCP

C	-3.31345	1.183813	0.133145
C	-2.90103	1.907693	-0.98277
C	-1.64232	2.495284	-1.0099

C	-0.76031	2.347172	0.065139
C	-1.28162	1.832237	1.256467
C	-2.54362	1.263388	1.291156
C	-1.64239	-2.49526	1.009927
C	-0.76041	-2.34723	-0.06522
C	-1.28176	-1.83241	-1.2565
C	-2.54381	-1.26351	-1.29115
C	-3.31352	-1.18379	-0.13315
C	-2.90103	-1.90763	0.982837
C	-4.28051	0.000045	0.000011
C	3.291497	-1.16503	0.168326
C	2.480597	-1.18433	1.304949
C	1.218265	-1.74645	1.276191
C	0.716393	-2.33636	0.111989
C	1.621569	-2.5437	-0.93008
C	2.888686	-1.96723	-0.9002
C	1.218423	1.746478	-1.27627
C	0.716495	2.33633	-0.11205
C	1.621646	2.543692	0.930028
C	2.888785	1.967247	0.90016
C	3.29162	1.165043	-0.16834
C	2.480759	1.184384	-1.30502
C	4.298899	-3.1E-05	0.000033
C	5.208793	0.162167	1.223164
C	5.20887	-0.16229	-1.22302
O	-5.07296	0.203695	-1.13674
O	-5.07294	-0.20349	1.136787
H	-5.55512	-0.61155	-1.298
H	-5.55479	0.611896	1.298221
H	5.806898	1.073774	1.13688
H	5.893601	-0.68686	1.28765
H	4.66715	0.211402	2.168098
H	4.667341	-0.21096	-2.16805
H	-3.5175	1.908169	-1.87406
H	-1.30256	2.972924	-1.92376
H	-0.63725	1.726024	2.122834
H	-2.86491	0.732839	2.181024
H	-1.30257	-2.97291	1.923758
H	-0.63749	-1.7262	-2.12294
H	-2.86504	-0.73292	-2.18102
H	-3.51749	-1.90812	1.874136
H	2.755923	-0.60873	2.181211
H	0.559914	-1.5846	2.122967
H	1.305381	-3.0733	-1.82393
H	3.515112	-2.06027	-1.78086
H	0.560094	1.584642	-2.12306
H	1.305447	3.073295	1.82387

2diols-[1,1][4]PCP			
C	-5.16908	2.02968	1.34891
C	-4.88949	2.90913	0.29688
C	-5.83566	3.01605	-0.725
C	-6.9308	2.16205	-0.78195
C	-7.12153	1.19936	0.20261
C	-6.27089	1.19442	1.30612
C	-8.04678	-0.00006	0.00005
C	-7.12149	-1.19945	-0.20254
C	-6.93072	-2.16215	0.782
C	-5.83556	-3.01612	0.72502
C	-4.8894	-2.90915	-0.29687
C	-5.16903	-2.02969	-1.34888
C	-6.27086	-1.19446	-1.30607
C	-3.53586	3.50995	0.18504
C	-3.53576	-3.50995	-0.18506
C	-2.75024	3.80469	1.30276
C	-1.38602	4.03041	1.18064
C	-0.75778	3.98849	-0.06609
C	-1.56975	3.84292	-1.1934
C	-2.92624	3.59733	-1.06988
C	-2.9261	-3.59734	1.06984
C	-1.56961	-3.84291	1.19333
C	-0.75767	-3.98846	0.066
C	-1.38593	-4.03036	-1.18072
C	-2.75015	-3.80465	-1.3028
C	5.47699	-2.51535	-1.04346
C	4.88219	-2.81827	0.18369
C	5.54861	-2.42754	1.34722
C	6.676	-1.62364	1.28429
C	7.17865	-1.2066	0.0557
C	6.61237	-1.7203	-1.10777
C	8.1156	0.00005	0.00006
C	7.17855	1.20663	-0.05563
C	6.6122	1.7203	1.10781
C	5.47682	2.51533	1.04346
C	4.88207	2.81828	-0.18371
C	5.54855	2.42755	-1.34721
C	6.67596	1.62367	-1.28424
C	0.71813	-3.89251	0.18406
C	3.48954	3.32694	-0.24522
C	2.97336	4.30053	0.61307
C	1.61151	4.57986	0.64154
C	0.71802	3.89255	-0.18417
C	1.24727	2.95994	-1.07998
C	2.60127	2.68488	-1.11113
C	1.24737	-2.95991	1.07989
C	2.60138	-2.68486	1.11105

C	3.48965	-3.32692	0.24515	H	-3.196	-3.80043	-2.29231
C	2.97347	-4.30049	-0.61316	H	4.98943	-2.82053	-1.96417
C	1.61162	-4.57981	-0.64165	H	5.1425	-2.70201	2.31558
O	-8.84476	-0.25865	1.1249	H	7.12392	-1.25208	2.19945
O	-8.84481	0.25849	-1.12478	H	7.01377	-1.42526	-2.07005
O	8.91308	0.1222	1.14657	H	7.01352	1.42523	2.07013
O	8.91318	-0.12207	-1.1464	H	4.98921	2.82049	1.96416
H	-4.46003	1.91813	2.16204	H	5.14249	2.70204	-2.31559
H	-5.68883	3.73649	-1.52332	H	7.12388	1.25211	-2.19939
H	-7.60647	2.20005	-1.62765	H	3.64233	4.83987	1.27693
H	-6.41744	0.45729	2.08851	H	1.23806	5.33631	1.32518
H	-7.60638	-2.20019	1.6277	H	0.57919	2.37895	-1.70636
H	-5.6887	-3.73657	1.52333	H	2.96507	1.8956	-1.76027
H	-4.45999	-1.91811	-2.16202	H	0.57929	-2.37894	1.70628
H	-6.41744	-0.45732	-2.08843	H	2.96517	-1.89559	1.76021
H	-3.19606	3.80048	2.29228	H	3.64245	-4.83983	-1.27702
H	-0.78914	4.15915	2.07827	H	1.23817	-5.33625	-1.3253
H	-1.12355	3.86101	-2.18244	H	9.41719	0.69243	-1.23083
H	-3.50141	3.38428	-1.96444	H	-9.31985	0.55062	1.3298
H	-3.50126	-3.3843	1.96442	H	-9.31978	-0.55083	-1.32975
H	-1.12338	-3.86099	2.18236	H	9.41712	-0.69228	1.23105
H	-0.78907	-4.15907	-2.07836				

8. References

[1] E. Akiyama, S. Nishimura, T. Suzuki, Y. Nagase, *Trans. Mater. Res. Soc. Jpn.* **2012**, *37*, 459-462.

[2] H. Mansilla, M. M. Afonso, *Synth. Commun.* **2008**, *38*, 2607-2618.

[3] Y. Yan, P. Wang, Y. Wang, J. Dong, G. Li, C. Wang, D. Xue, *Org. Lett.* **2024**, *26*, 1370-1375.

[4] (a) Sheldrick, G. SHELXT - Integrated space-group and crystal-structure determination. *Acta Crystallog*r. A **2015**, A71, 3-8; (b) Sheldrick, G. Crystal structure refinement with SHELXL. *Acta Crystallogr.* **2015**, C71, 3-8; (c) Dolomanov, O. V.; Bourhis, L. J.; Gildea, R. J.; Howard, J. A. K.; Puschmann, H. OLEX2: a complete structure solution, refinement and analysis program. *J. Appl. Crystallogr.* **2009**, *42*, 339-341.

[5] Frisch, M. J.; Trucks, G. W.; Schlegel, H. B.; Scuseria, G. E.; Robb, M. A.; Cheeseman, J. R.; Scalmani, G.; Barone, V.; Petersson, G. A.; Nakatsuji, H.; Li, X.; Caricato, M.; Marenich, A. V.; Bloino, J.; Janesko, B. G.; Gomperts, R.; Mennucci, B.; Hratchian, H. P.; Ortiz, J. V.; Izmaylov, A. F.; Sonnenberg, J. L.;Williams; Ding, F.; Lipparini, F.; Egidi, F.; Goings, J.; Peng,

B.; Petrone, A.; Henderson, T.; Ranasinghe, D.; Zakrzewski, V. G.; Gao, J.; Rega, N.; Zheng, G.; Liang, W.; Hada, M.; Ehara, M.; Toyota, K.; Fukuda, R.; Hasegawa, J.; Ishida, M.; Nakajima, T.; Honda, Y.; Kitao, O.; Nakai, H.; Vreven, T.; Throssell, K.; Montgomery Jr., J. A.; Peralta, J. E.; Ogliaro, F.; Bearpark, M. J.; Heyd, J. J.; Brothers, E. N.; Kudin, K. N.; Staroverov, V. N.; Keith, T. A.; Kobayashi, R.; Normand, J.; Raghavachari, K.; Rendell, A. P.; Burant, J. C.; Iyengar, S. S.; Tomasi, J.; Cossi, M.; Millam, J. M.; Klene, M.; Adamo, C.; Cammi, R.; Ochterski, J. W.; Martin, R. L.; Morokuma, K.; Farkas, O.; Foresman, J. B.; Fox, D. J. Gaussian 16 Rev. C.01, Wallingford, CT, **2016**.

[6] W. Humphrey, A. Dalke, K. Schulten, *J. Mol. Graph.* **1996**, *14*, 33-38.

[7] T. Lu, F. Chen, *J. Comput. Chem.* **2012**, *33*, 580-592.

[8] T. Lu, *J. Chem. Phys.* **2024**, *161*, 082503.

[9] C. E. Colwell, T. W. Price, T. Stauch, R. Jasti, *Chem. Sci.* **2020**, *11*, 3923-3930.

[10] Y. Segawa, H. Omachi, K. Itami, *Org. Lett.* **2010**, *12*, 2262-2265.

[11] S. M. Bachrach, D. Stück, *J. Org. Chem.* **2010**, *75*, 6595-6604.

[12] T. Iwamoto, Y. Watanabe, Y. Sakamoto, T. Suzuki, S. Yamago, *J. Am. Chem. Soc.* **2011**, *133*, 8354-8361.
